# Supplementary material for: Pyro-catalysis for tooth whitening via oral temperature fluctuation
Source: Nat Commun. 2022 Jul 29;13:4419. doi: 10.1038/s41467-022-32132-3 (PMC9338087; doi:10.1038/s41467-022-32132-3)
Supplement: Supplementary file 1 — Supporting-Pyro-catalysis for tooth whitening via oral temperature fluctuation [file 41467_2022_32132_MOESM1_ESM.pdf]

Supplementary information for

Pyro-catalysis for tooth whitening via oral temperature fluctuation

Yang Wang[1], Shuhao Wang[1], Yanze Meng[2], Zhen Liu[1], Dijie Li[1], Yunyang Bai[3], Guoliang Yuan[1], Yaojin Wang[1,*], Xuehui Zhang[2,4,*], Xiaoguang Li[5] and Xuliang Deng[3,4,*]

[1]School of Materials Science and Engineering, Nanjing University of Science and Technology, Nanjing 210094, Jiangsu, China.

[2]Department of Dental Materials & Dental Medical Devices Testing Center, Peking University School and Hospital of Stomatology, Beijing 100081, China.

[3]Department of Geriatric Dentistry, Peking University School and Hospital of Stomatology, Beijing 100081, China.

[4]National Engineering Research Center of Oral Biomaterials and Digital Medical Devices, NMPA Key Laboratory for Dental Materials, Beijing Laboratory of Biomedical Materials & Beijing Key Laboratory of Digital Stomatology, Peking University School and Hospital of Stomatology, Beijing 100081, PR China.

[5]Hefei National Laboratory for Physical Sciences at the Microscale, Department of Physics, and CAS Key Laboratory of Strongly-Coupled Quantum Matter Physics University of Science and Technology of China Hefei 230026, China.

Supplementary Table 1. Comparison of highly effective tooth whitening methods

Tooth Whitening Methods	Materials	Required Physical Field	Damage to Enamel	Shortages
**Commercial Gels**	Peroxide	None	Erosion	Gingival irritation, mineral loss, and tooth hypersensitivity
**Photo-catalysis**	Photo-catalyst (TiO_2)	Blue light	None	Photo-toxic, photo-allergic
**Piezo-catalysis**	Piezo-catalyst ($BaTiO_3$)	Vibration	None	Unavailable intrinsic physical stimuli

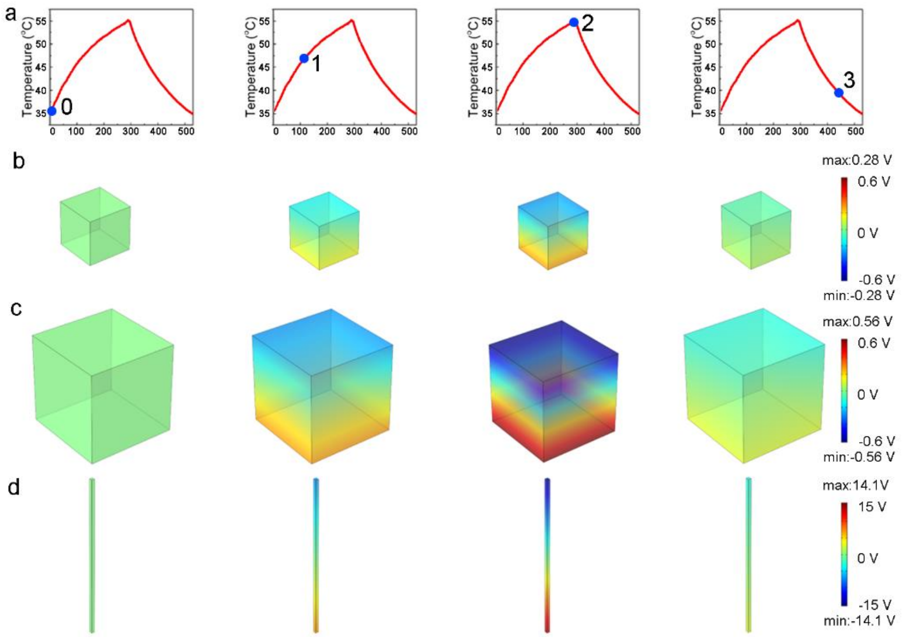

Supplementary Fig. 1 **a** Temperature fluctuations used in the simulation, and the corresponding pyroelectric potential of BTO nanocrystals in the form of **b** nanoparticle (100 nm×100 nm×100 nm), **c** nanoparticle (200 nm×200 nm×200 nm), and **d** nanowire (100 nm×100 nm×5000 nm).

The pyroelectric potential generated by BTO nanomaterials with different morphologies was simulated by Comsol Multiphysics. The crystal polarization is set along *z*-axis, and the constitutive relation is described by

$$D = P_s + p(T - T_0) + \varepsilon_0 \varepsilon_r E$$

where T_0 is the initial temperature, D is the electric displacement vector, ε_0 is the permittivity of vacuum, ε_r is the permittivity of BTO, P_s is the spontaneous polarization at T_0 with a value of 0.25 C m^{-2}, p denotes the pyroelectric coefficient which is 210 μC m^{-2} K^{-1}, and E is the internal electric field. The other parameters used in this simulation are predefined parameters in Comsol Multiphysics. It can be seen from the results that the pyroelectric potential is distributed along the polar axis. The maximum pyroelectric potential was observed when the largest temperature variation was reached. The comparison of pyroelectric potentials generated by BTO nanomaterials with different morphologies reveals that the pyroelectric potential generated by BTO nanowires is significantly increased, and impressively, the BTO nanowires generated a pyroelectric potential of 28.2 V with a length of 5 μm in this simulation.

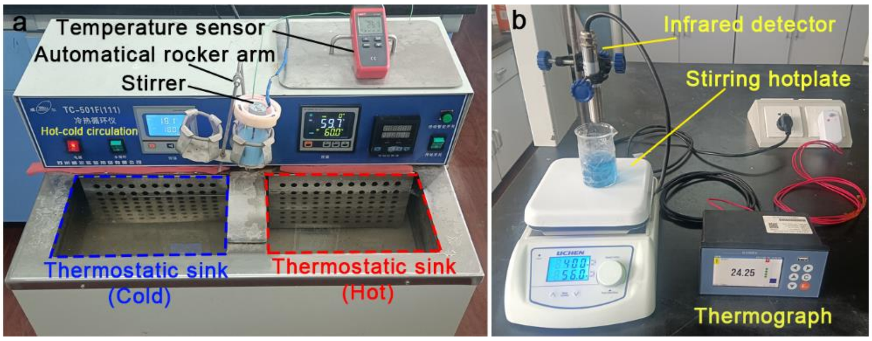

Supplementary Fig. 2 **a** Automatic heating-cooling circulation equipment for pyro-catalytic degradation and **b** stirring hotplate with infrared temperature recording system.

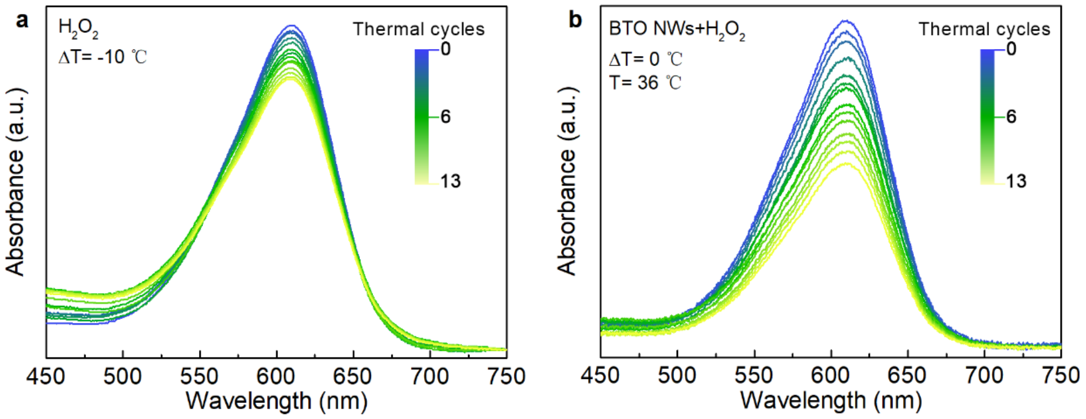

Supplementary Fig. 3 UV-Vis absorption spectra of Indigo Carmine solutions using **a** H_2O_2 at ΔT= -10 °C, **b** BTO NWs with H_2O_2 in 36 °C insulation without thermal cycling. Source data are provided as a Source Data file.

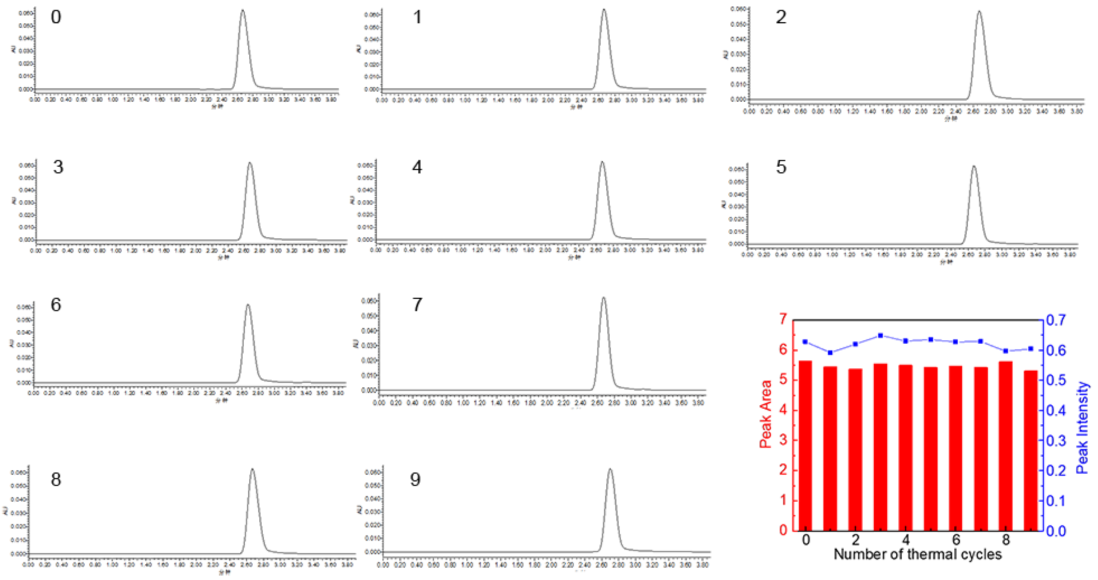

Supplementary Fig. 4 Concentration change of hydrogen peroxide with increasing number of cycles. Source data are provided as a Source Data file.

Hydrogen peroxide was present throughout the catalytic experiments. In order to verify that the hydrogen peroxide was only present as a co-catalyst throughout the experiments and did not have any degradation effect on the organic matter, liquid chromatography was used to detect the concentration change of hydrogen peroxide in the solution after each thermal cycle. The concentration of hydrogen peroxide in the solution after each thermal cycle was examined for temperature fluctuations of +5 ℃. Both the peak intensity and the peak area of the characteristic peaks of hydrogen peroxide in the spectra after each thermal cycle remained at a constant level throughout the catalytic process. It means that the hydrogen peroxide did not actually participate in the degradation of the organic matter during the whole catalytic process, but was only present as a co-catalyst. This result further confirmed the degradation of organic matter was attributed to the pyroelectric properties of the catalyst. Source data are provided as a Source Data file.

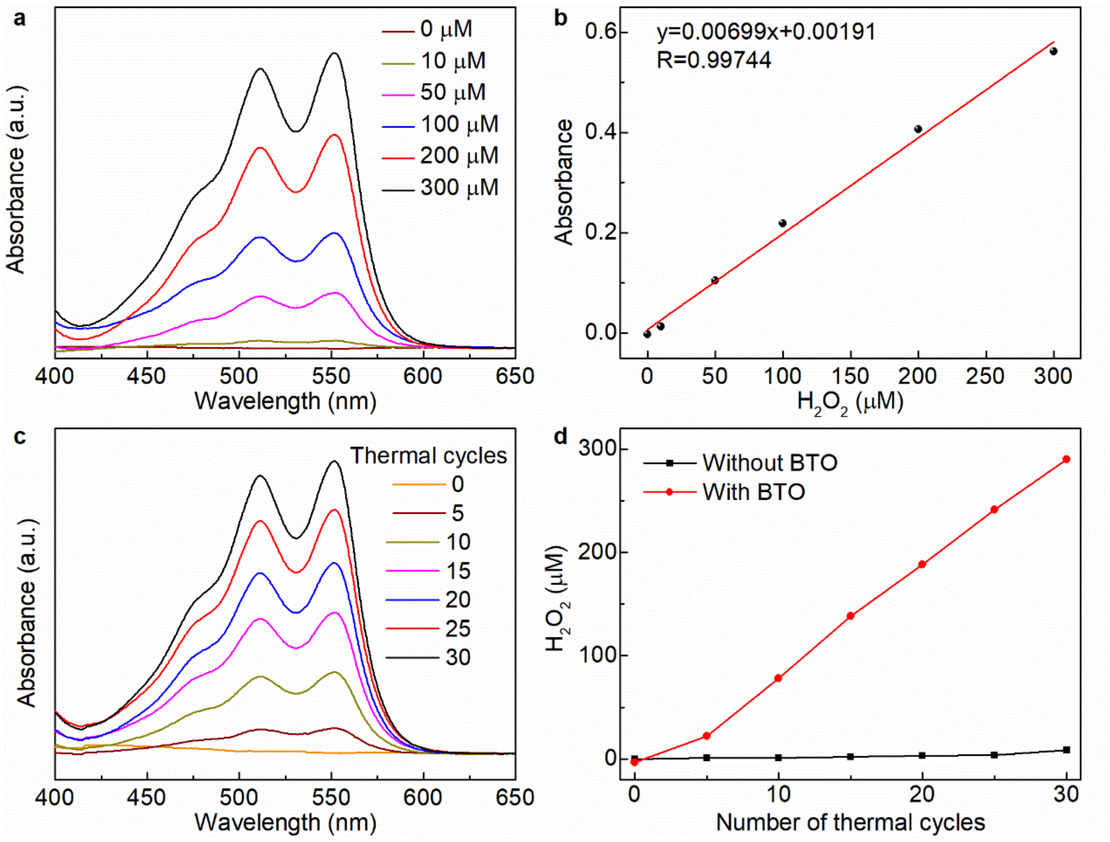

Supplementary Fig. 5 a Typical absorption spectra obtained by testing different concentrations of H_2O_2 using the DPD-POD method. **b** The linear relationship between the H_2O_2 concentration and the peak of the absorption spectrum at 551 nm can be used to calculate the H_2O_2 concentration in the unknown solution. **c** the absorption spectra of H_2O_2 concentrations created by BTO during pyro-catalysis. **d** The concentration of H_2O_2 in the reaction system with and without BTO after a specific number of thermal cycles. Source data are provided as a Source Data file.

H_2O_2 detection. The concentration of H_2O_2 created during pyro-catalysis process was measured by a DPD−POD method. Briefly, 50mg of BTO was dispersed in 50ml of water, and after a certain number of thermal cycles, samples of the liquid were centrifuged and 1mL of the supernatant was added to a mixture of 3 mL of phosphate buffer (0.5 M, pH = 6), 5.9 mL of water, 0.05 mL of N,N-diethyl-p-phenylenediamine sulfate (DPD, 10 mg mL^{-1}) and 0.05 mL of peroxidase (POD, 1 mg mL^{-1}). After 30 s, the H_2O_2 concentration was measured at 551 nm on a UV−vis spectrophotometer.

DPD-POD method was further employed to verify the role of small amount addition of H_2O_2 during pyro-catalysis process[1, 2]. First, the linear relationship between the peak of the absorption spectrum at 551 nm and concentrations of added H_2O_2 indicates the validity of the DPD-POD method, as shown in Supplementary Fig. 4 a and b. It can be seen that The generation of H_2O_2 increase with the number of thermal cycles and about 300 μM of H_2O_2 was produced in the reaction system after 30 hot and cold cycles at a temperature fluctuation of +20 °C, as shown in Supplementary Fig. 4 a and b.

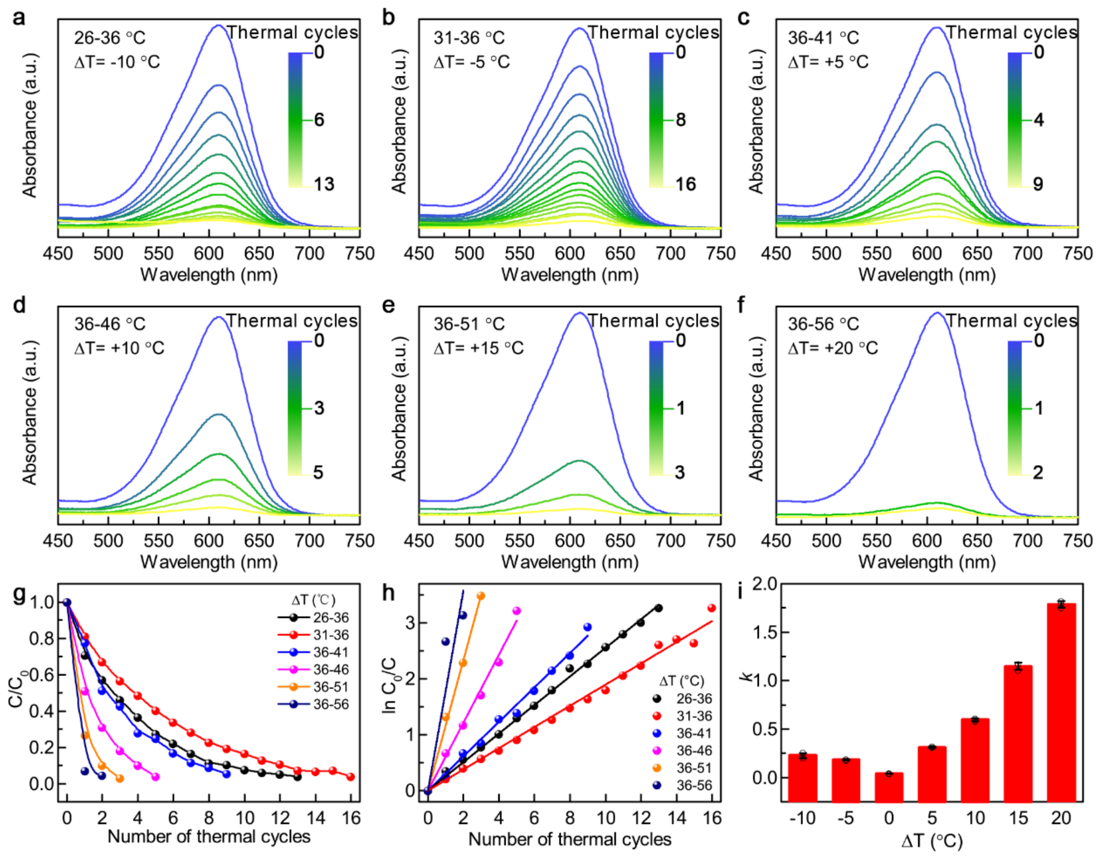

Supplementary Fig. 6 UV-Vis absorption spectra of Indigo Carmine solutions with respect to temperature vibration range **a-f** *ΔT*= -10, -5, +5, +10, +15, +20 ℃. Pyro-catalytic degradation efficiency performance of PMN-PT powders in **g** direct concentration ratio C/C_0 and **h** the pseudo-first-order reaction kinetics of different temperature fluctuations. **i** The kinetic rate constant for the Indigo Carmine solutions pyroelectric degradation reaction performed with different temperature fluctuations (n=3). Data are presented as mean values ± SD. Source data are provided as a Source Data file.

PMN-PT single crystal powder with high pyroelectric properties was used for comparative pyro-catalytic degradation experiment. Single crystals were first crushed and then poled using corona poling method[3]. The same degradation of indigo carmine using PMN-PT powder was performed as BTO nanowires at different temperature fluctuation. The results show that PMN-PT single crystal powder required fewer thermal cycles and the organic dyes can be degraded more completely, which is consistent with previous reports[4].

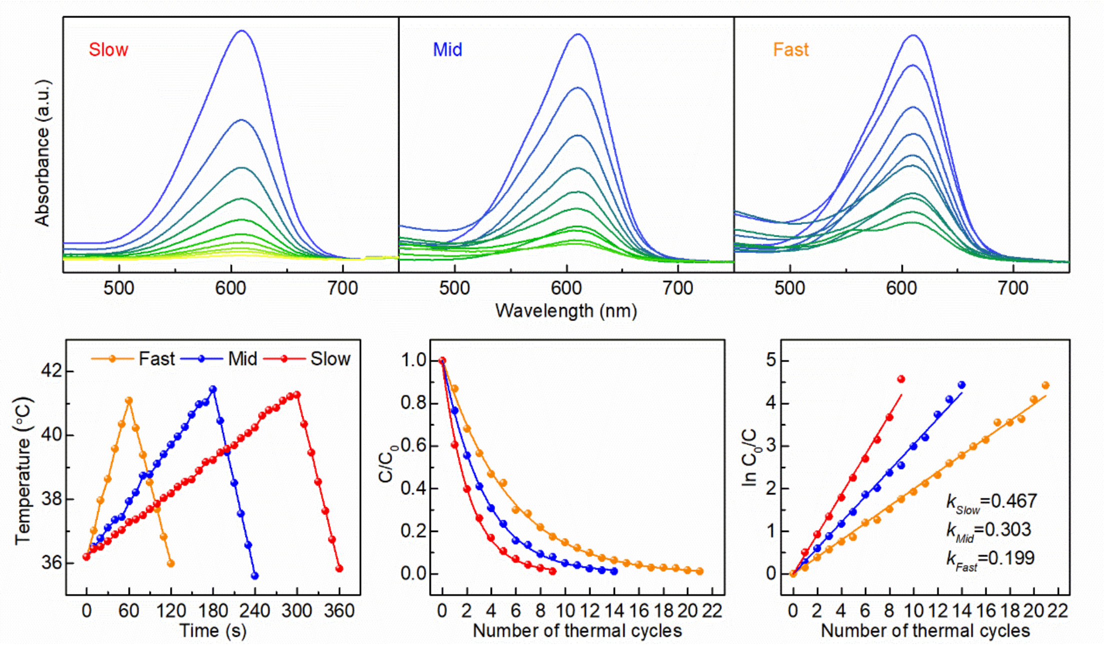

Supplementary Fig. 7 UV-Vis absorption spectra of Indigo Carmine solutions using different heating rate **a** slow (1 °C min^{-1}), **b** mid (1.7 °C min^{-1}) and **c** fast (5 °C min^{-1}) under the same cooling rate (5 °C min^{-1}). **d** Temperature change record by infrared temperature detector. **e** Direct concentration ratio C/C_0 and **f** the pseudo-first-order reaction kinetics of different heating rate. Source data are provided as a Source Data file.

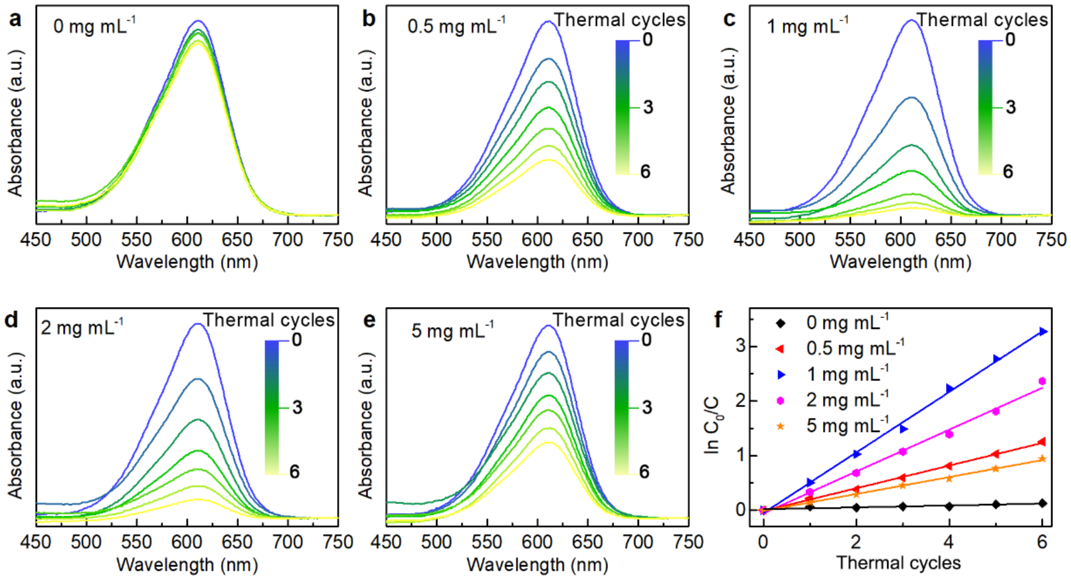

Supplementary Fig. 8 a-e UV-Vis absorption spectra of Indigo Carmine solutions with respect to BTO concentration. **f** The pseudo-first-order reaction kinetics of different pyro-catalyst concentration. Source data are provided as a Source Data file.

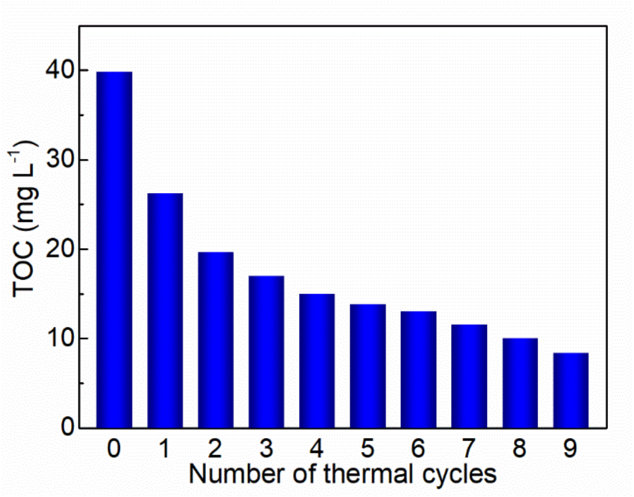

Supplementary Fig. 9 Total organic carbon (TOC) removal during Indigo Carmine pyro-catalysis. Source data are provided as a Source Data file.

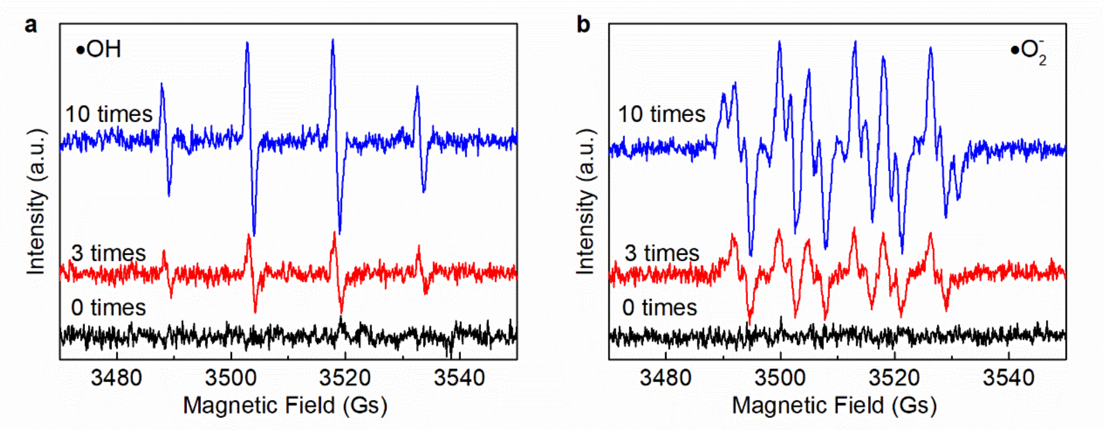

Supplementary Fig. 10 Electron paramagnetic resonance spectra (EPR) of radical **a** •OH and **b** $•O_2^-$ created by pyro-catalysis over different cycling times. Source data are provided as a Source Data file.

In the EPR spectrum of DMPO-$•O_2^-$, a typical peak belonging to DMPO-$•CH_3$ was found. This is due to the fact that DMSO is used as an •OH trapping agent when testing $•O_2^-$, but at the same time, DMSO is also oxidized by OH to produce CH_3 that cannot be produced by DMPO, and the reaction process can be expressed by the following equations[5, 6]:

$$(CH_3)_2SO + •OH \rightarrow •CH_3 + CH_3(OH)SO \quad (1)$$

$$•CH_3 + O_2 \rightarrow CH_3OO• \quad (2)$$

$$DMPO + CH_3OO• \rightarrow •DMPO\text{-}OOCH_3 \quad (3)$$

$$2•DMPO\text{-}OOCH_3 \rightarrow O_2 + 2•DMPO\text{-}OCH_3 \quad (4)$$

$$2CH_3OO• \rightarrow 2CH_3O• + O_2 \quad (5)$$

$$DMPO + CH_3O• \rightarrow •DMPO\text{-}OCH_3 \quad (6)$$

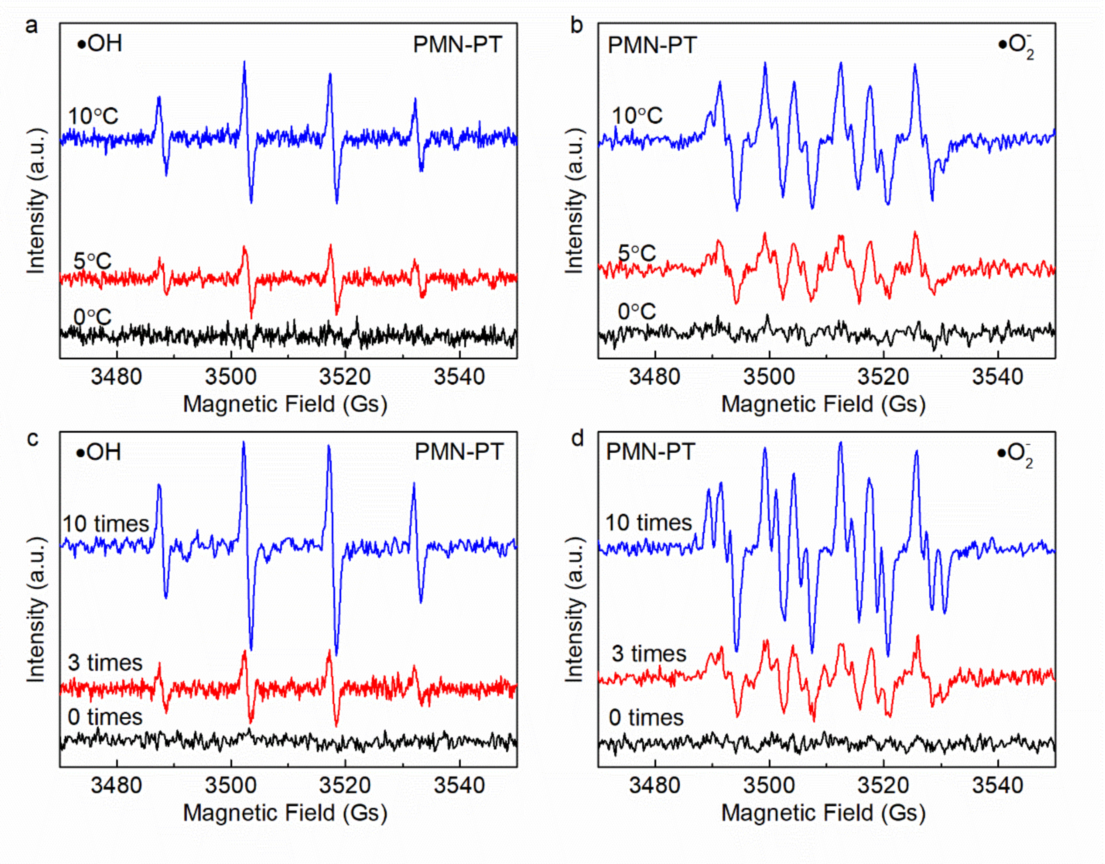

Supplementary Fig. 11 Electron paramagnetic resonance spectra (EPR) of radical •OH and $•O_2^-$ created by PMN-PT over **a-b** different temperature range and **c-d** different cycling times. Source data are provided as a Source Data file.

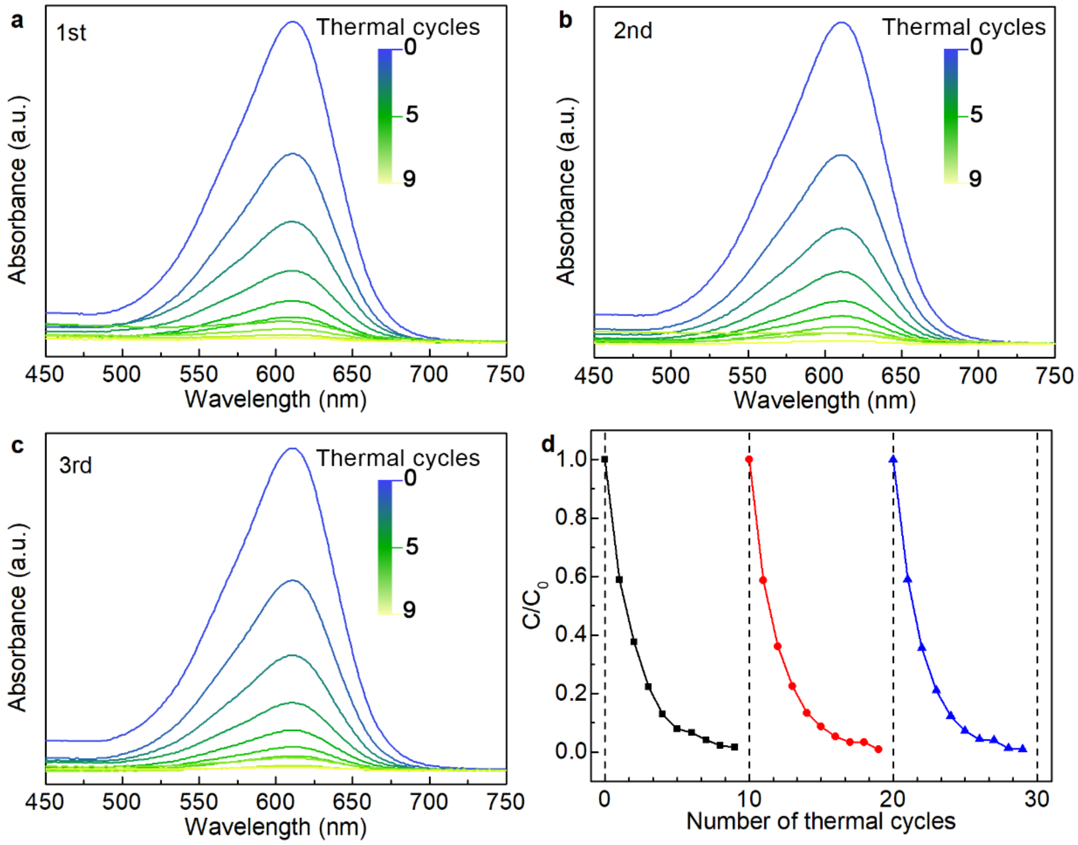

Supplementary Fig. 12 a-c UV-Vis absorption spectra of Indigo Carmine solutions with artificial saliva as solvent using the same BTO nanowires for three cycles. **b** Cyclic stability of BTO nanowires degraded indigo solution. Source data are provided as a Source Data file.

Artificial saliva (purchased from Shanghai yuanye Bio-Technology Co., Ltd) was used to simulate the real environment of human oral cavity, which is mainly composed of deionized water, NaCl, KCL, Na_2SO_4, NH_4Cl, $CaCl_2$-$2H_2O$, NaH_2PO_4-$2H_2O$, CN_2H_4O, NaF, and this is more than 99% similar to human saliva. After the indigo carmine was dissolved, pyro-catalytic degradation experiments were performed.

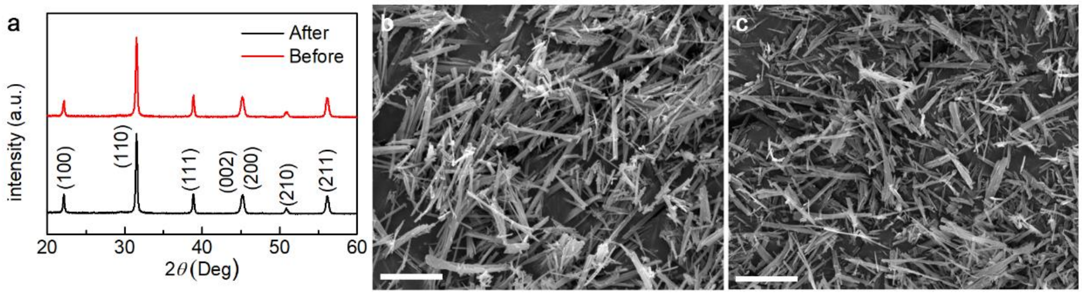

Supplementary Fig. 13 **a** X-ray diffraction pattern of the BTO nanowires before and after pyro-catalysis, and scanning electron microscope image of BTO nanowires **b** before and **c** after pyro-catalysis. Scale bars are 5 μm. The experiments in **b-c** were repeated independently for three times with similar results. Source data are provided as a Source Data file.

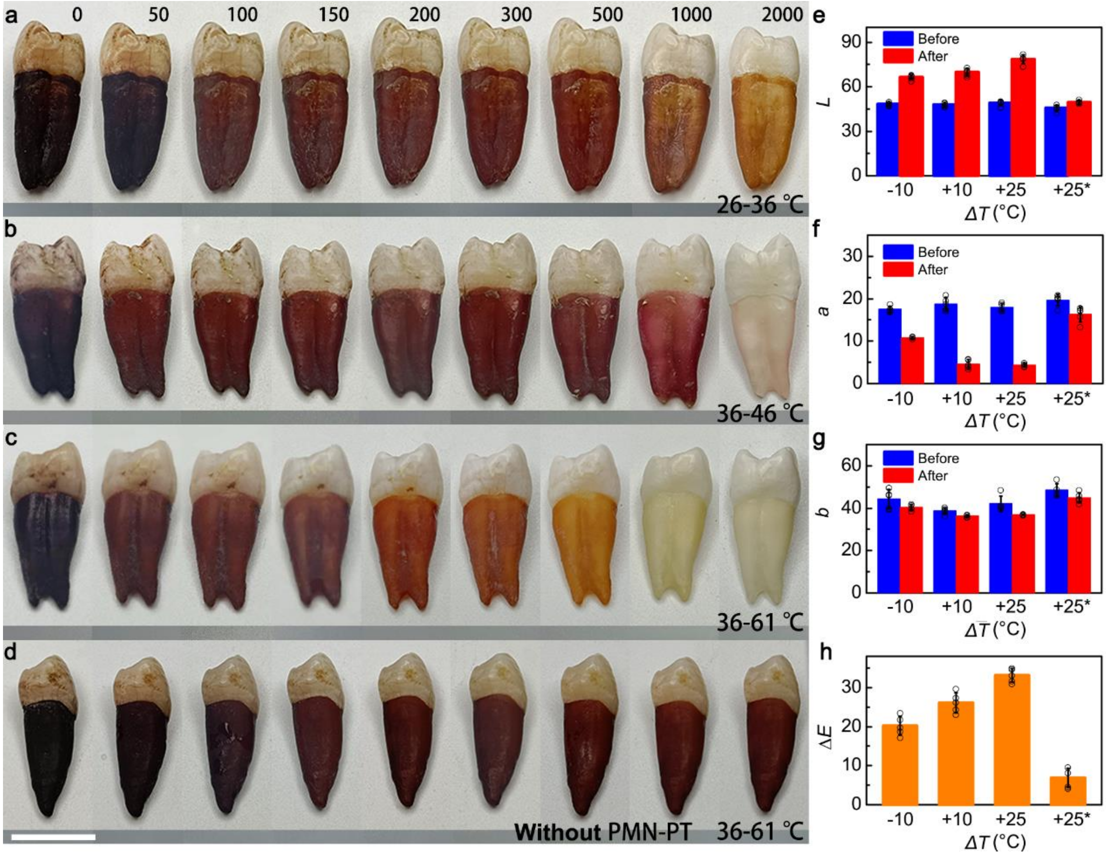

Supplementary Fig. 14 Photographs of teeth under treatment in turbid liquid of PMN-PT powders with different temperature fluctuations **a-c** *ΔT*= -10, +10, +25 ℃, respectively. **d** Photographs of teeth under treatment in pure water with a temperature fluctuation of +25 ℃. Comparison of different temperature fluctuations on the tooth whitening levels demonstrated by CIELab results **e** luminance *L*, **f** color value of red–green axis *a*, **g** color value of blue-yellow axis *b* and **h** color difference Δ*E*. (+25* means without PMN-PT powders). Scale bar is 1 cm. Data are presented as mean values ± SD (n=5). Source data are provided as a Source Data file.

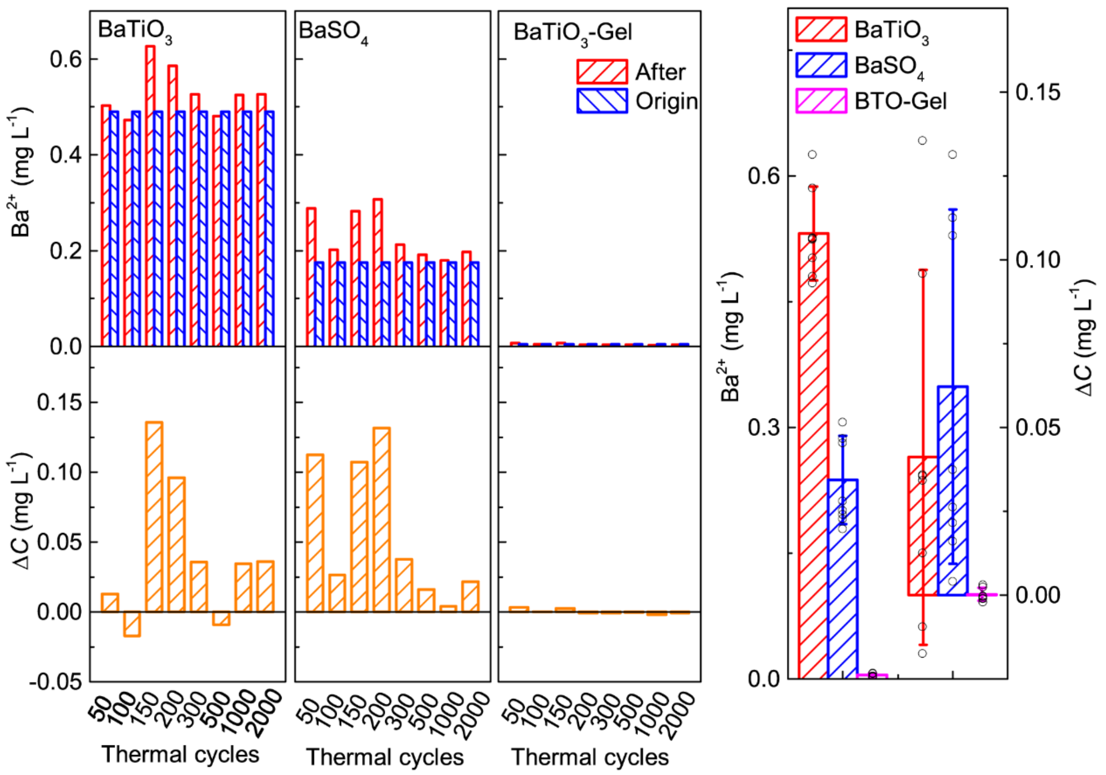

Supplementary Fig. 15 a the concentration of total Ba element (including Ba^{2+} and compound) in the supernatant obtained from BTO, $BaSO_4$ and BTO gel before (blue) and after (red) 2000 thermal cycles; **b** the Ba element concentration difference of before thermal cycles and after 2000 thermal cycles; **c** the concentration of total Ba element and the difference with experimental errors. Data are presented as mean values ± SD (n=8). Source data are provided as a Source Data file.

In order to check the possible leakage of Ba ions during tooth whitening, the same experiment was taken using $BaSO_4$ as comparison, because the barium meal ($BaSO_4$) is safe for gastrointestinal radiological examinations. BTO, $BaSO_4$ and cured BTO gel was put in pure water to get through 2000 thermal cycles. 10 ml suspension was collected after a certain thermal cycle and centrifuged to remove the nanoparticles. The supernatant was collected and this process repeated for three times. The concentration of barium in the supernatant was measured by Inductively Coupled Plasma-Optical Emission Spectroscopy (ICP-OMS). The left column of Supplementary Fig. 15a shows that the barium element was detected in the supernatant of BTO $BaSO_4$ and BTO gel before and after thermal cycles, while the concentration difference of barium element before and after thermal cycles is shown in Supplementary Fig. 15b. The obtained concentration of Ba element and the difference with experimental errors of before and after thermal cycles was given in Supplementary Fig. 15c. The existence of Ba element in the supernatant of BTO and $BaSO_4$ is possible due to the incomplete removal of nanoparticles by centrifugation. This can be confirmed from the results of BTO gel. After the BTO nanowires were compounded with the hydrogel, they cannot be dispersed in the aqueous solution, thus avoiding the possible presence of trace amounts of BTO nanowires in the solution after centrifugation. Therefore, almost invisible Ba

ions were detected in BTO gel. The comparison of the barium concentration difference among BTO, $BaSO_4$ and BTO gel reveals that the barium concentration difference is within the experimental error. Thus, we can claim that the variation of detected barium concentration before and after thermal cycles was due to experimental errors rather than the leakage of Ba^{2+}.

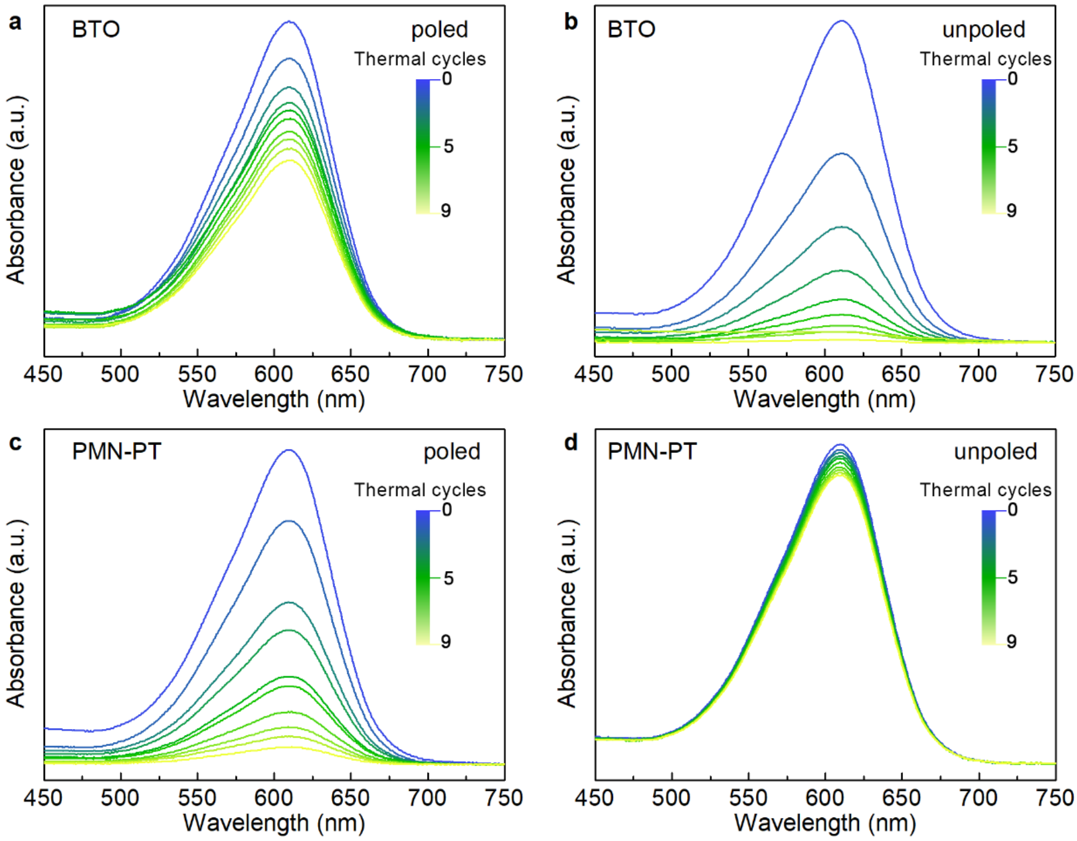

Supplementary Fig. 16 UV-Vis absorption spectra of Indigo Carmine solutions using **a** poled, **b** unpoled BTO and **c** poled and **d** unpoled PMN-PT with a temperature fluctuation of +5 °C. Source data are provided as a Source Data file.

It can be seen the pyro-catalytic performance of the poled BTO nanowires was decreased related to the unpoled BTO nanowires (or as-grown). It is highly possible that the as-grown BTO nanowires are mainly along the [001] direction, which is the highest pyroelectric coefficient direction for the tetragonal BTO. The grown nanowires have highly self-poled along the length direction. However, after being poled using an applied electric field, the polarization along the length direction was disrupted and rearranged leading to a decrease of pyroelectric properties. The same experiment was carried out using PMN-PT single crystal powder. In contrast to BTO nanowires, the catalytic performance of PMN-PT powder after poling was greatly improved for PMN-PT powder with randomly distributed polarization direction. These comparative experiments unambiguous verify that the pyro-catalysis performance is determined by the pyroelectricity (or degree of polarization), which is similar to the piezo-catalysis[3].

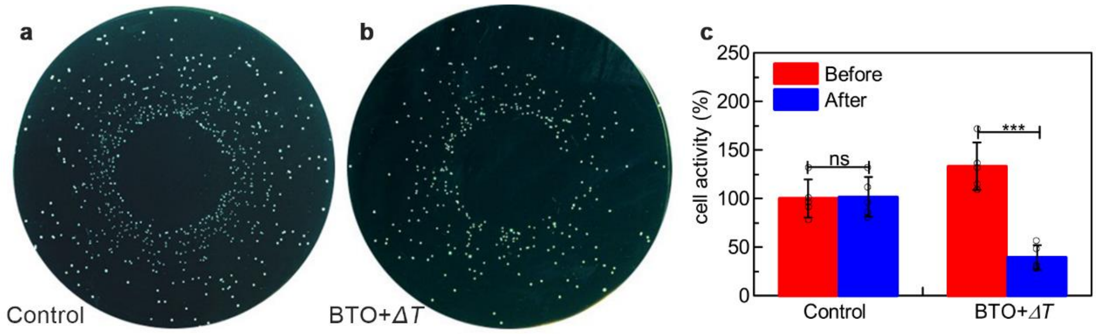

Supplementary Fig. 17 pyro-catalysis for antibacterial activity in vitro. **a-b** Results of spiral inoculation and **c** cell activity before and after pyro-catalysis. Statistical analysis was performed with two-sided Students' t-test, NS = $p > 0.05$ (with the exact p =0.9135), *** = $p < 0.01$ (with the exact p = 6.2388E-05). Data are presented as mean values ± SD (n=5). Source data are provided as a Source Data file.

The pyro-catalytic bacteria sterilization experiment was performed using streptococcus mutans (UA159), which is the culprit of dental plaque and tooth decay. BTO nanowires were first added to EP-tubes for UV sterilization, and then the bacterial solution with an O.D. value of 0.25 at 630 nm was diluted 10,000 times using phosphate buffered saline (PBS). After 400 μl of the diluted solution was added to the sterilized EP-tubes containing BTO, 20 thermal cycles were performed at 20-45 ℃ and each cycle took 18 min. Finally, the bacterial solution was transferred to the agar surface using spiral inoculation and their survival was characterized. The results (Supplementary Fig. 17) reveal that the active radicals released by BTO nanowires through pyro-catalysis have a significant bactericidal effect with only 40% survival.

1. Shi L, *et al.* Photoassisted construction of holey defective g-C_3N_4 photocatalysts for efficient visible-light-driven H_2O_2 production. *Small* **14**, 1703142 (2018).

2. Liu Y, *et al.* Pyroelectric synthesis of metal-$BaTiO_3$ hybrid nanoparticles with enhanced pyrocatalytic performance. *ACS Sustainable Chemistry Engineering* **7**, 2602-2609 (2018).

3. Wang Y, *et al.* Piezo-catalysis for nondestructive tooth whitening. *Nat Commun* **11**, 1328 (2020).

4. You H, Jia Y, Wu Z, Wang F, Huang H, Wang Y. Room-temperature pyro-catalytic hydrogen generation of 2D few-layer black phosphorene under cold-hot alternation. *Nat Commun* **9**, 2889 (2018).

5. Brezová V, Gabčová S, Dvoranová D, Staško A. Reactive oxygen species produced upon photoexcitation of sunscreens containing titanium dioxide (an EPR study). *Journal of Photochemistry and Photobiology B: Biology* **79**, 121-134 (2005).

6. Woodward JR, Lin TS, Sakaguchi Y, Hayashi H. Detection of Transient Intermediates in the

Photochemical Reaction of Hydrogen Peroxide with Dimethyl sulfoxide by Time-Resolved EPR Techniques. *Journal of Physical Chemistry A* **104**, 557-561 (2000).
