# Supplementary figures and images for: Pyro-catalysis for tooth whitening via oral temperature fluctuation (part 2 of 2)
Source: Nat Commun. 2022 Jul 29;13:4419. doi: 10.1038/s41467-022-32132-3 (PMC9338087; doi:10.1038/s41467-022-32132-3)

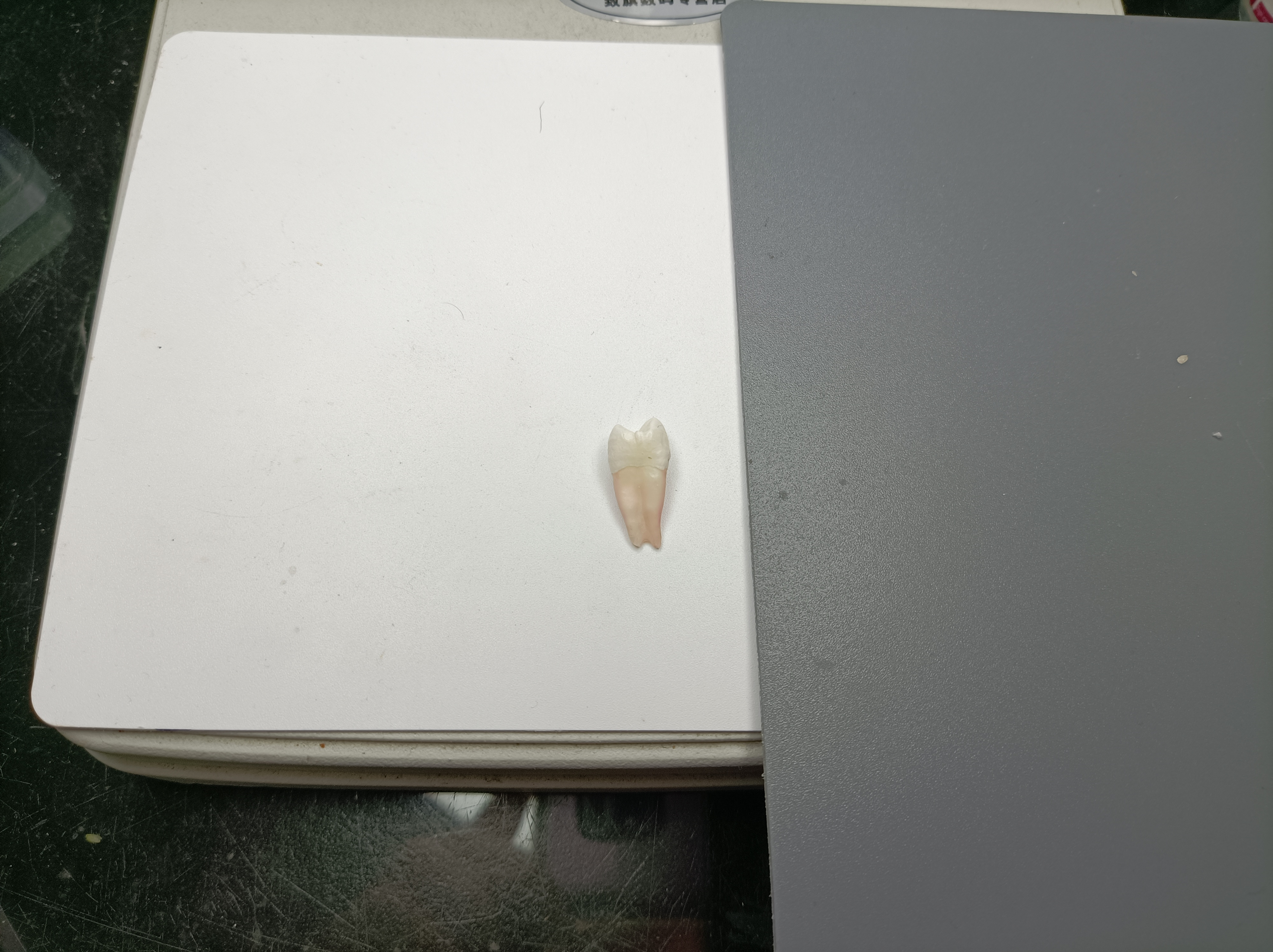

Supplement: Supplementary file 6 — Source data [file 41467_2022_32132_MOESM6_ESM.zip › Source data/supporting/S14/10/2000.jpg]

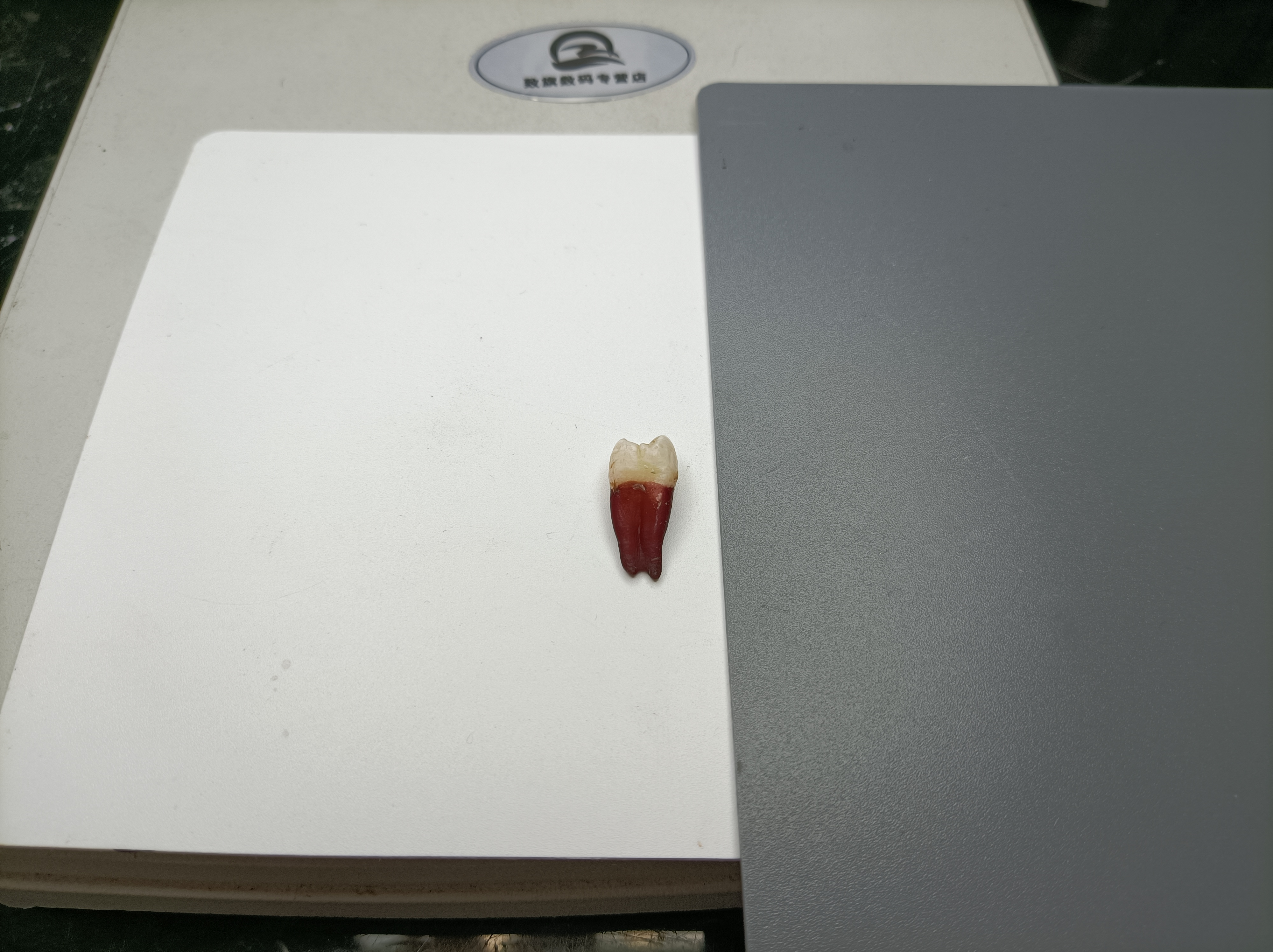

Supplement: Supplementary file 6 — Source data [file 41467_2022_32132_MOESM6_ESM.zip › Source data/supporting/S14/10/300.jpg]

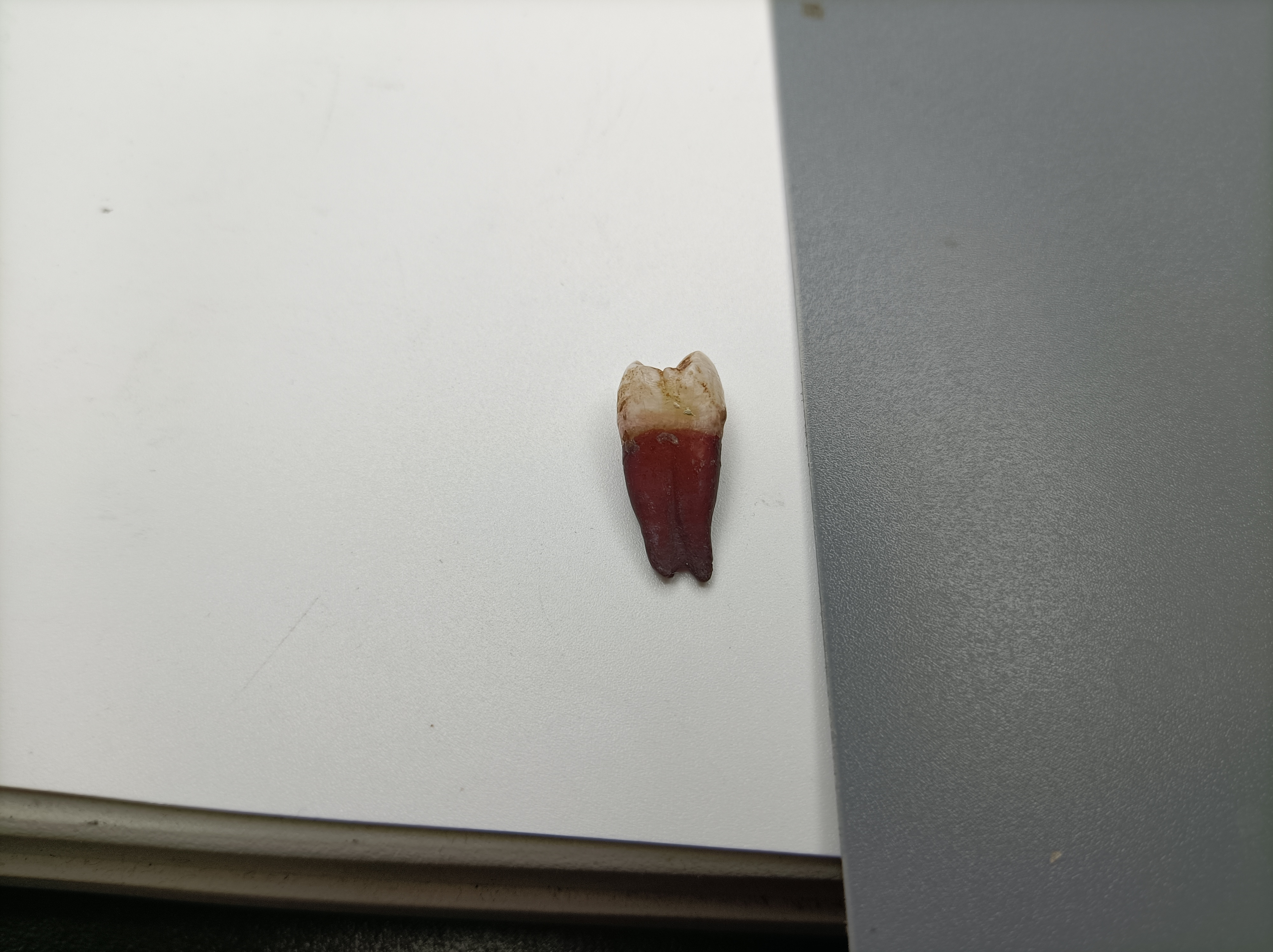

Supplement: Supplementary file 6 — Source data [file 41467_2022_32132_MOESM6_ESM.zip › Source data/supporting/S14/10/50.jpg]

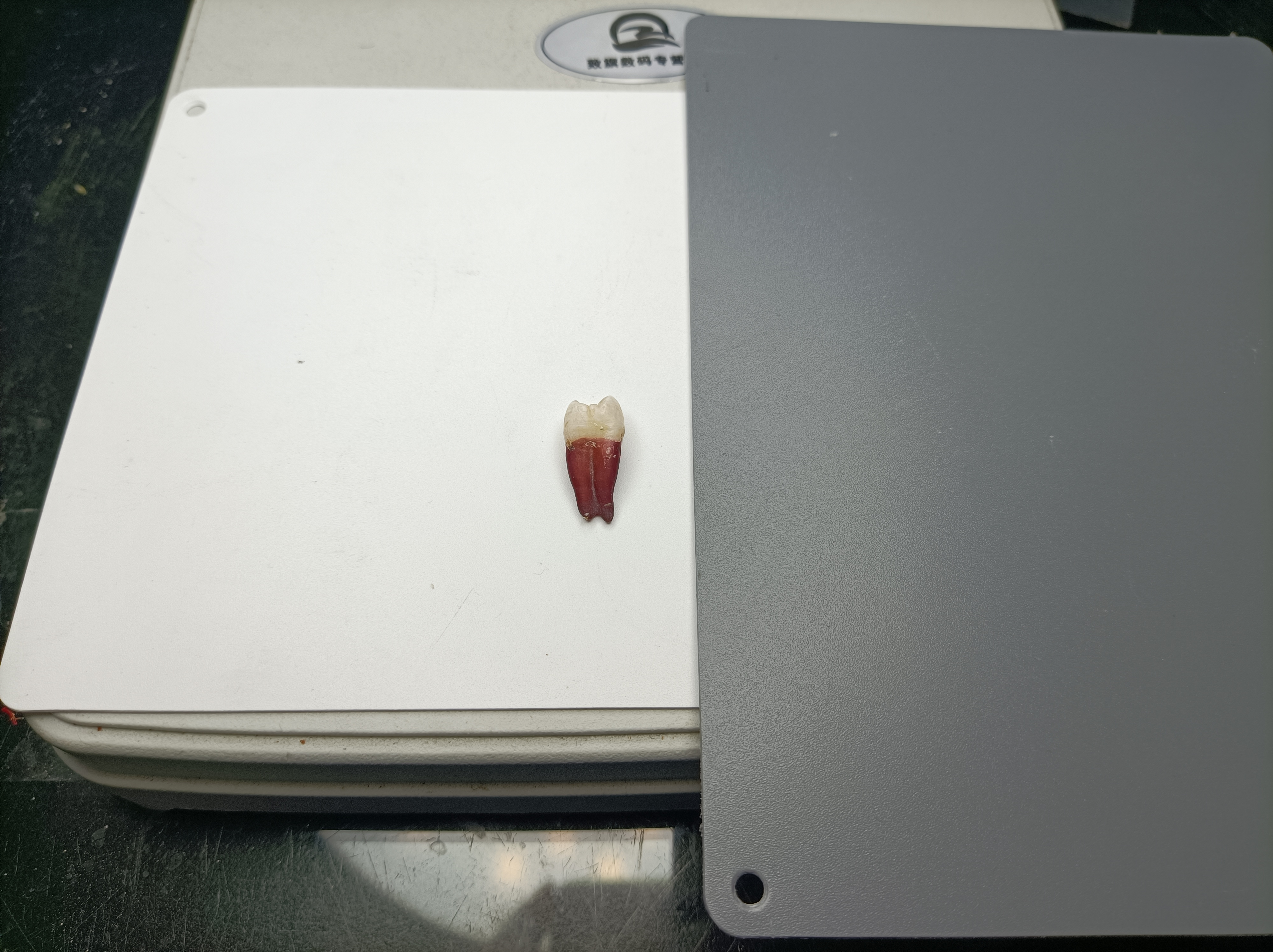

Supplement: Supplementary file 6 — Source data [file 41467_2022_32132_MOESM6_ESM.zip › Source data/supporting/S14/10/500.jpg]

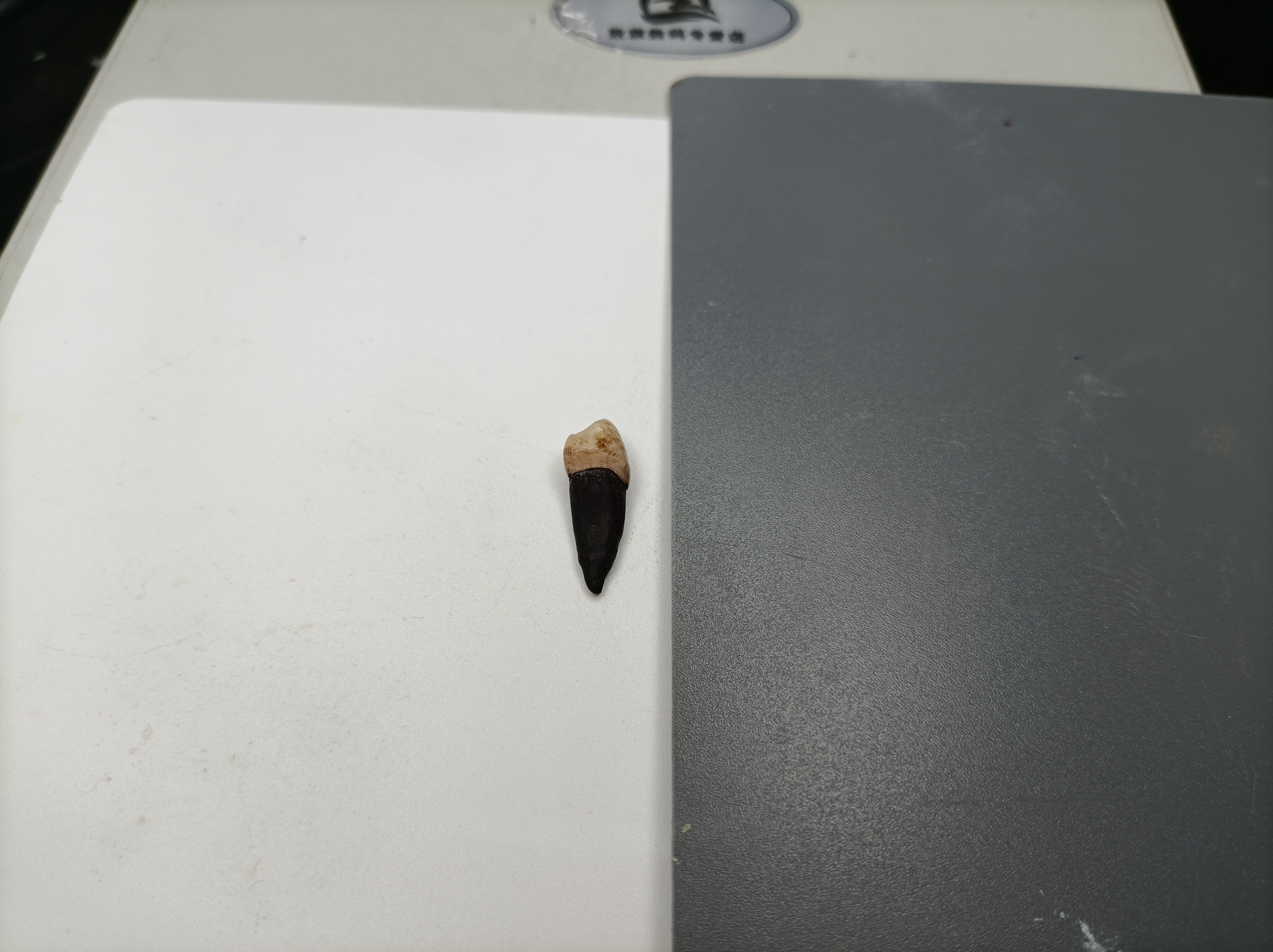

Supplement: Supplementary file 6 — Source data [file 41467_2022_32132_MOESM6_ESM.zip › Source data/supporting/S14/35 without PMNPT/0.jpg]

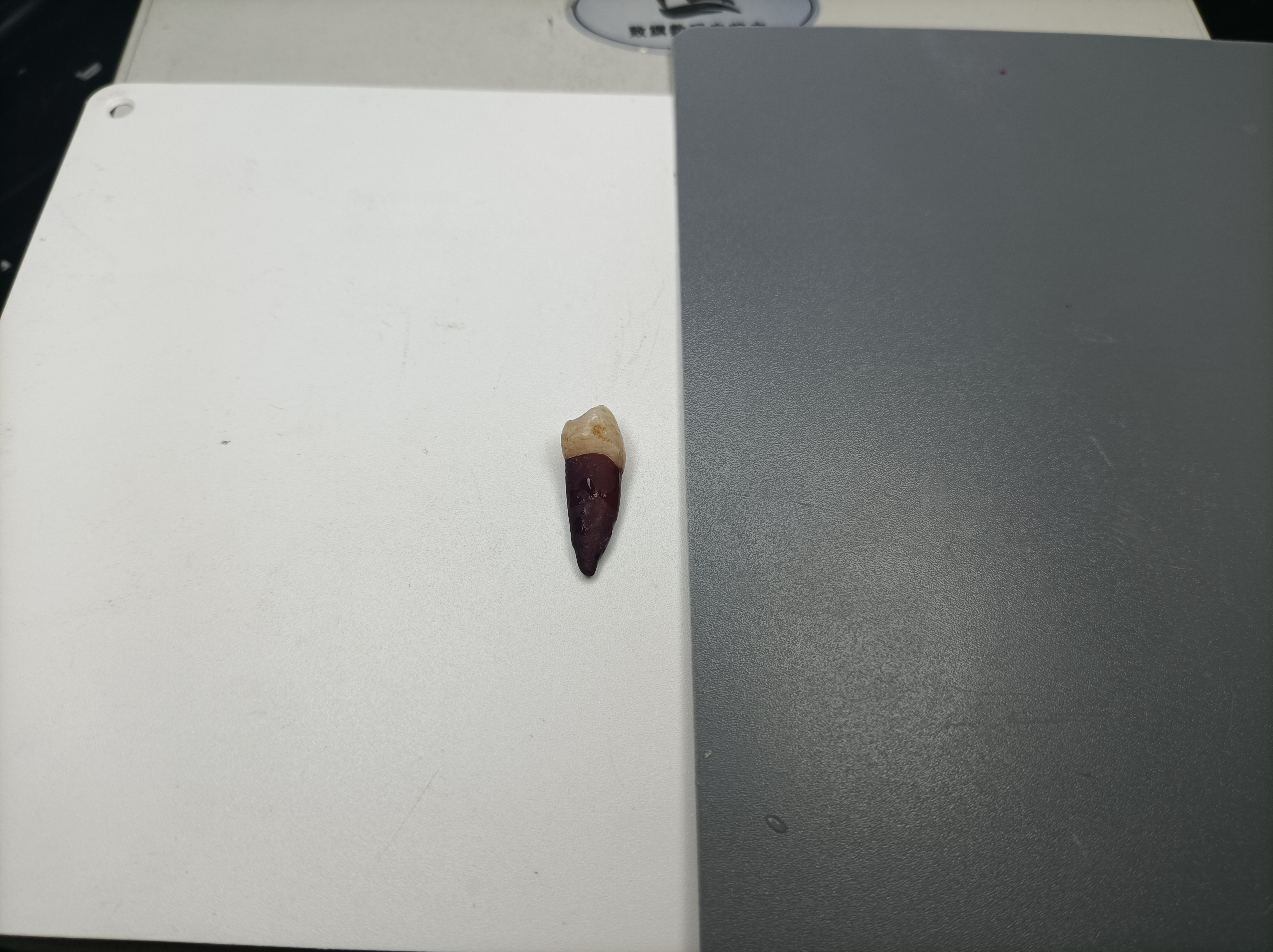

Supplement: Supplementary file 6 — Source data [file 41467_2022_32132_MOESM6_ESM.zip › Source data/supporting/S14/35 without PMNPT/100.jpg]

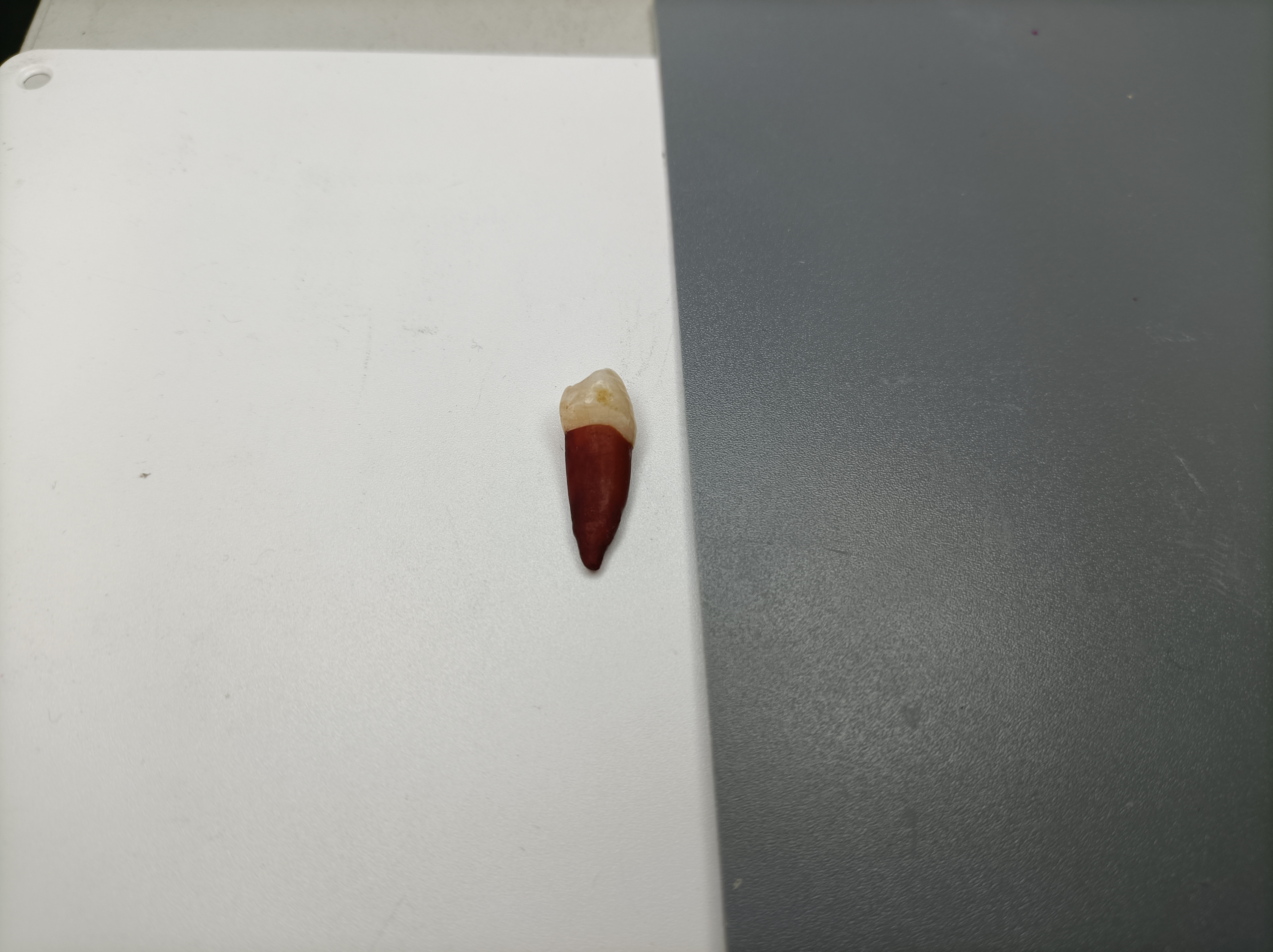

Supplement: Supplementary file 6 — Source data [file 41467_2022_32132_MOESM6_ESM.zip › Source data/supporting/S14/35 without PMNPT/1000.jpg]

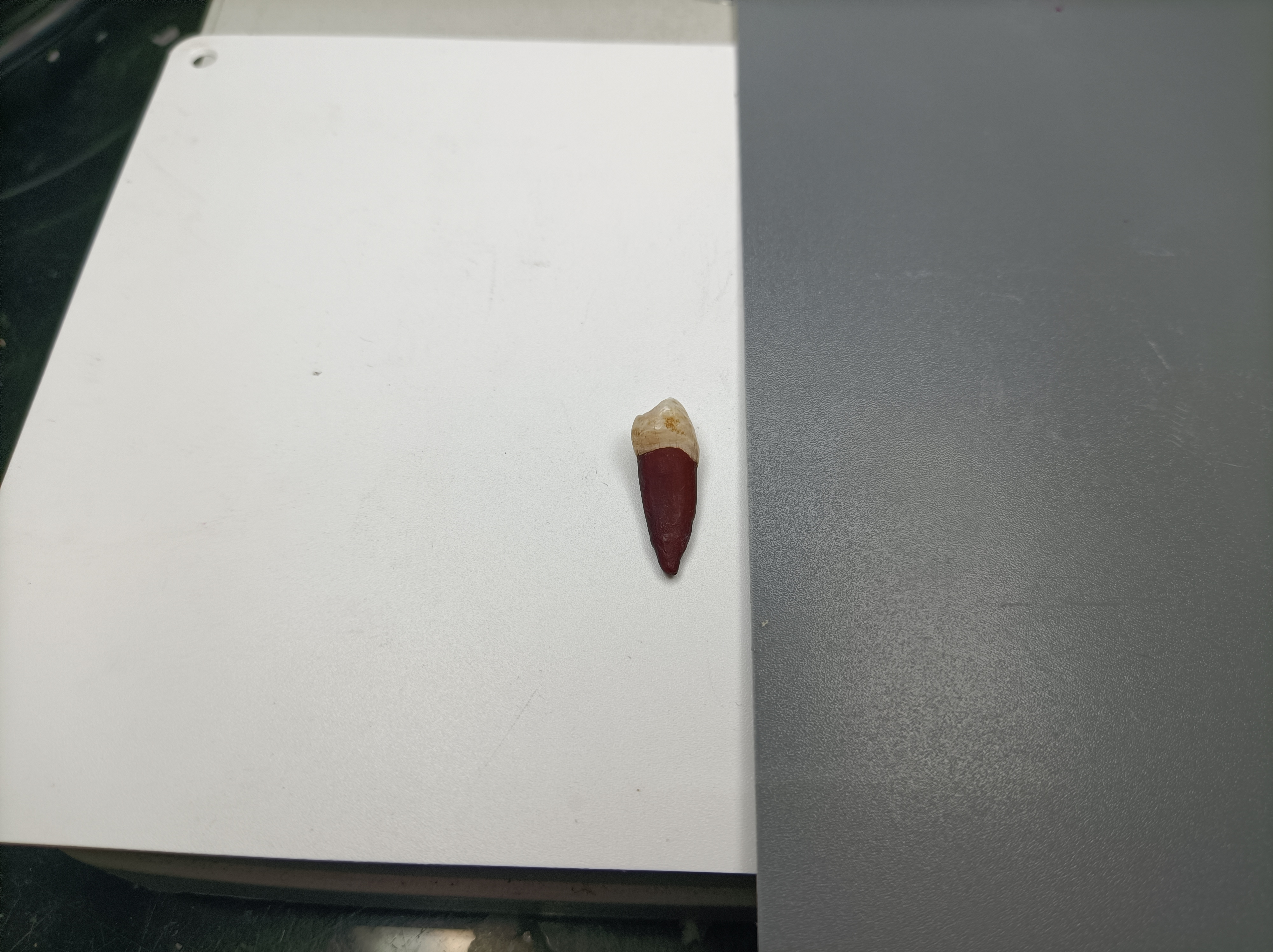

Supplement: Supplementary file 6 — Source data [file 41467_2022_32132_MOESM6_ESM.zip › Source data/supporting/S14/35 without PMNPT/150.jpg]

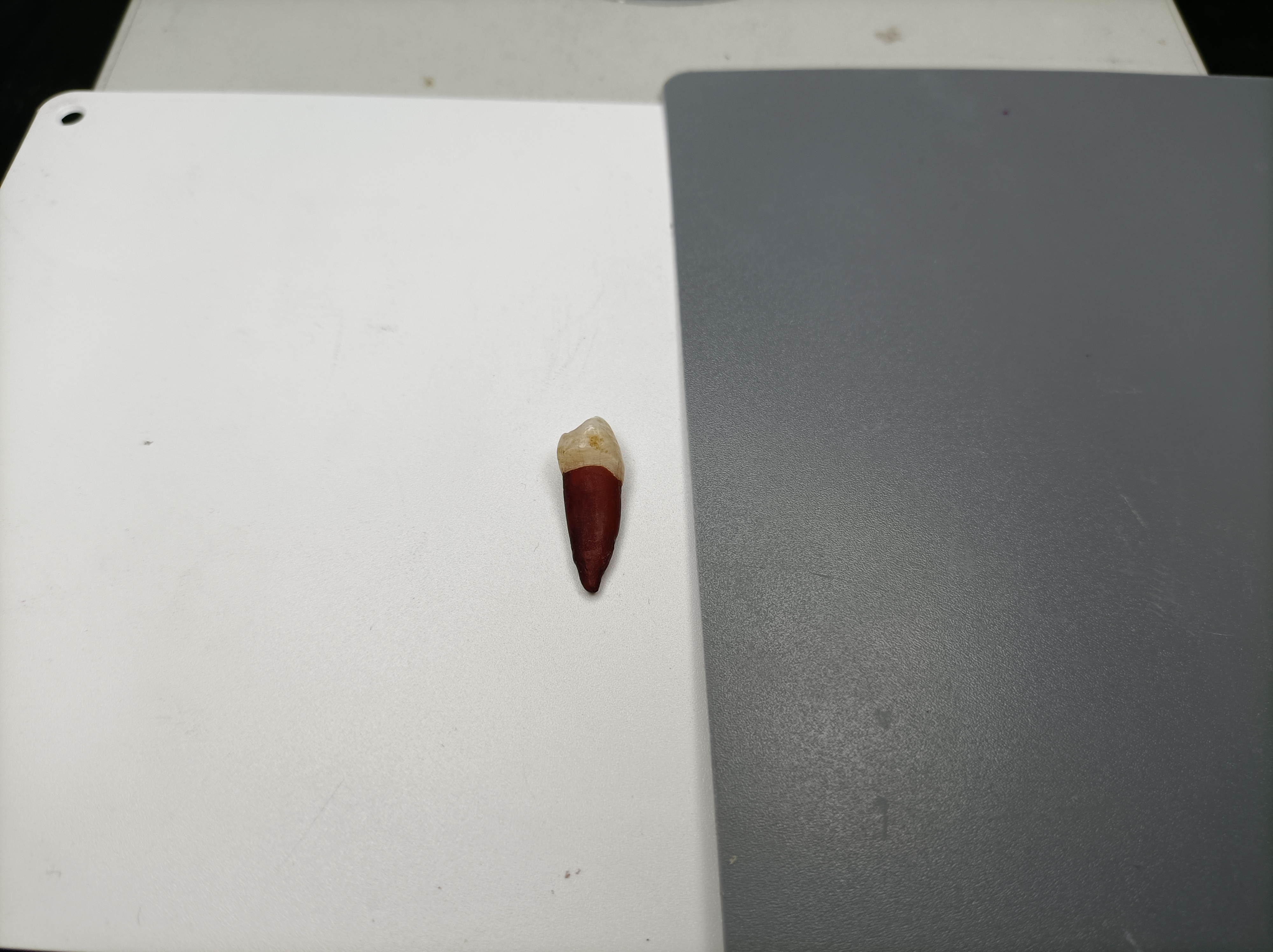

Supplement: Supplementary file 6 — Source data [file 41467_2022_32132_MOESM6_ESM.zip › Source data/supporting/S14/35 without PMNPT/200.jpg]

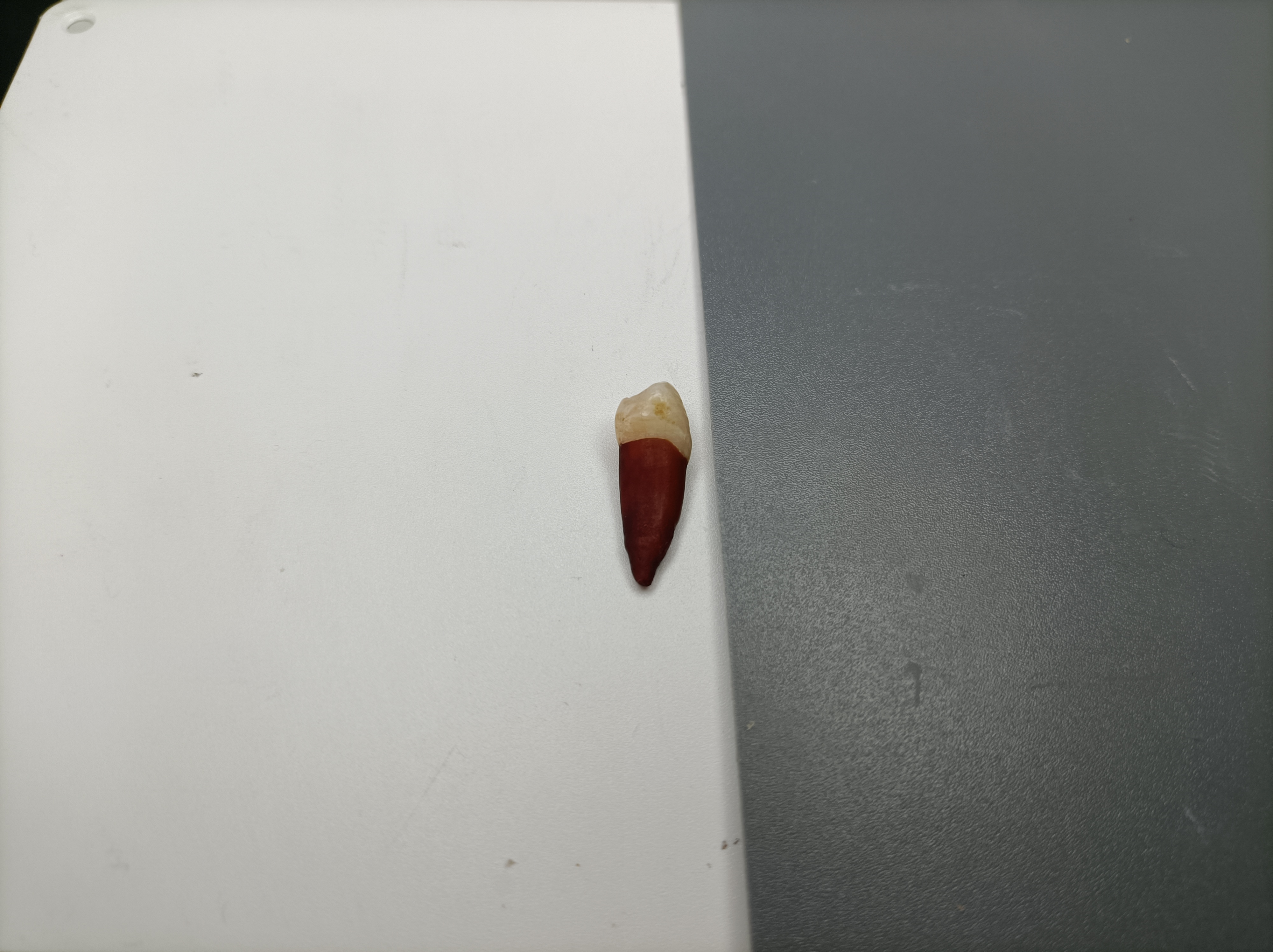

Supplement: Supplementary file 6 — Source data [file 41467_2022_32132_MOESM6_ESM.zip › Source data/supporting/S14/35 without PMNPT/2000.jpg]

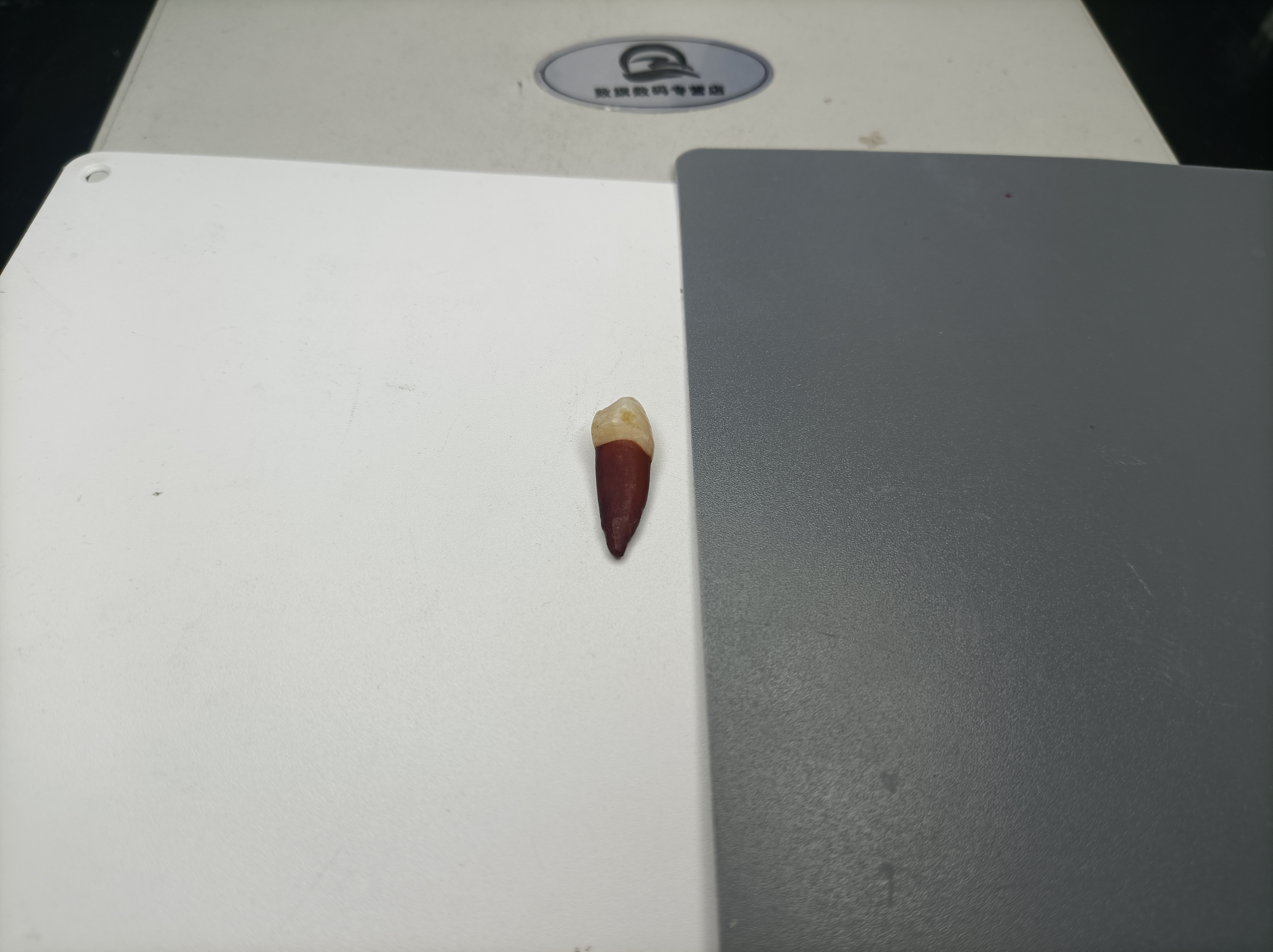

Supplement: Supplementary file 6 — Source data [file 41467_2022_32132_MOESM6_ESM.zip › Source data/supporting/S14/35 without PMNPT/300.jpg]

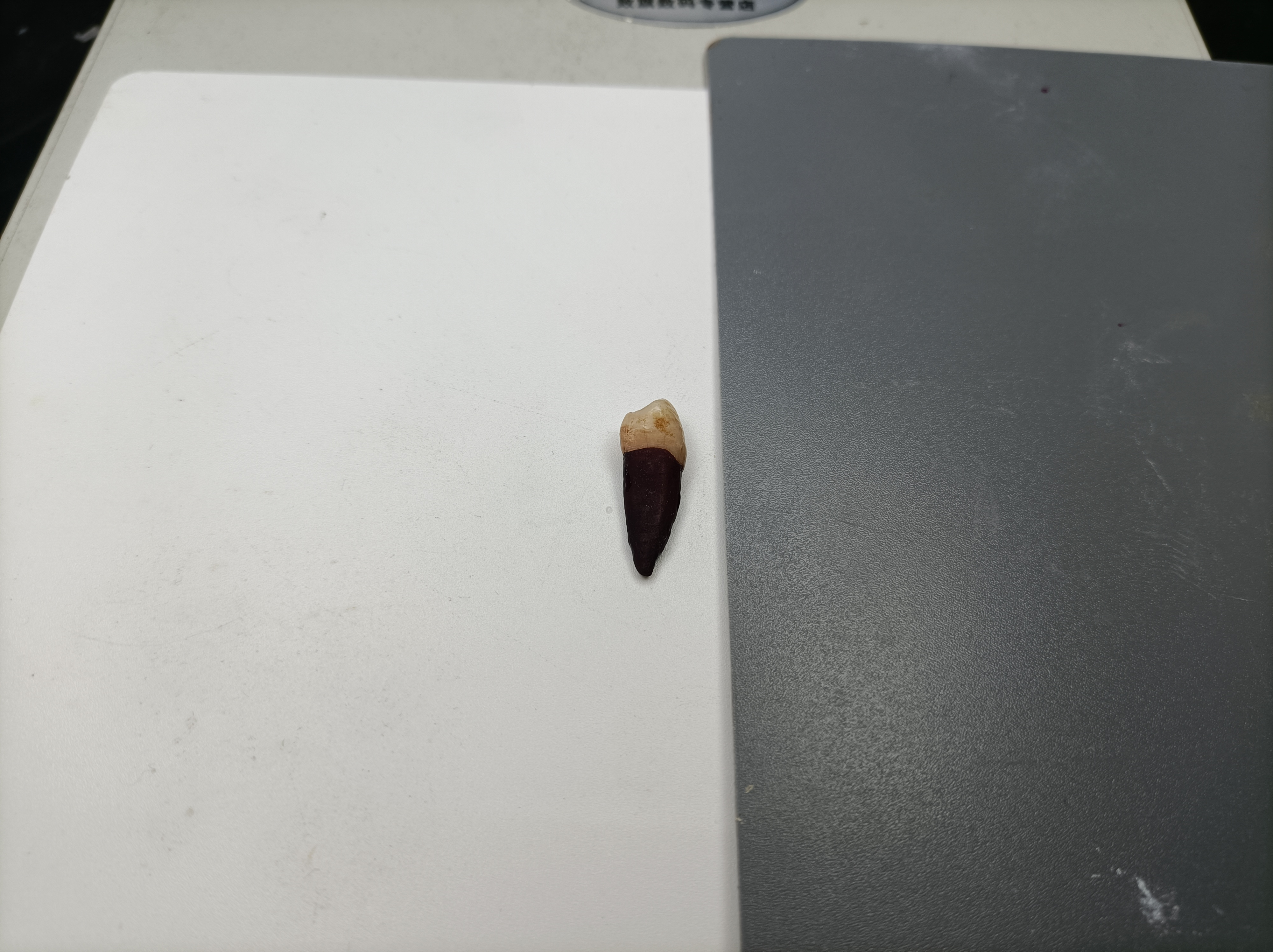

Supplement: Supplementary file 6 — Source data [file 41467_2022_32132_MOESM6_ESM.zip › Source data/supporting/S14/35 without PMNPT/50.jpg]

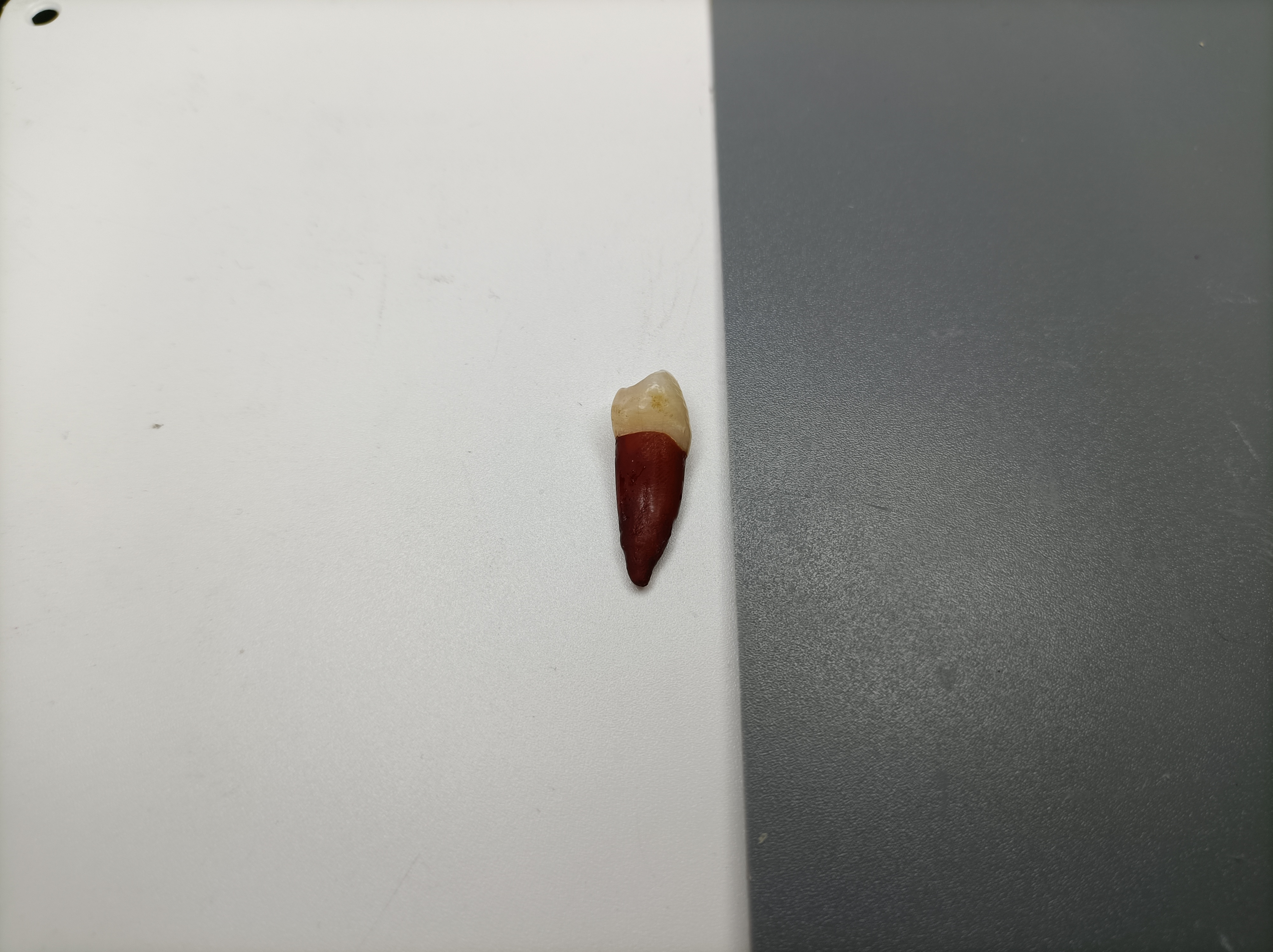

Supplement: Supplementary file 6 — Source data [file 41467_2022_32132_MOESM6_ESM.zip › Source data/supporting/S14/35 without PMNPT/500.jpg]

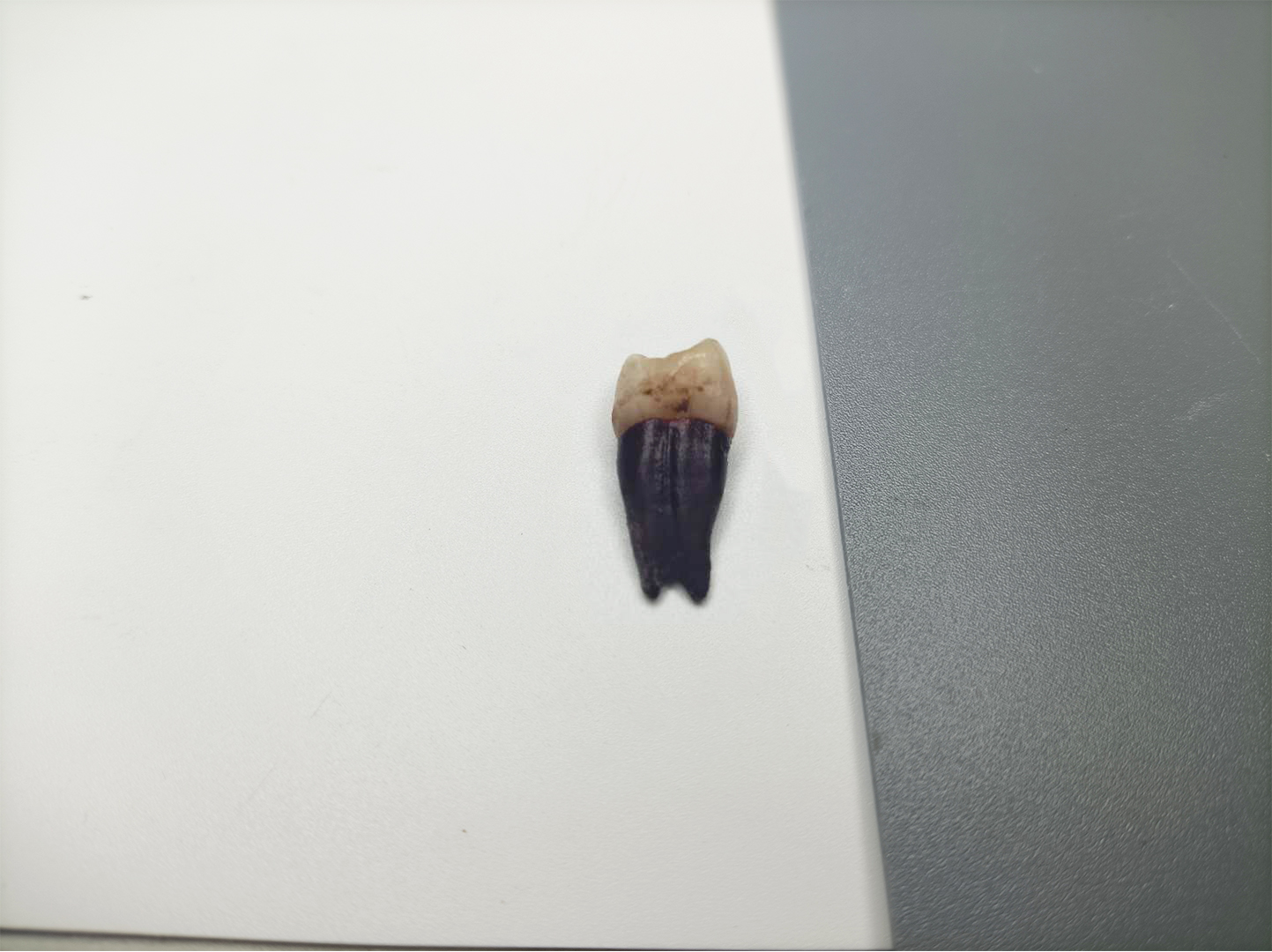

Supplement: Supplementary file 6 — Source data [file 41467_2022_32132_MOESM6_ESM.zip › Source data/supporting/S14/35/0.jpg]

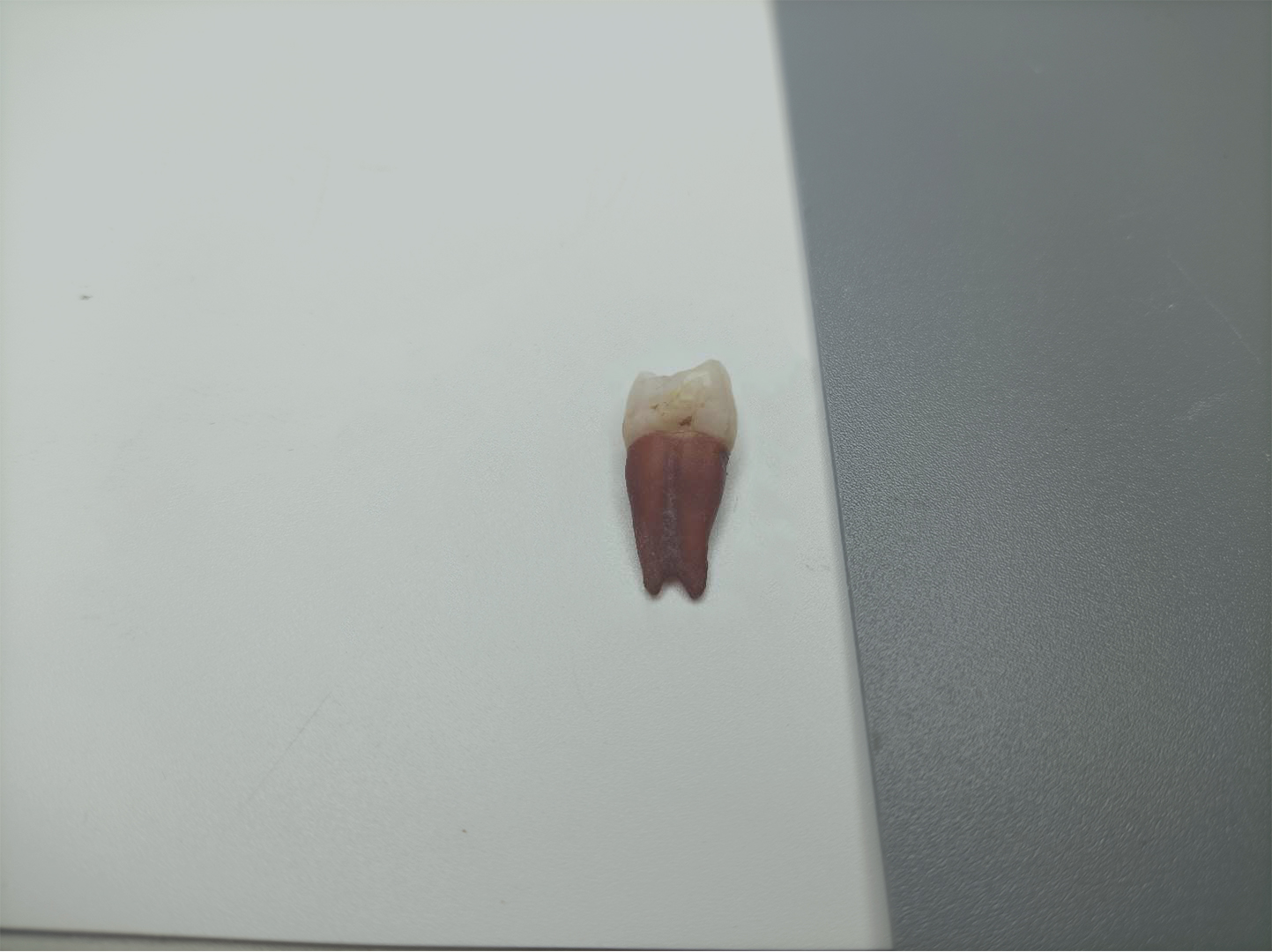

Supplement: Supplementary file 6 — Source data [file 41467_2022_32132_MOESM6_ESM.zip › Source data/supporting/S14/35/100.jpg]

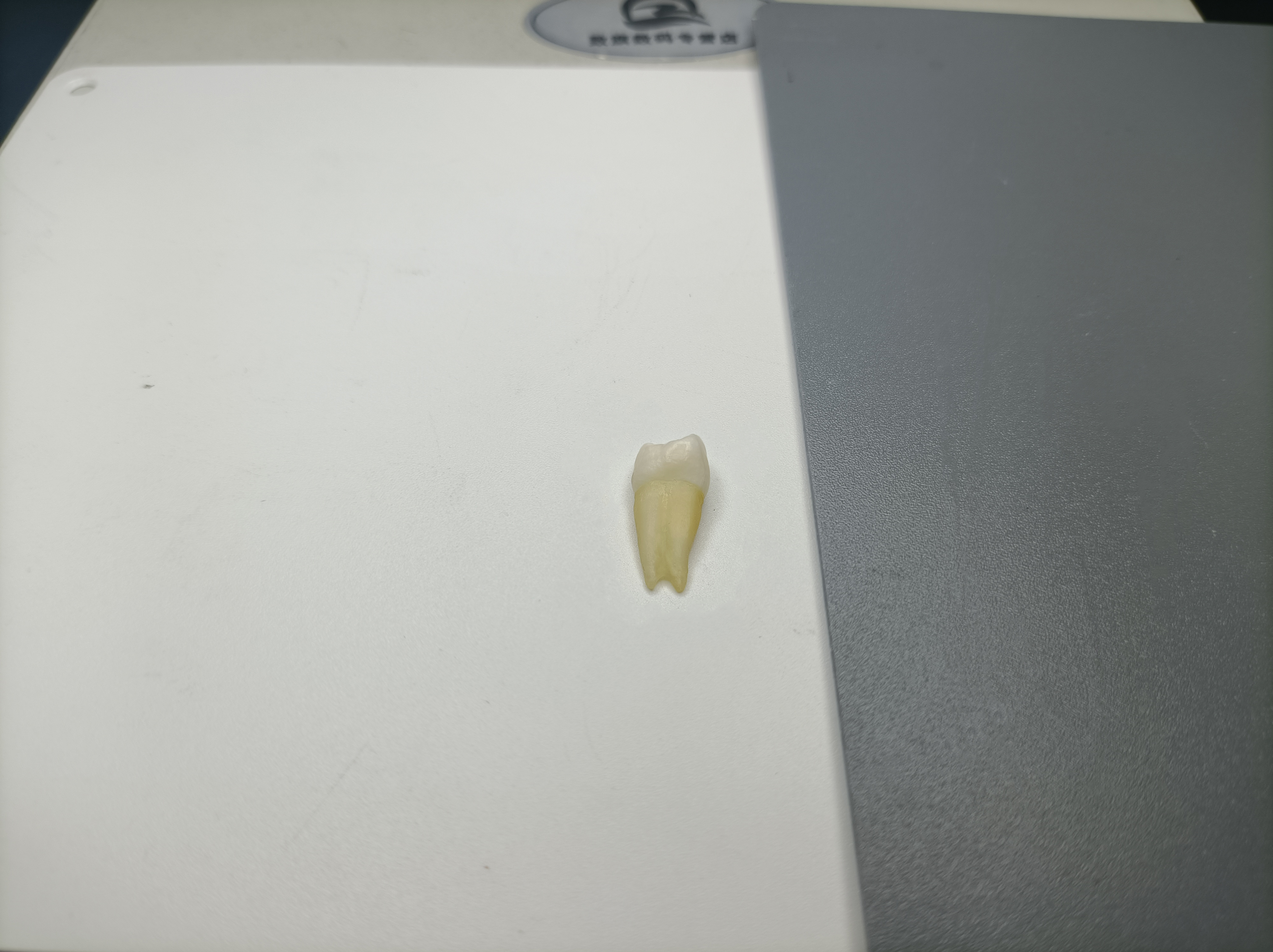

Supplement: Supplementary file 6 — Source data [file 41467_2022_32132_MOESM6_ESM.zip › Source data/supporting/S14/35/1000.jpg]

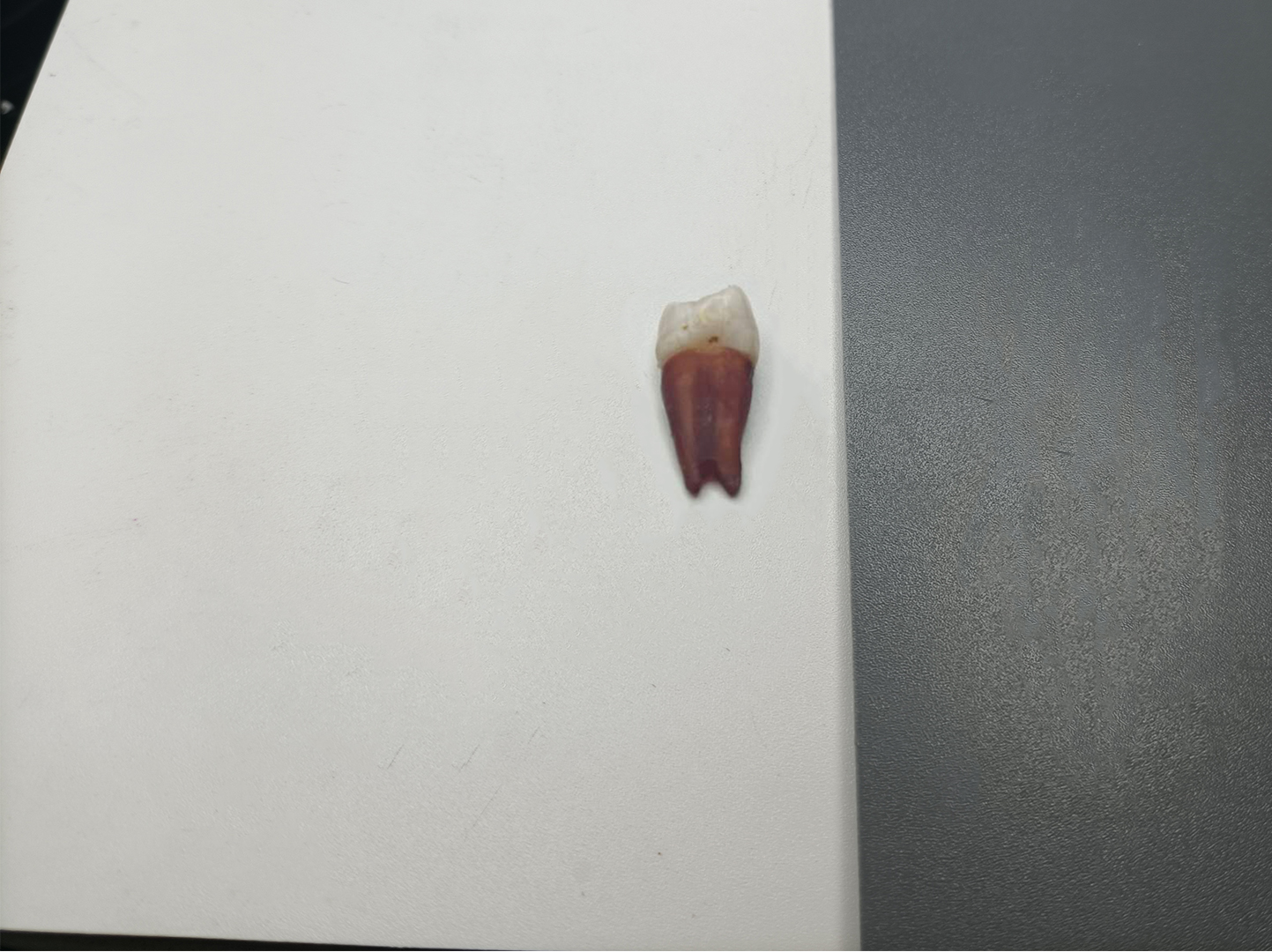

Supplement: Supplementary file 6 — Source data [file 41467_2022_32132_MOESM6_ESM.zip › Source data/supporting/S14/35/150.jpg]

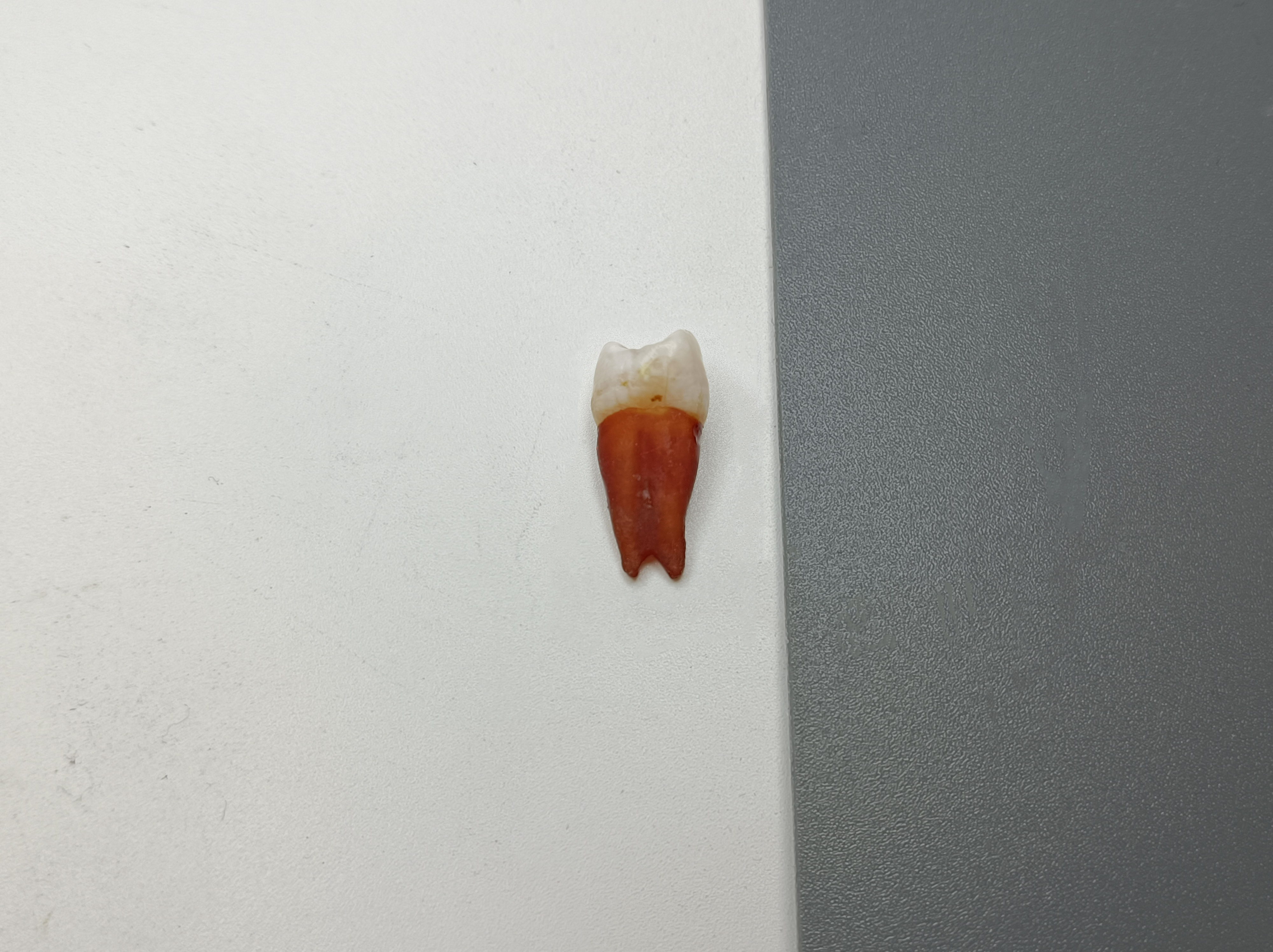

Supplement: Supplementary file 6 — Source data [file 41467_2022_32132_MOESM6_ESM.zip › Source data/supporting/S14/35/200.jpg]

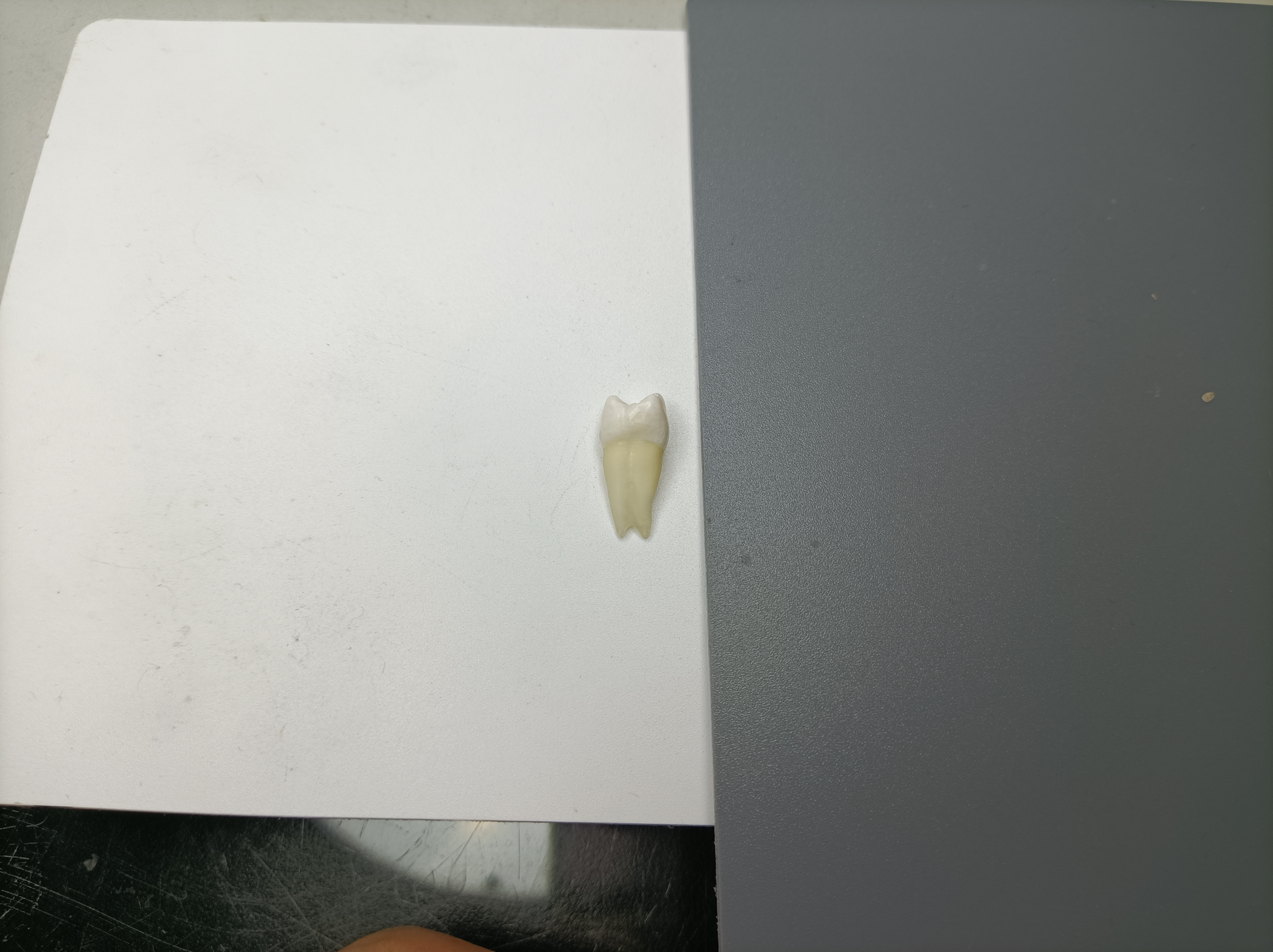

Supplement: Supplementary file 6 — Source data [file 41467_2022_32132_MOESM6_ESM.zip › Source data/supporting/S14/35/2000.jpg]

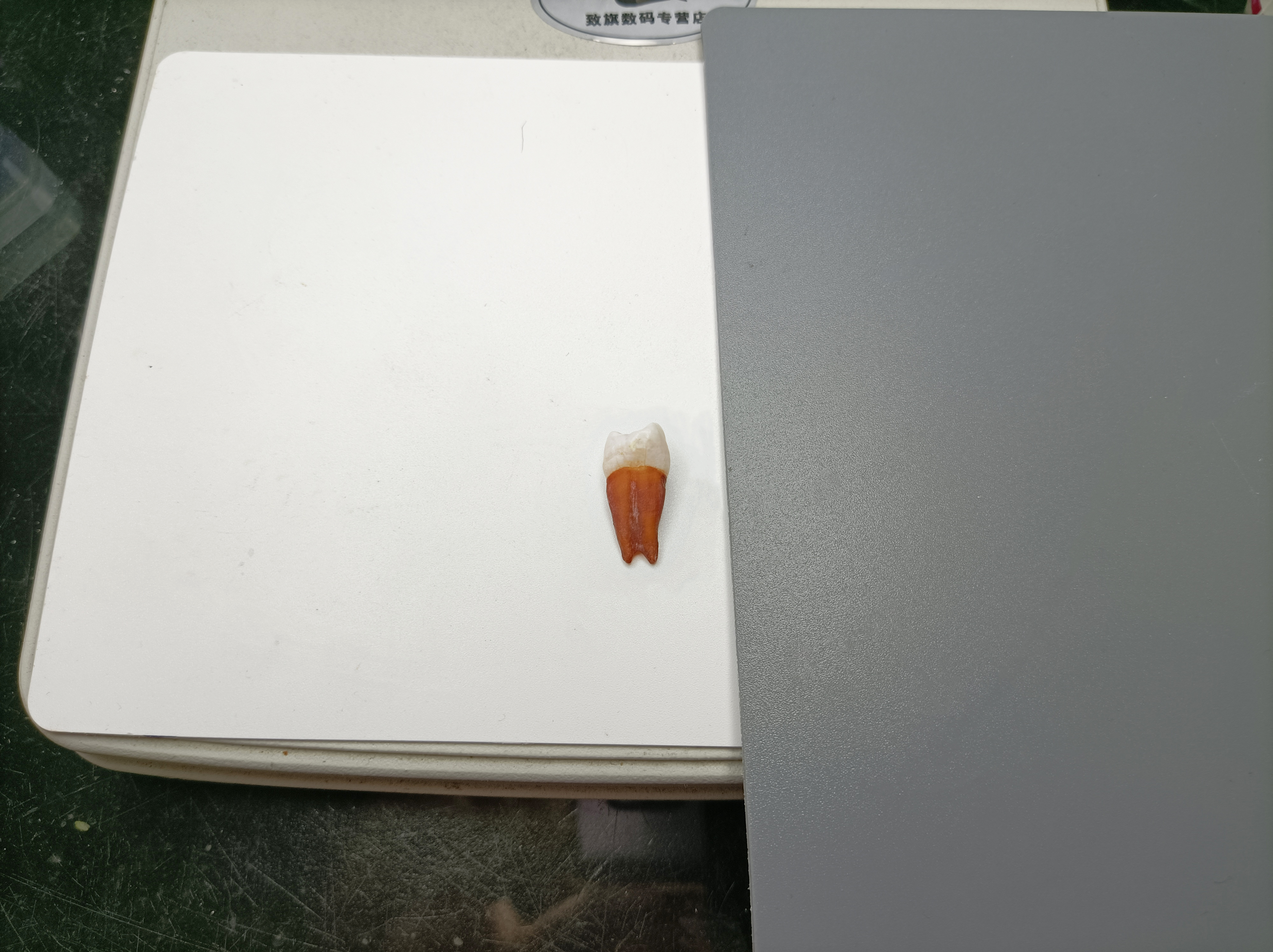

Supplement: Supplementary file 6 — Source data [file 41467_2022_32132_MOESM6_ESM.zip › Source data/supporting/S14/35/300.jpg]

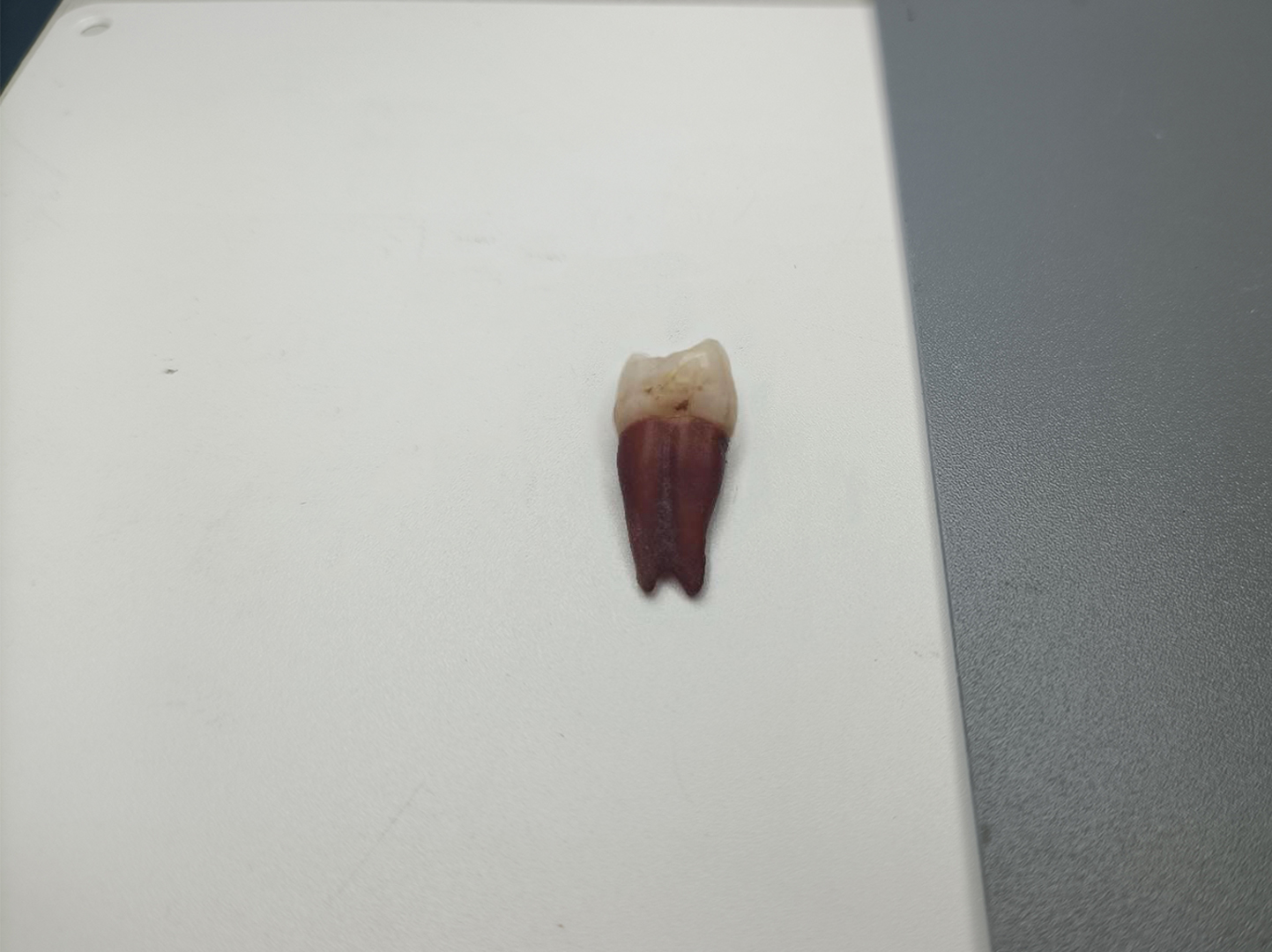

Supplement: Supplementary file 6 — Source data [file 41467_2022_32132_MOESM6_ESM.zip › Source data/supporting/S14/35/50.jpg]

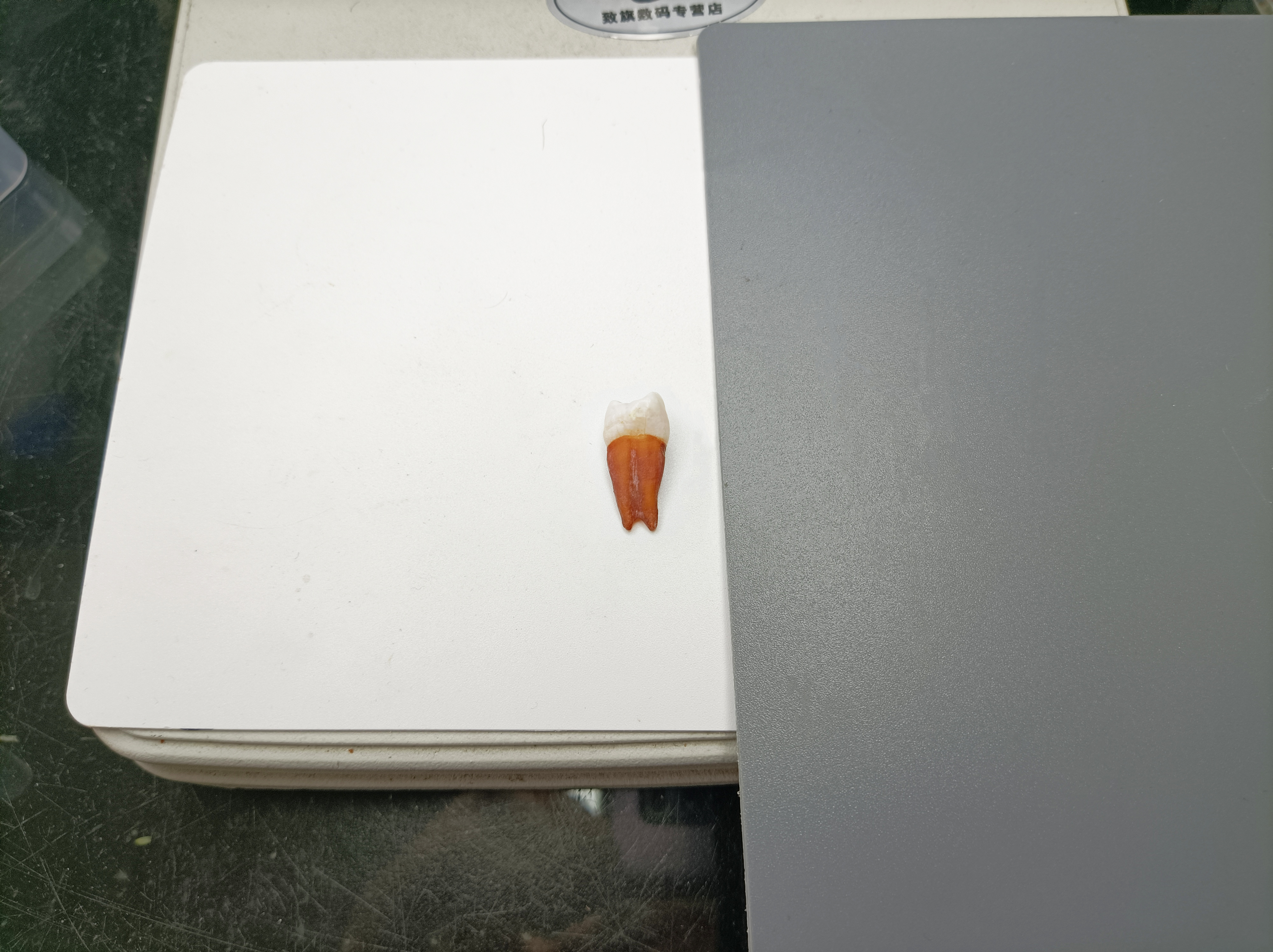

Supplement: Supplementary file 6 — Source data [file 41467_2022_32132_MOESM6_ESM.zip › Source data/supporting/S14/35/500.jpg]

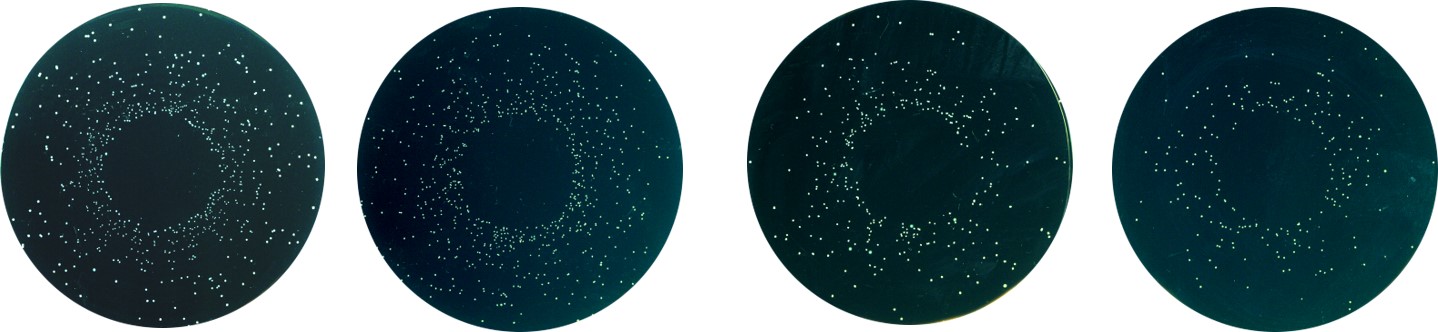

Supplement: Supplementary file 6 — Source data [file 41467_2022_32132_MOESM6_ESM.zip › Source data/supporting/S17/Figure S17a-b.jpg]
