# Supplementary figures and images for: Pyro-catalysis for tooth whitening via oral temperature fluctuation (part 1 of 2)
Source: Nat Commun. 2022 Jul 29;13:4419. doi: 10.1038/s41467-022-32132-3 (PMC9338087; doi:10.1038/s41467-022-32132-3)

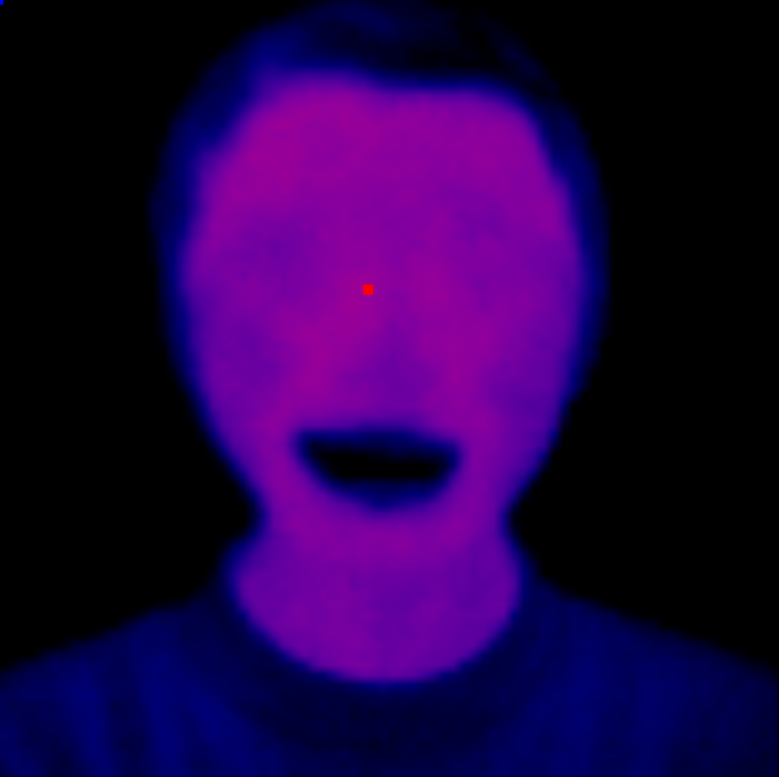

Supplement: Supplementary file 6 — Source data [file 41467_2022_32132_MOESM6_ESM.zip › Source data/main text/Figure 1/cooling.png]

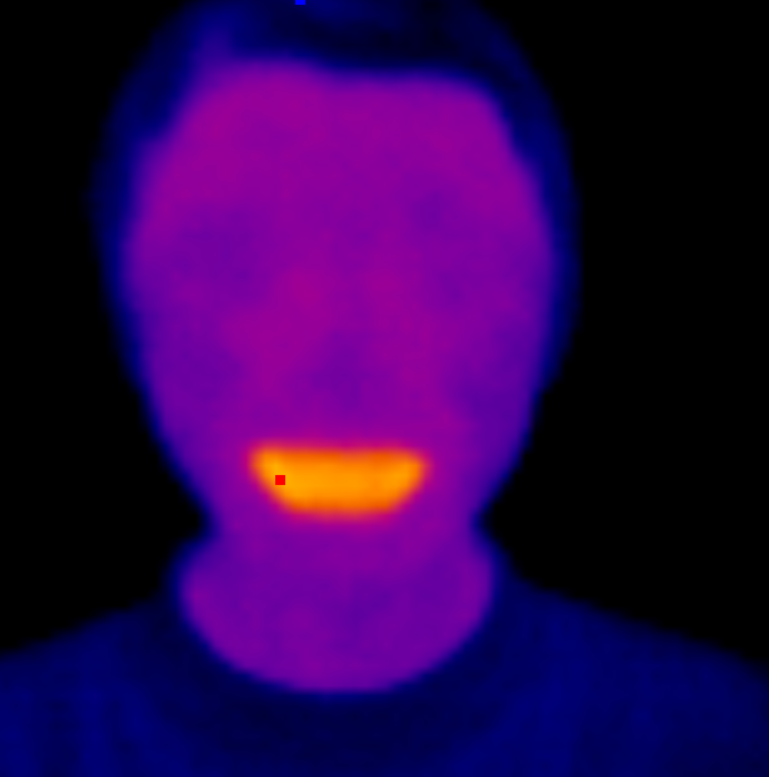

Supplement: Supplementary file 6 — Source data [file 41467_2022_32132_MOESM6_ESM.zip › Source data/main text/Figure 1/heating.png]

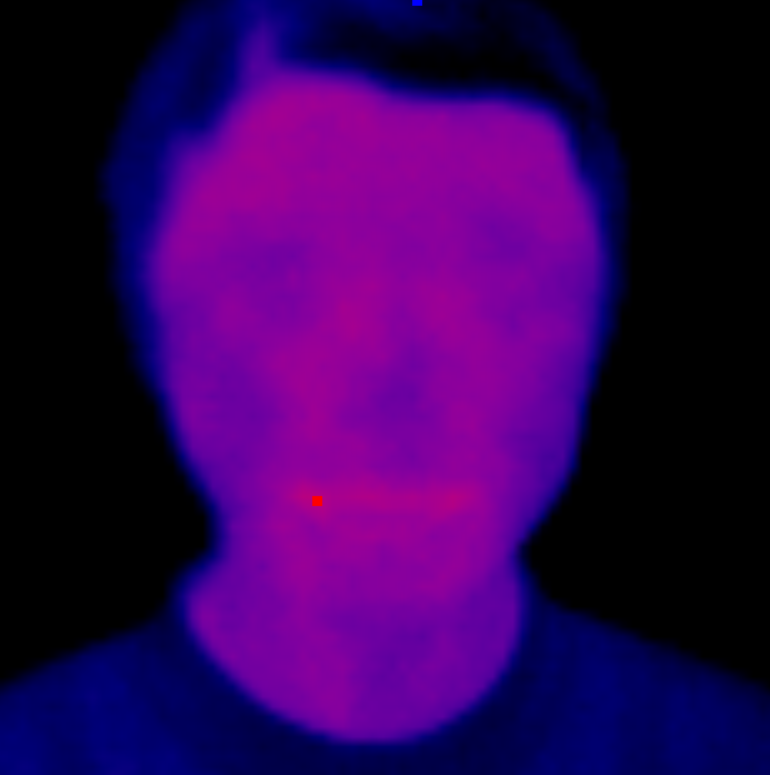

Supplement: Supplementary file 6 — Source data [file 41467_2022_32132_MOESM6_ESM.zip › Source data/main text/Figure 1/origin.png]

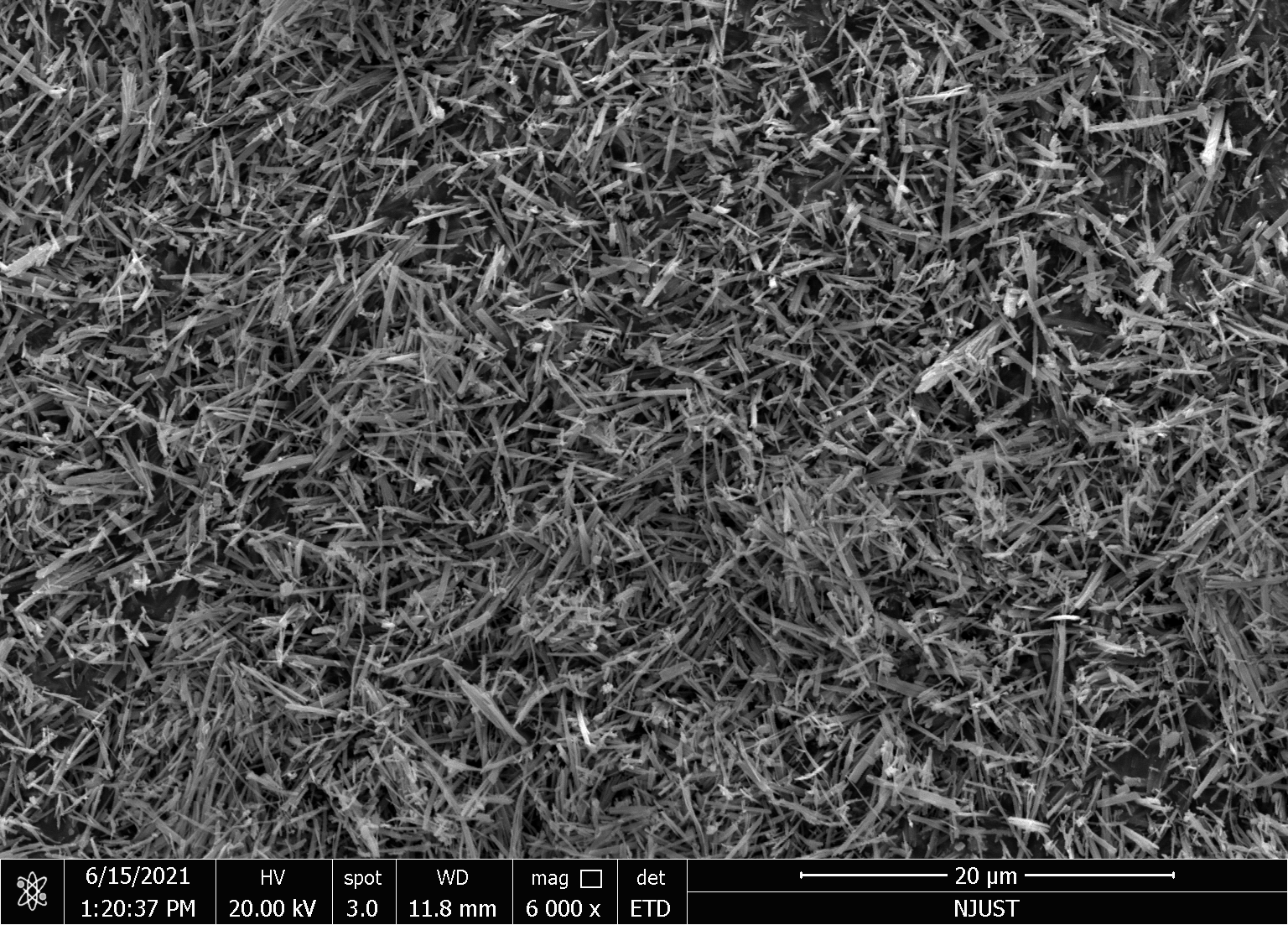

Supplement: Supplementary file 6 — Source data [file 41467_2022_32132_MOESM6_ESM.zip › Source data/main text/Figure 2/Figures/Fig2c.tif]

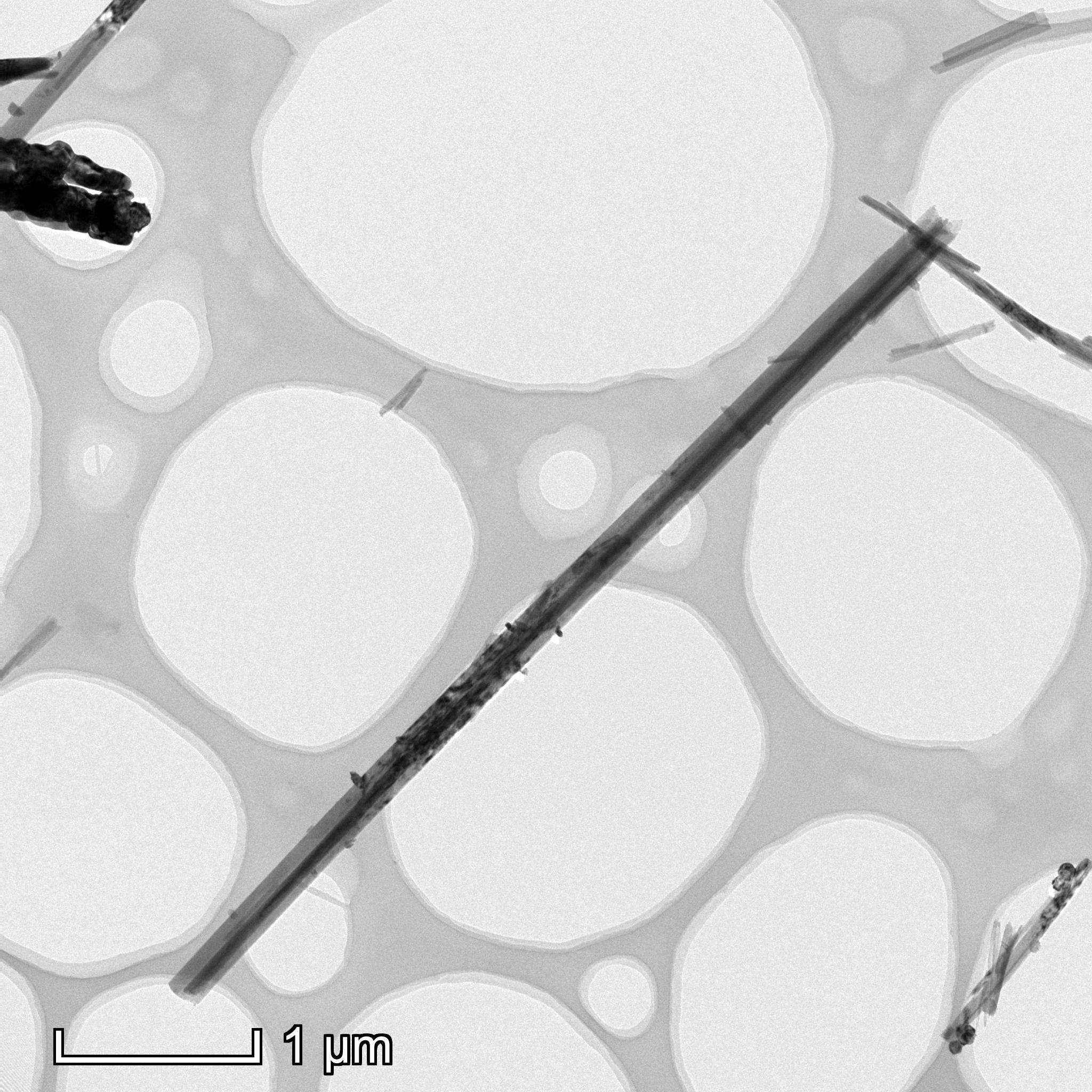

Supplement: Supplementary file 6 — Source data [file 41467_2022_32132_MOESM6_ESM.zip › Source data/main text/Figure 2/Figures/Fig2d.tif]

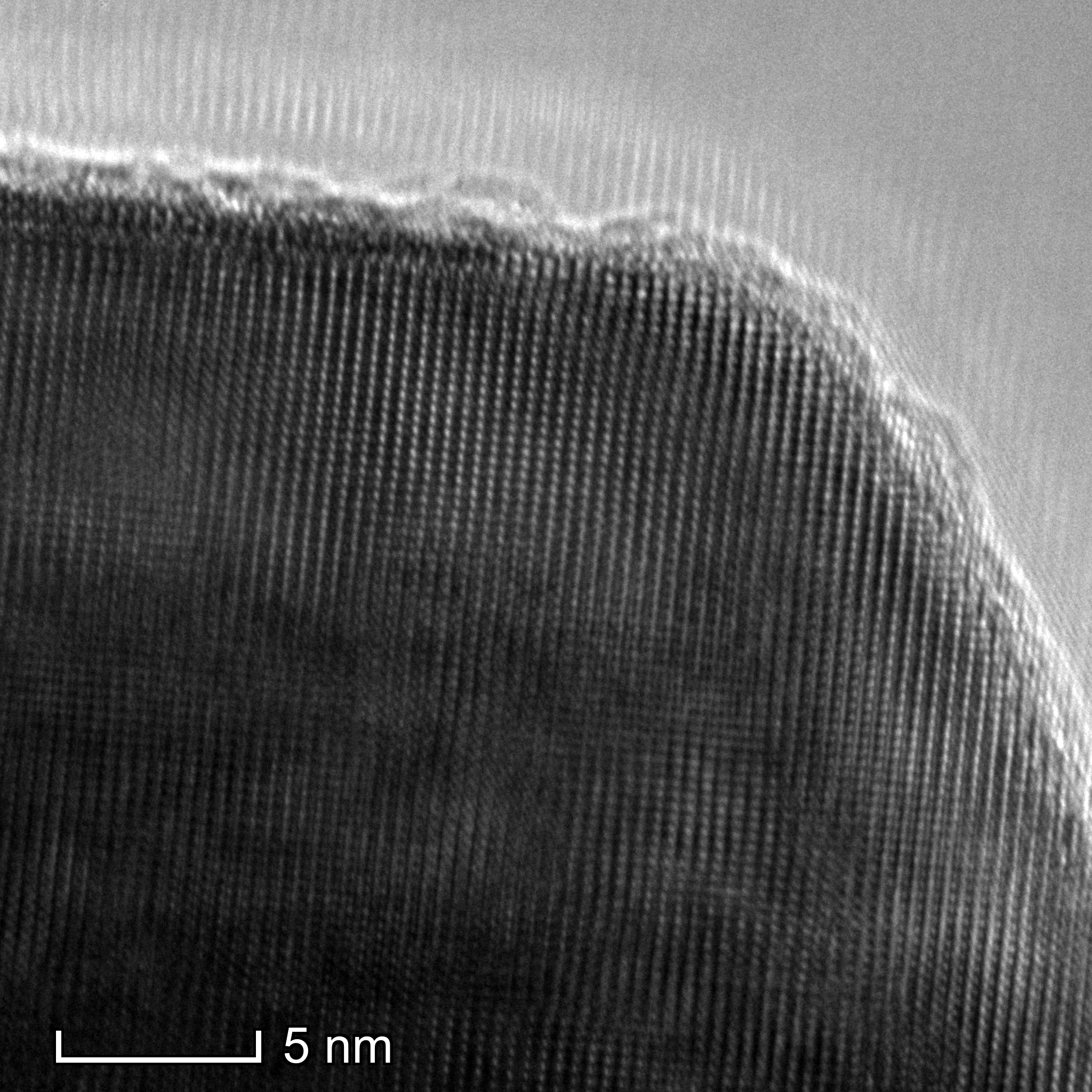

Supplement: Supplementary file 6 — Source data [file 41467_2022_32132_MOESM6_ESM.zip › Source data/main text/Figure 2/Figures/Fig2e.tif]

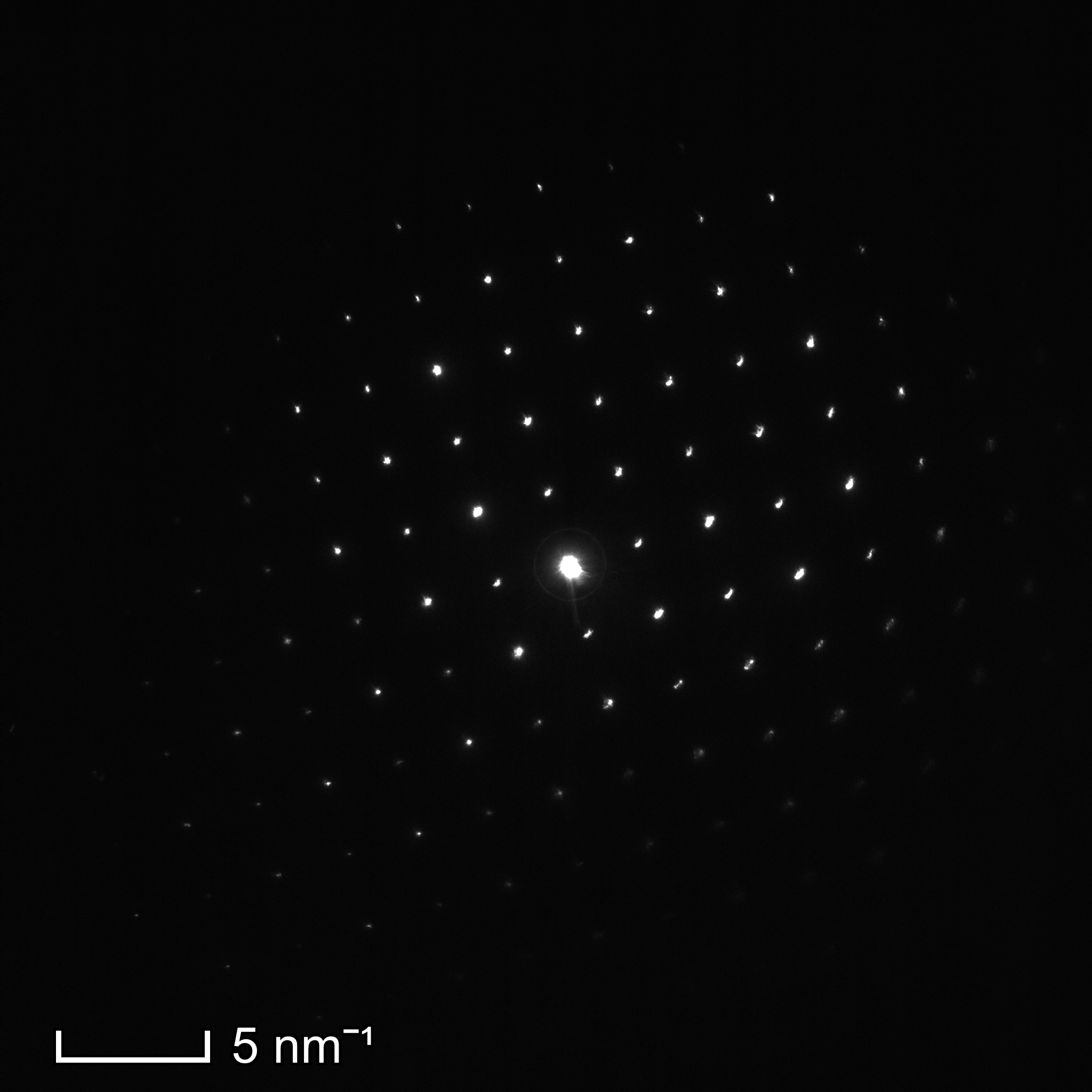

Supplement: Supplementary file 6 — Source data [file 41467_2022_32132_MOESM6_ESM.zip › Source data/main text/Figure 2/Figures/Fig2f.tif]

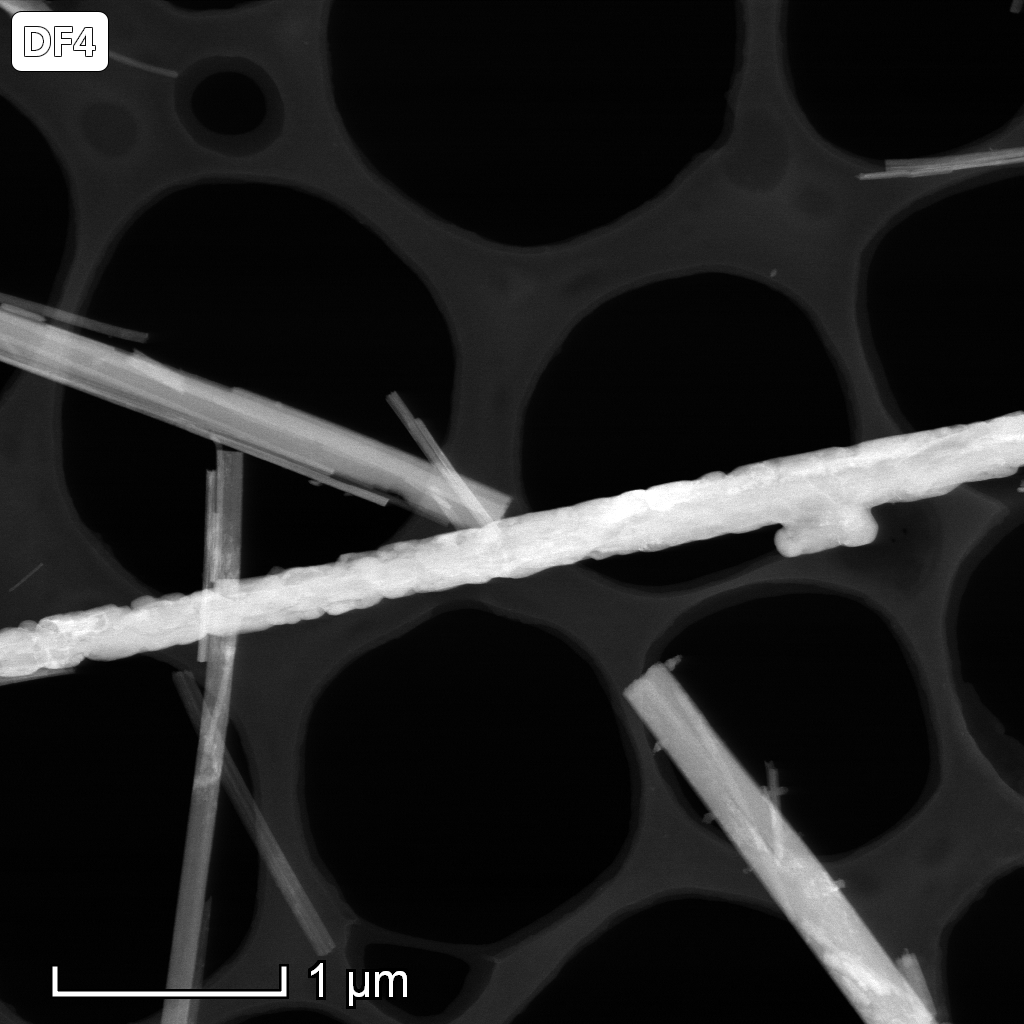

Supplement: Supplementary file 6 — Source data [file 41467_2022_32132_MOESM6_ESM.zip › Source data/main text/Figure 2/Figures/Fig2g.tif]

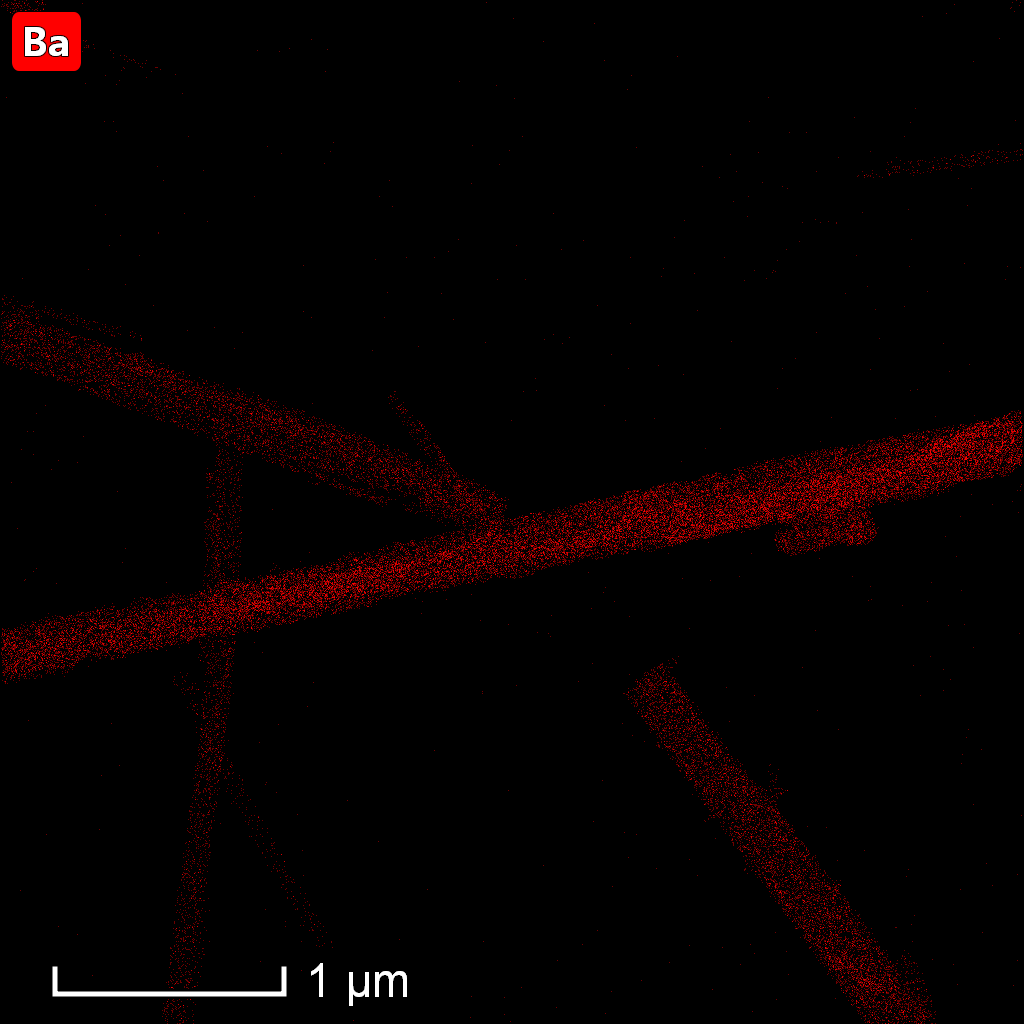

Supplement: Supplementary file 6 — Source data [file 41467_2022_32132_MOESM6_ESM.zip › Source data/main text/Figure 2/Figures/Fig2h.tif]

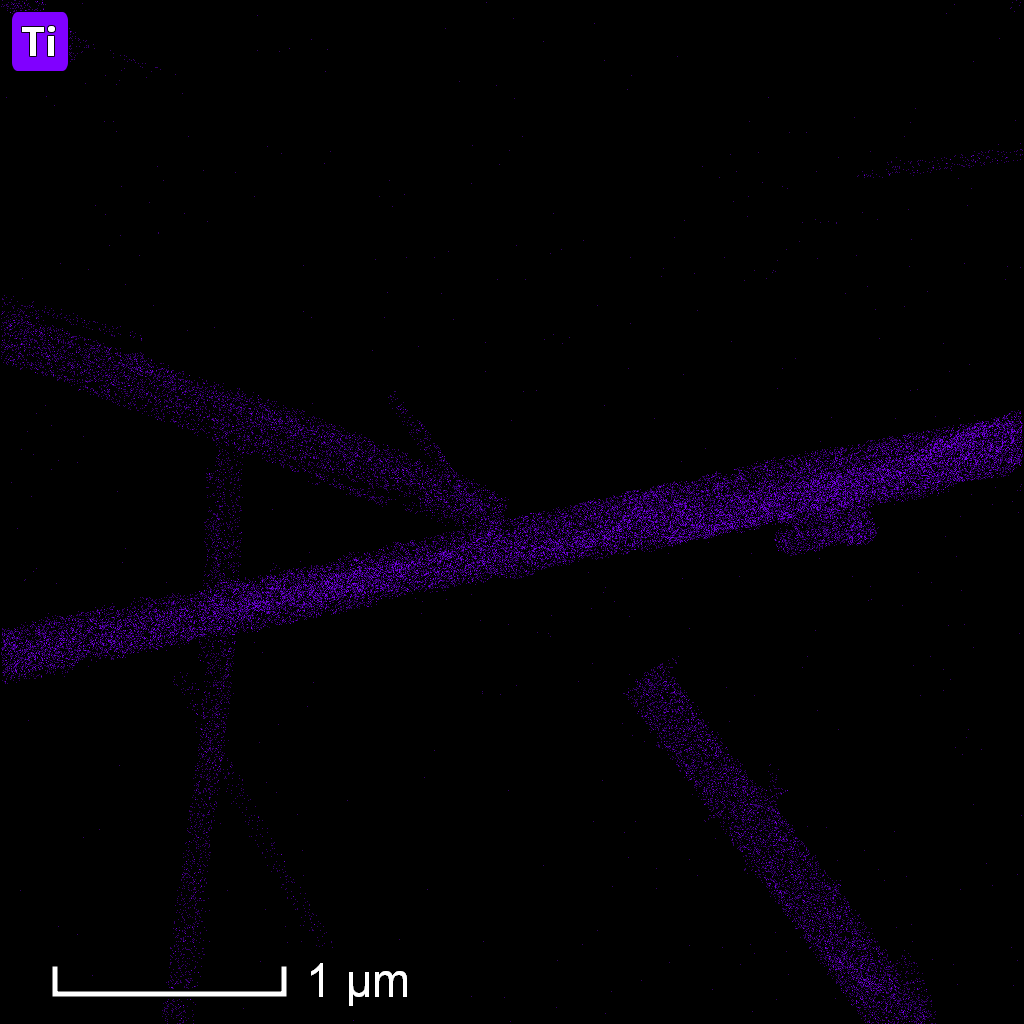

Supplement: Supplementary file 6 — Source data [file 41467_2022_32132_MOESM6_ESM.zip › Source data/main text/Figure 2/Figures/Fig2i.tif]

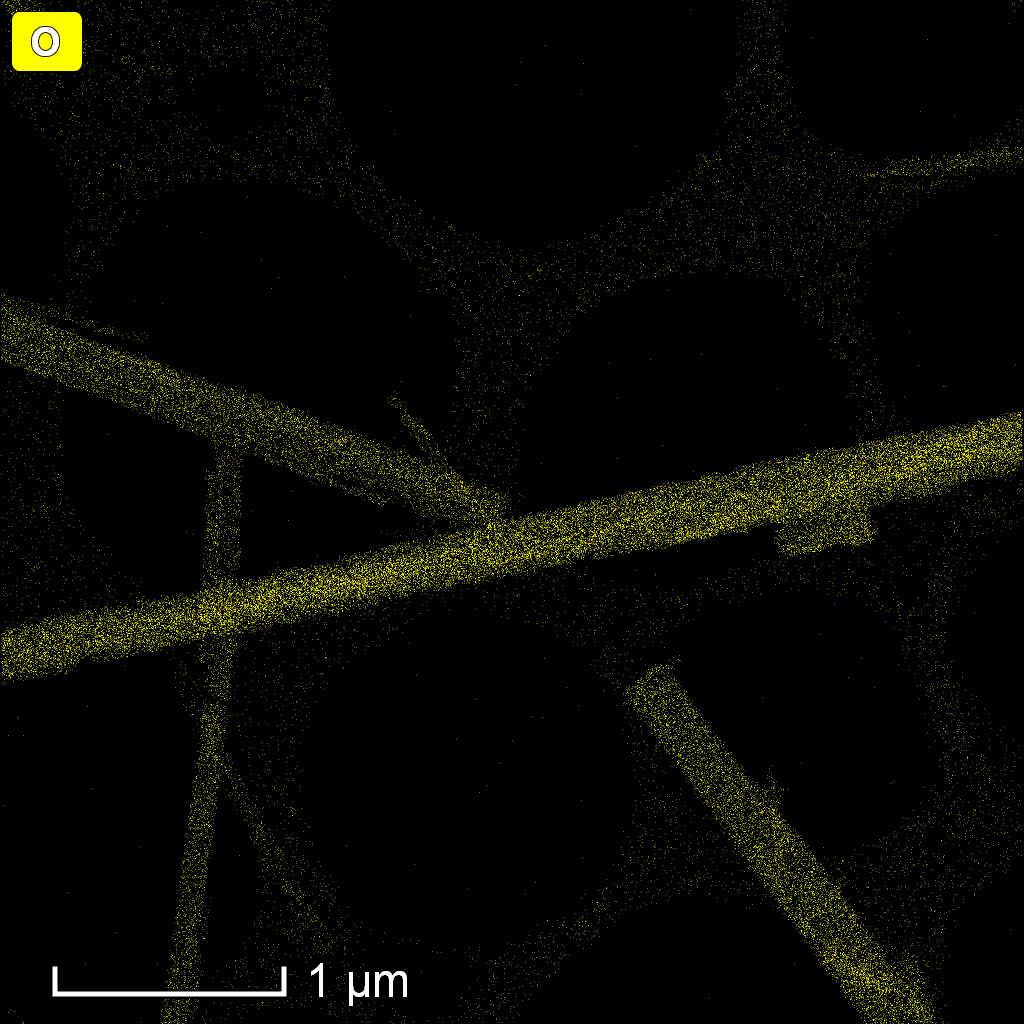

Supplement: Supplementary file 6 — Source data [file 41467_2022_32132_MOESM6_ESM.zip › Source data/main text/Figure 2/Figures/Fig2j.tif]

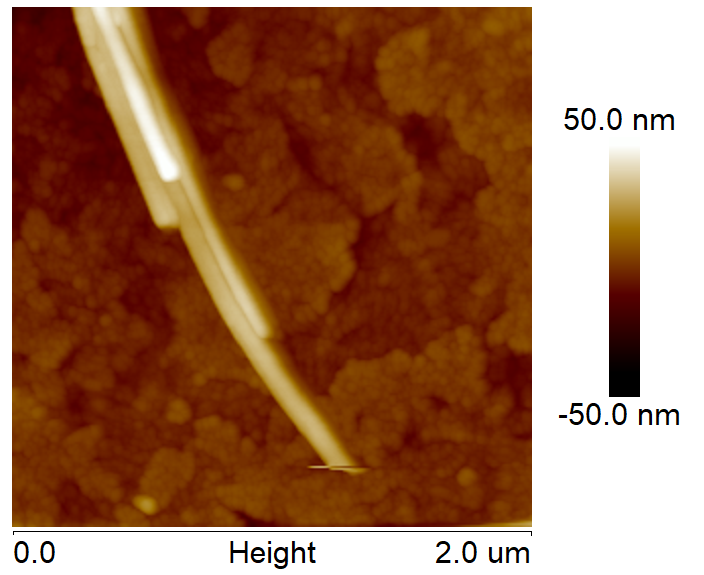

Supplement: Supplementary file 6 — Source data [file 41467_2022_32132_MOESM6_ESM.zip › Source data/main text/Figure 2/Figures/Fig2k.tif]

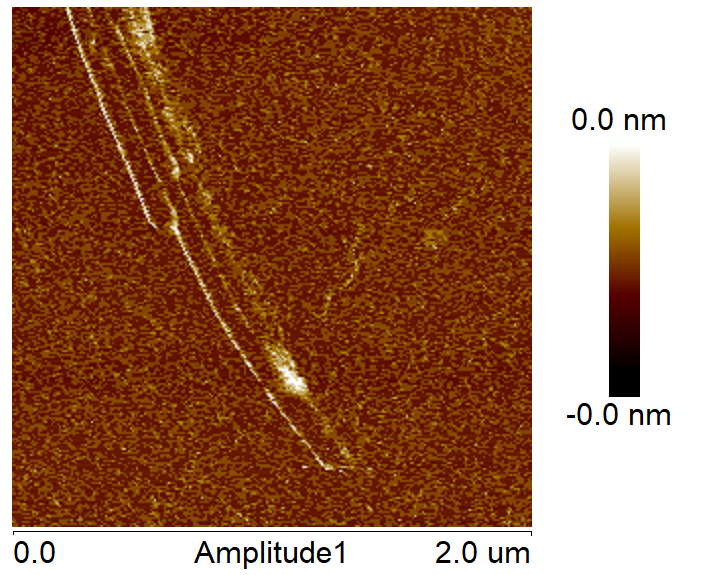

Supplement: Supplementary file 6 — Source data [file 41467_2022_32132_MOESM6_ESM.zip › Source data/main text/Figure 2/Figures/Fig2l.tif]

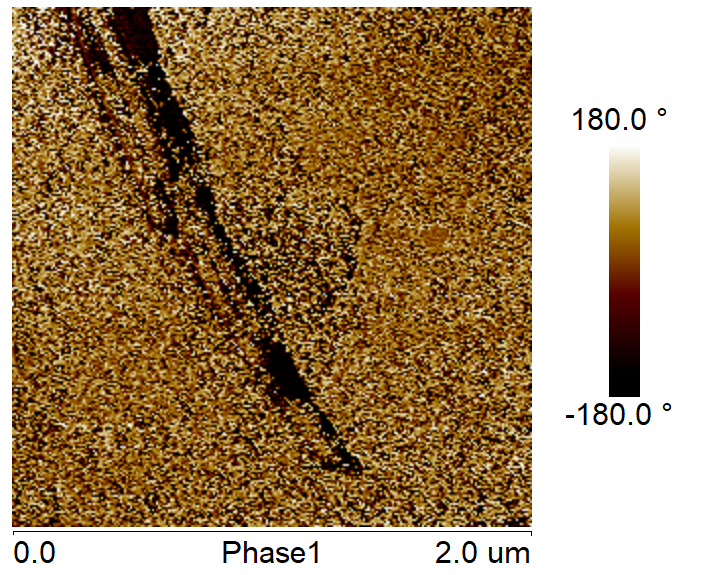

Supplement: Supplementary file 6 — Source data [file 41467_2022_32132_MOESM6_ESM.zip › Source data/main text/Figure 2/Figures/Fig2m.tif]

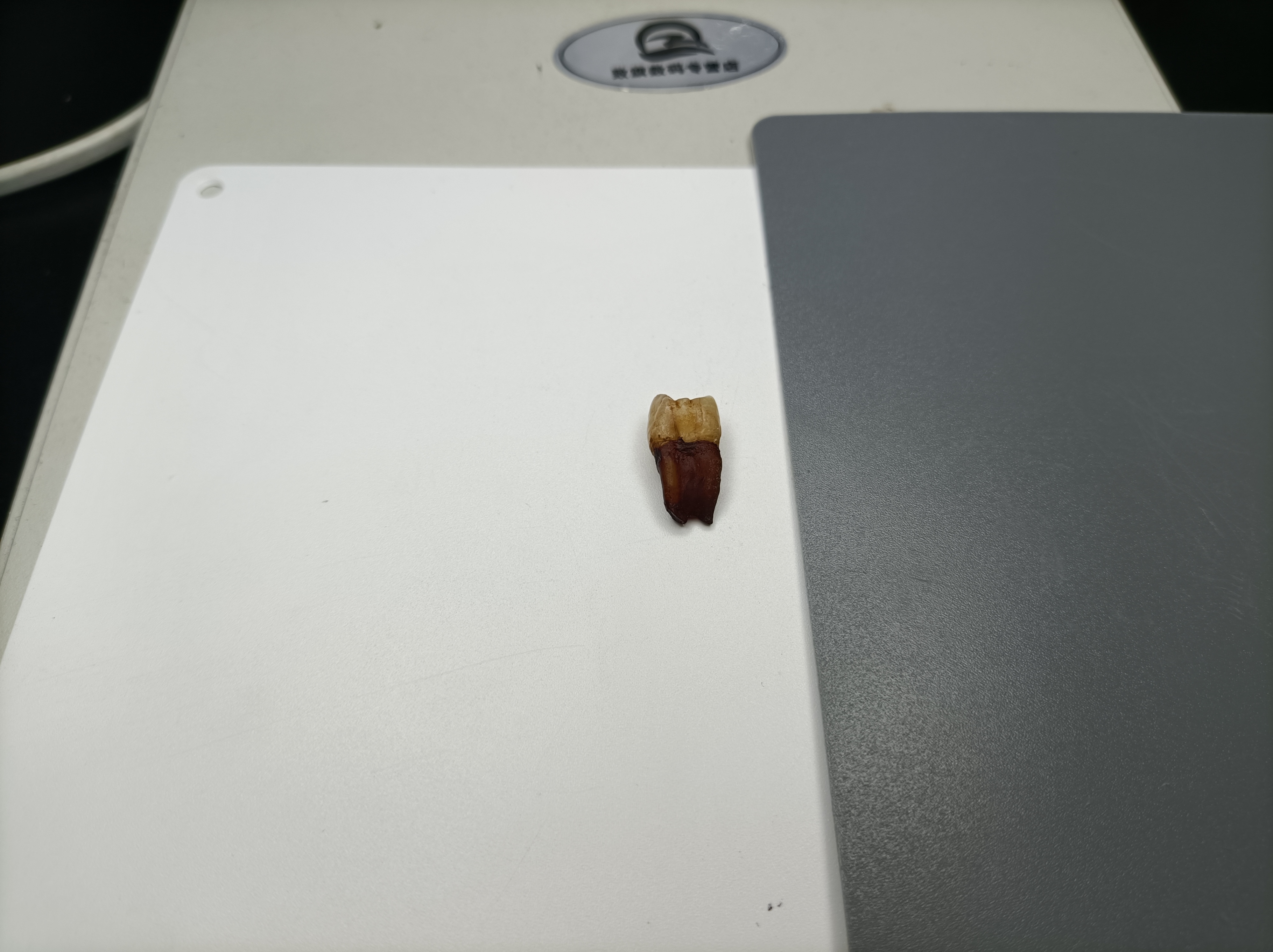

Supplement: Supplementary file 6 — Source data [file 41467_2022_32132_MOESM6_ESM.zip › Source data/main text/Figure 4/Figures/26-36/0.jpg]

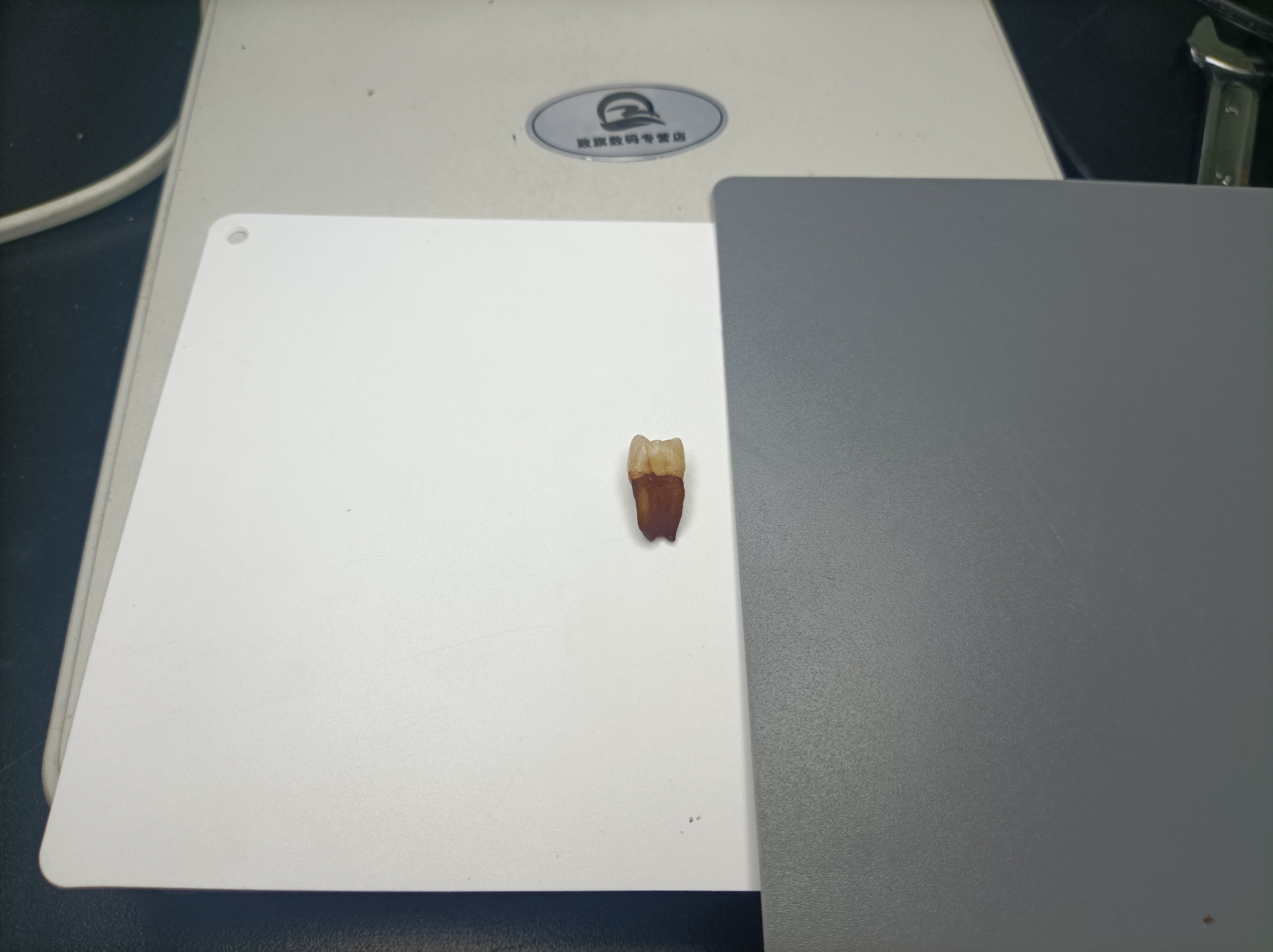

Supplement: Supplementary file 6 — Source data [file 41467_2022_32132_MOESM6_ESM.zip › Source data/main text/Figure 4/Figures/26-36/100.jpg]

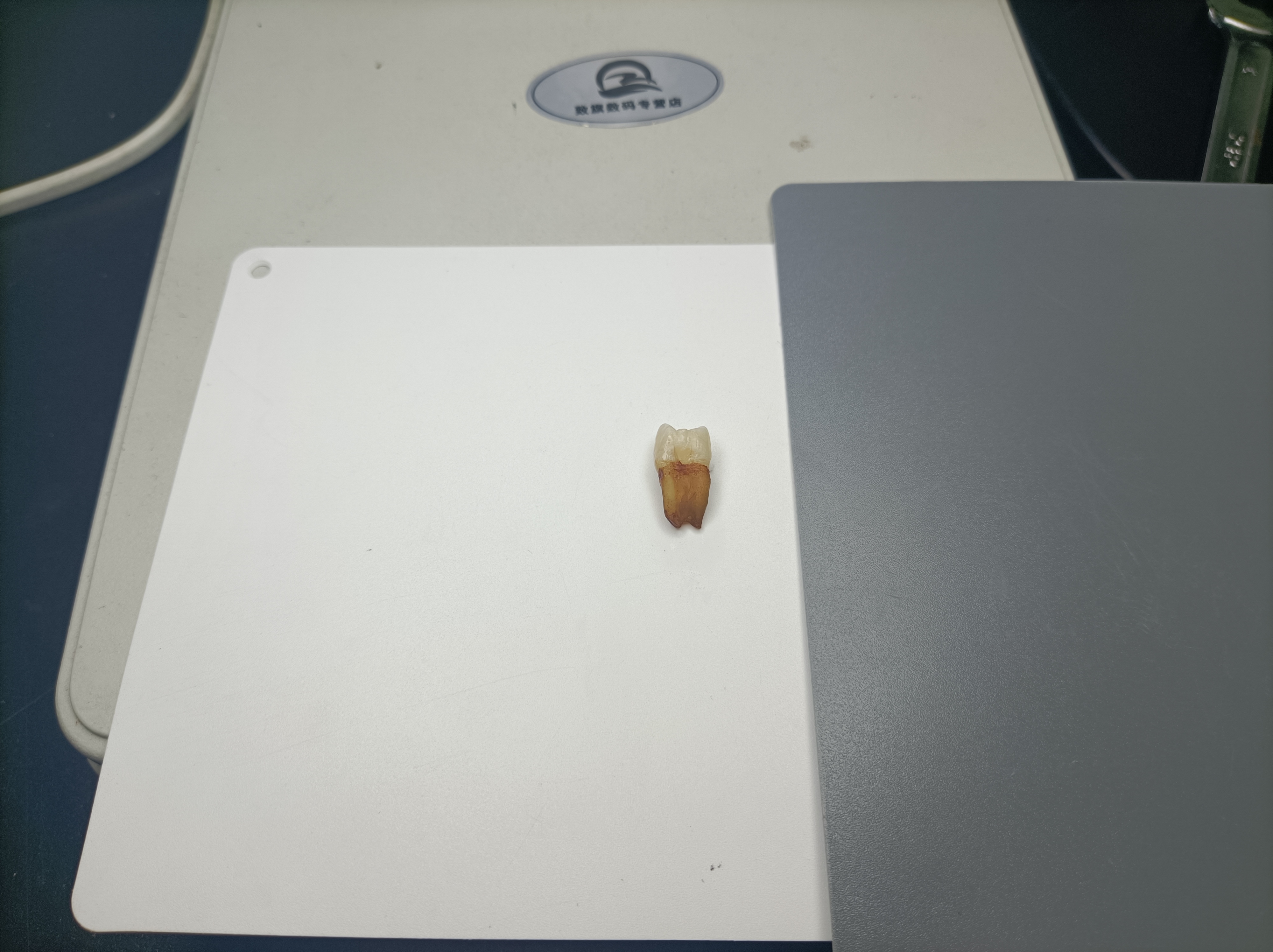

Supplement: Supplementary file 6 — Source data [file 41467_2022_32132_MOESM6_ESM.zip › Source data/main text/Figure 4/Figures/26-36/1000.jpg]

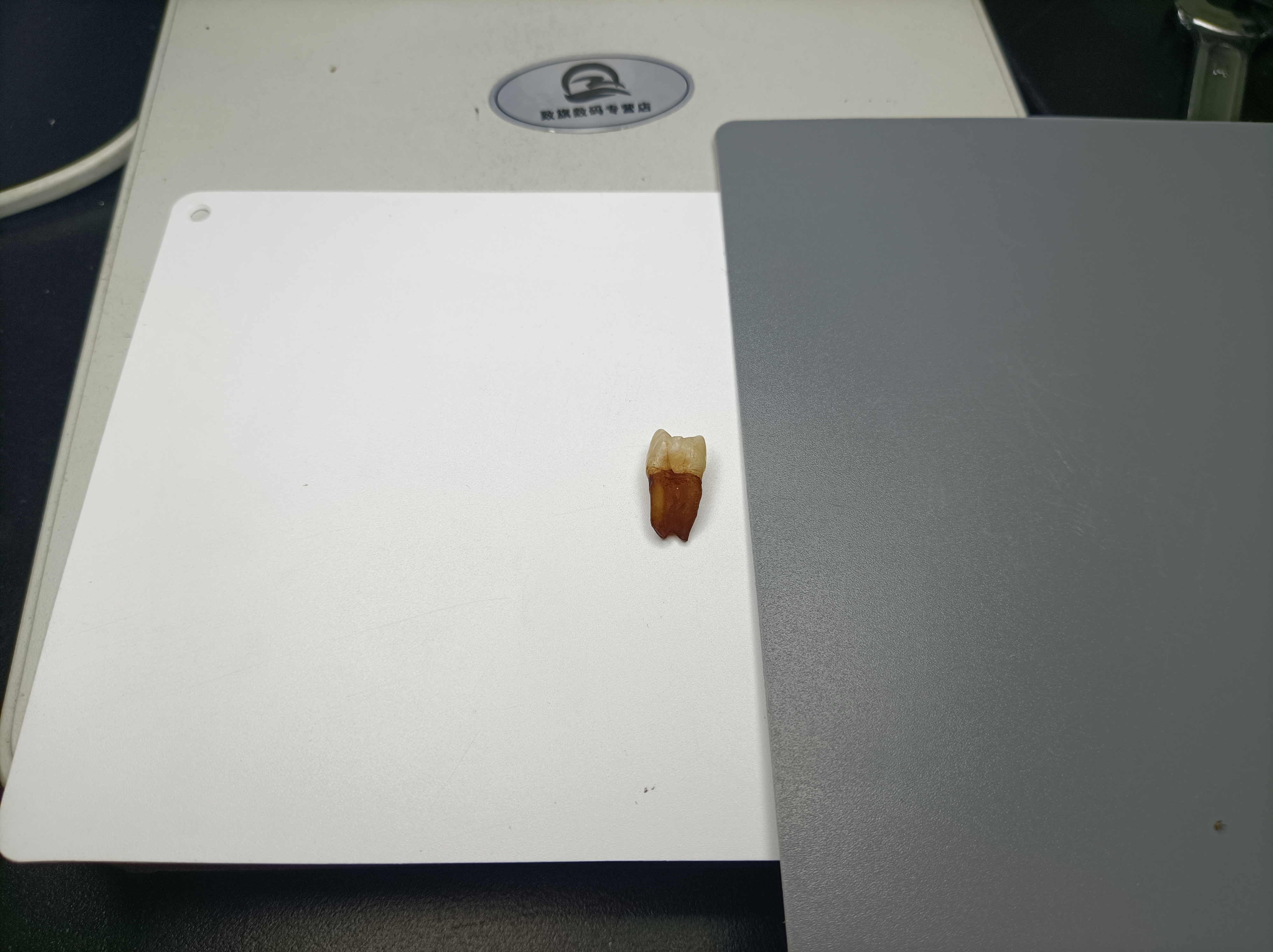

Supplement: Supplementary file 6 — Source data [file 41467_2022_32132_MOESM6_ESM.zip › Source data/main text/Figure 4/Figures/26-36/150.jpg]

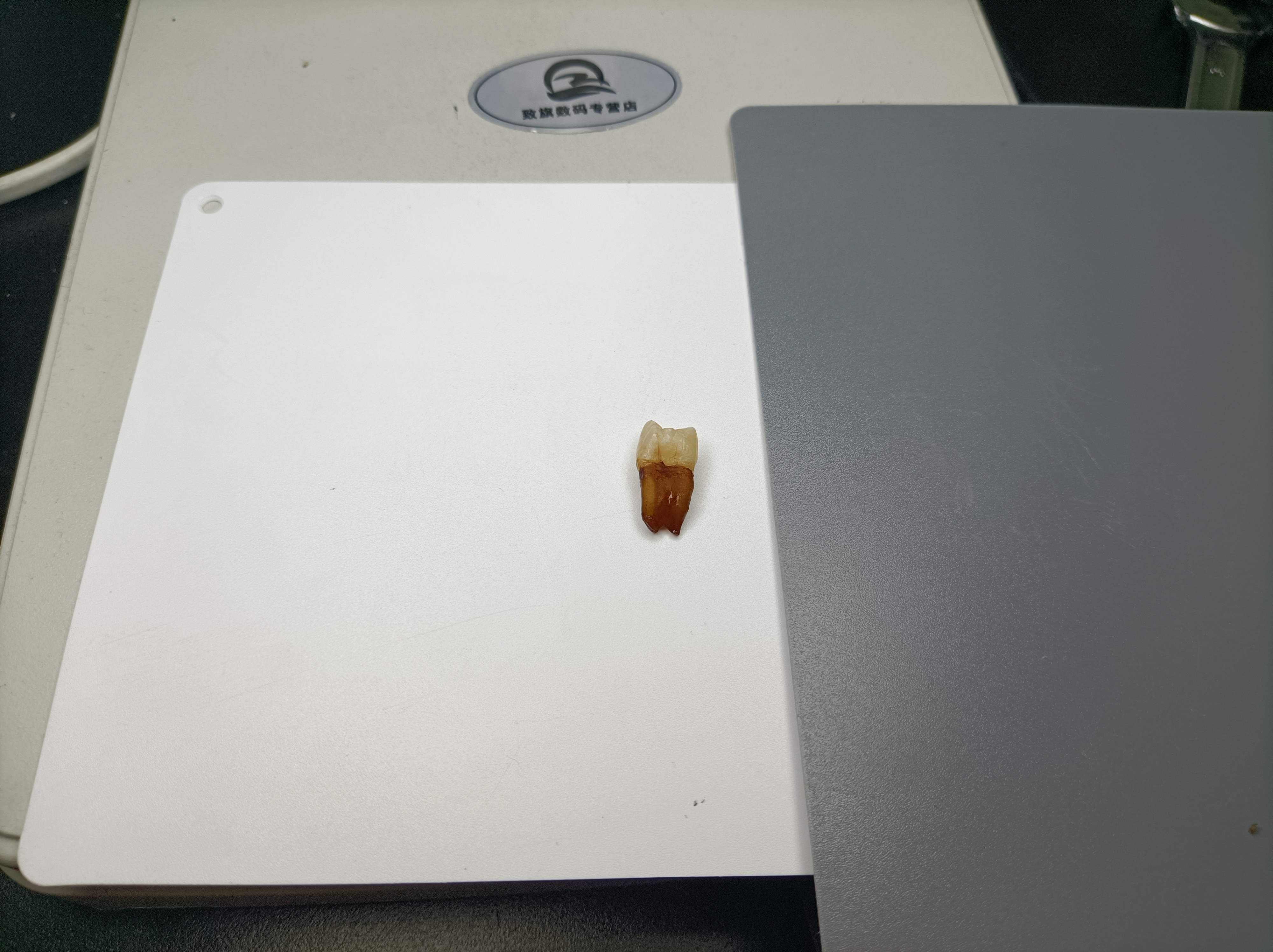

Supplement: Supplementary file 6 — Source data [file 41467_2022_32132_MOESM6_ESM.zip › Source data/main text/Figure 4/Figures/26-36/200.jpg]

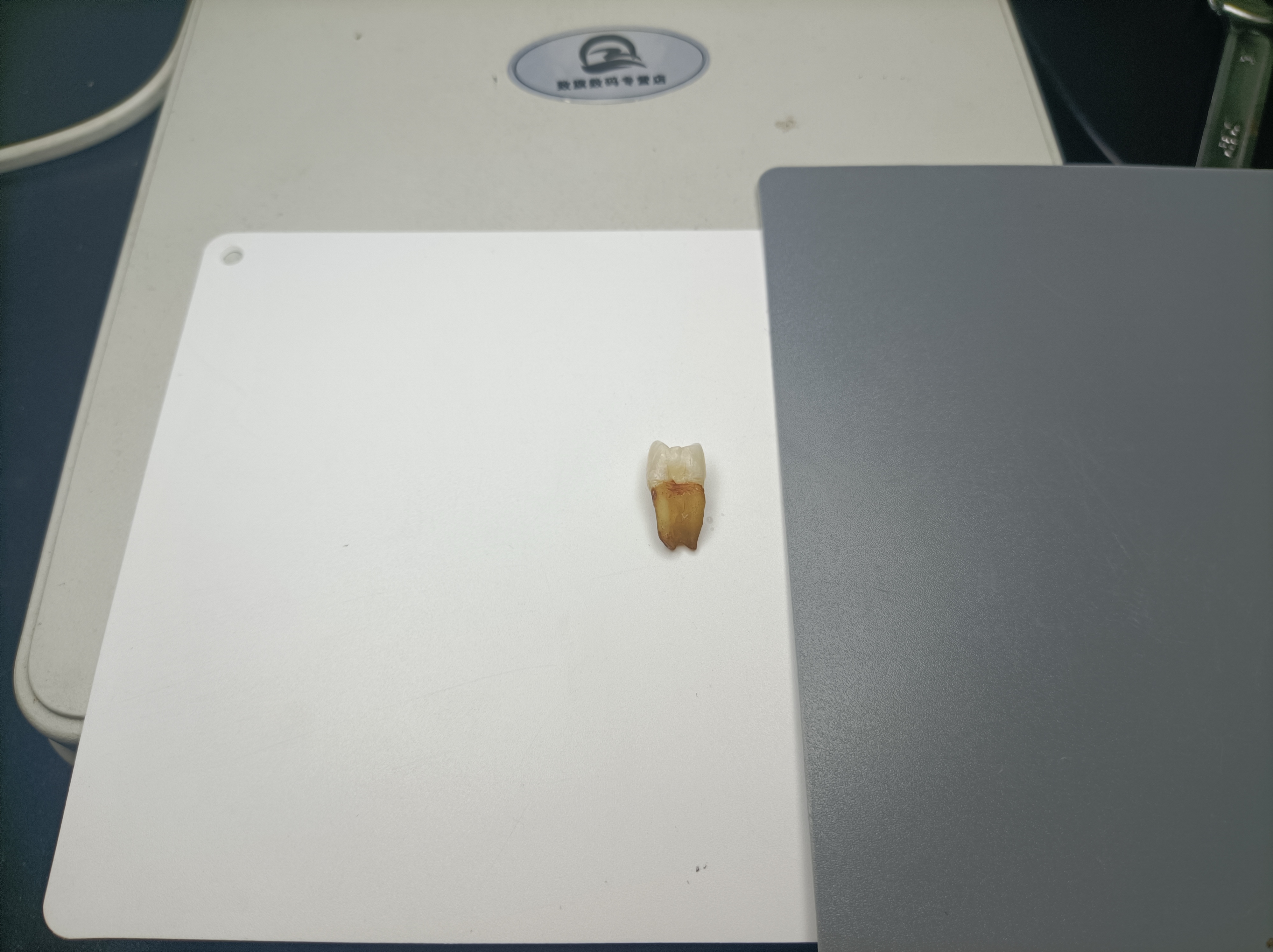

Supplement: Supplementary file 6 — Source data [file 41467_2022_32132_MOESM6_ESM.zip › Source data/main text/Figure 4/Figures/26-36/2000.jpg]

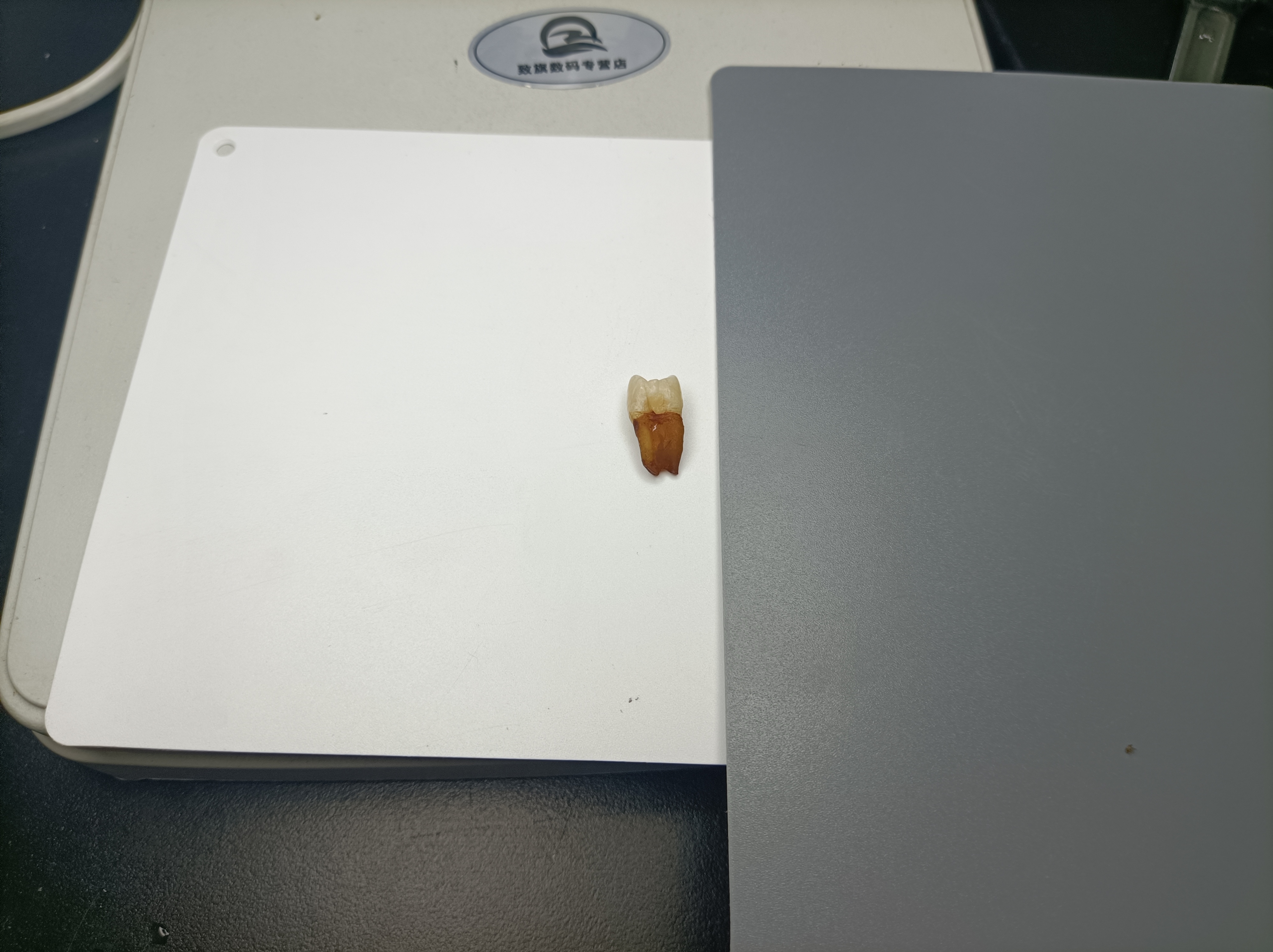

Supplement: Supplementary file 6 — Source data [file 41467_2022_32132_MOESM6_ESM.zip › Source data/main text/Figure 4/Figures/26-36/300.jpg]

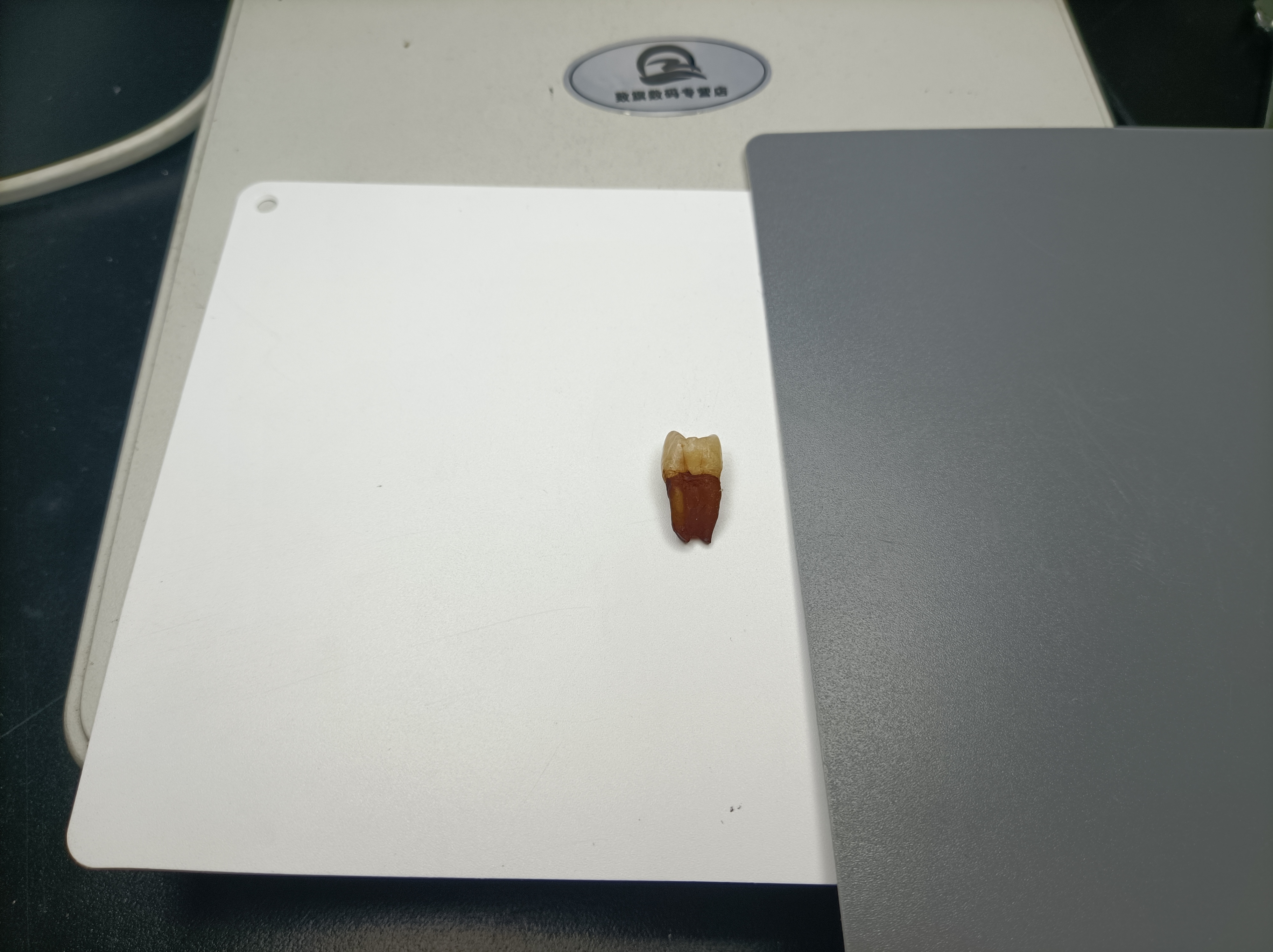

Supplement: Supplementary file 6 — Source data [file 41467_2022_32132_MOESM6_ESM.zip › Source data/main text/Figure 4/Figures/26-36/50.jpg]

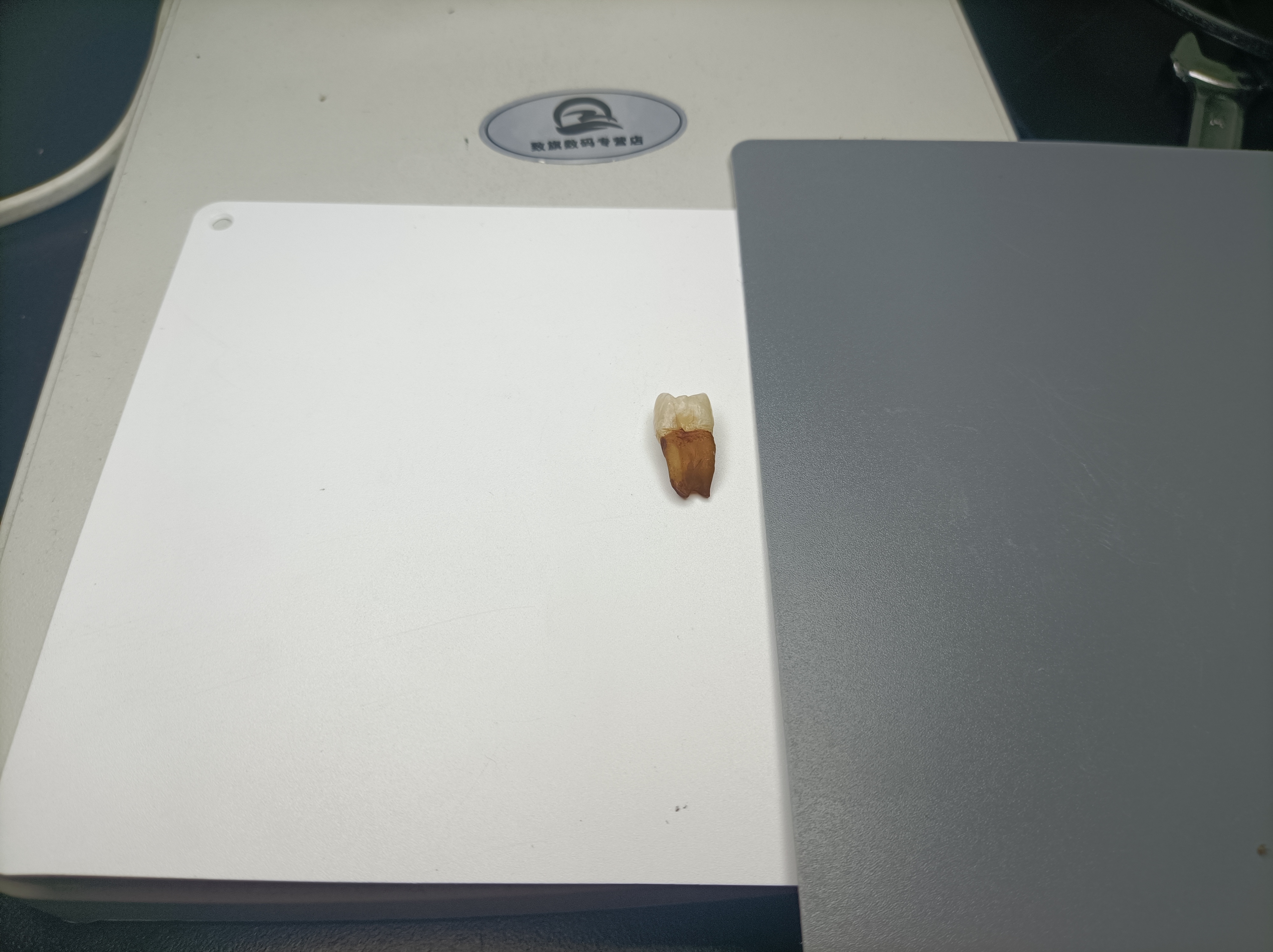

Supplement: Supplementary file 6 — Source data [file 41467_2022_32132_MOESM6_ESM.zip › Source data/main text/Figure 4/Figures/26-36/500.jpg]

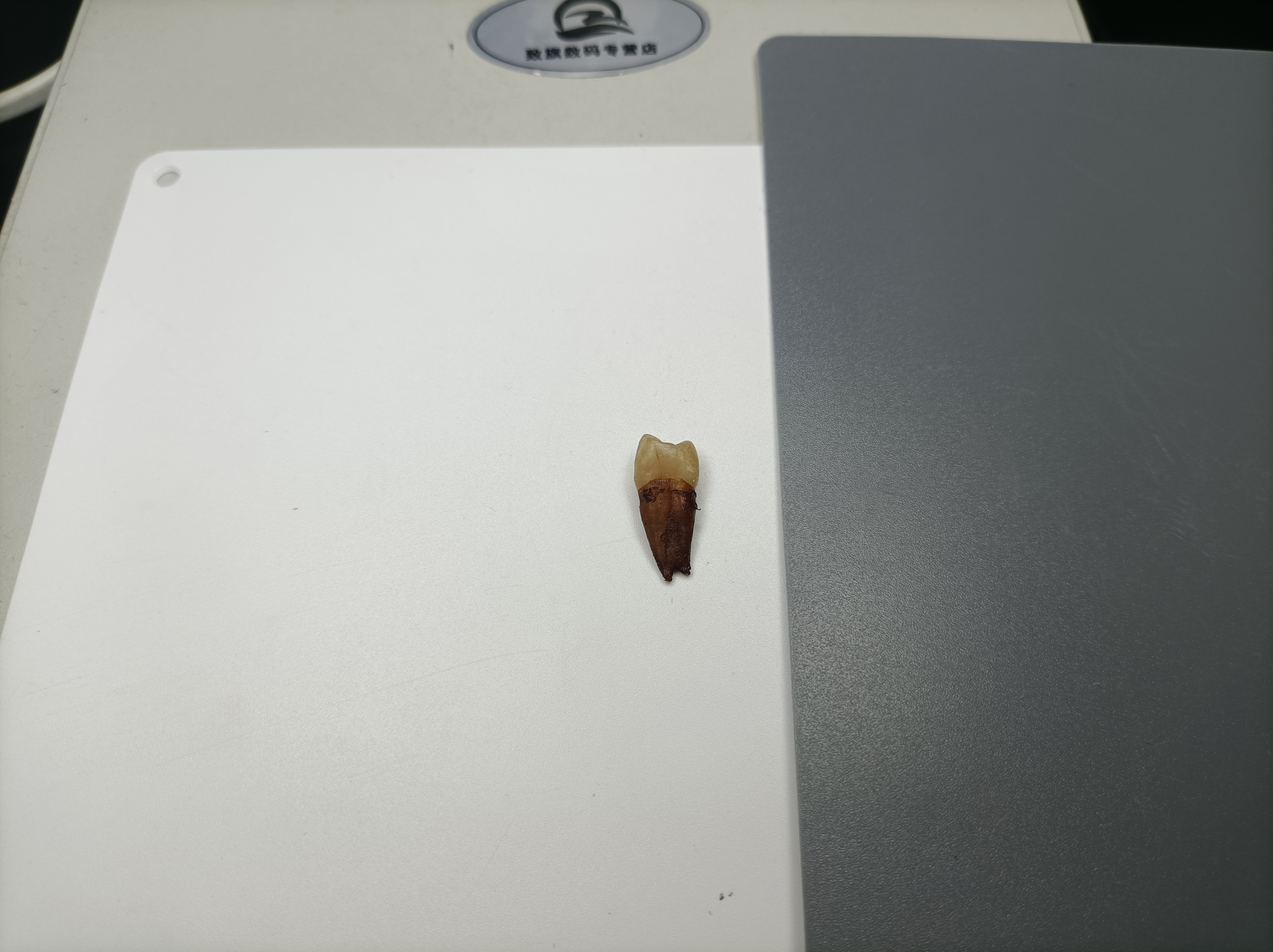

Supplement: Supplementary file 6 — Source data [file 41467_2022_32132_MOESM6_ESM.zip › Source data/main text/Figure 4/Figures/36-46/0.jpg]

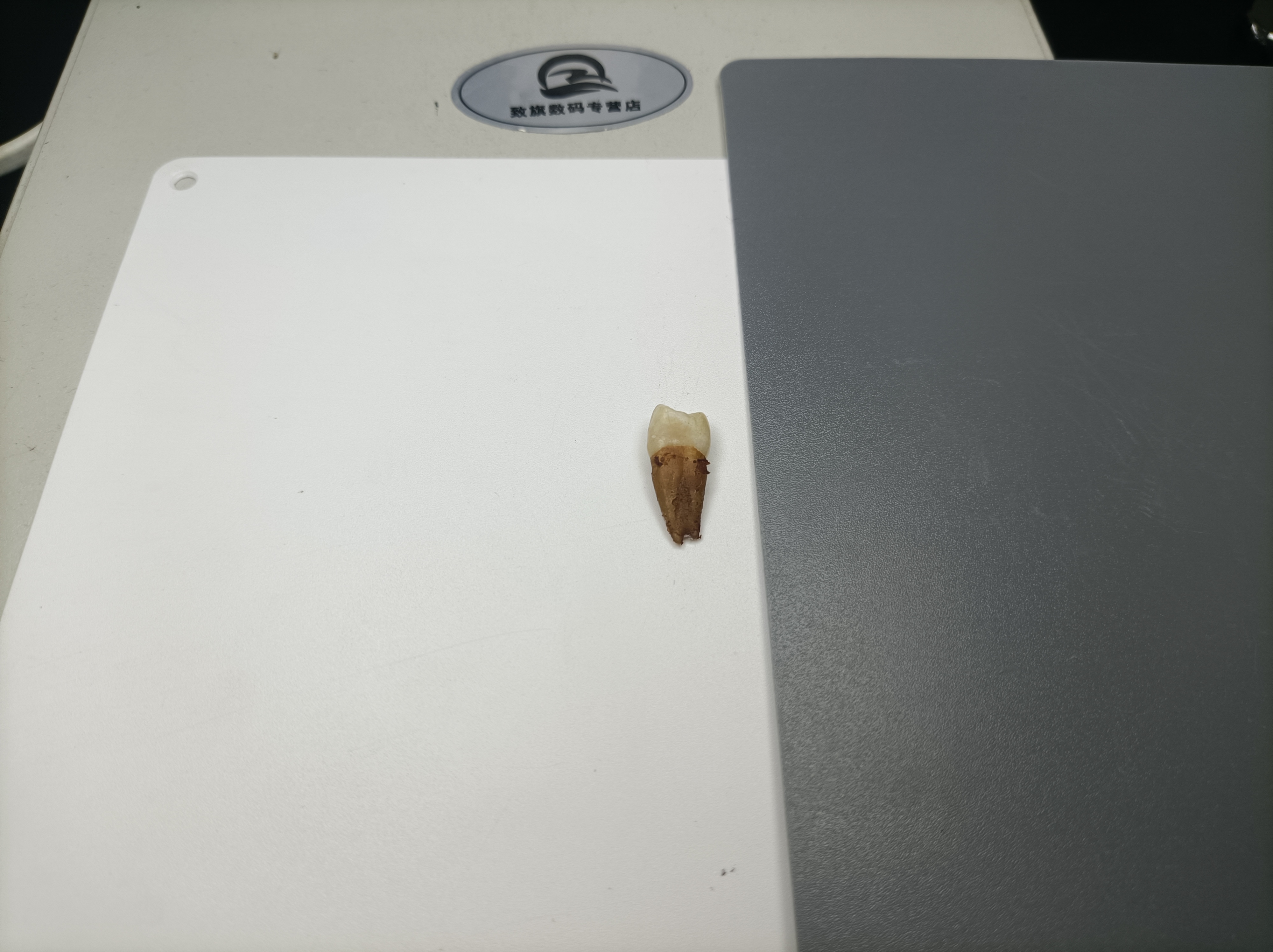

Supplement: Supplementary file 6 — Source data [file 41467_2022_32132_MOESM6_ESM.zip › Source data/main text/Figure 4/Figures/36-46/100.jpg]

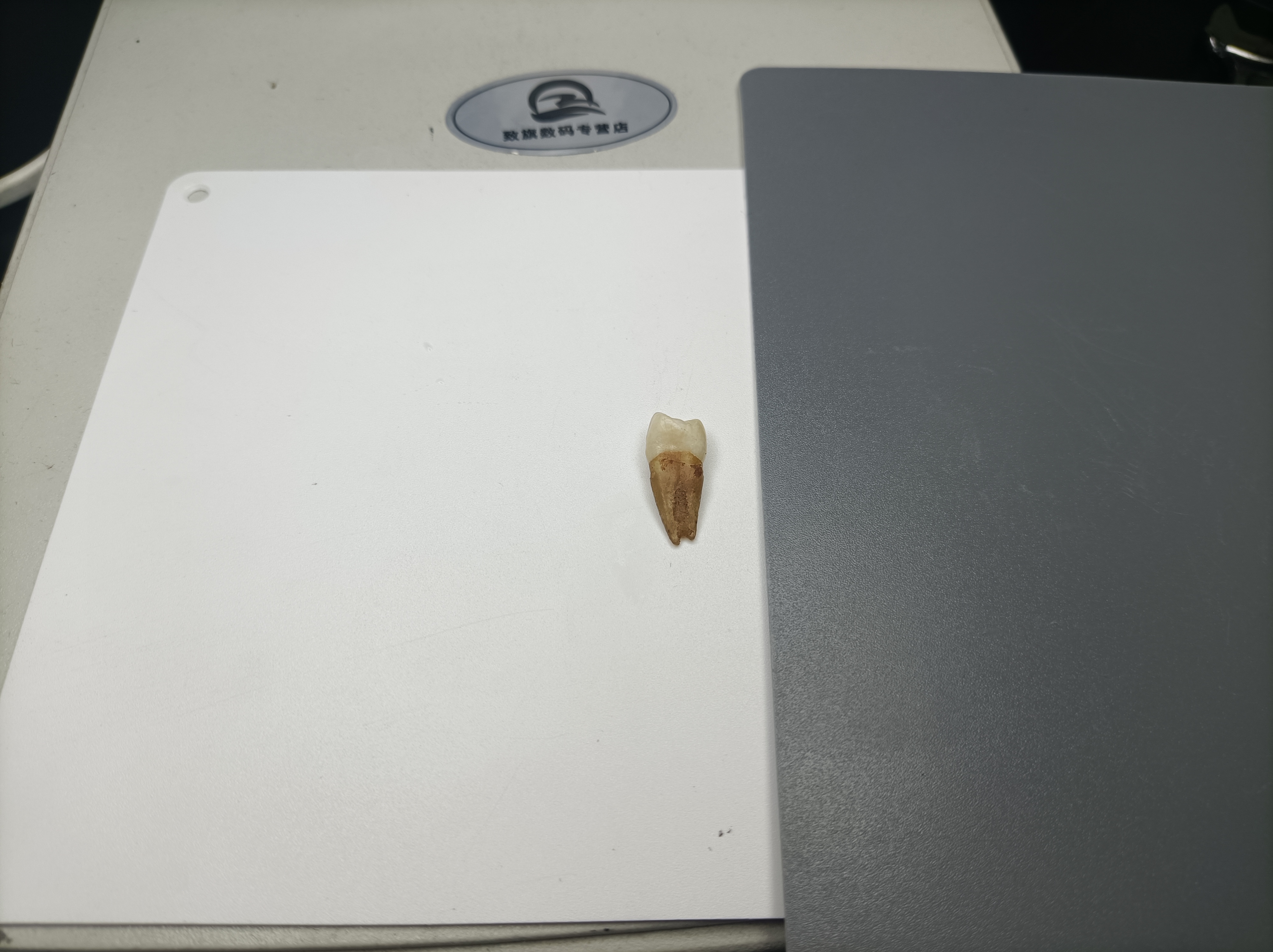

Supplement: Supplementary file 6 — Source data [file 41467_2022_32132_MOESM6_ESM.zip › Source data/main text/Figure 4/Figures/36-46/1000.jpg]

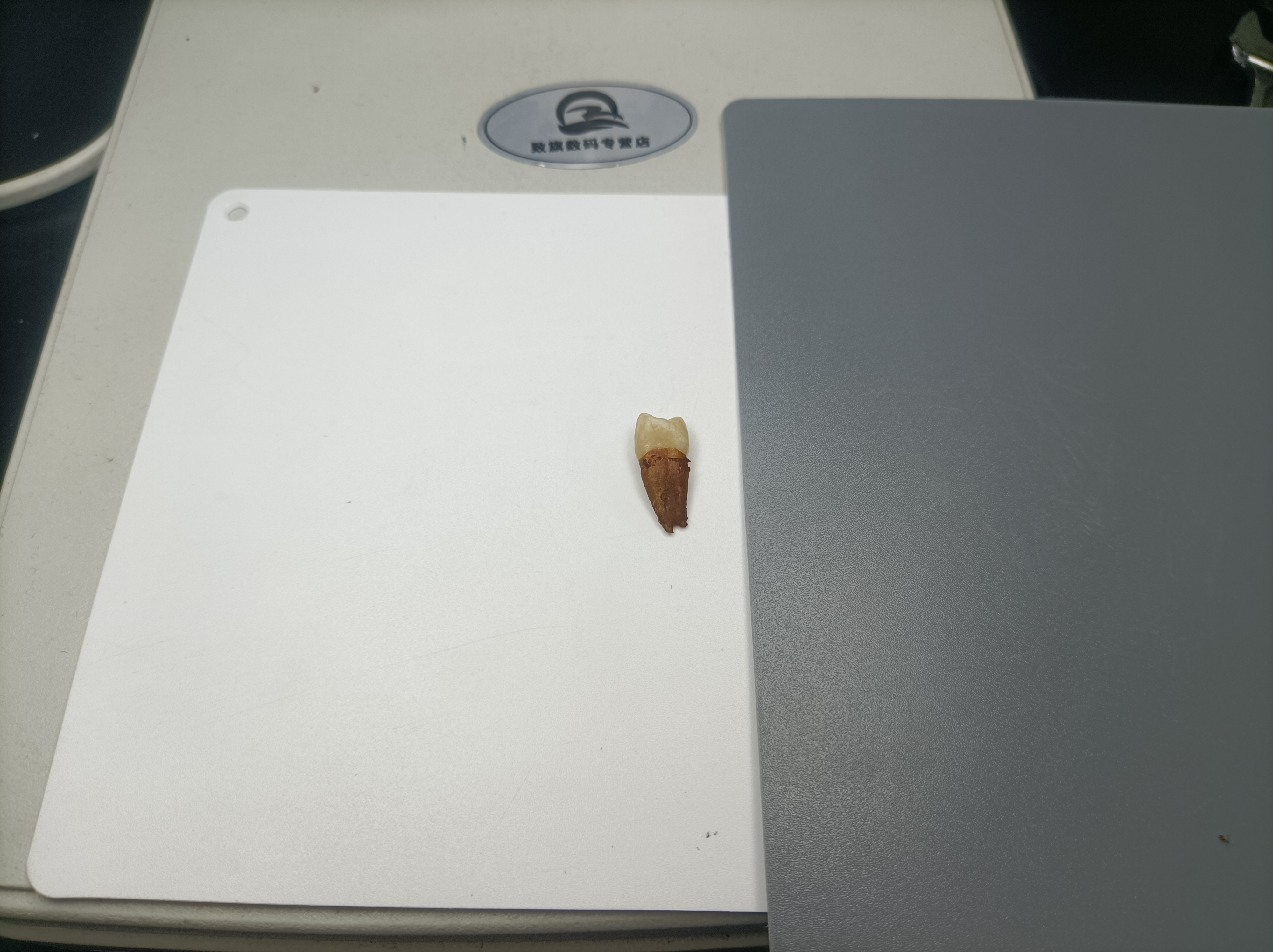

Supplement: Supplementary file 6 — Source data [file 41467_2022_32132_MOESM6_ESM.zip › Source data/main text/Figure 4/Figures/36-46/150.jpg]

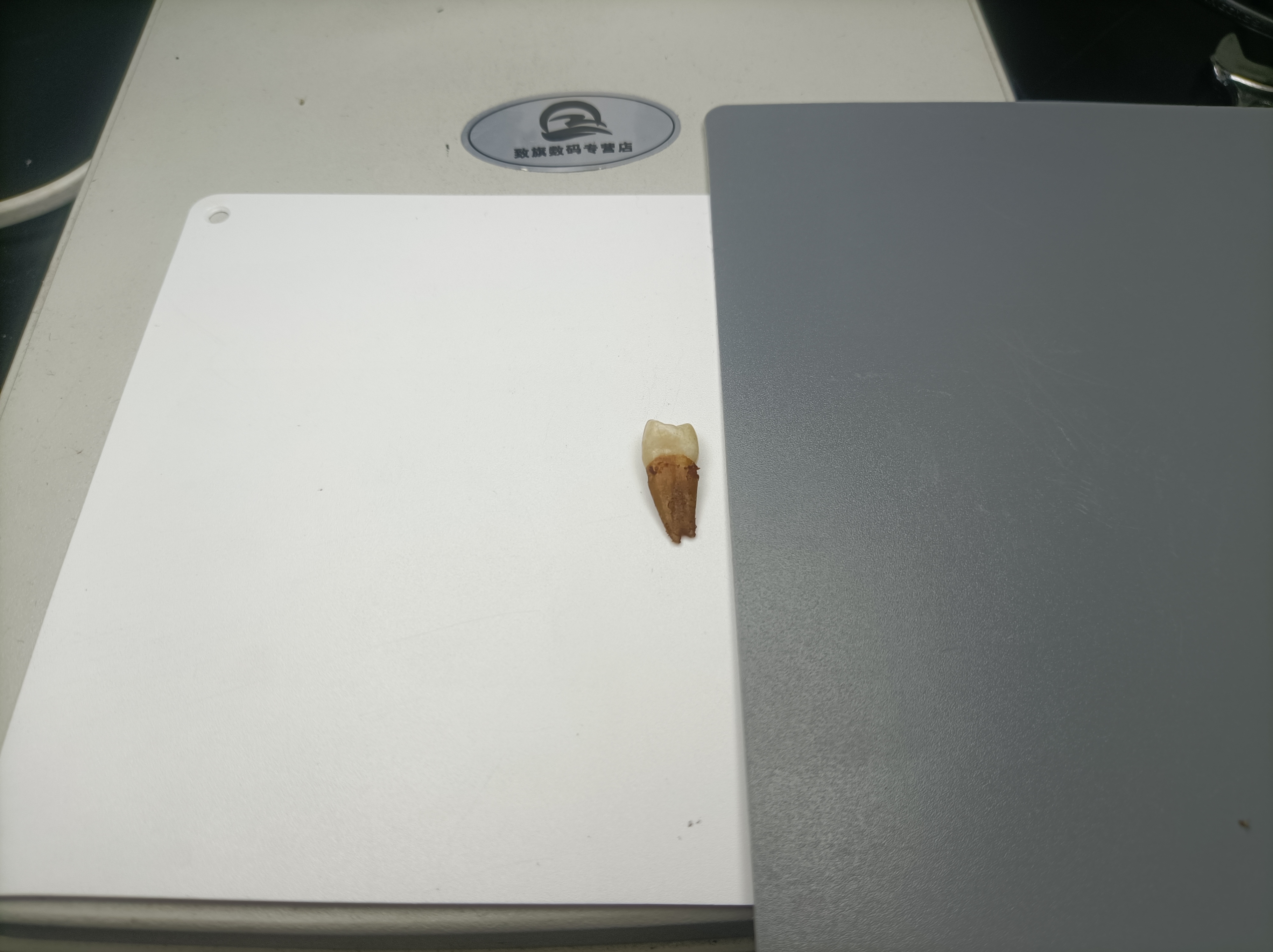

Supplement: Supplementary file 6 — Source data [file 41467_2022_32132_MOESM6_ESM.zip › Source data/main text/Figure 4/Figures/36-46/200.jpg]

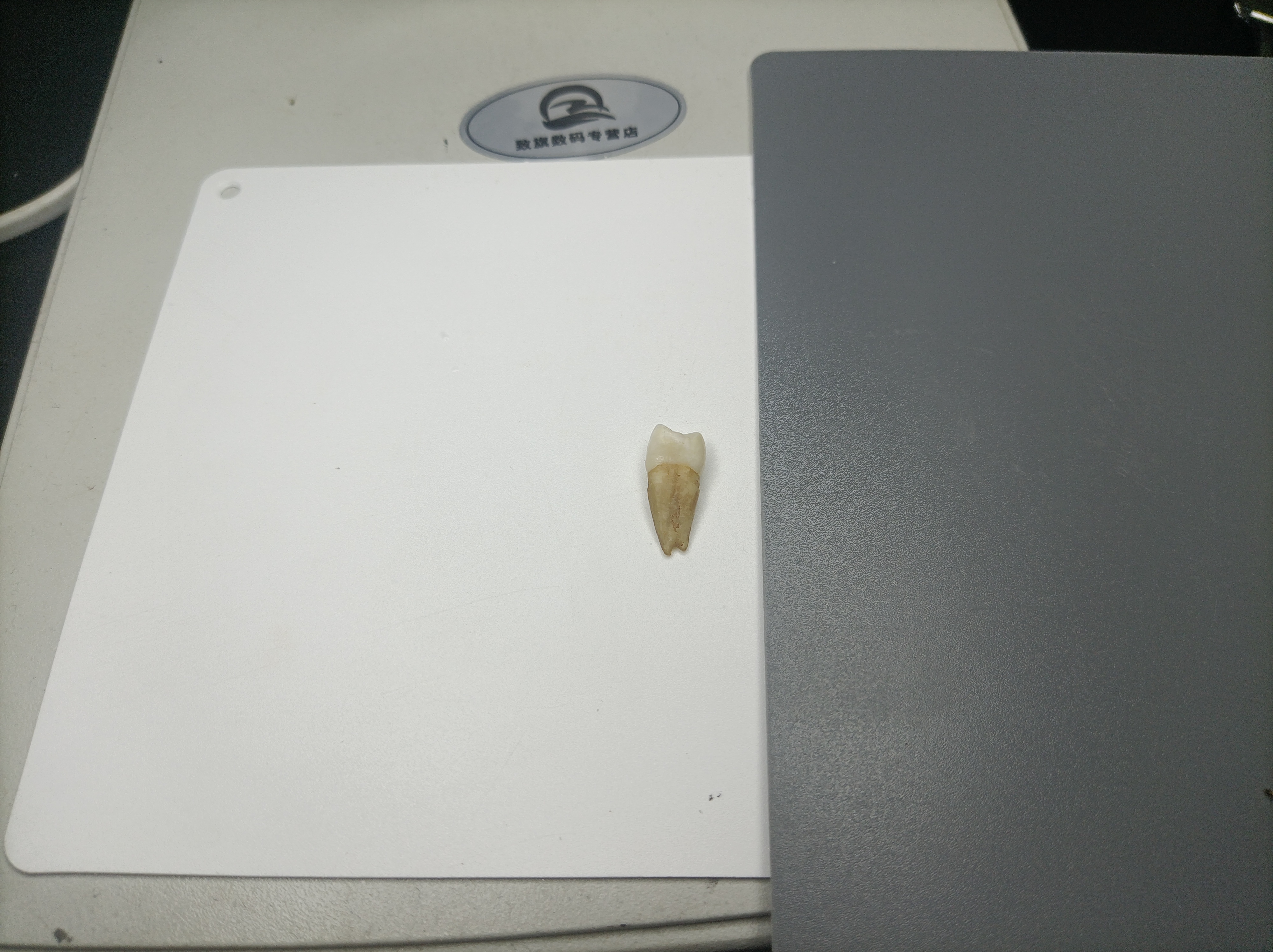

Supplement: Supplementary file 6 — Source data [file 41467_2022_32132_MOESM6_ESM.zip › Source data/main text/Figure 4/Figures/36-46/2000.jpg]

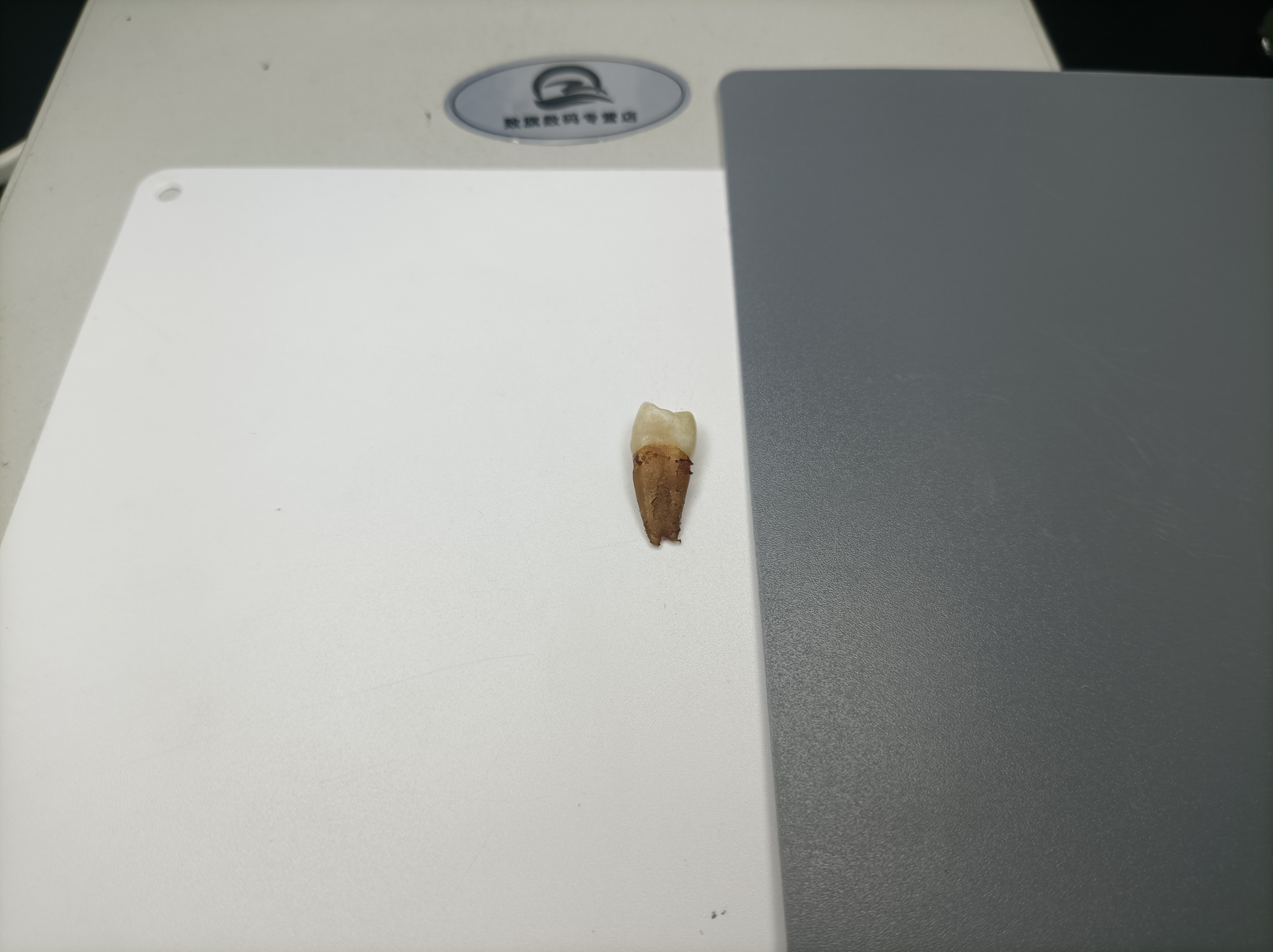

Supplement: Supplementary file 6 — Source data [file 41467_2022_32132_MOESM6_ESM.zip › Source data/main text/Figure 4/Figures/36-46/300.jpg]

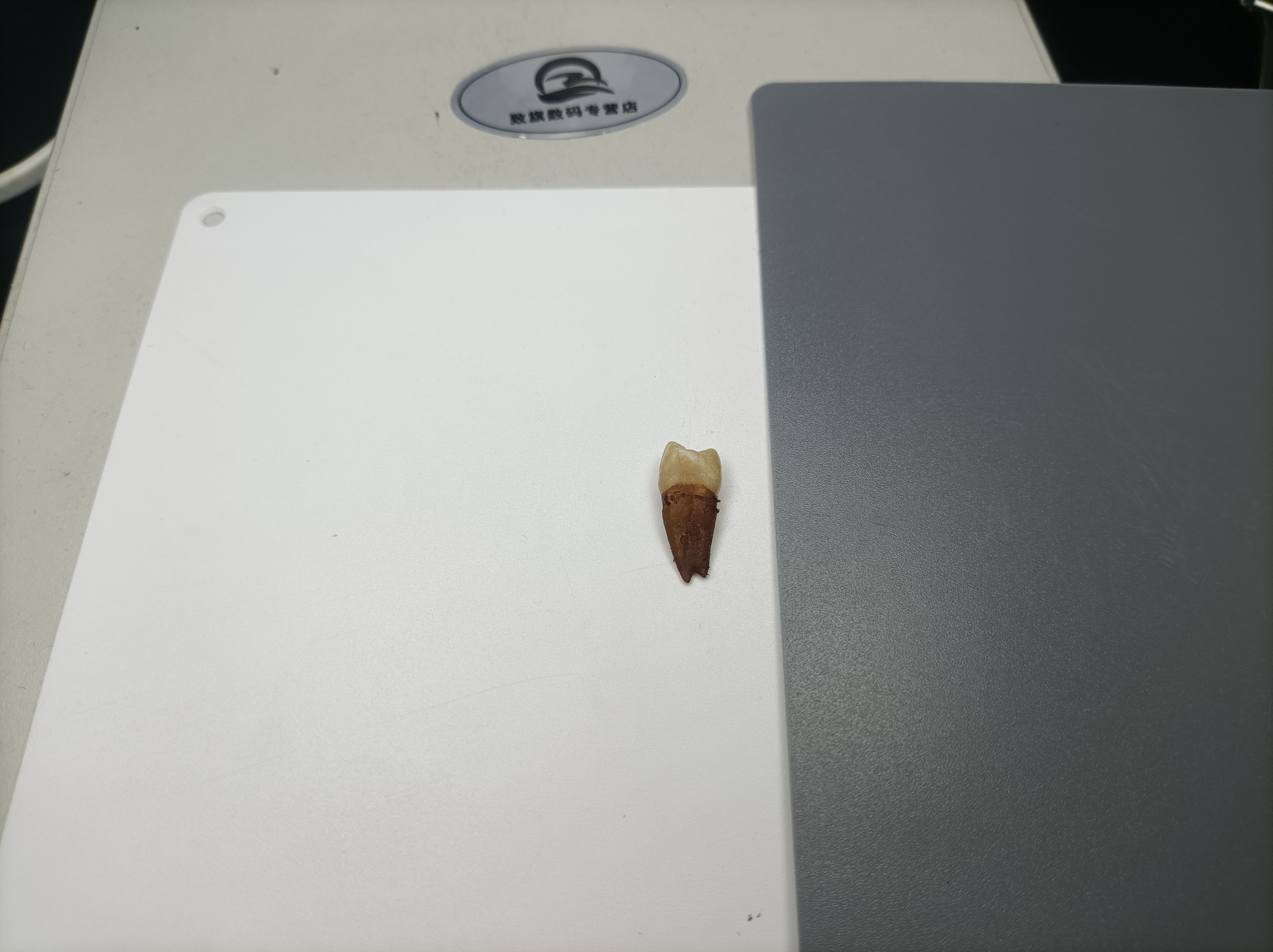

Supplement: Supplementary file 6 — Source data [file 41467_2022_32132_MOESM6_ESM.zip › Source data/main text/Figure 4/Figures/36-46/50.jpg]

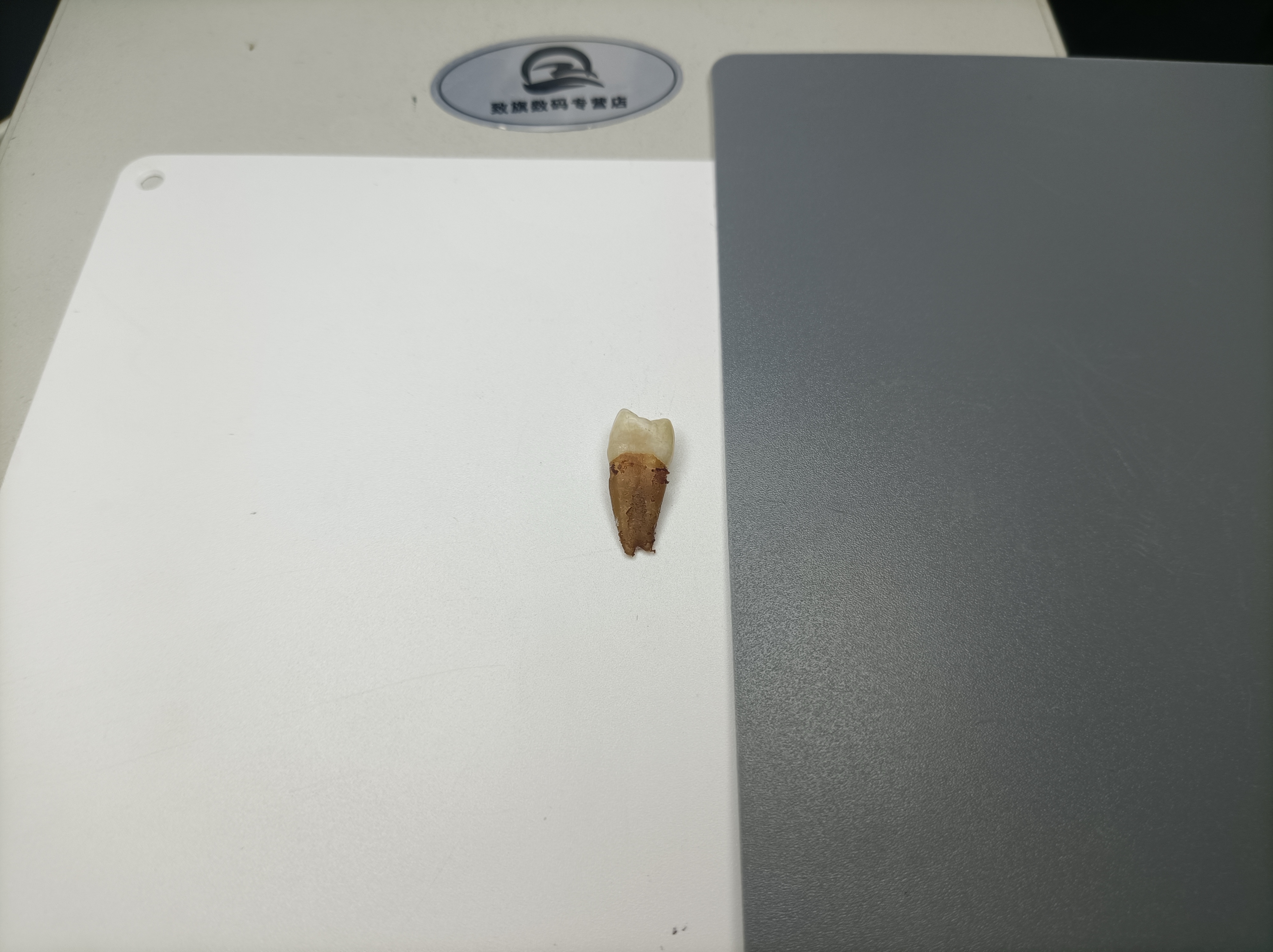

Supplement: Supplementary file 6 — Source data [file 41467_2022_32132_MOESM6_ESM.zip › Source data/main text/Figure 4/Figures/36-46/500.jpg]

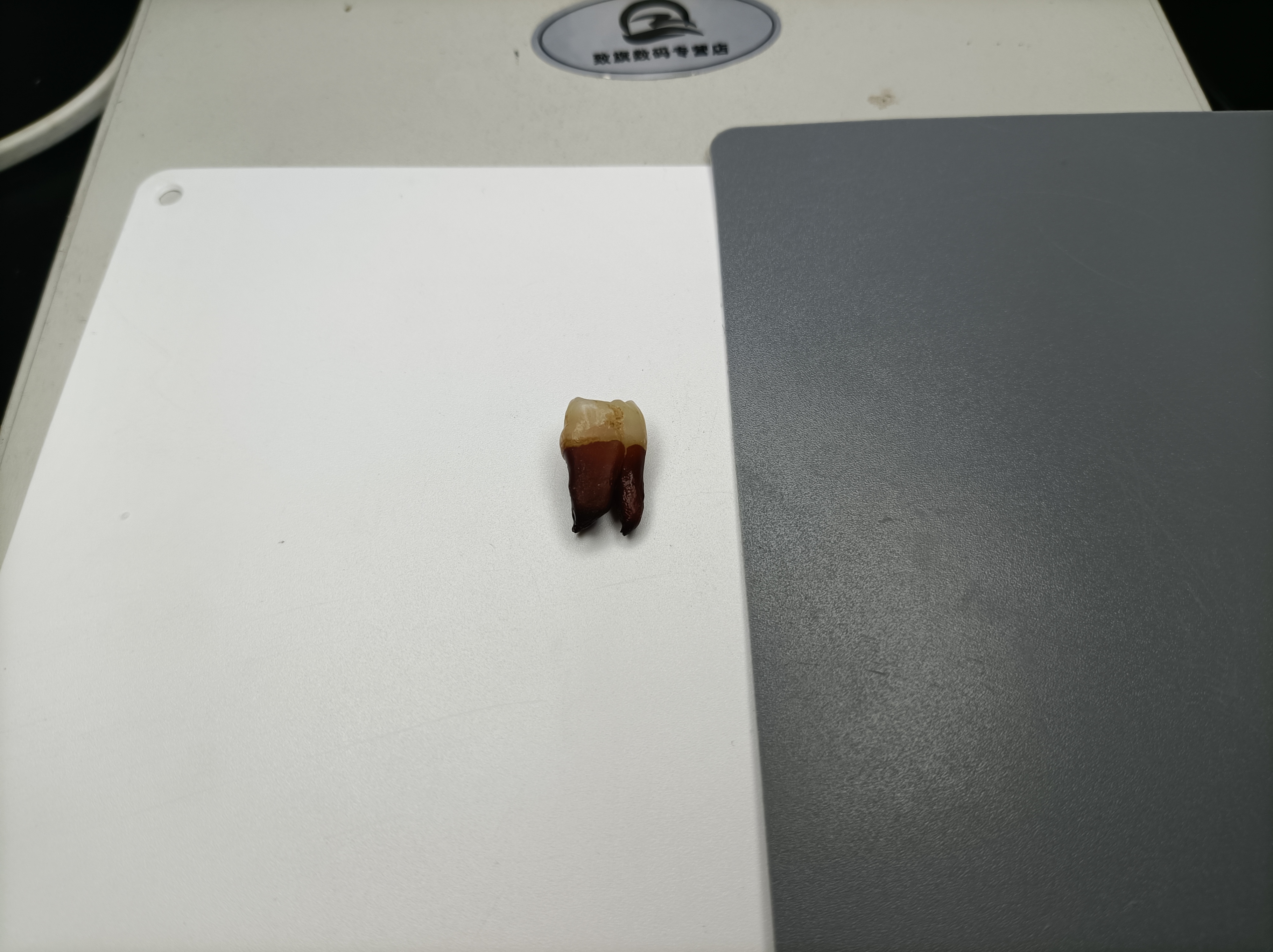

Supplement: Supplementary file 6 — Source data [file 41467_2022_32132_MOESM6_ESM.zip › Source data/main text/Figure 4/Figures/36-61/0.jpg]

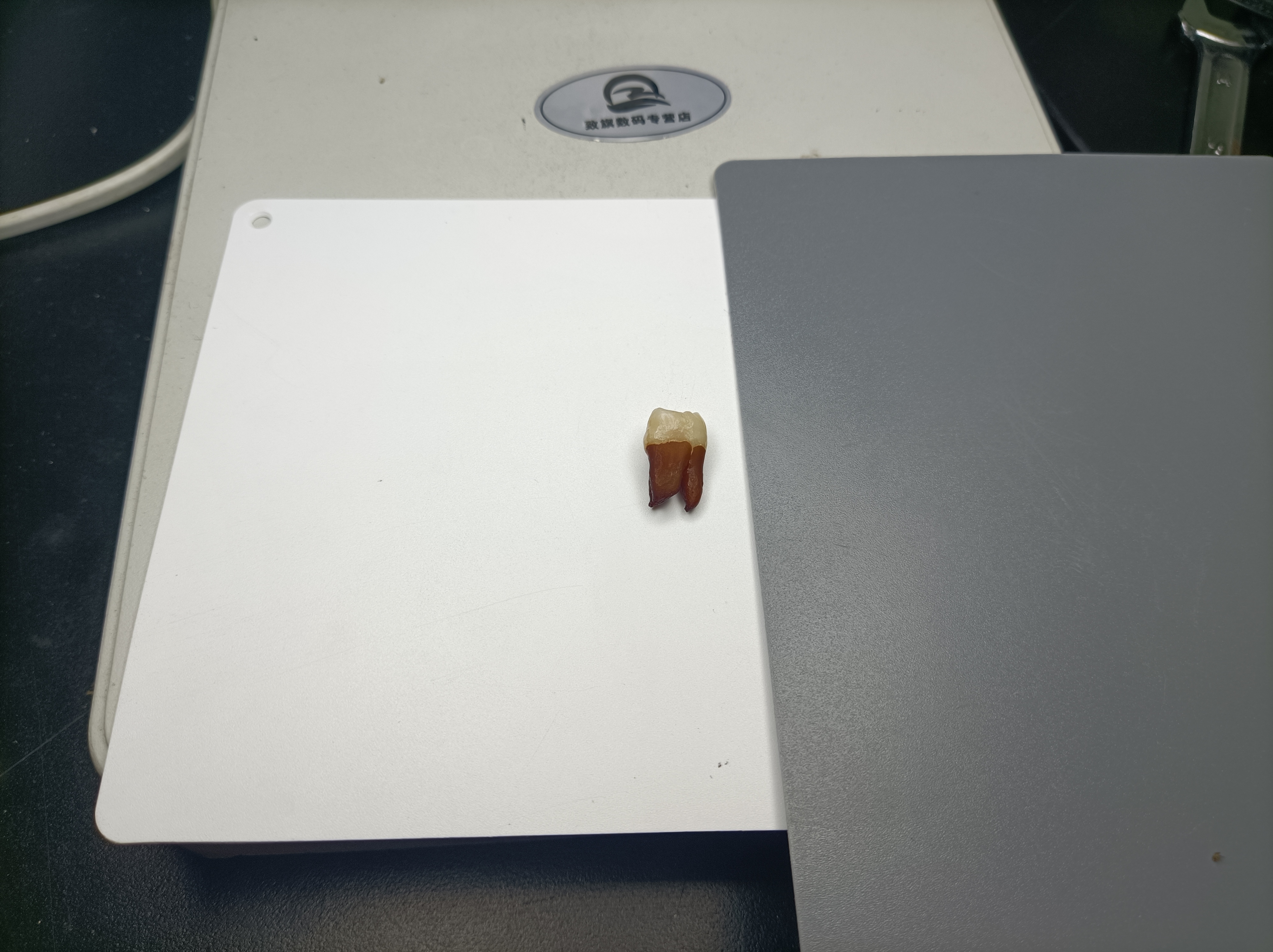

Supplement: Supplementary file 6 — Source data [file 41467_2022_32132_MOESM6_ESM.zip › Source data/main text/Figure 4/Figures/36-61/100.jpg]

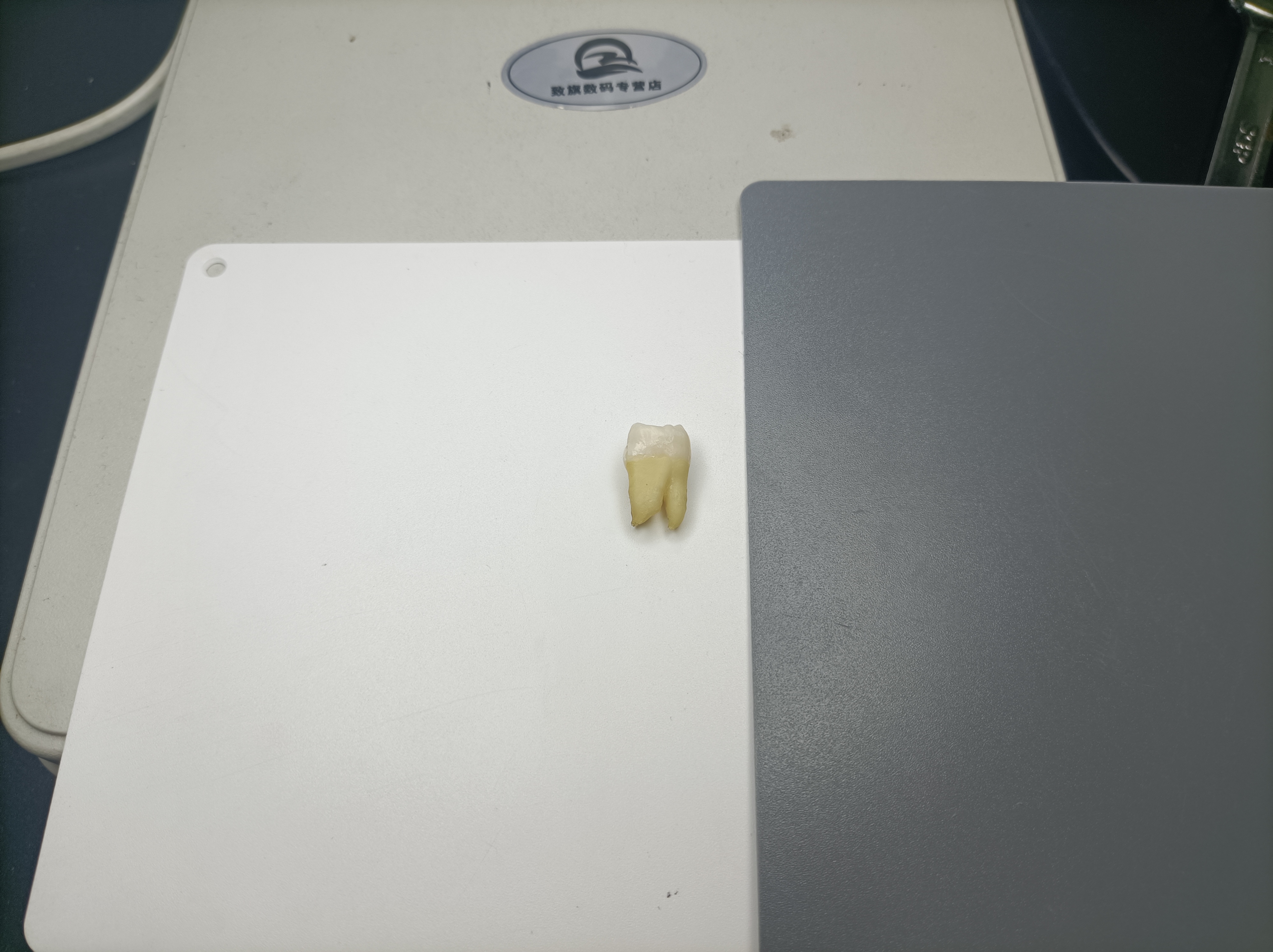

Supplement: Supplementary file 6 — Source data [file 41467_2022_32132_MOESM6_ESM.zip › Source data/main text/Figure 4/Figures/36-61/1000.jpg]

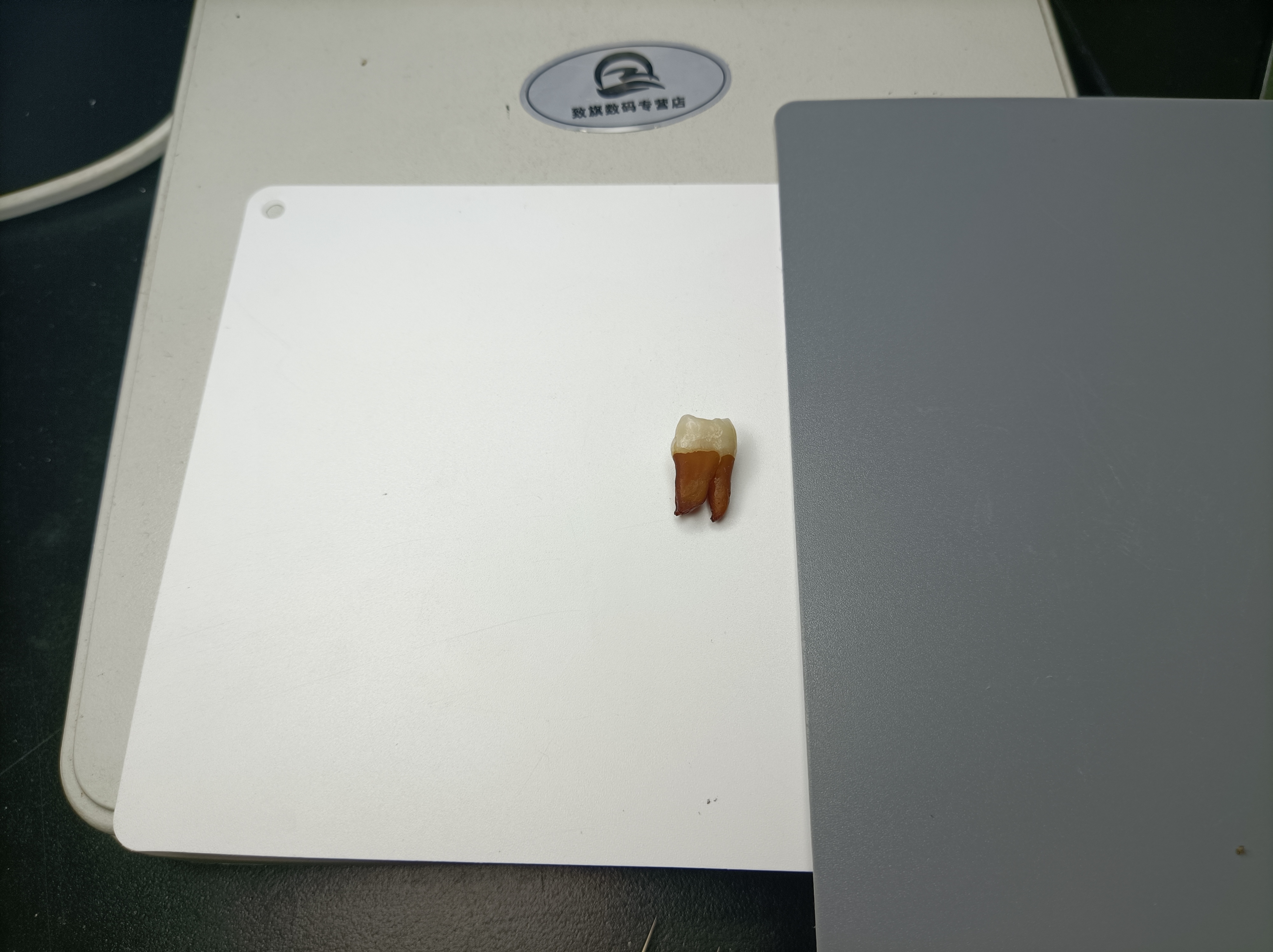

Supplement: Supplementary file 6 — Source data [file 41467_2022_32132_MOESM6_ESM.zip › Source data/main text/Figure 4/Figures/36-61/150.jpg]

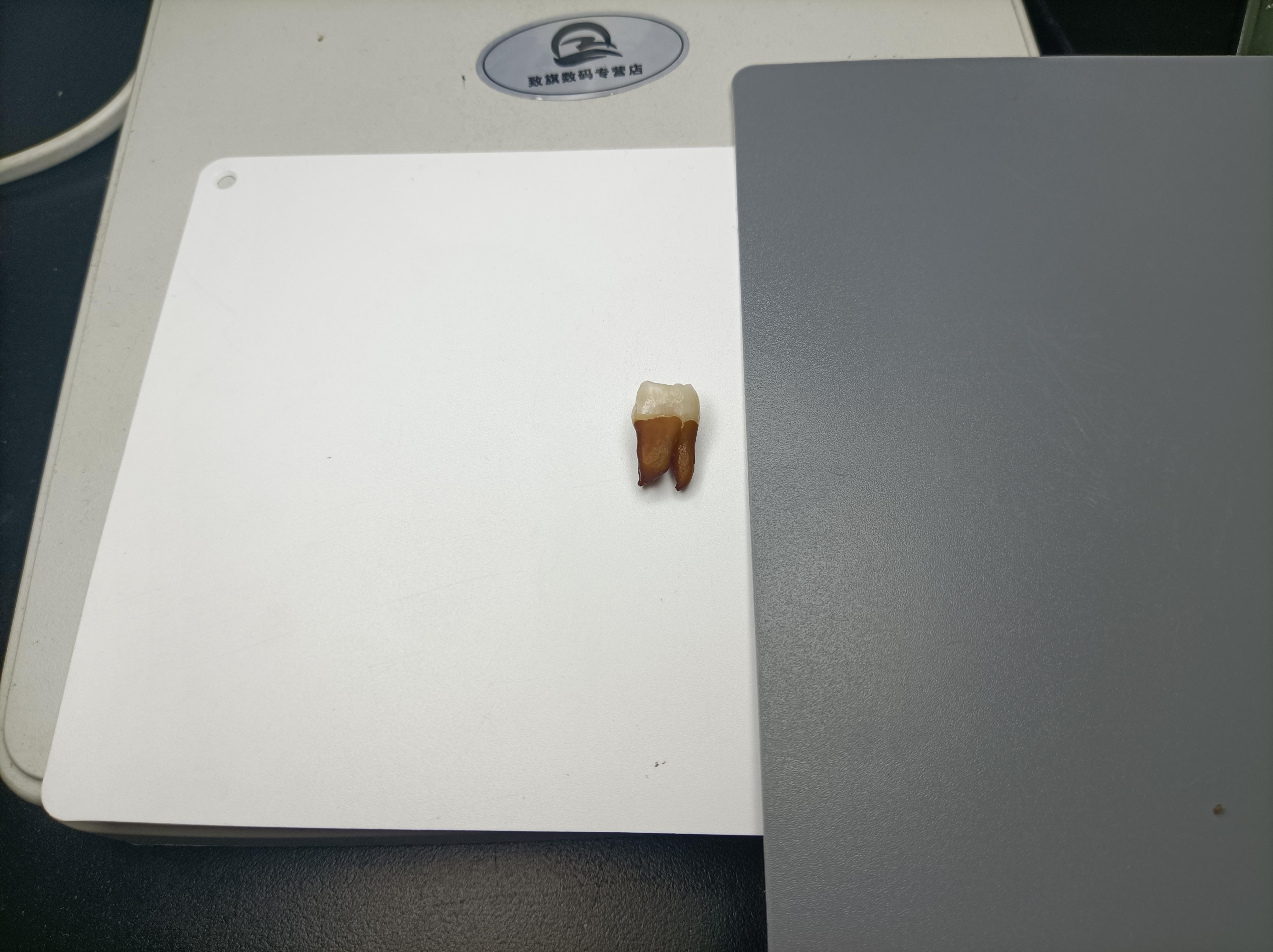

Supplement: Supplementary file 6 — Source data [file 41467_2022_32132_MOESM6_ESM.zip › Source data/main text/Figure 4/Figures/36-61/200.jpg]

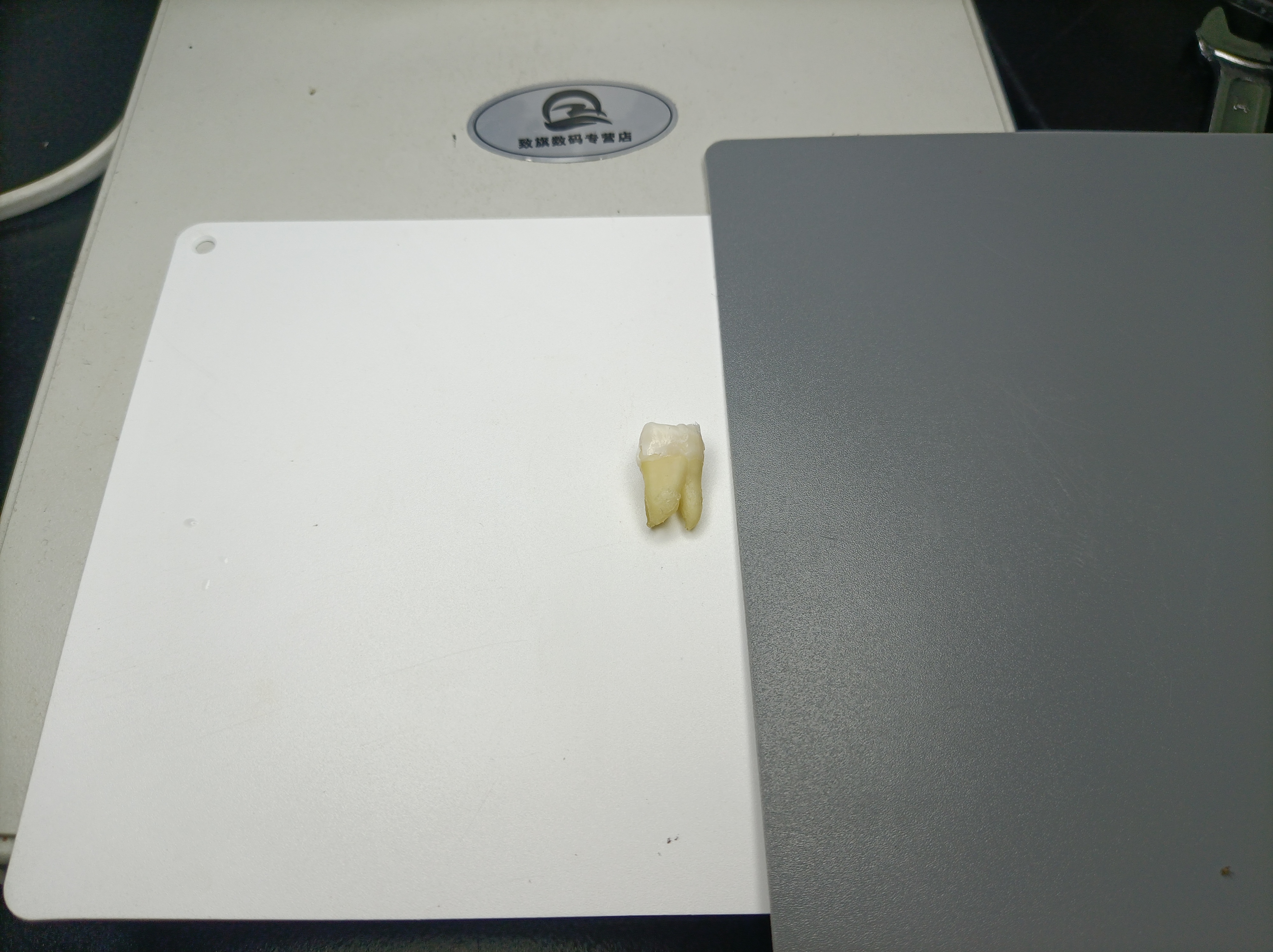

Supplement: Supplementary file 6 — Source data [file 41467_2022_32132_MOESM6_ESM.zip › Source data/main text/Figure 4/Figures/36-61/2000.jpg]

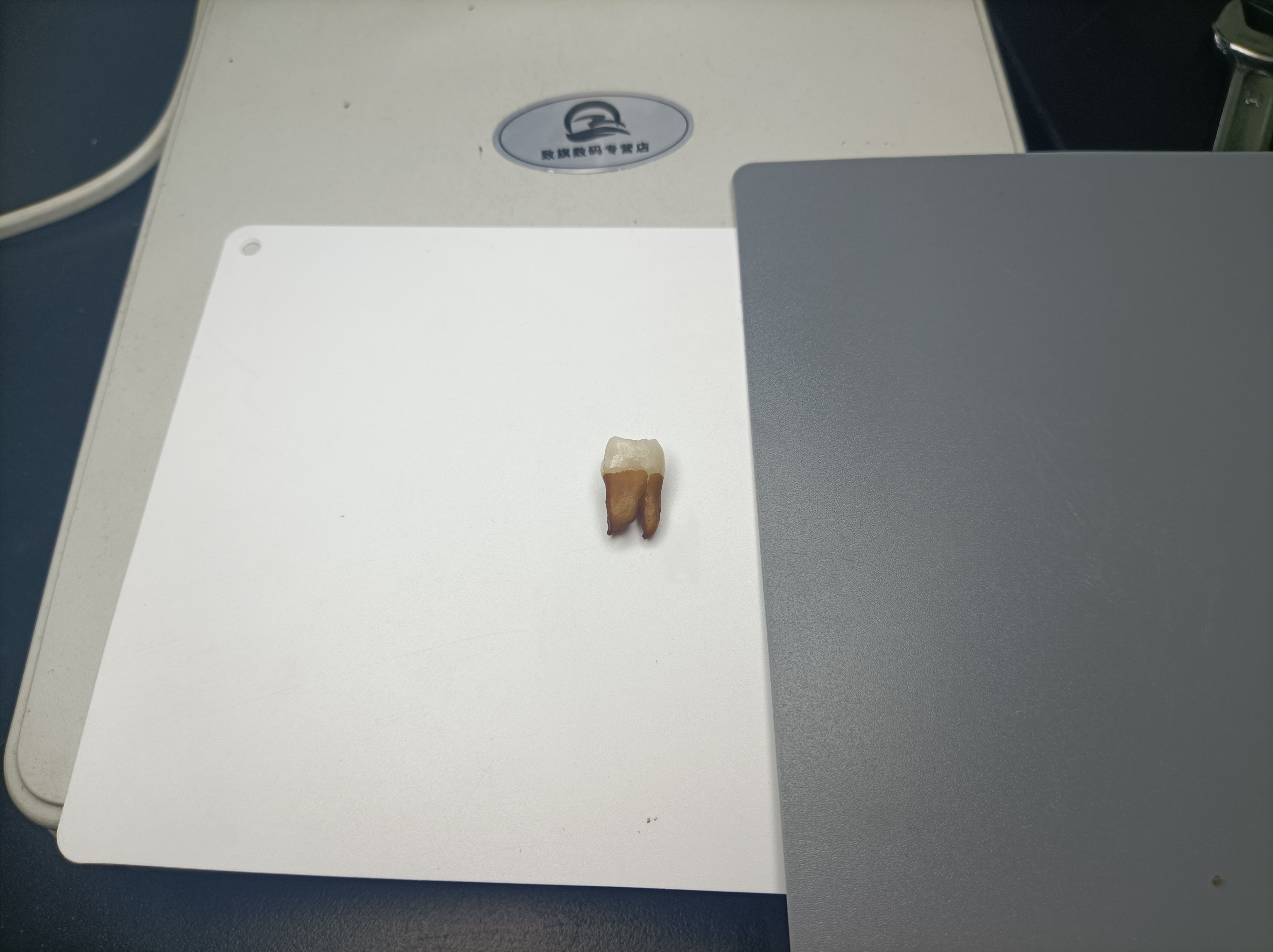

Supplement: Supplementary file 6 — Source data [file 41467_2022_32132_MOESM6_ESM.zip › Source data/main text/Figure 4/Figures/36-61/300.jpg]

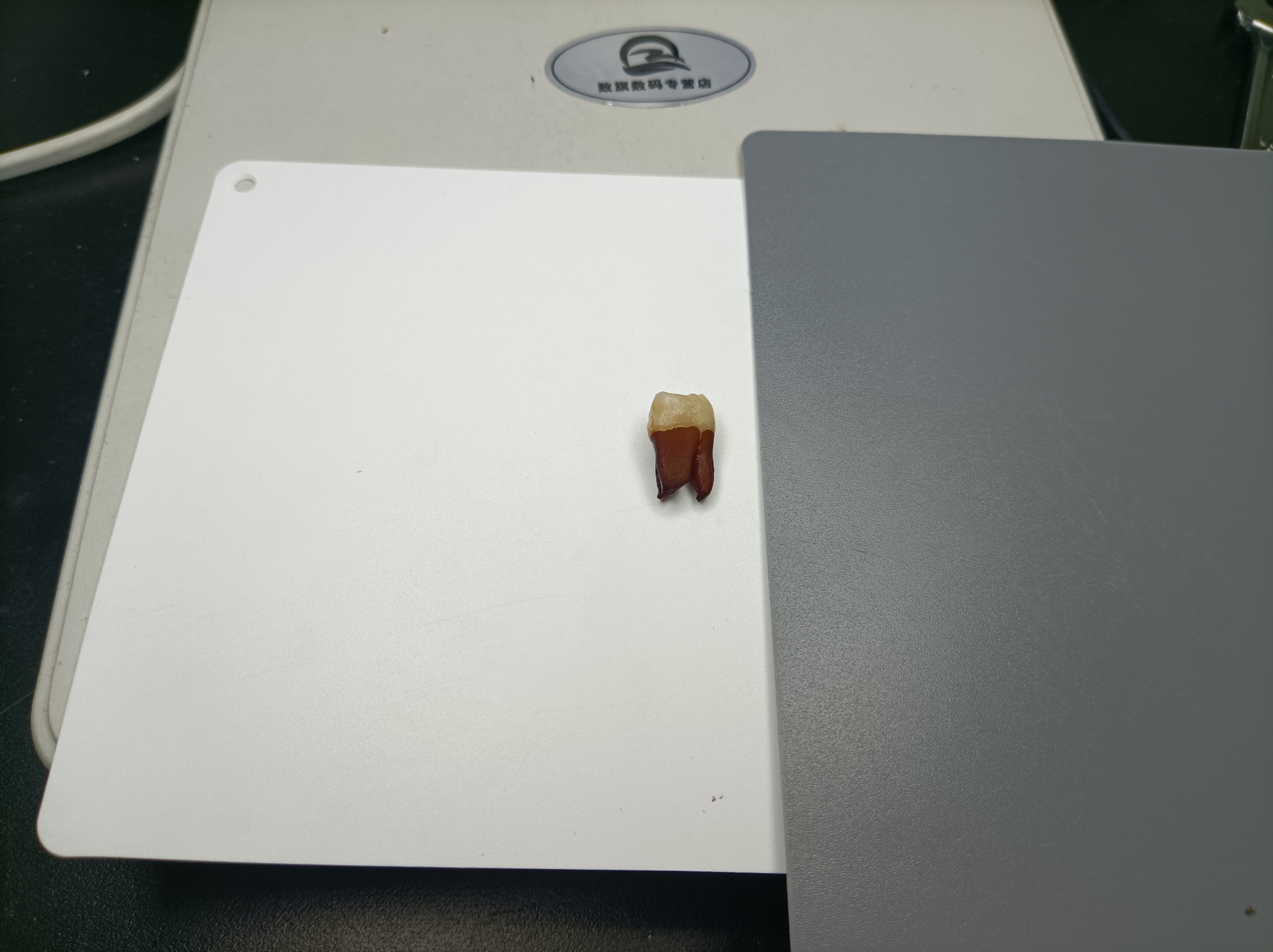

Supplement: Supplementary file 6 — Source data [file 41467_2022_32132_MOESM6_ESM.zip › Source data/main text/Figure 4/Figures/36-61/50.jpg]

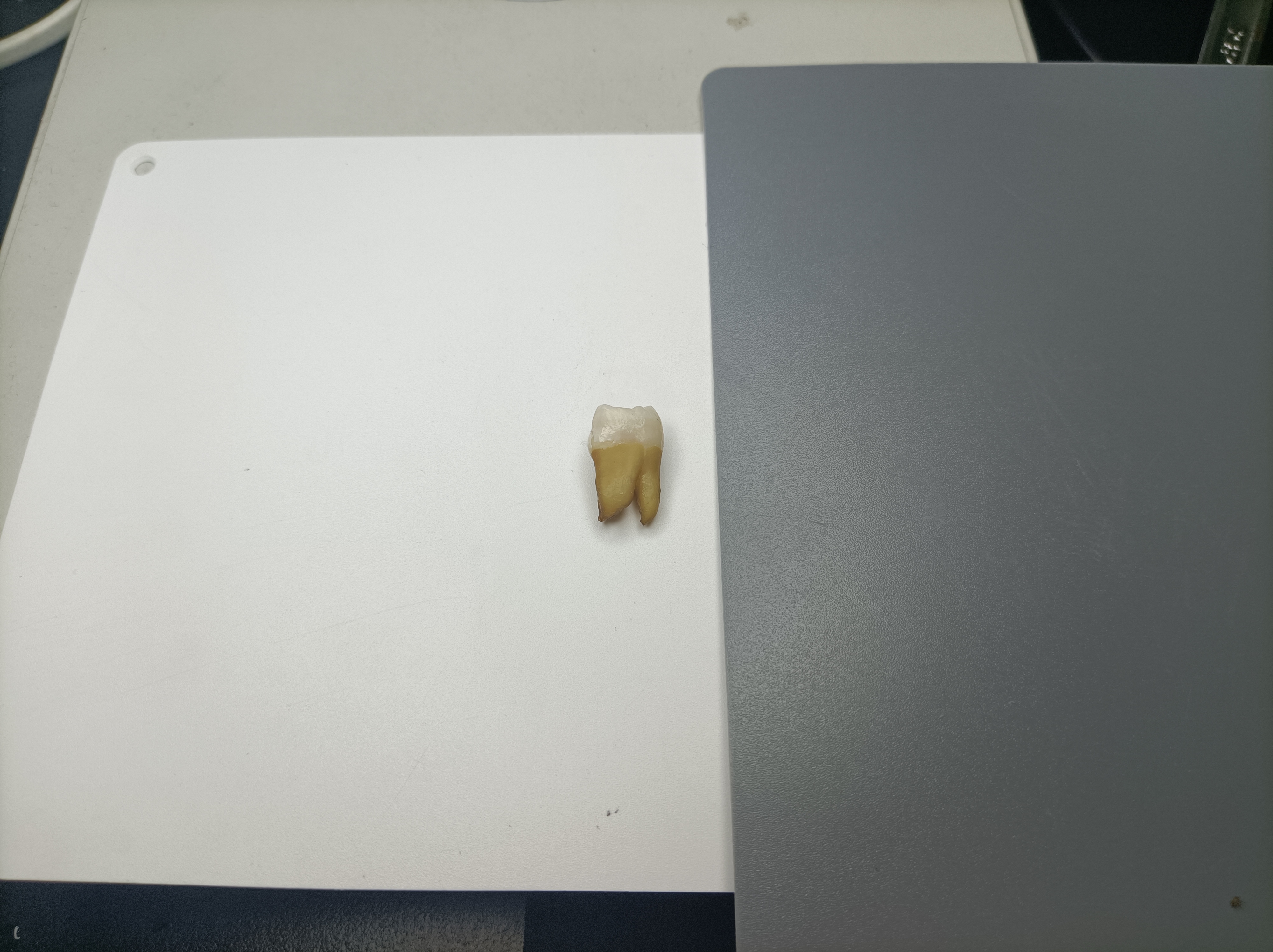

Supplement: Supplementary file 6 — Source data [file 41467_2022_32132_MOESM6_ESM.zip › Source data/main text/Figure 4/Figures/36-61/500.jpg]

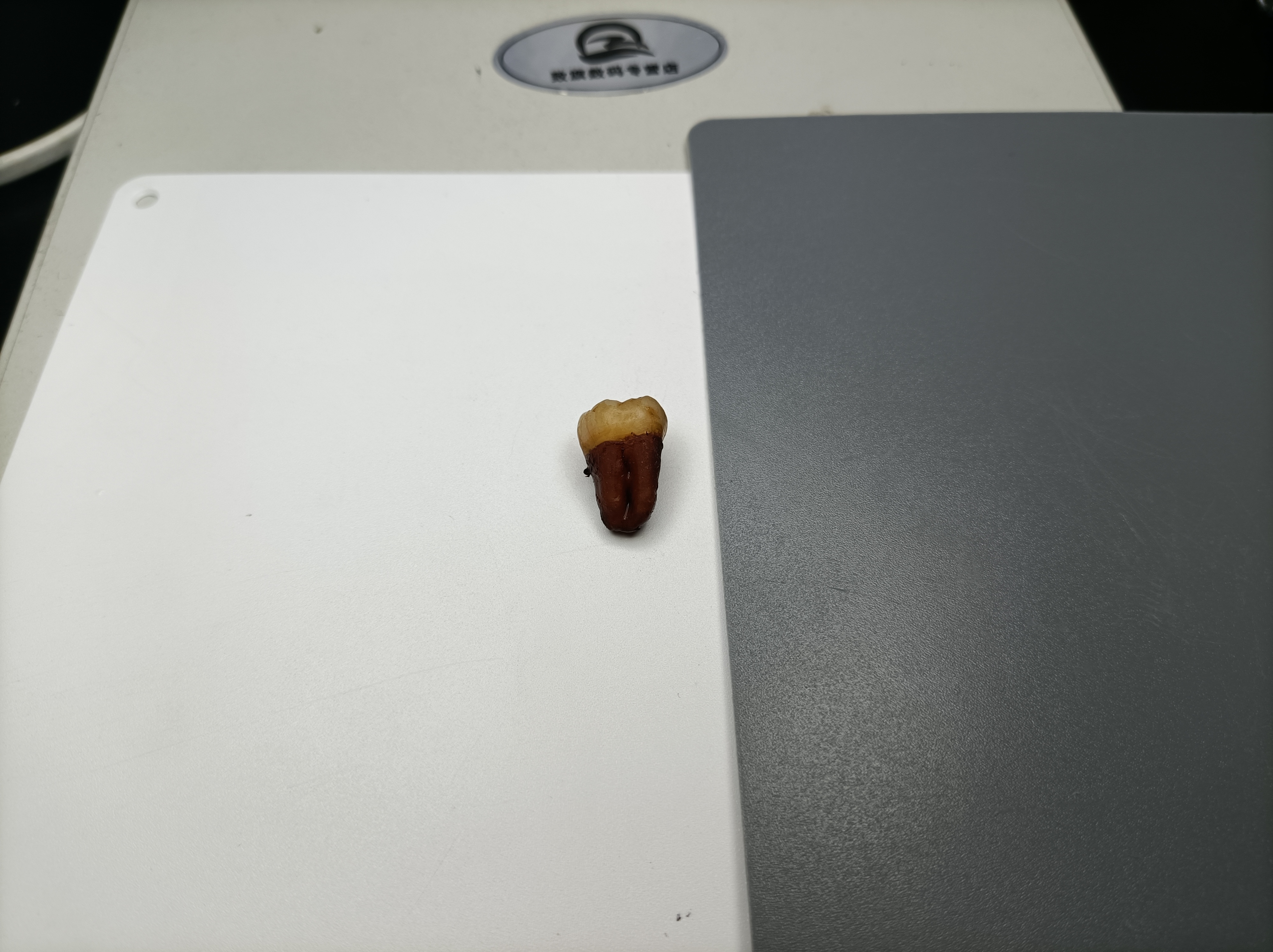

Supplement: Supplementary file 6 — Source data [file 41467_2022_32132_MOESM6_ESM.zip › Source data/main text/Figure 4/Figures/without BTO 36-61/0.jpg]

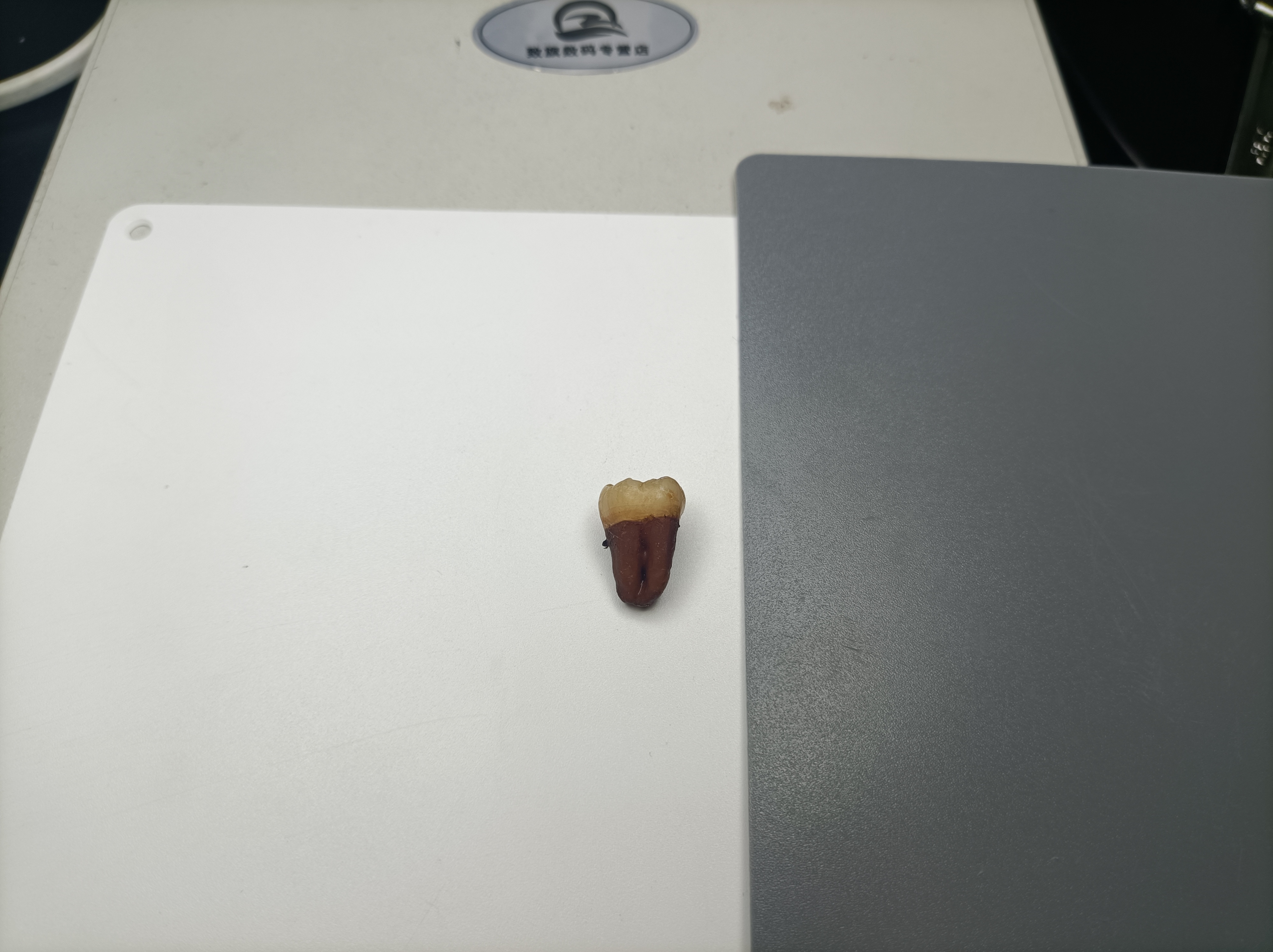

Supplement: Supplementary file 6 — Source data [file 41467_2022_32132_MOESM6_ESM.zip › Source data/main text/Figure 4/Figures/without BTO 36-61/100.jpg]

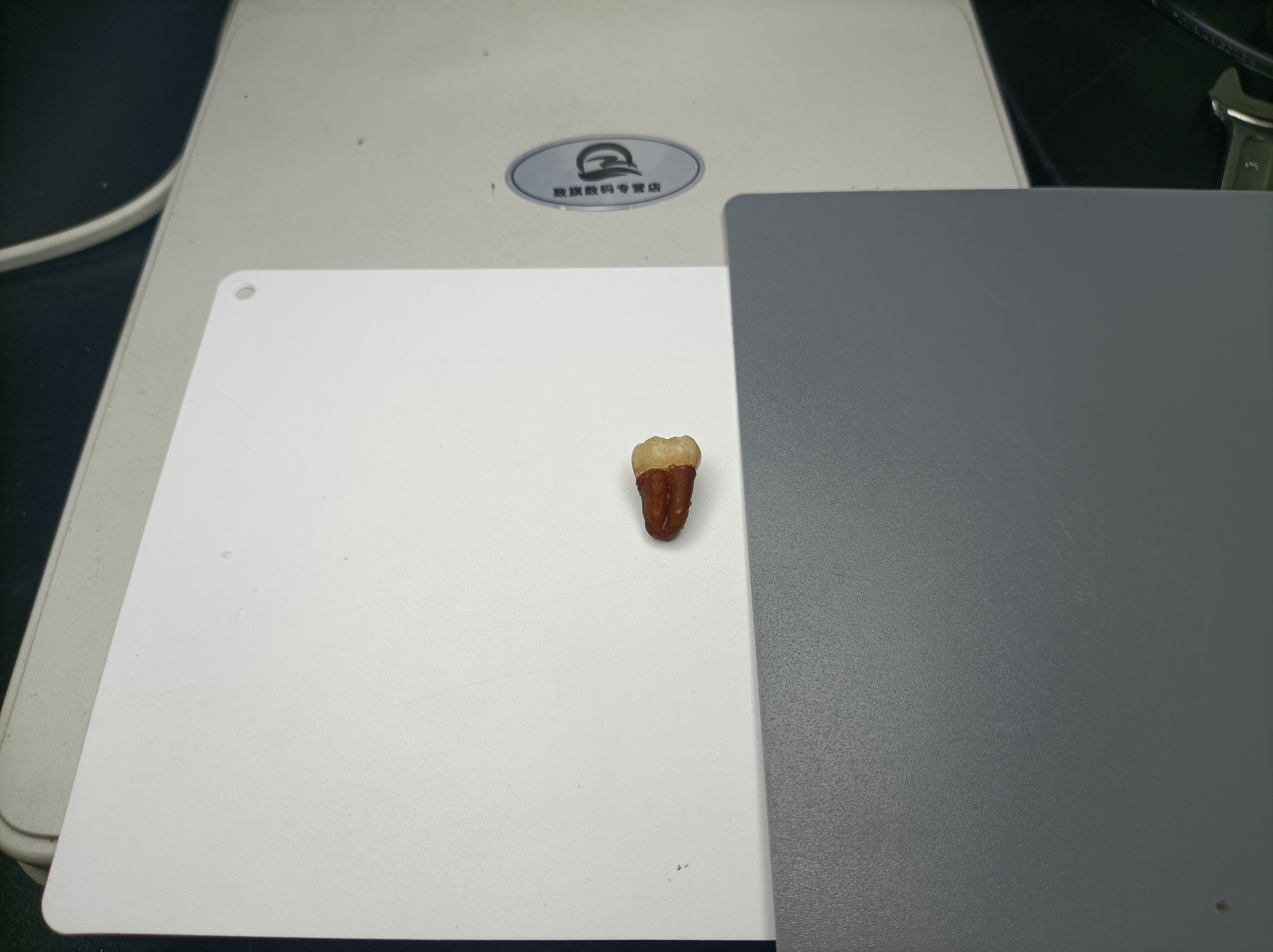

Supplement: Supplementary file 6 — Source data [file 41467_2022_32132_MOESM6_ESM.zip › Source data/main text/Figure 4/Figures/without BTO 36-61/1000.jpg]

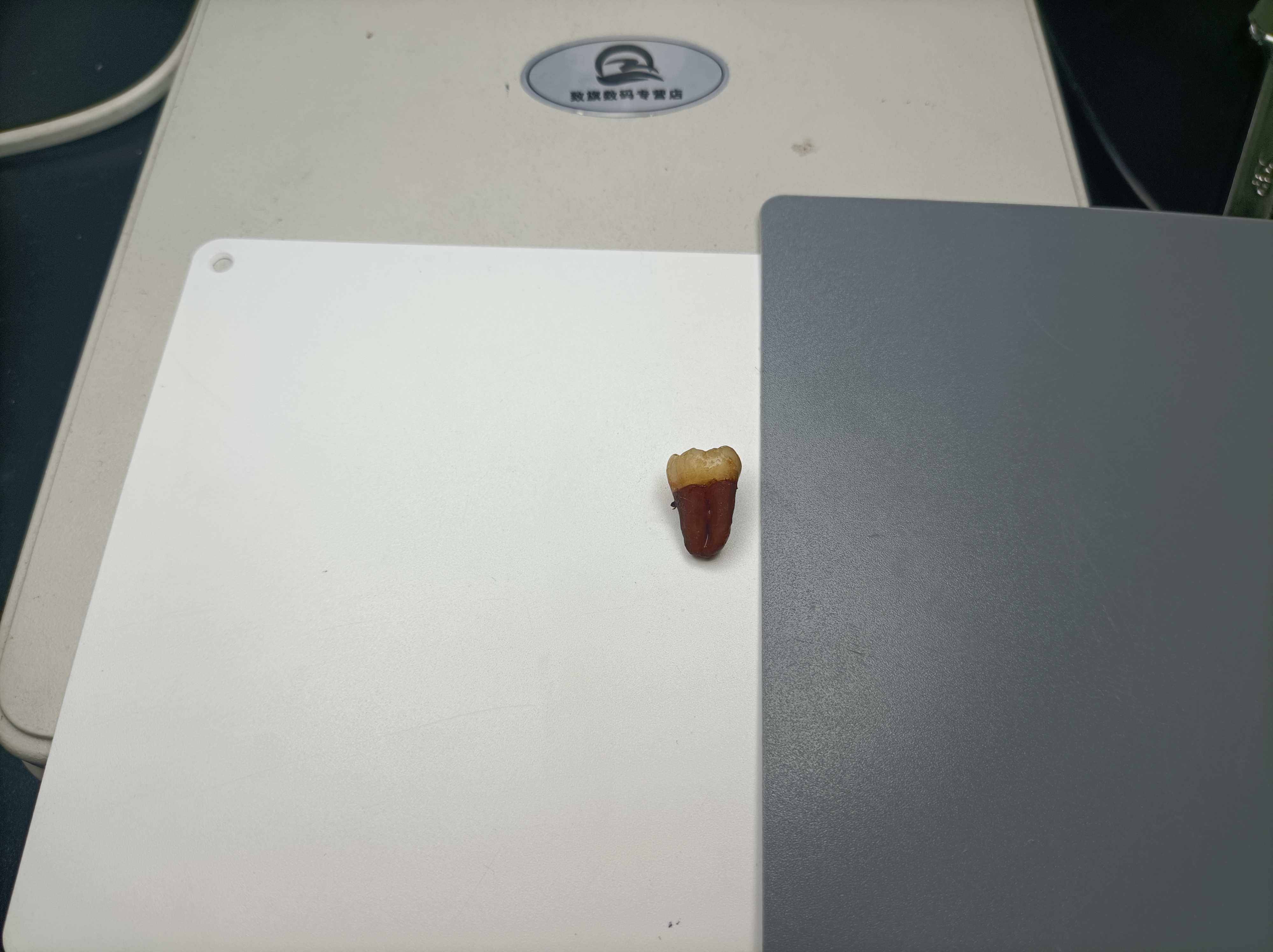

Supplement: Supplementary file 6 — Source data [file 41467_2022_32132_MOESM6_ESM.zip › Source data/main text/Figure 4/Figures/without BTO 36-61/150.jpg]

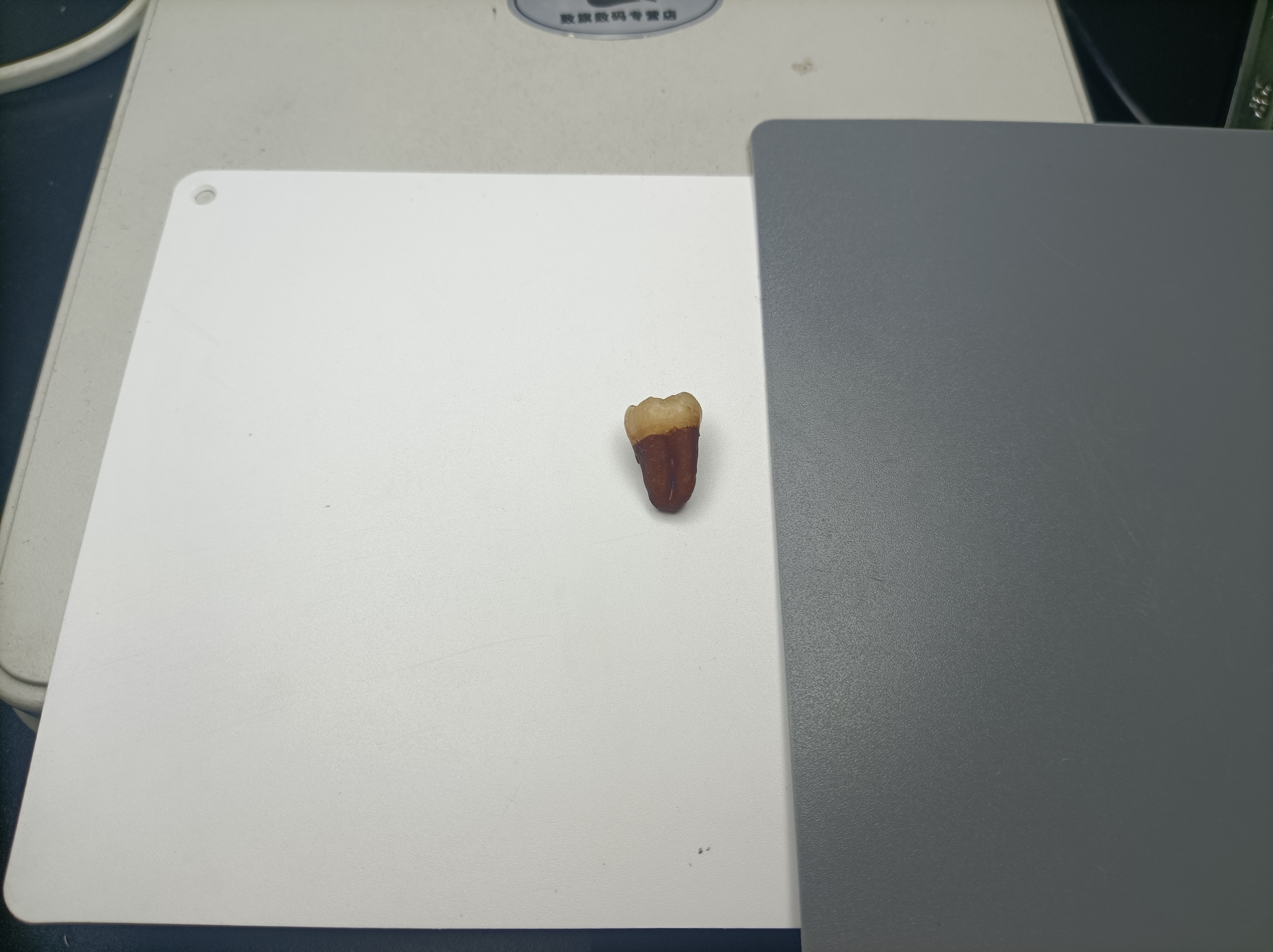

Supplement: Supplementary file 6 — Source data [file 41467_2022_32132_MOESM6_ESM.zip › Source data/main text/Figure 4/Figures/without BTO 36-61/200.jpg]

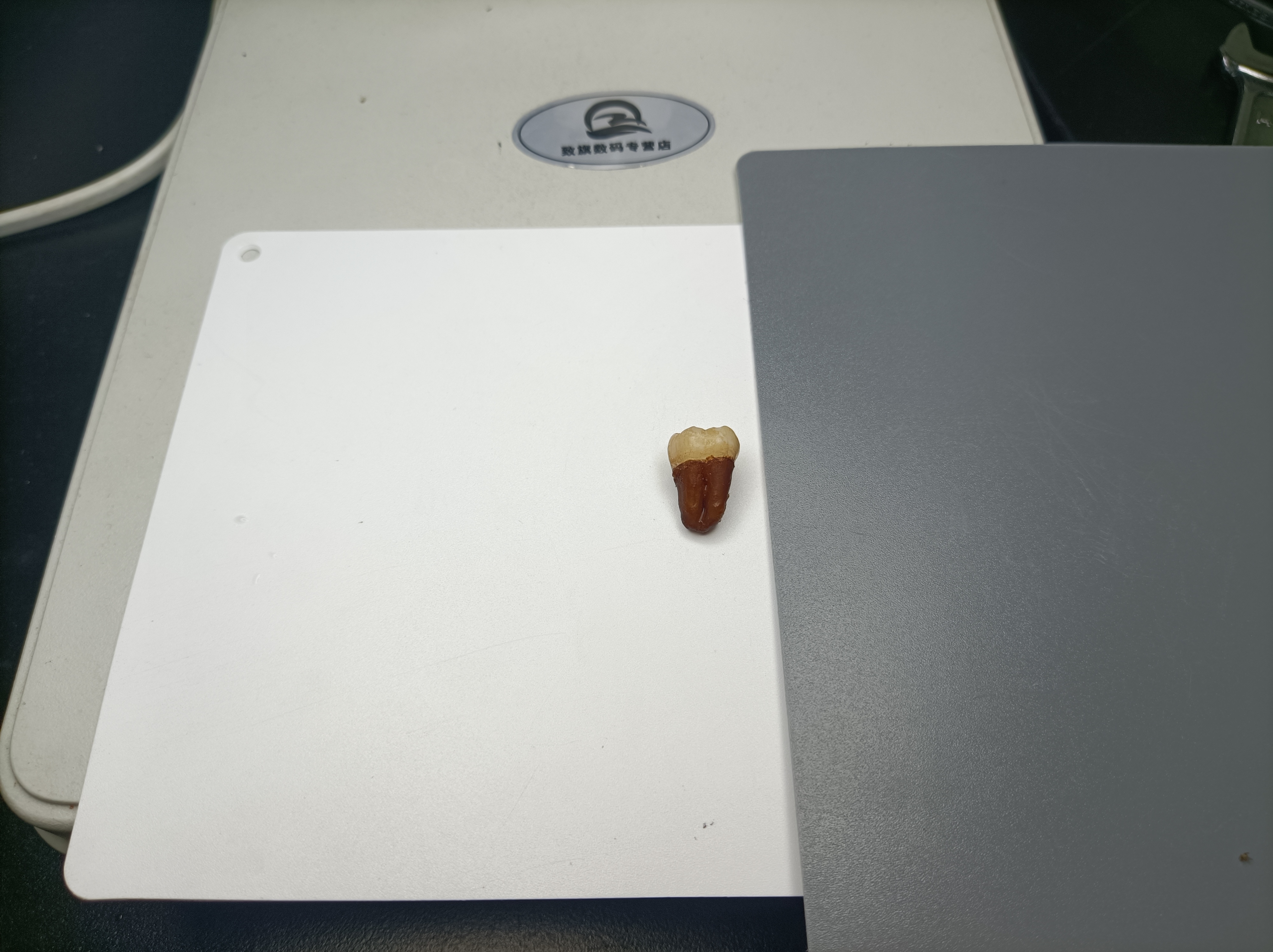

Supplement: Supplementary file 6 — Source data [file 41467_2022_32132_MOESM6_ESM.zip › Source data/main text/Figure 4/Figures/without BTO 36-61/2000.jpg]

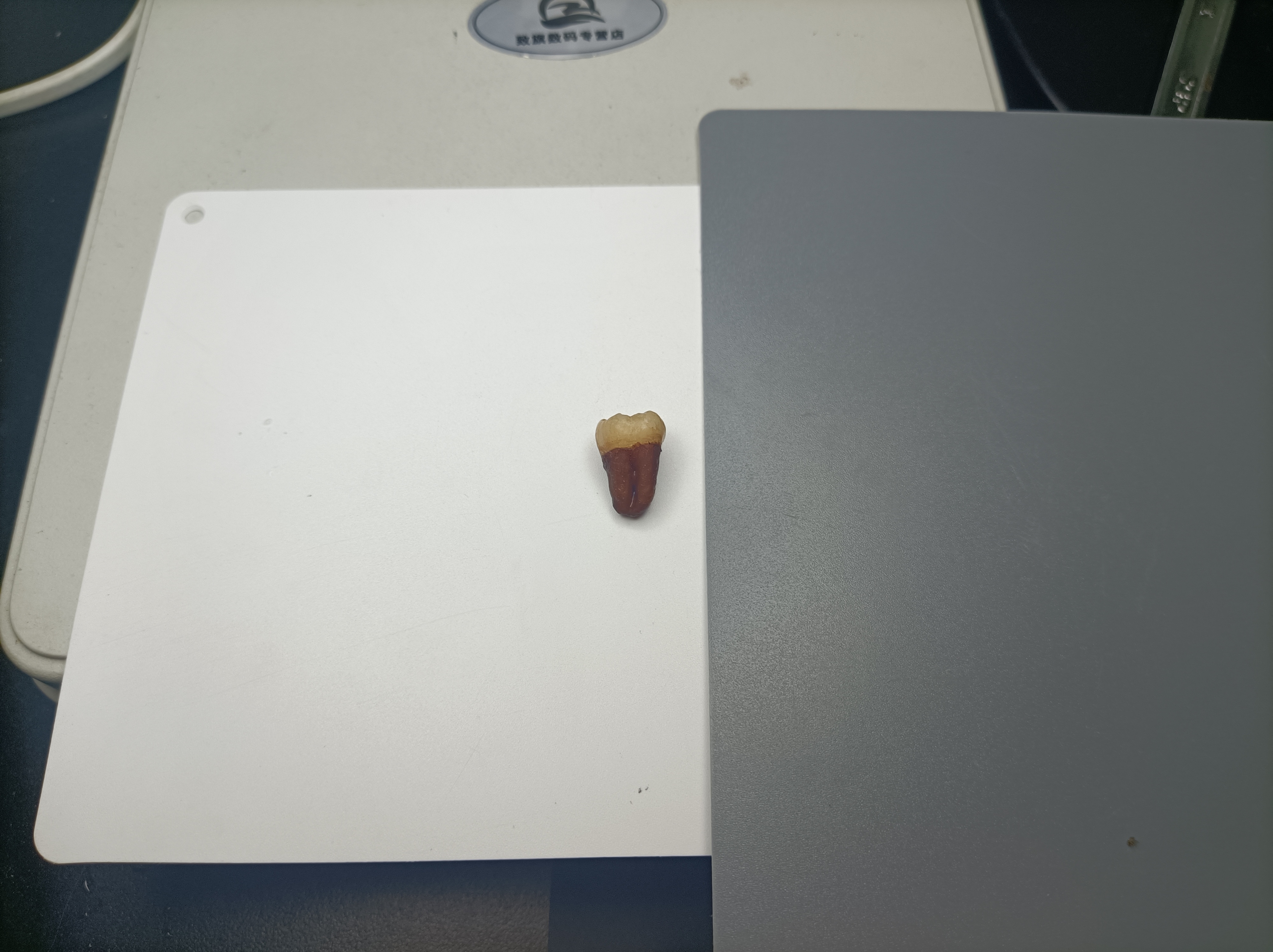

Supplement: Supplementary file 6 — Source data [file 41467_2022_32132_MOESM6_ESM.zip › Source data/main text/Figure 4/Figures/without BTO 36-61/300.jpg]

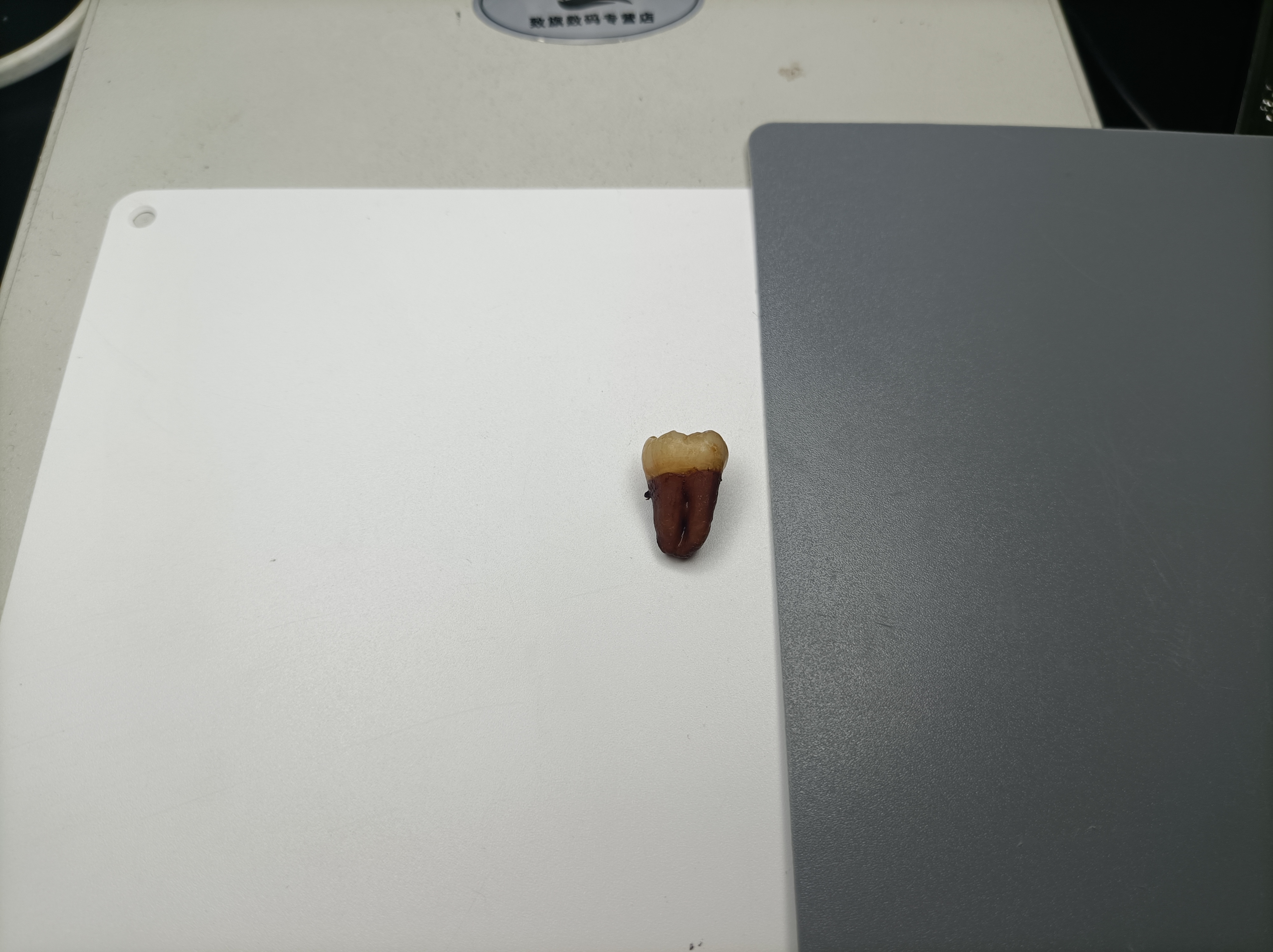

Supplement: Supplementary file 6 — Source data [file 41467_2022_32132_MOESM6_ESM.zip › Source data/main text/Figure 4/Figures/without BTO 36-61/50.jpg]

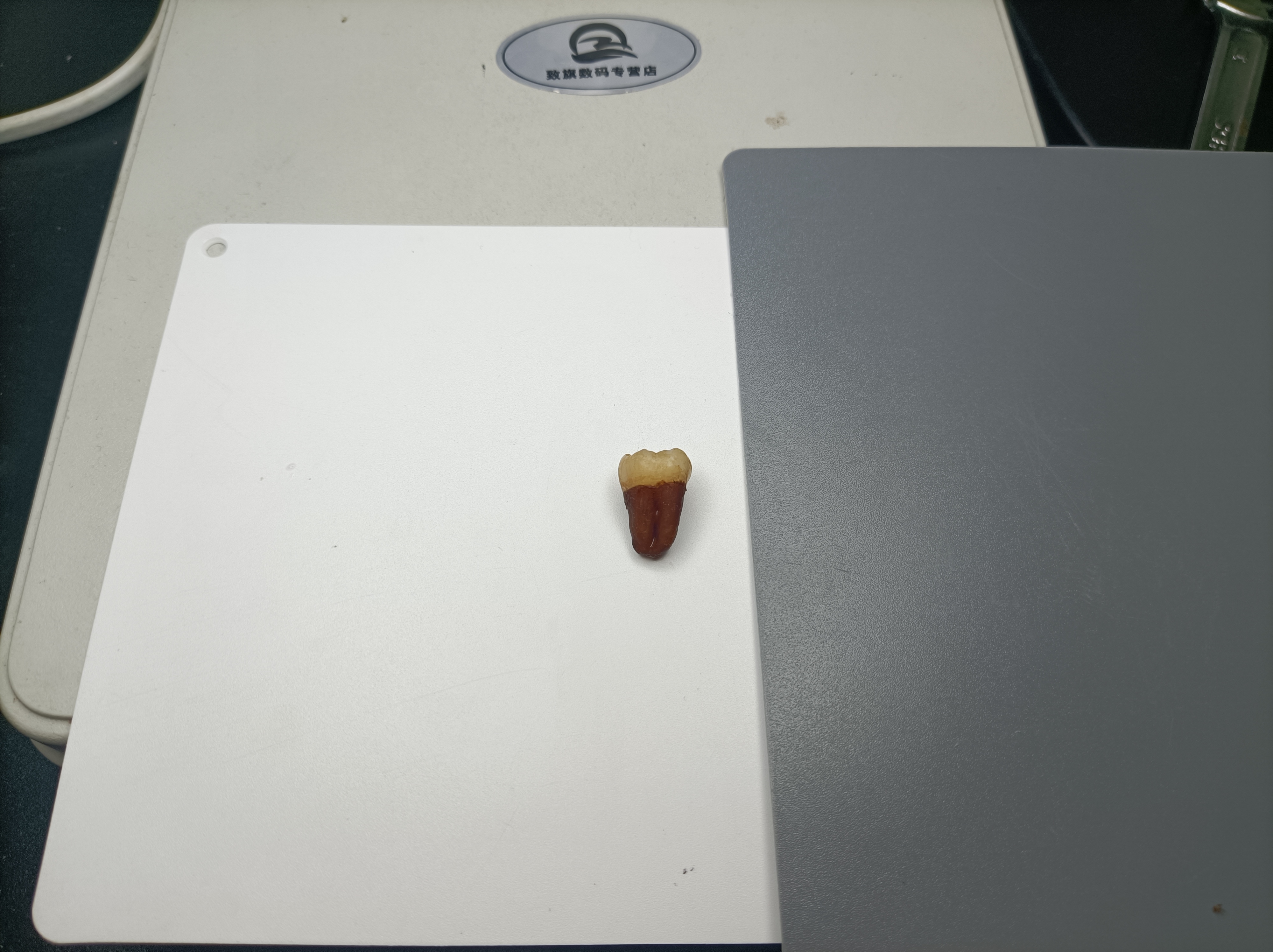

Supplement: Supplementary file 6 — Source data [file 41467_2022_32132_MOESM6_ESM.zip › Source data/main text/Figure 4/Figures/without BTO 36-61/500.jpg]

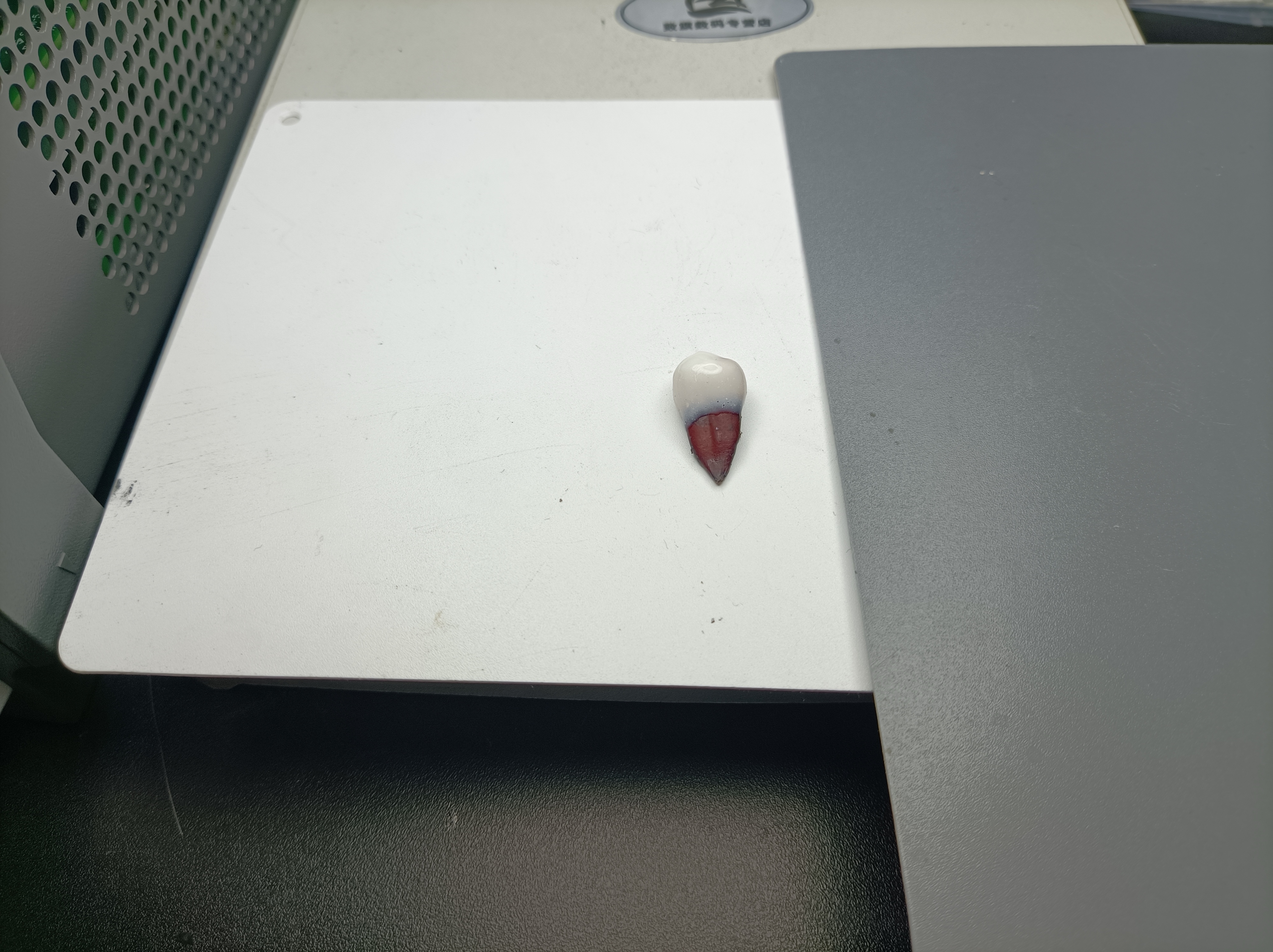

Supplement: Supplementary file 6 — Source data [file 41467_2022_32132_MOESM6_ESM.zip › Source data/main text/Figure 5/Figures/fast/BTO-gel.jpg]

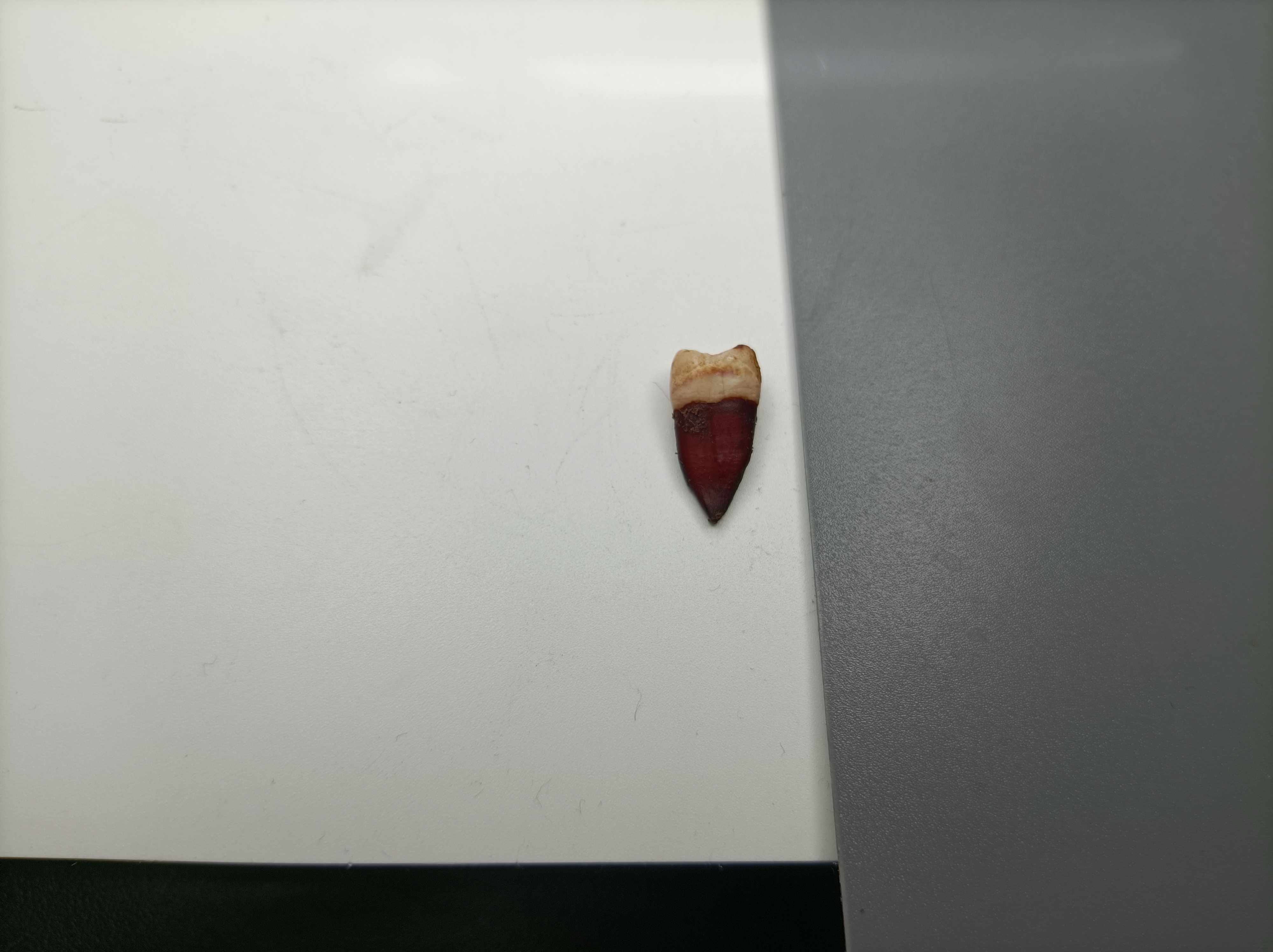

Supplement: Supplementary file 6 — Source data [file 41467_2022_32132_MOESM6_ESM.zip › Source data/main text/Figure 5/Figures/fast/origin.jpg]

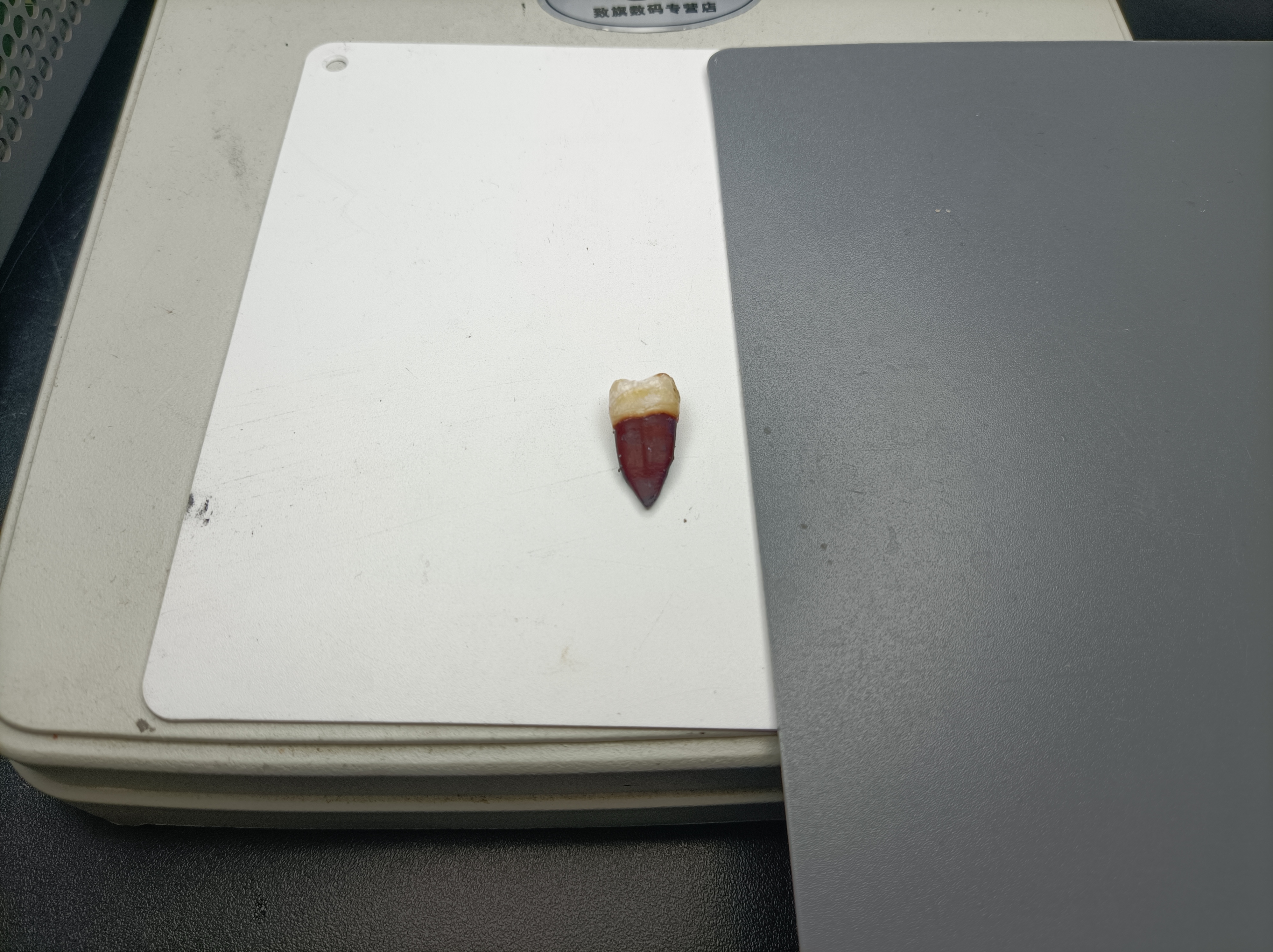

Supplement: Supplementary file 6 — Source data [file 41467_2022_32132_MOESM6_ESM.zip › Source data/main text/Figure 5/Figures/fast/whitened.jpg]

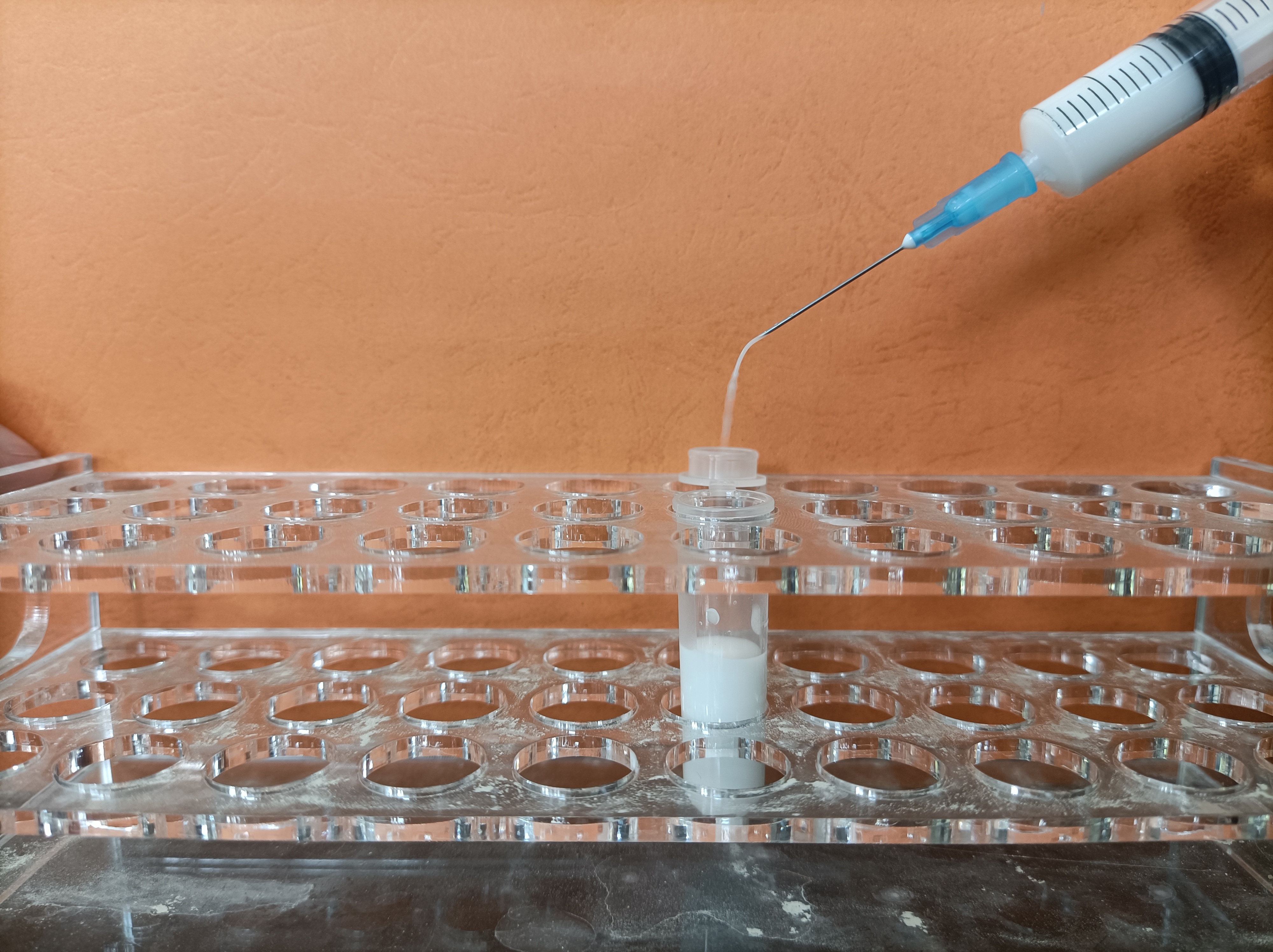

Supplement: Supplementary file 6 — Source data [file 41467_2022_32132_MOESM6_ESM.zip › Source data/main text/Figure 5/Figures/Gel.jpg]

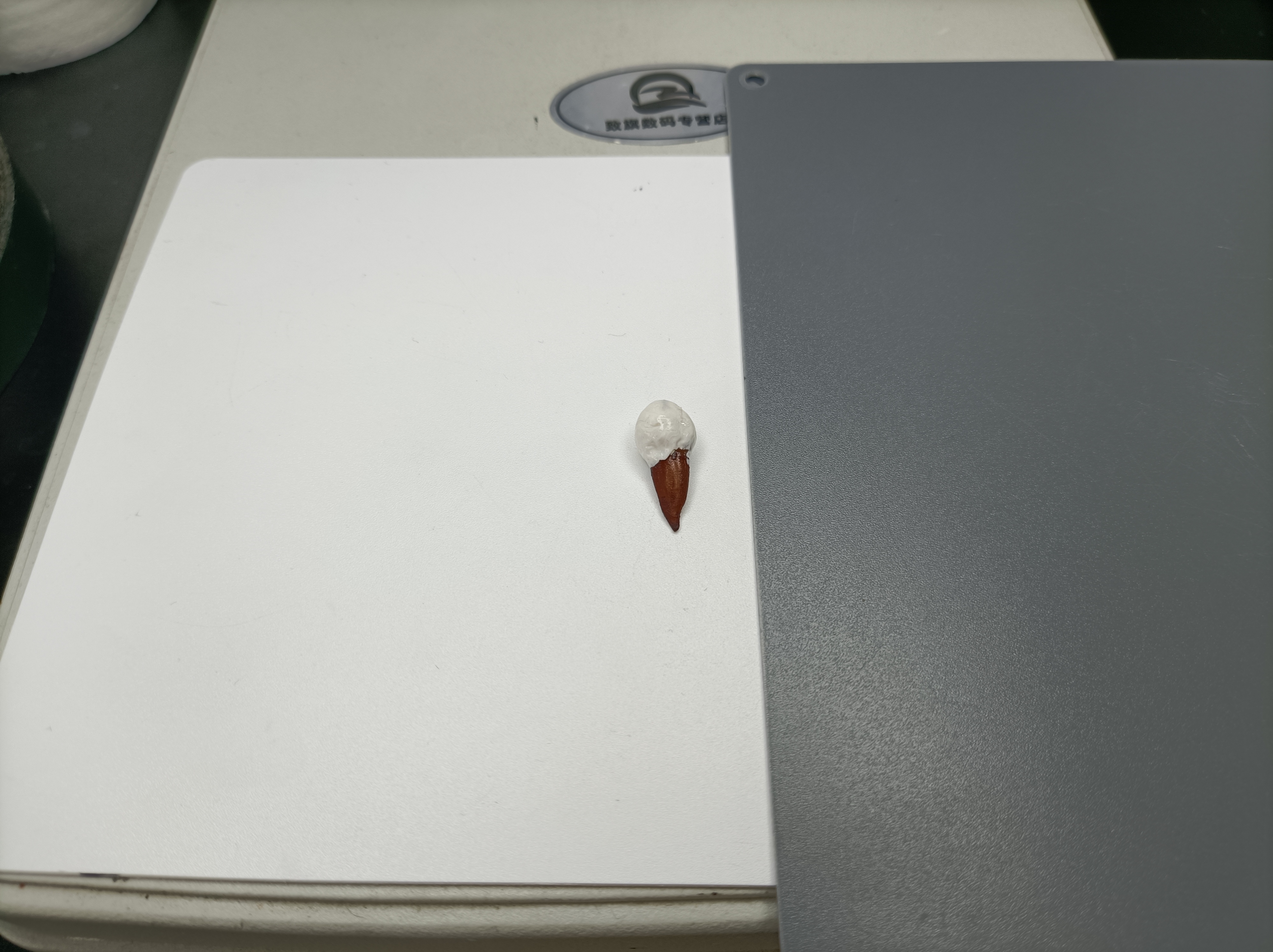

Supplement: Supplementary file 6 — Source data [file 41467_2022_32132_MOESM6_ESM.zip › Source data/main text/Figure 5/Figures/slow/BTO-gel.jpg]

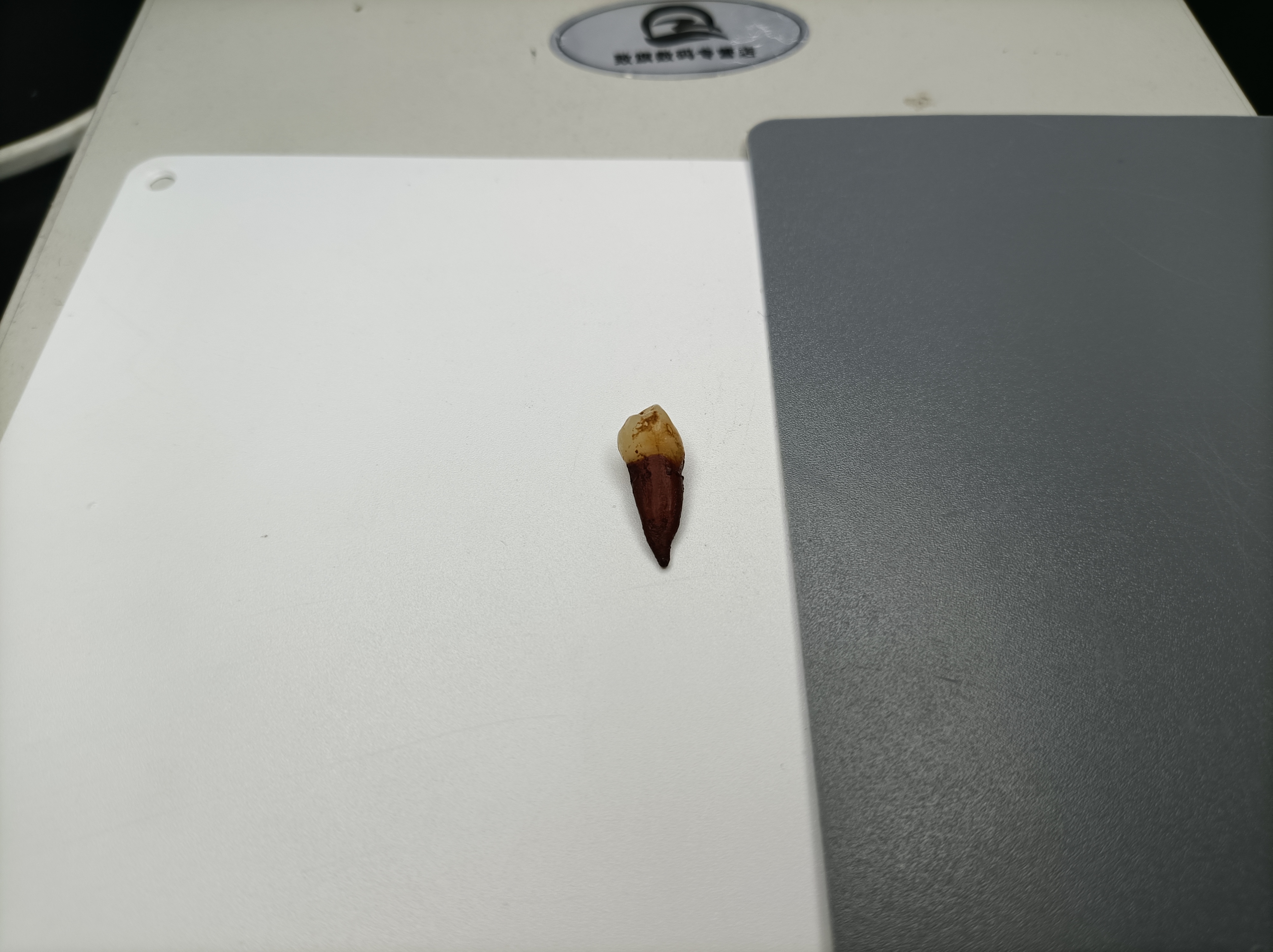

Supplement: Supplementary file 6 — Source data [file 41467_2022_32132_MOESM6_ESM.zip › Source data/main text/Figure 5/Figures/slow/origin.jpg]

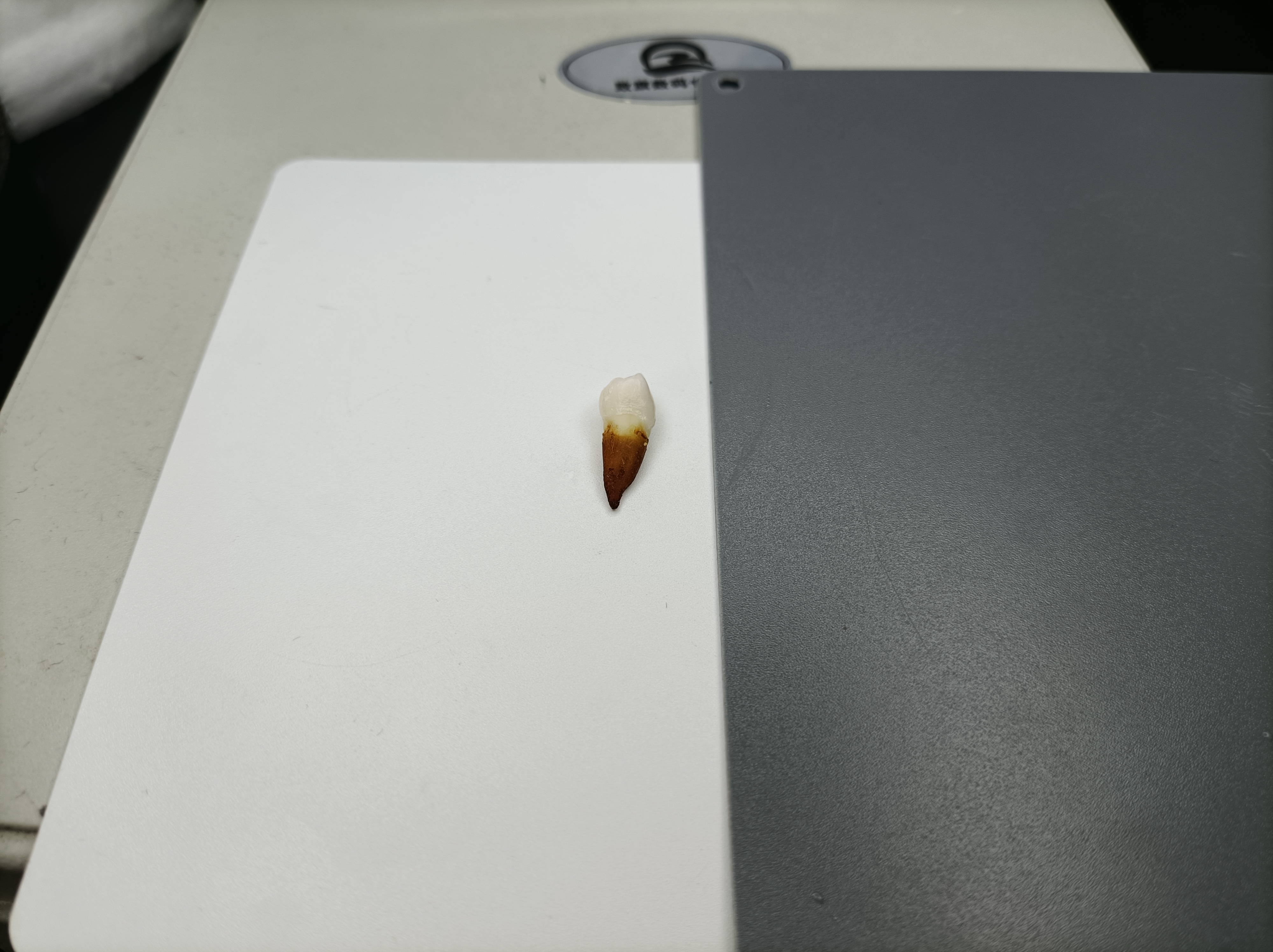

Supplement: Supplementary file 6 — Source data [file 41467_2022_32132_MOESM6_ESM.zip › Source data/main text/Figure 5/Figures/slow/whitened.jpg]

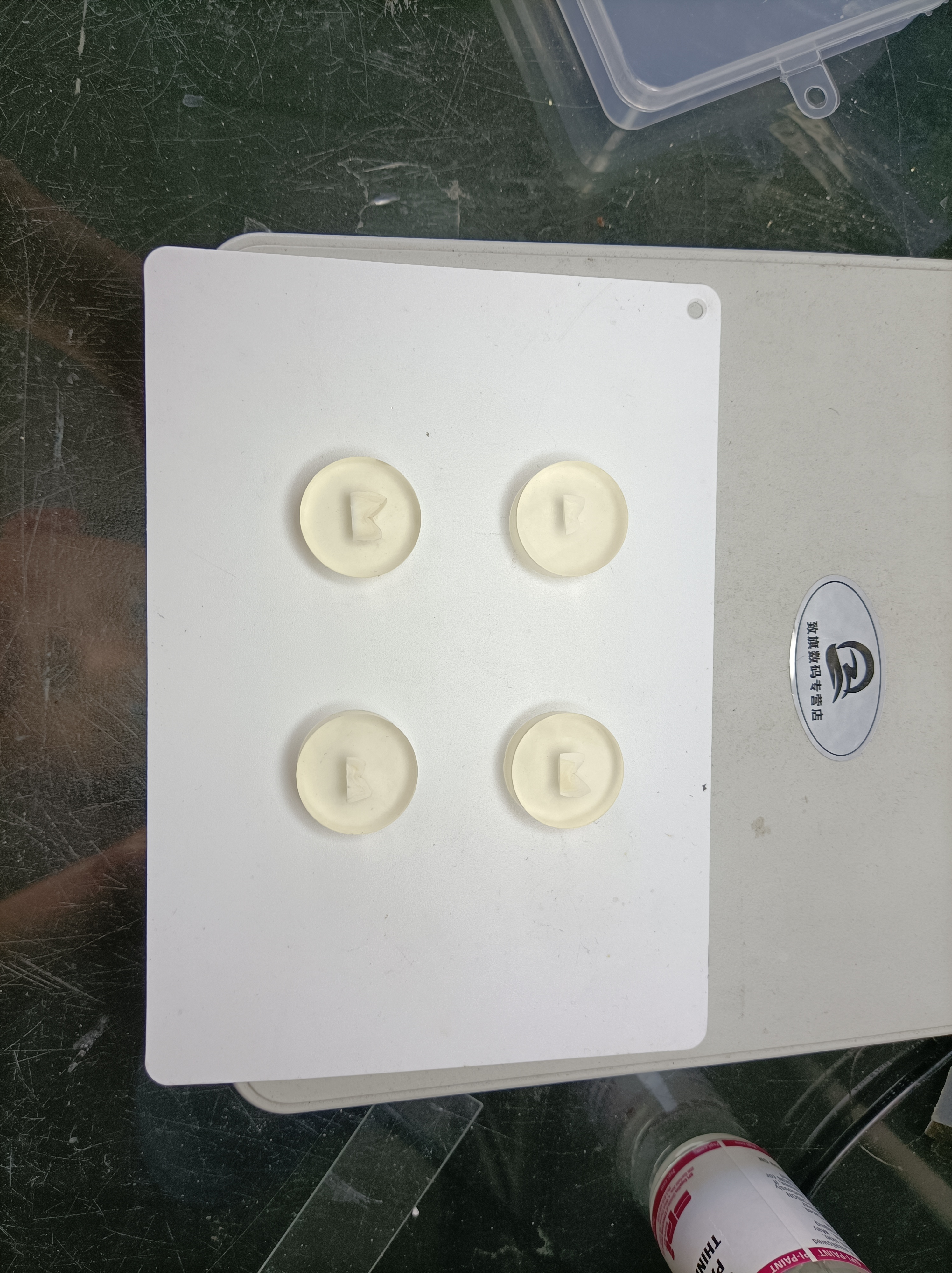

Supplement: Supplementary file 6 — Source data [file 41467_2022_32132_MOESM6_ESM.zip › Source data/main text/Figure 6/Figures/hardness/origin.jpg]

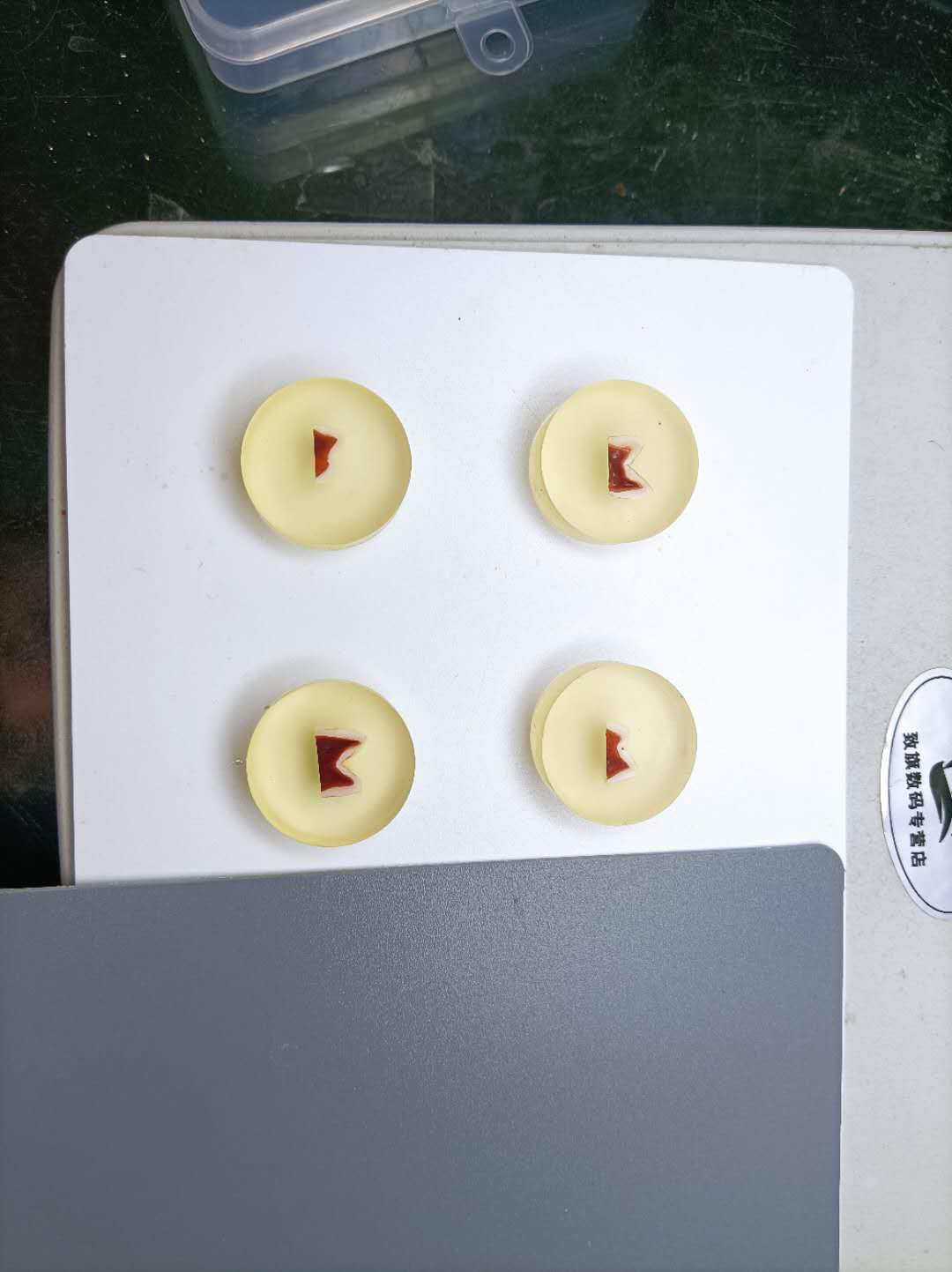

Supplement: Supplementary file 6 — Source data [file 41467_2022_32132_MOESM6_ESM.zip › Source data/main text/Figure 6/Figures/hardness/stained.jpg]

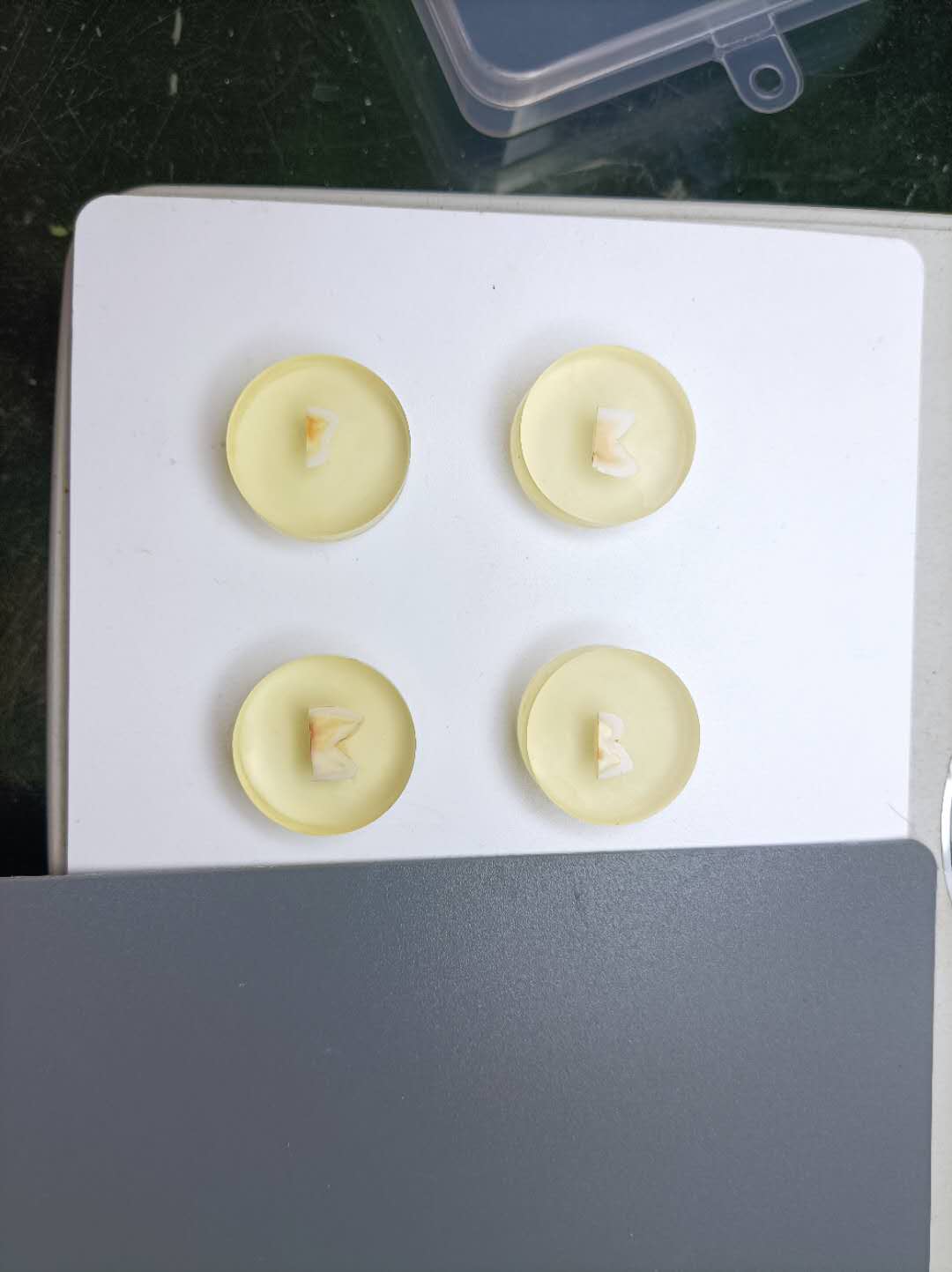

Supplement: Supplementary file 6 — Source data [file 41467_2022_32132_MOESM6_ESM.zip › Source data/main text/Figure 6/Figures/hardness/whitened.jpg]

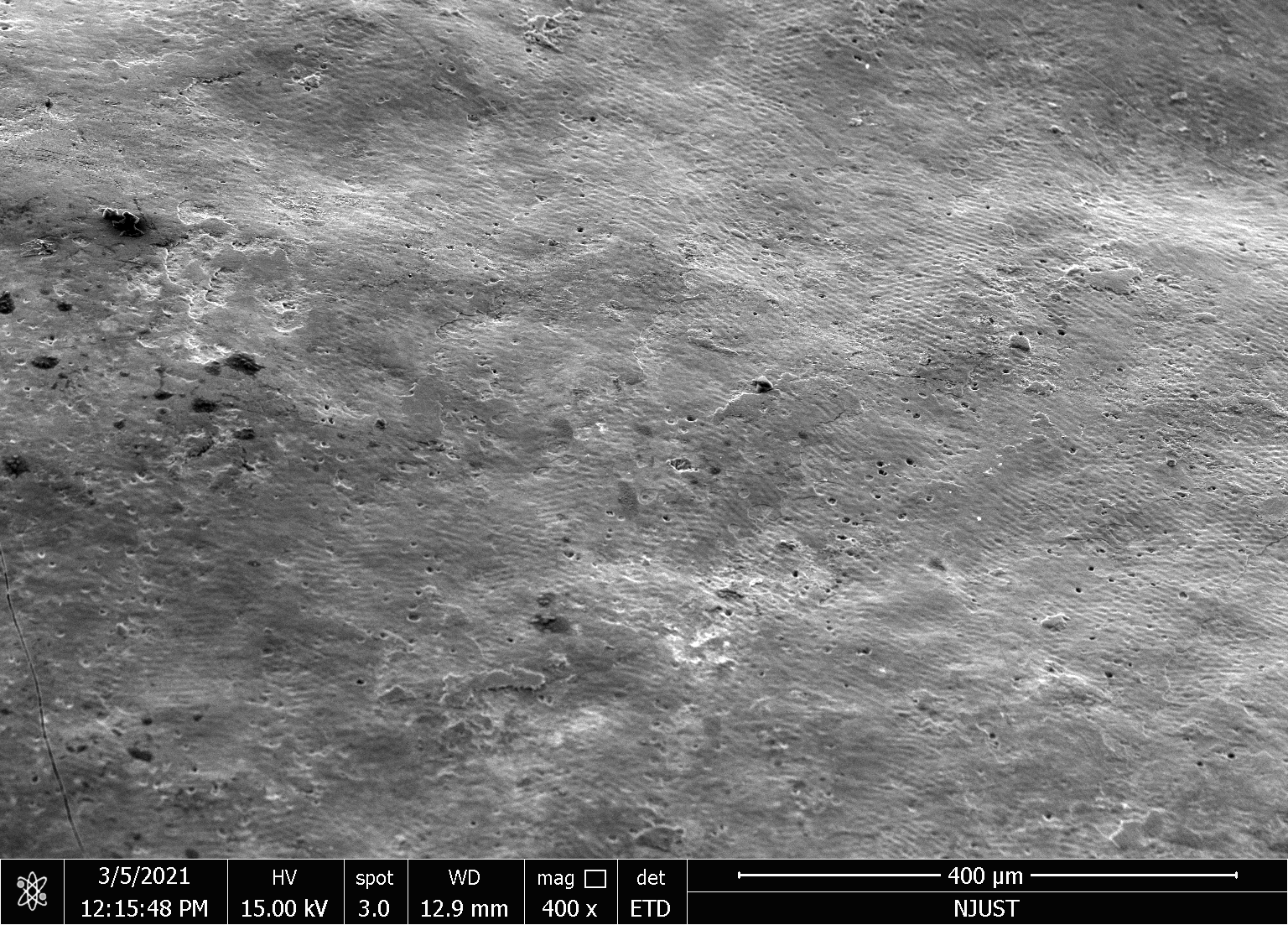

Supplement: Supplementary file 6 — Source data [file 41467_2022_32132_MOESM6_ESM.zip › Source data/main text/Figure 6/Figures/SEM/origin/tooth.tif]

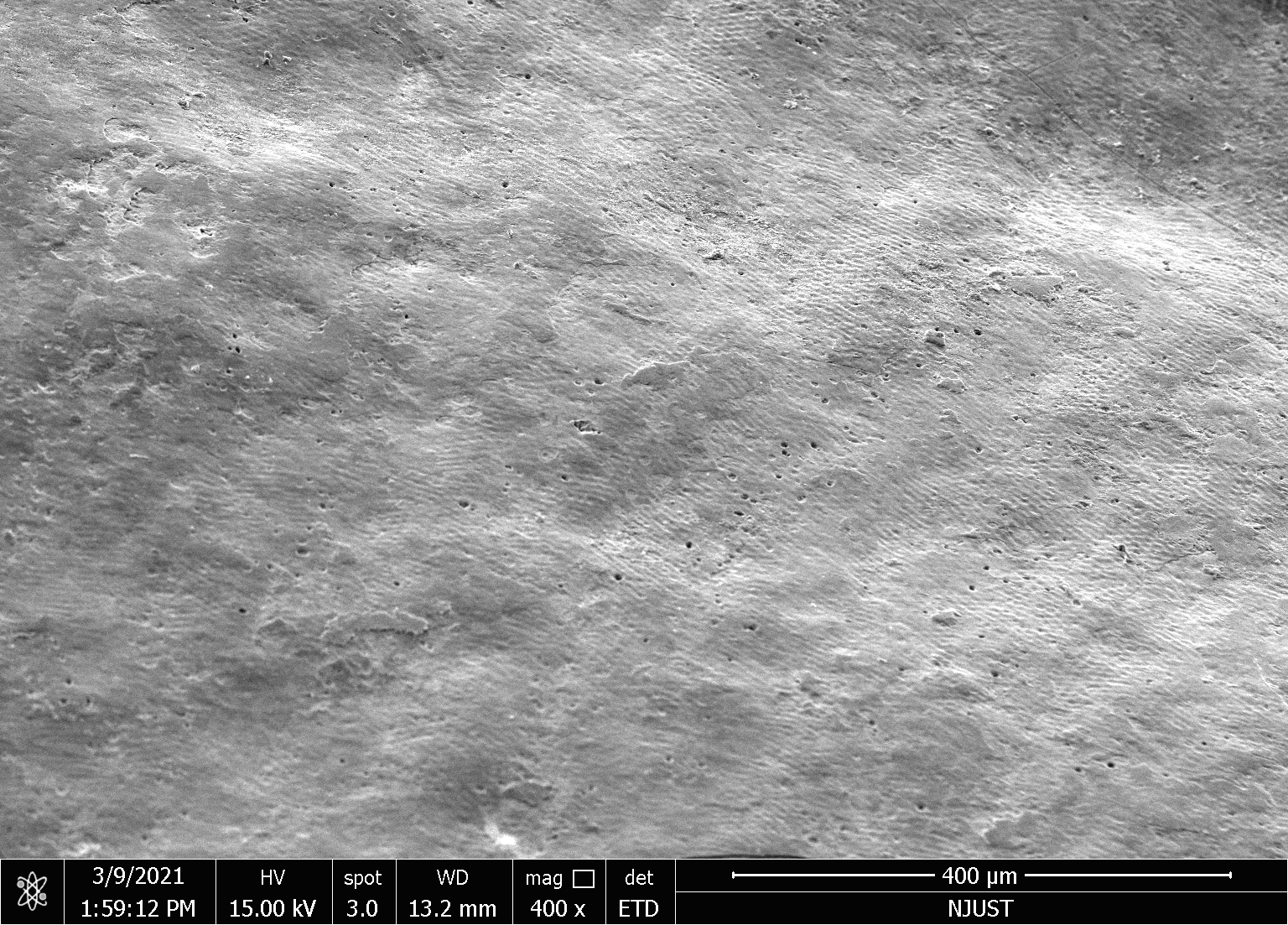

Supplement: Supplementary file 6 — Source data [file 41467_2022_32132_MOESM6_ESM.zip › Source data/main text/Figure 6/Figures/SEM/pyro-particles/tooth.tif]

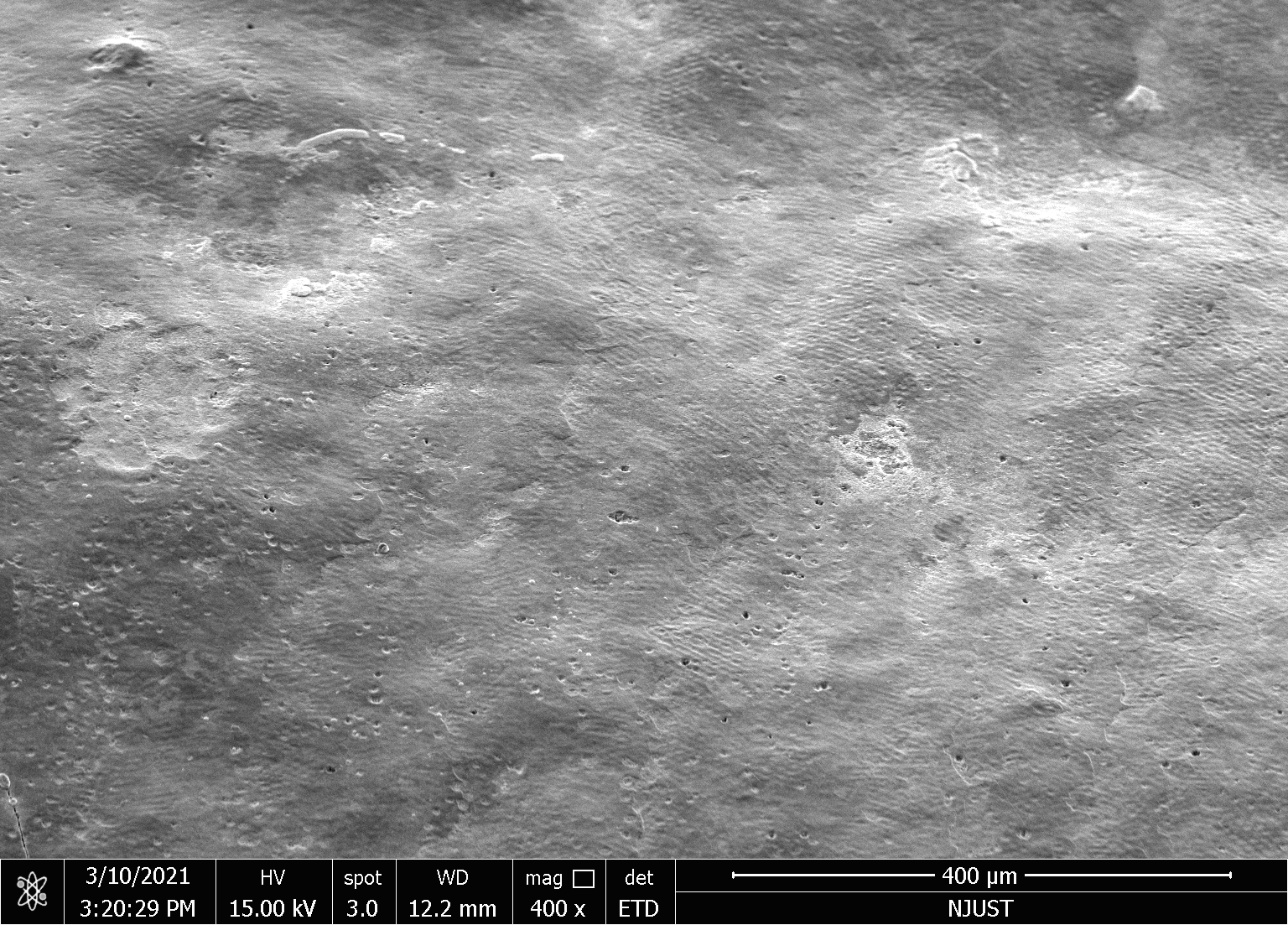

Supplement: Supplementary file 6 — Source data [file 41467_2022_32132_MOESM6_ESM.zip › Source data/main text/Figure 6/Figures/SEM/whitening agent/tooth.tif]

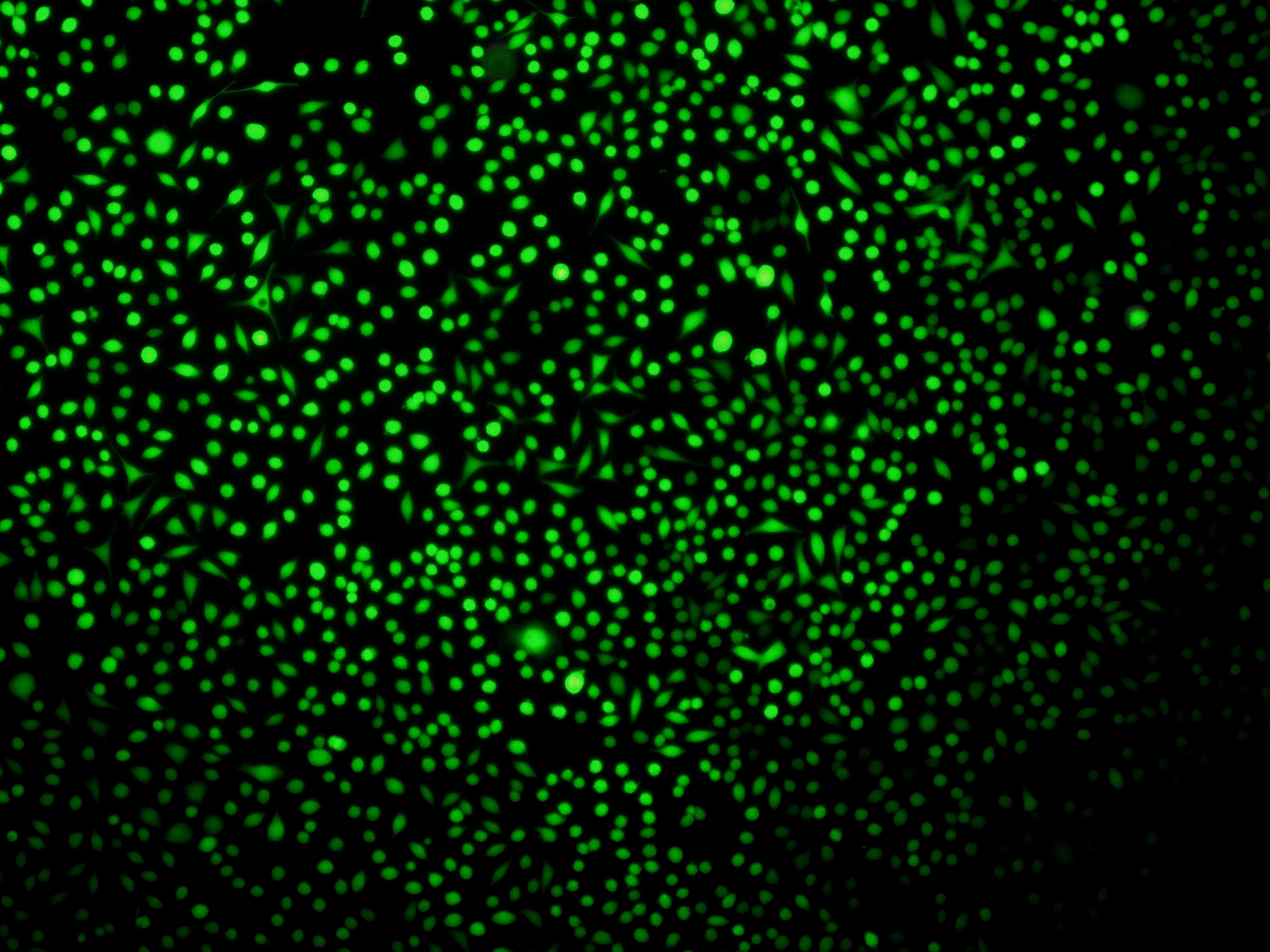

Supplement: Supplementary file 6 — Source data [file 41467_2022_32132_MOESM6_ESM.zip › Source data/main text/Figure 7/Figures/day1/0.1.tif]

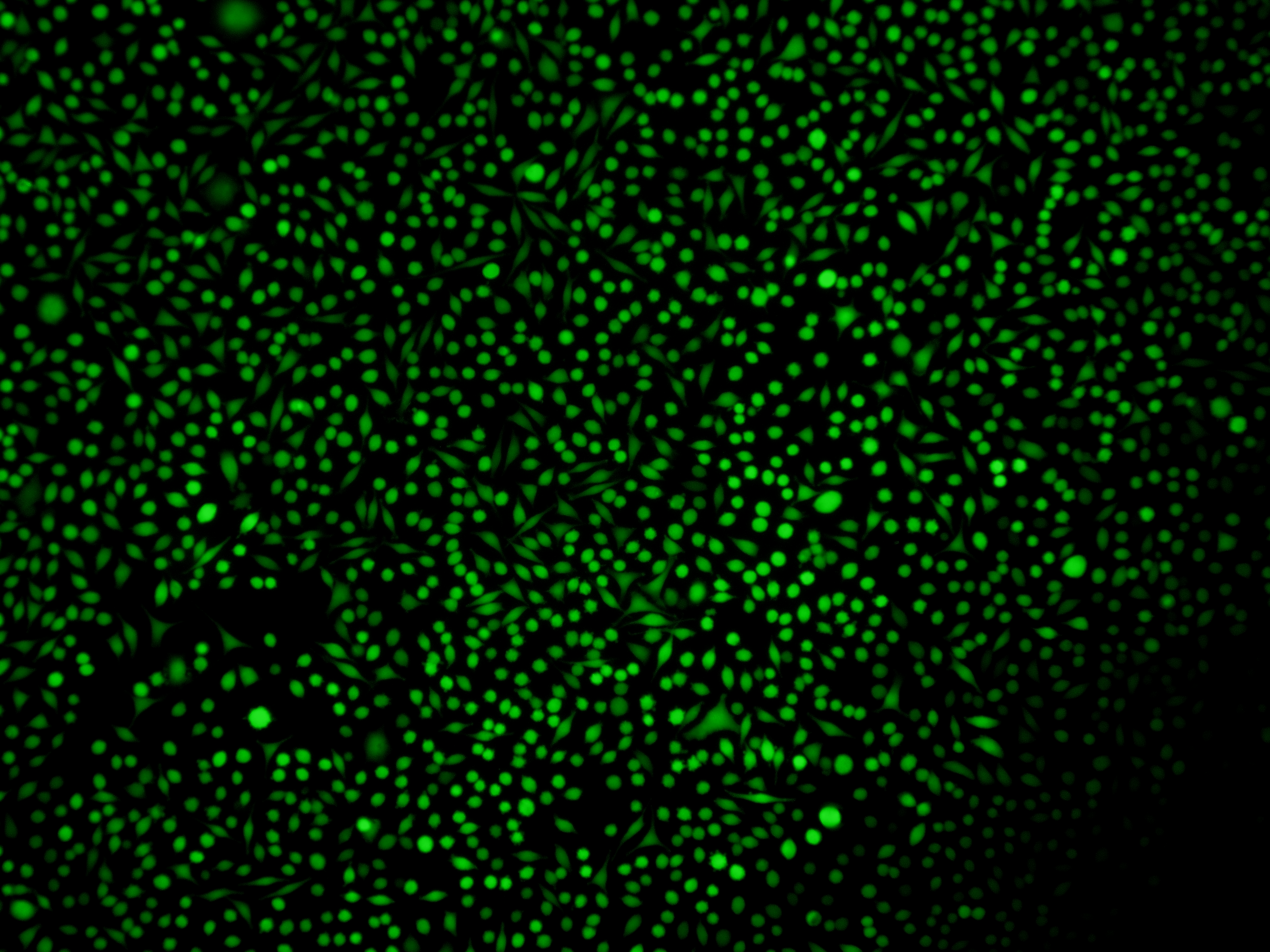

Supplement: Supplementary file 6 — Source data [file 41467_2022_32132_MOESM6_ESM.zip › Source data/main text/Figure 7/Figures/day1/0.2.tif]

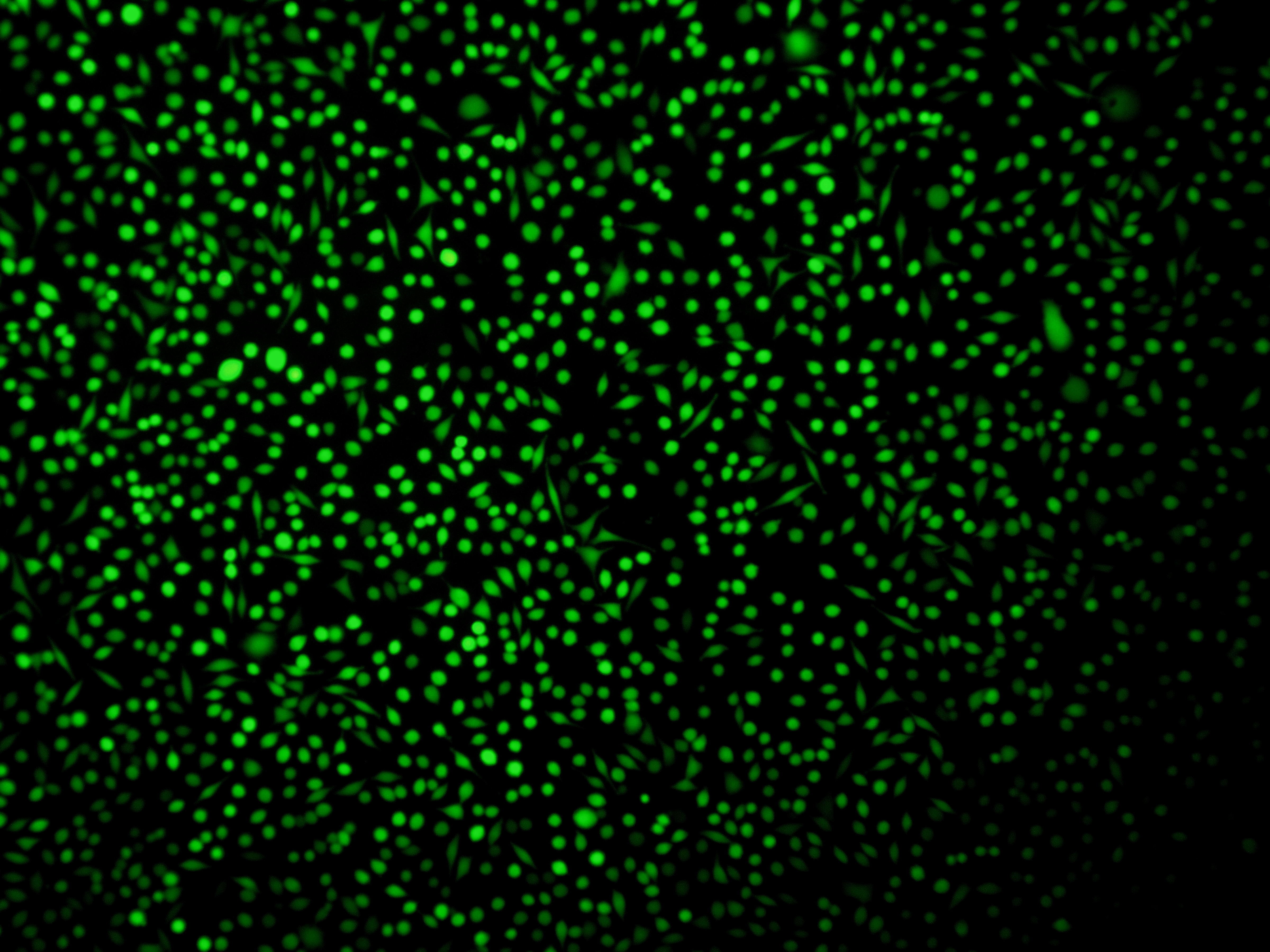

Supplement: Supplementary file 6 — Source data [file 41467_2022_32132_MOESM6_ESM.zip › Source data/main text/Figure 7/Figures/day1/0.3.tif]

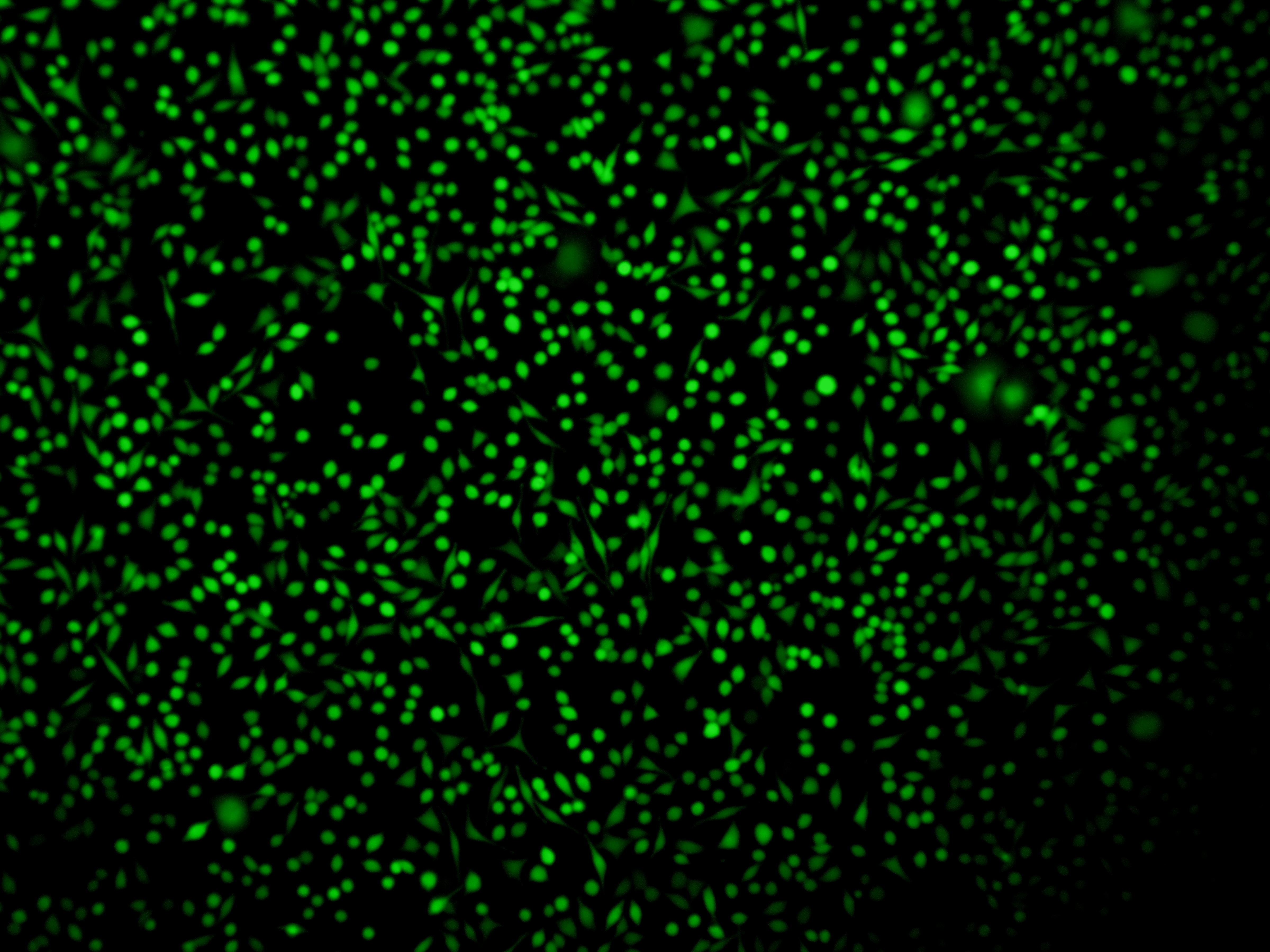

Supplement: Supplementary file 6 — Source data [file 41467_2022_32132_MOESM6_ESM.zip › Source data/main text/Figure 7/Figures/day1/control.tif]

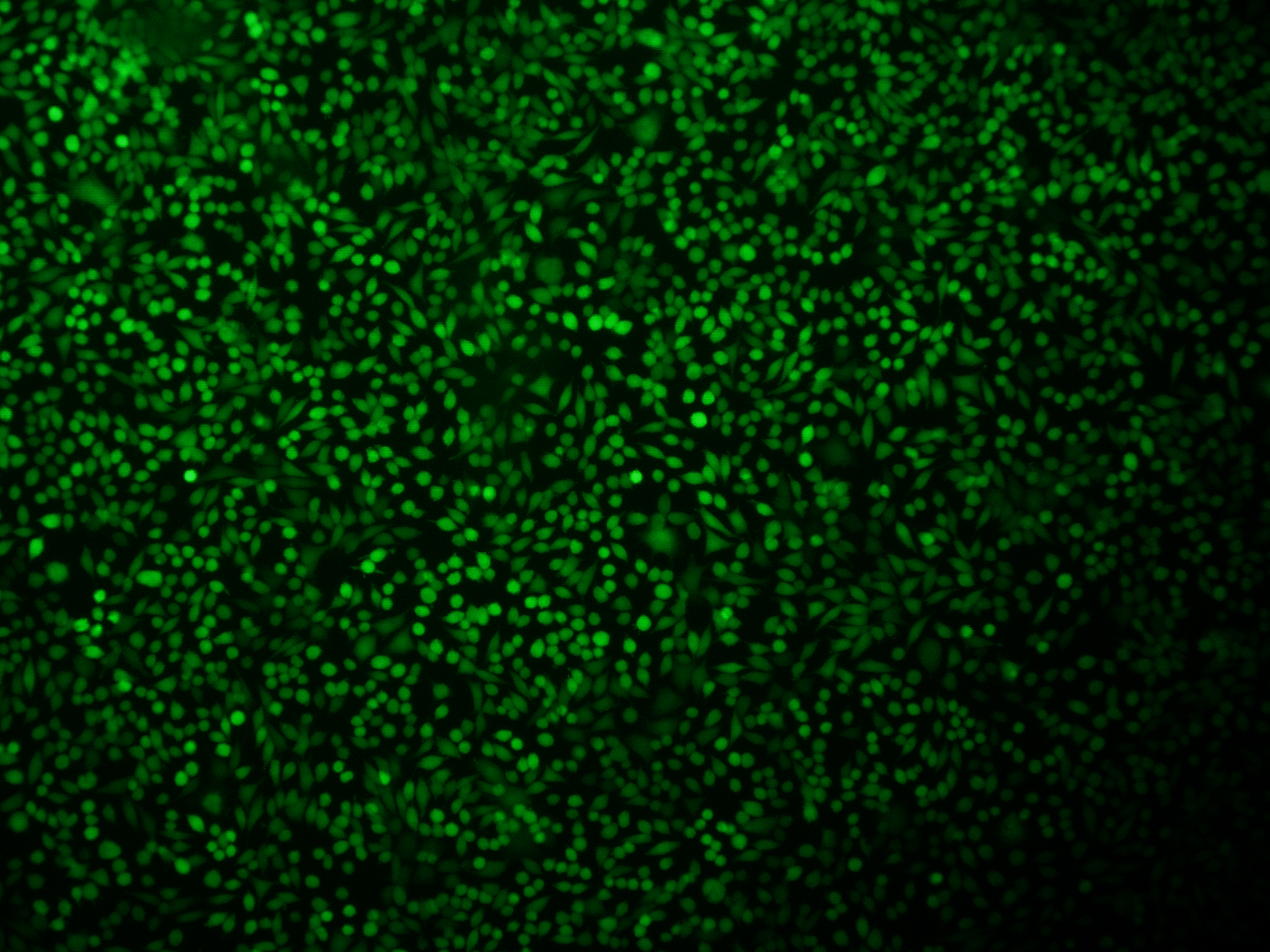

Supplement: Supplementary file 6 — Source data [file 41467_2022_32132_MOESM6_ESM.zip › Source data/main text/Figure 7/Figures/day2/0.1.tif]

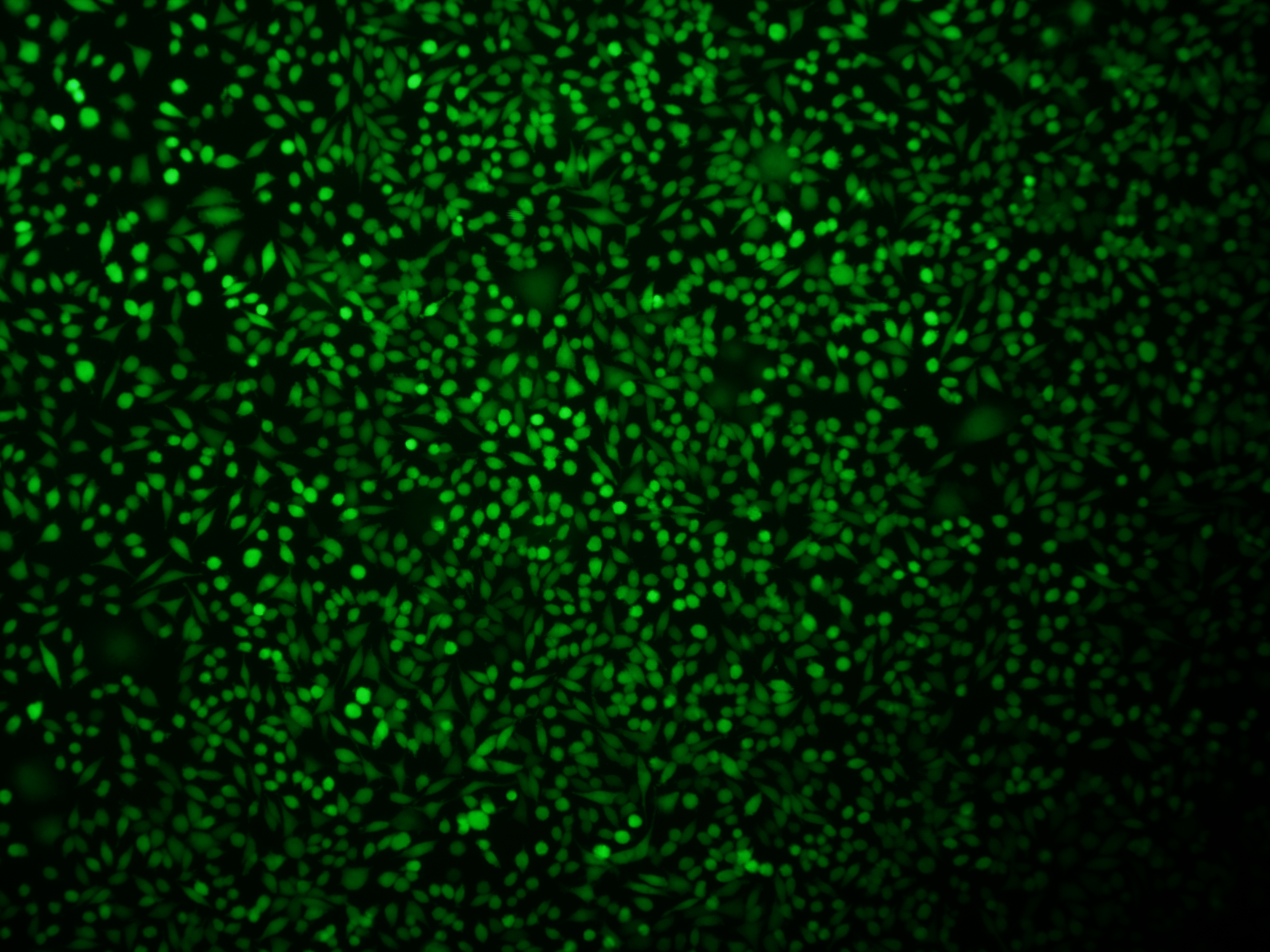

Supplement: Supplementary file 6 — Source data [file 41467_2022_32132_MOESM6_ESM.zip › Source data/main text/Figure 7/Figures/day2/0.2.tif]

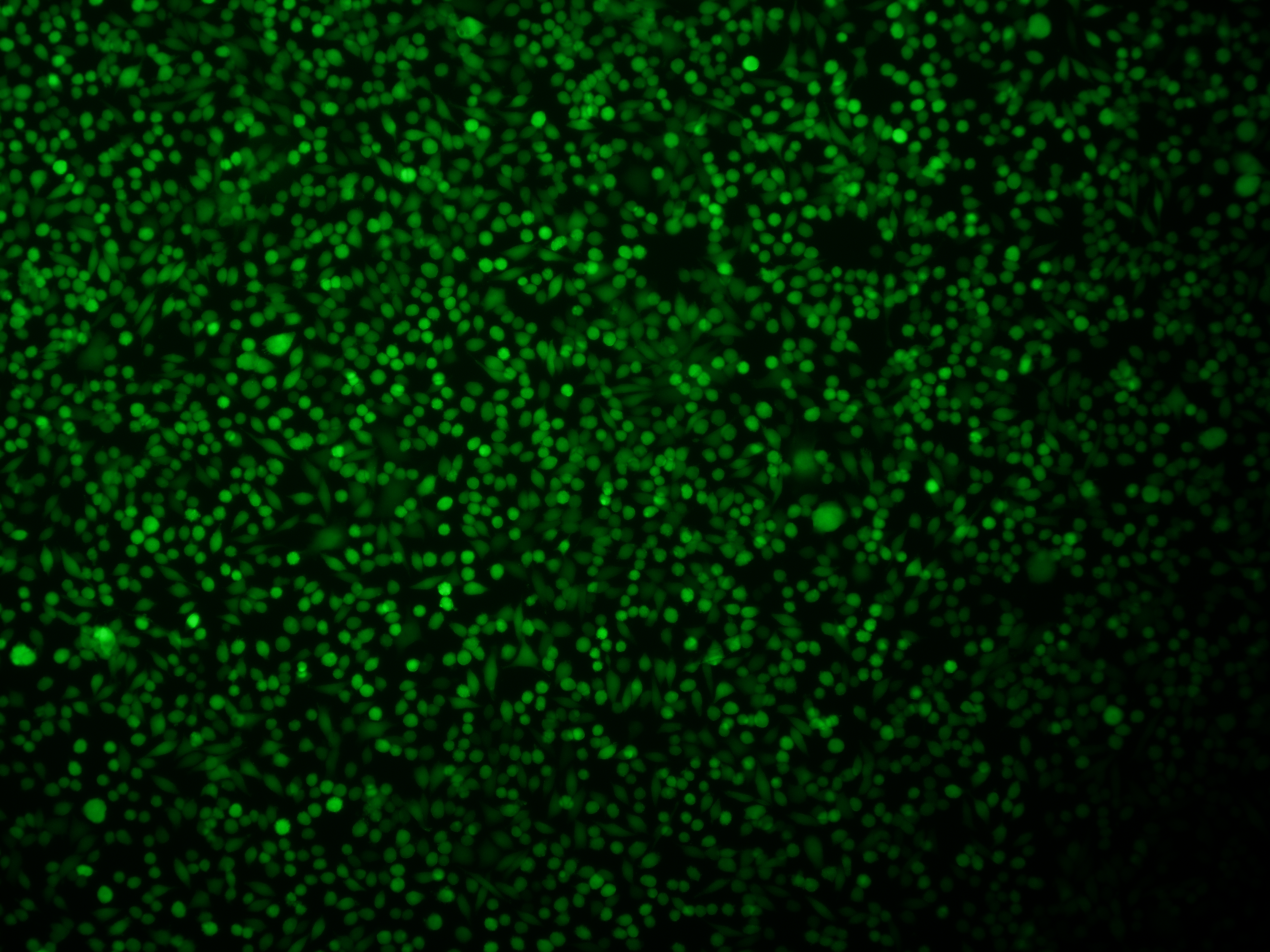

Supplement: Supplementary file 6 — Source data [file 41467_2022_32132_MOESM6_ESM.zip › Source data/main text/Figure 7/Figures/day2/0.3.tif]

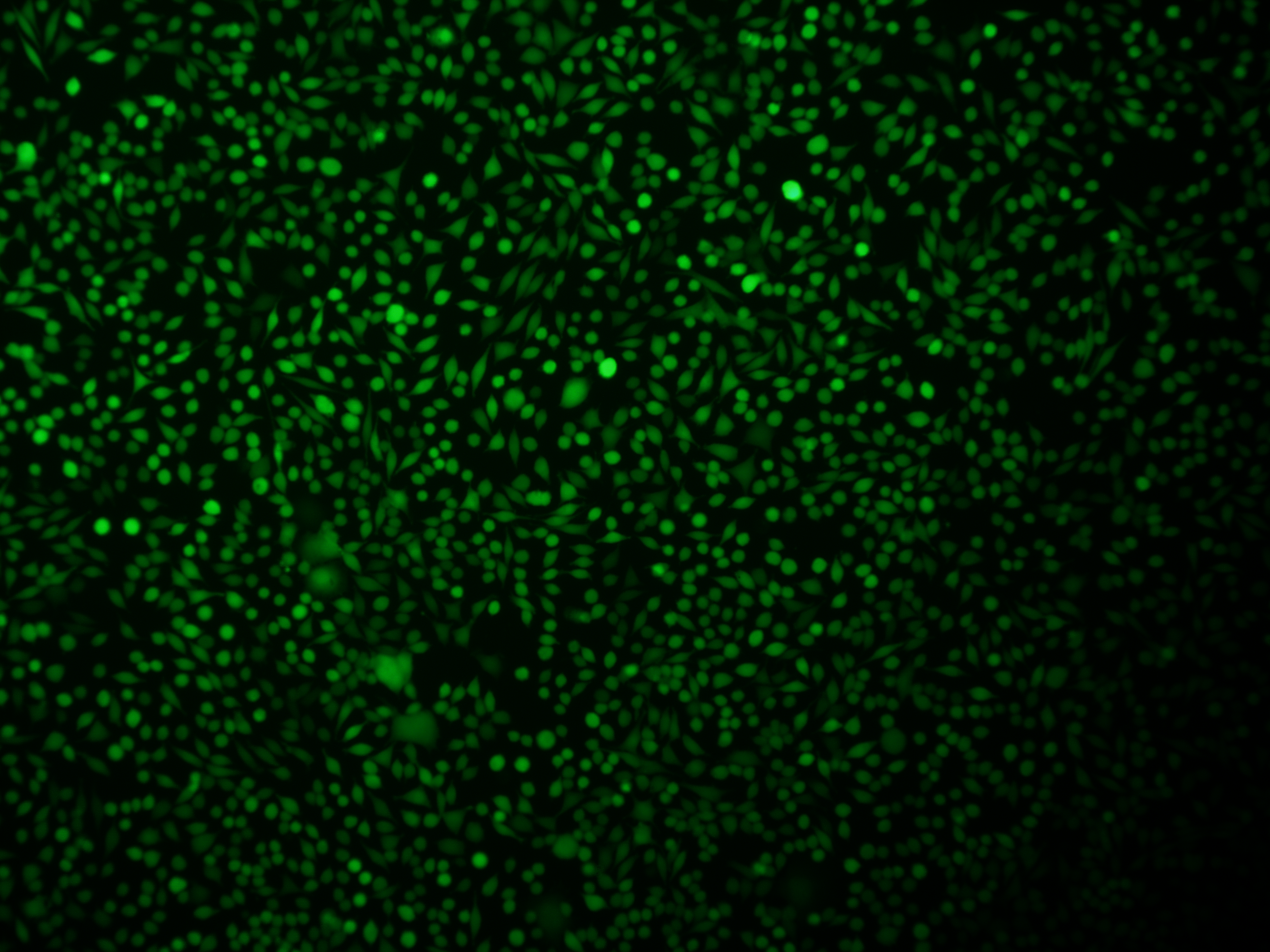

Supplement: Supplementary file 6 — Source data [file 41467_2022_32132_MOESM6_ESM.zip › Source data/main text/Figure 7/Figures/day2/control.tif]

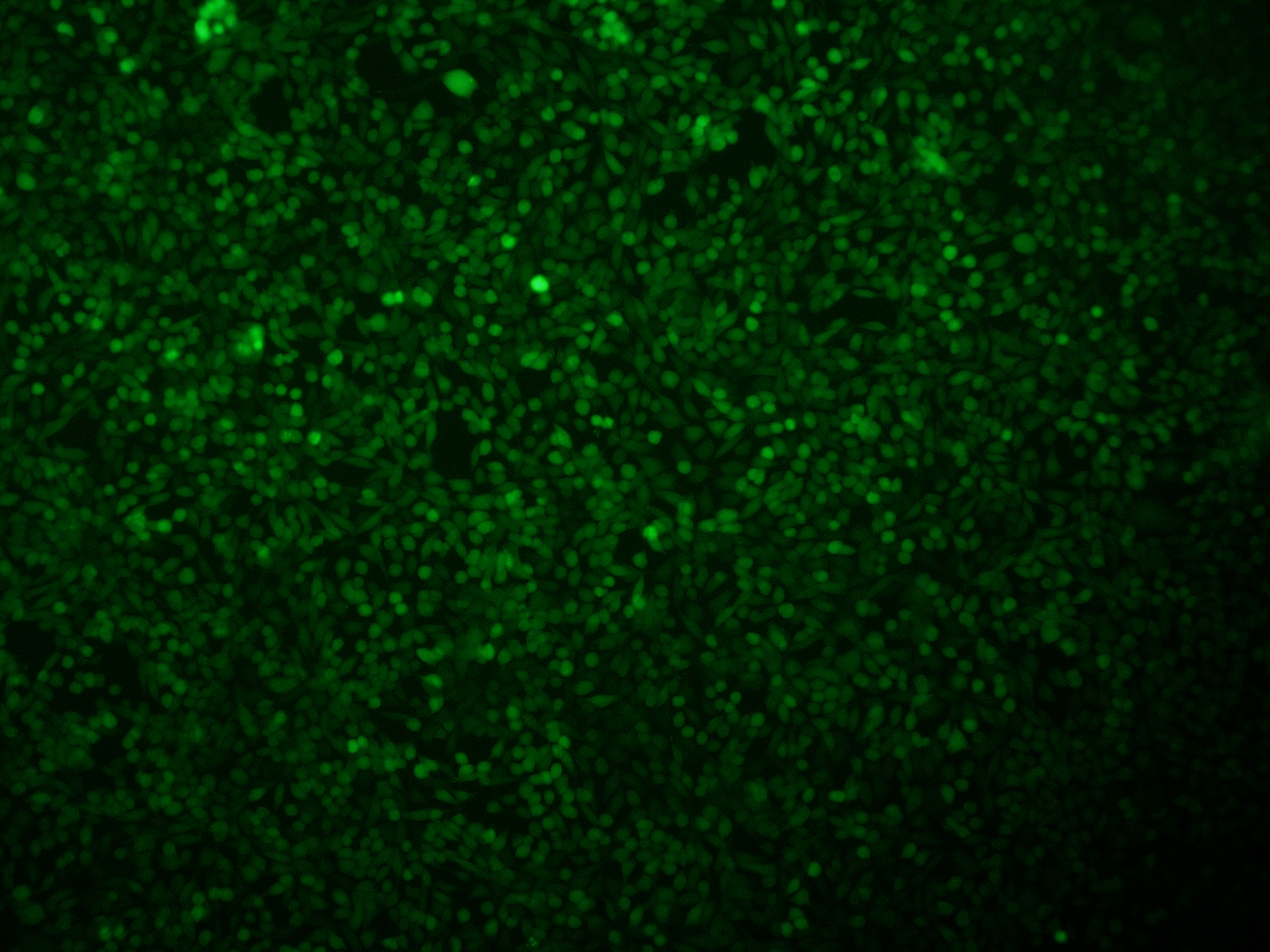

Supplement: Supplementary file 6 — Source data [file 41467_2022_32132_MOESM6_ESM.zip › Source data/main text/Figure 7/Figures/day3/0.1.tif]

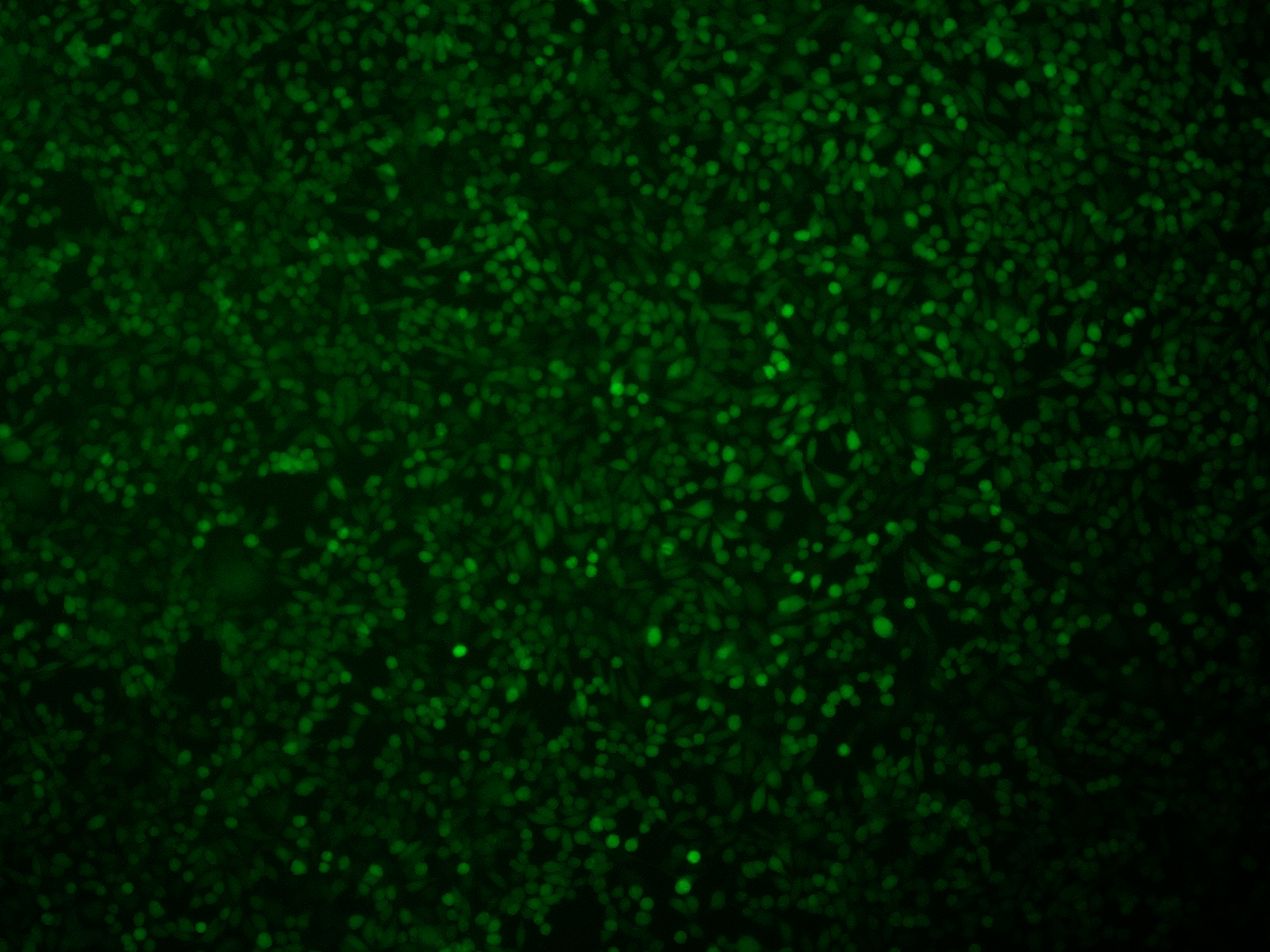

Supplement: Supplementary file 6 — Source data [file 41467_2022_32132_MOESM6_ESM.zip › Source data/main text/Figure 7/Figures/day3/0.2.tif]

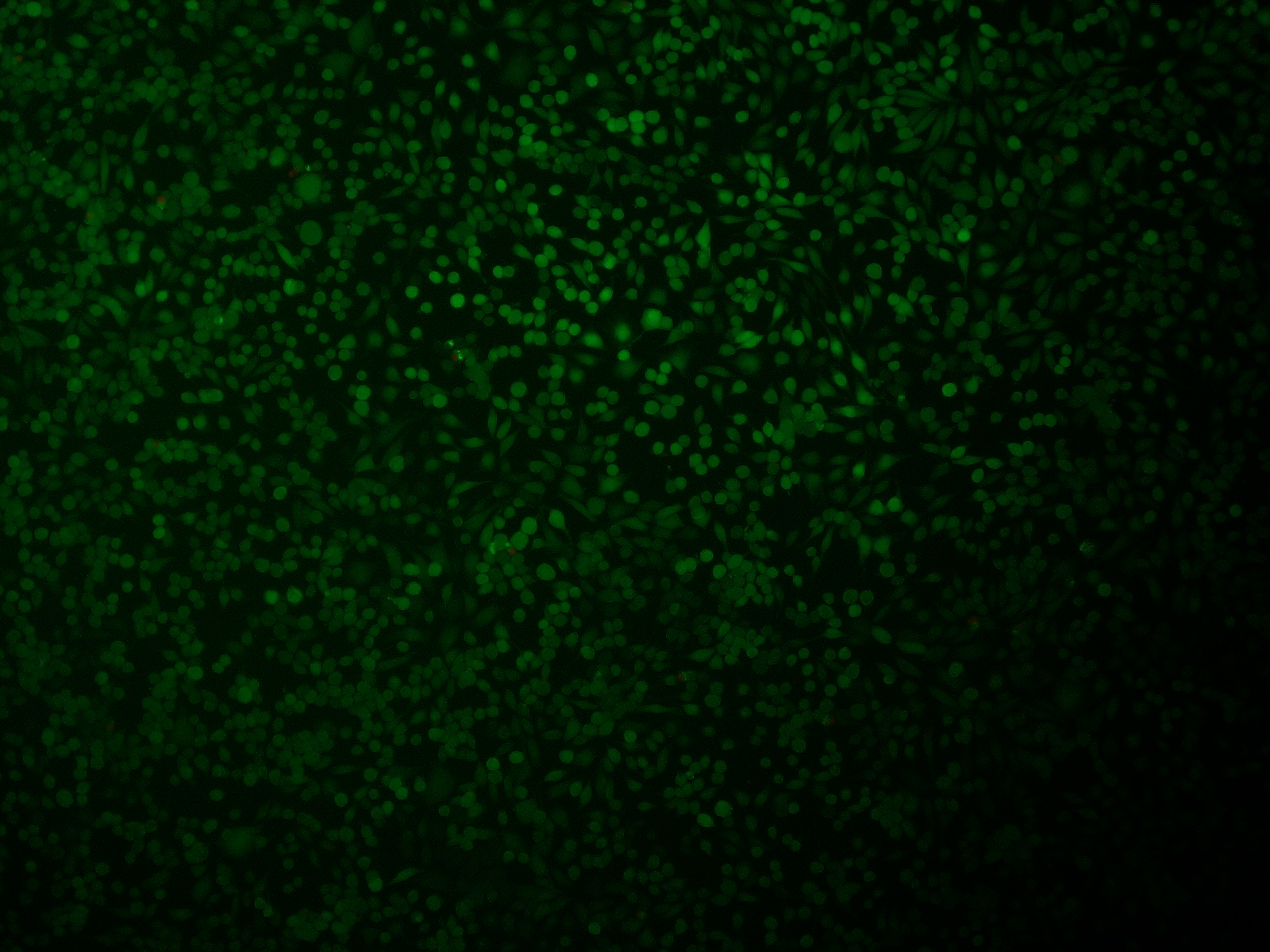

Supplement: Supplementary file 6 — Source data [file 41467_2022_32132_MOESM6_ESM.zip › Source data/main text/Figure 7/Figures/day3/0.3.tif]

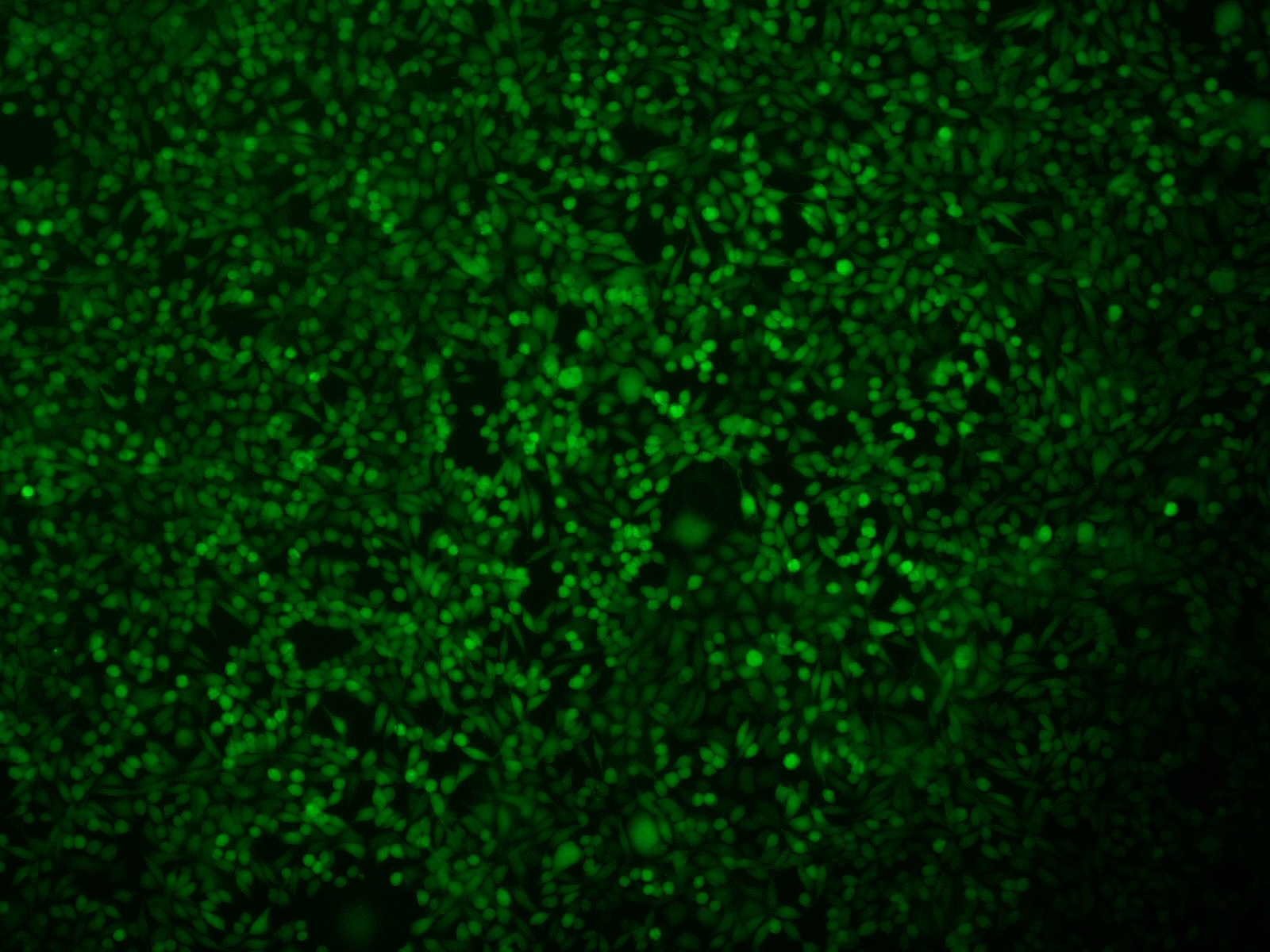

Supplement: Supplementary file 6 — Source data [file 41467_2022_32132_MOESM6_ESM.zip › Source data/main text/Figure 7/Figures/day3/control.tif]

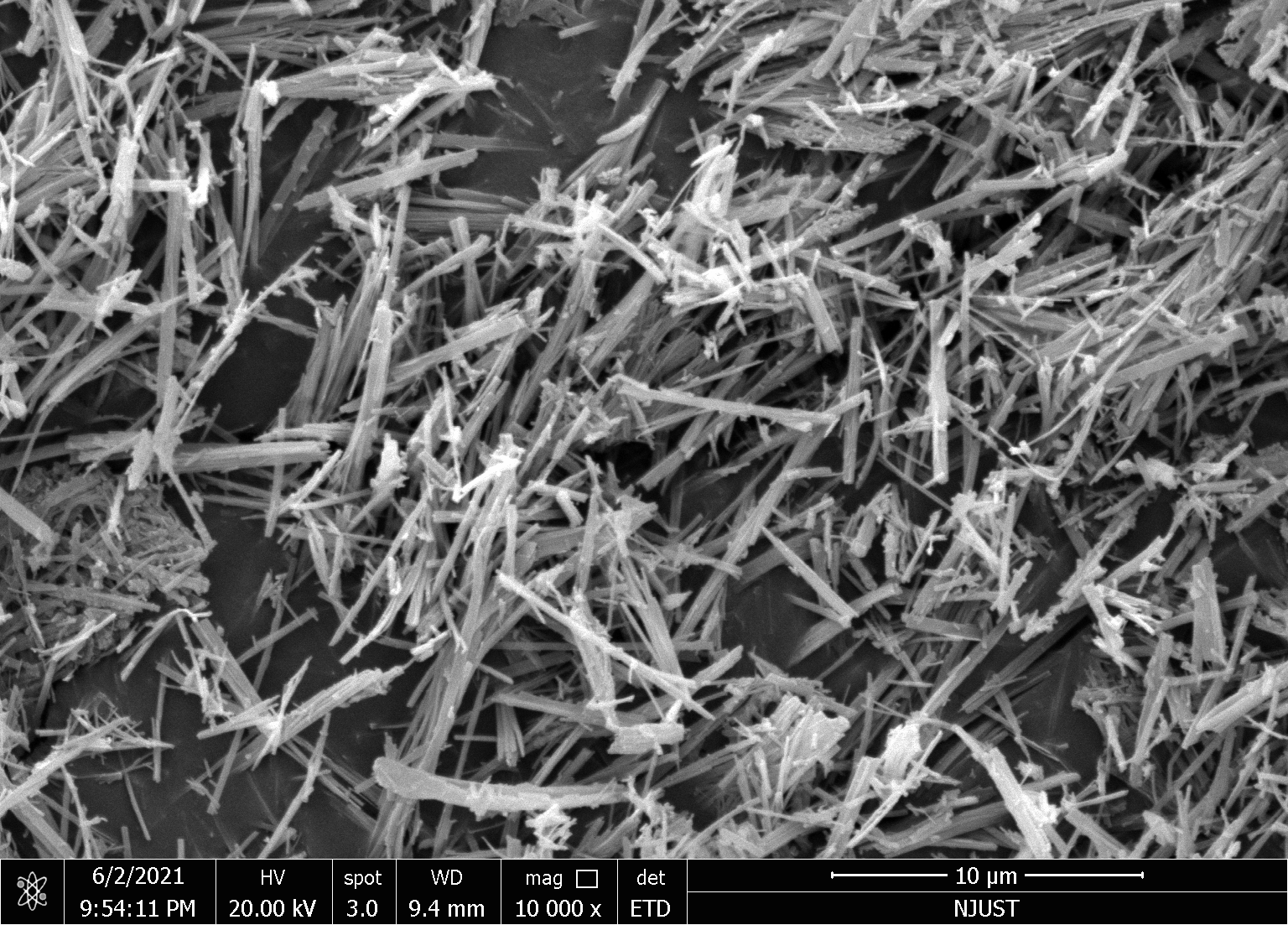

Supplement: Supplementary file 6 — Source data [file 41467_2022_32132_MOESM6_ESM.zip › Source data/supporting/S13/Figure S13b.tif]

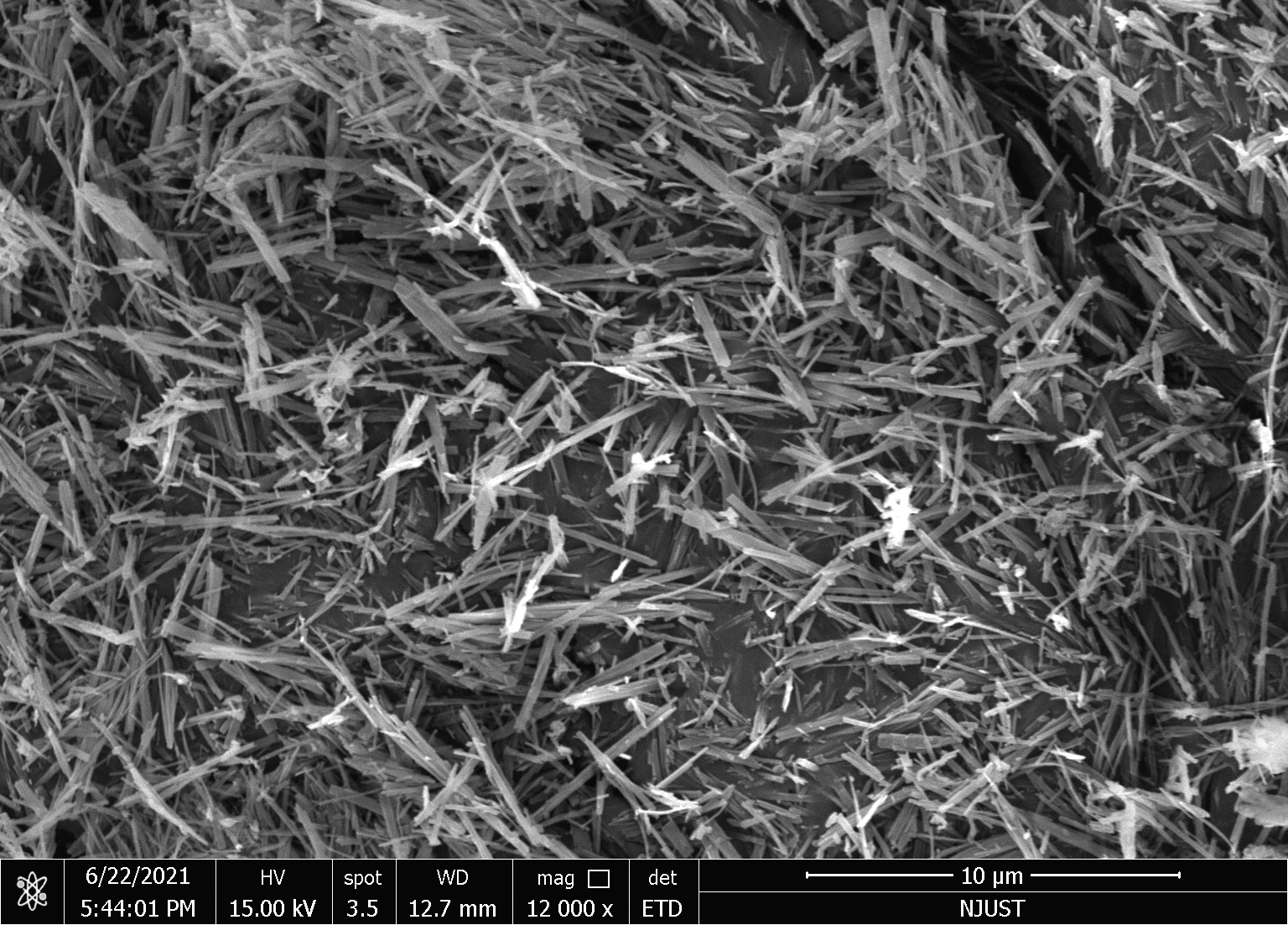

Supplement: Supplementary file 6 — Source data [file 41467_2022_32132_MOESM6_ESM.zip › Source data/supporting/S13/Figure S13c.tif]

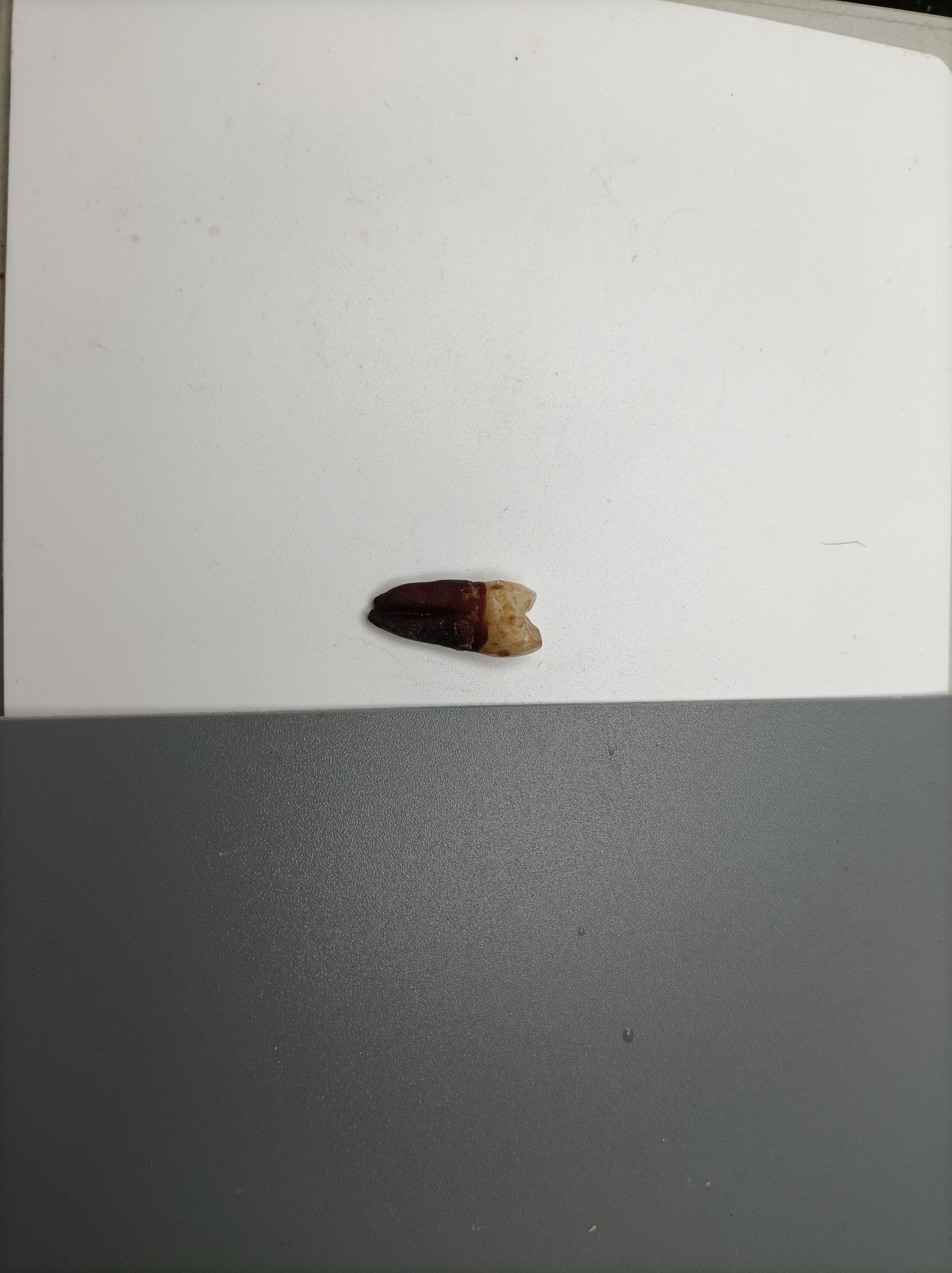

Supplement: Supplementary file 6 — Source data [file 41467_2022_32132_MOESM6_ESM.zip › Source data/supporting/S14/-10/0-2.jpg]

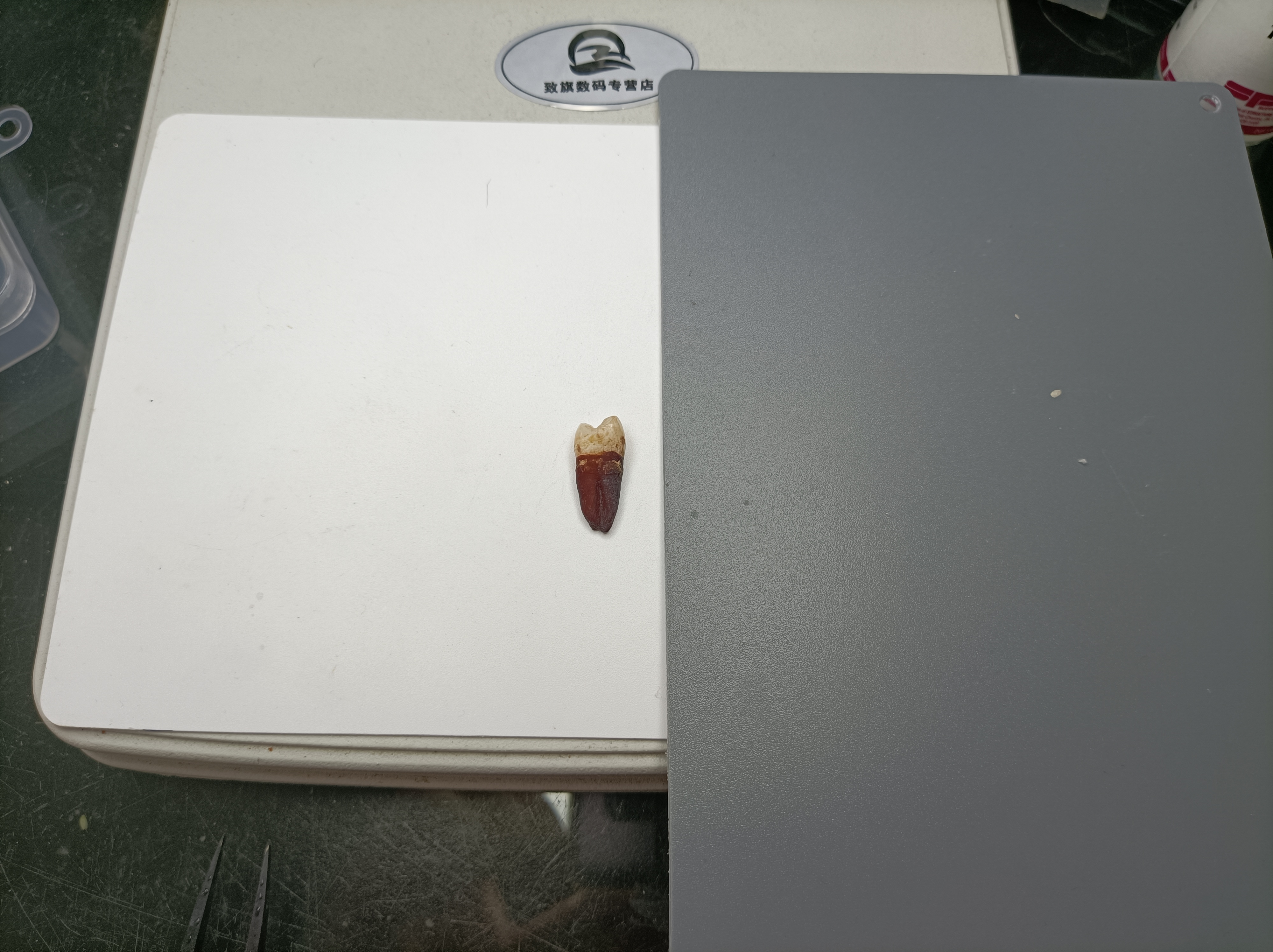

Supplement: Supplementary file 6 — Source data [file 41467_2022_32132_MOESM6_ESM.zip › Source data/supporting/S14/-10/100-2.jpg]

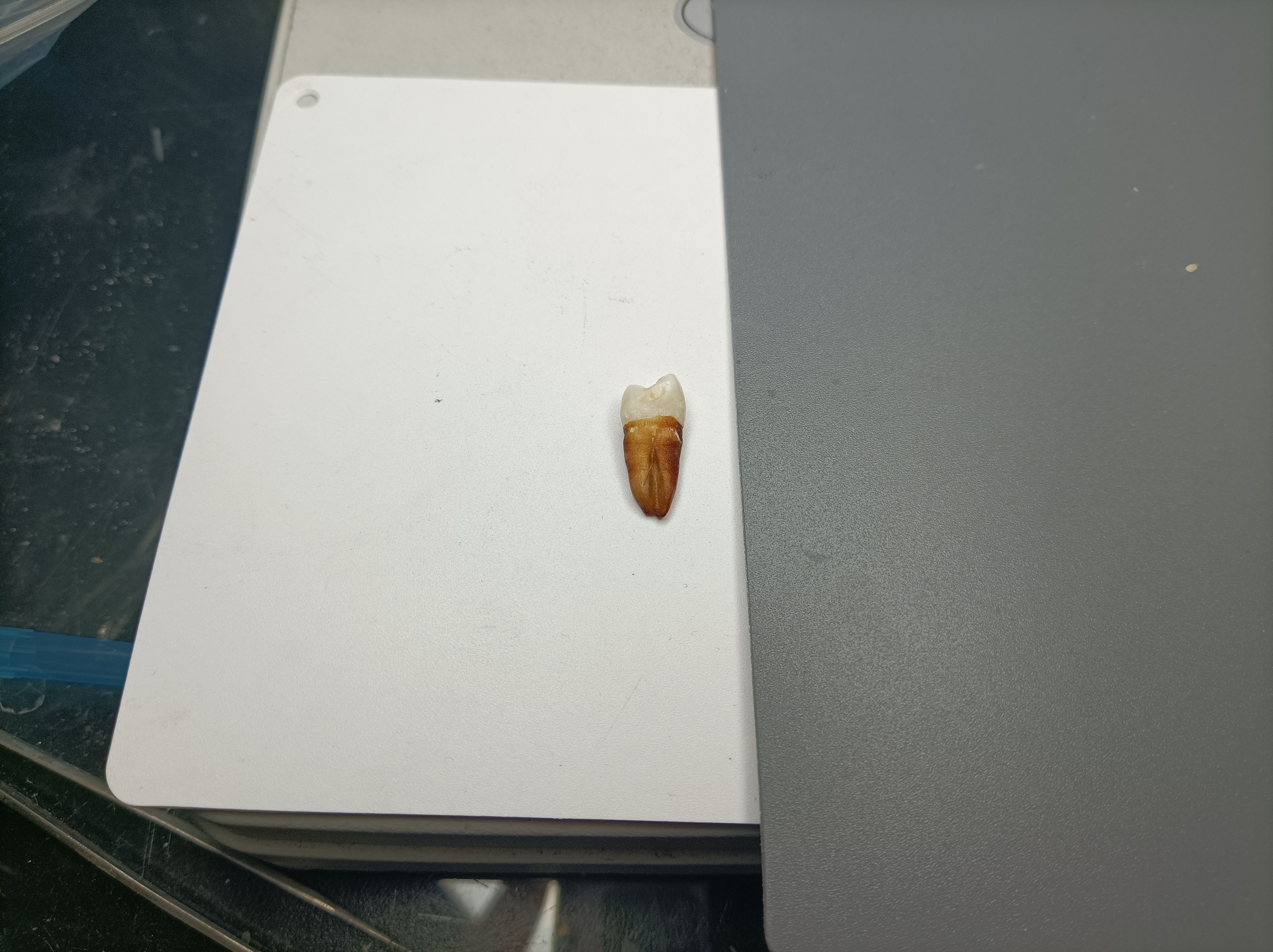

Supplement: Supplementary file 6 — Source data [file 41467_2022_32132_MOESM6_ESM.zip › Source data/supporting/S14/-10/1000-2.jpg]

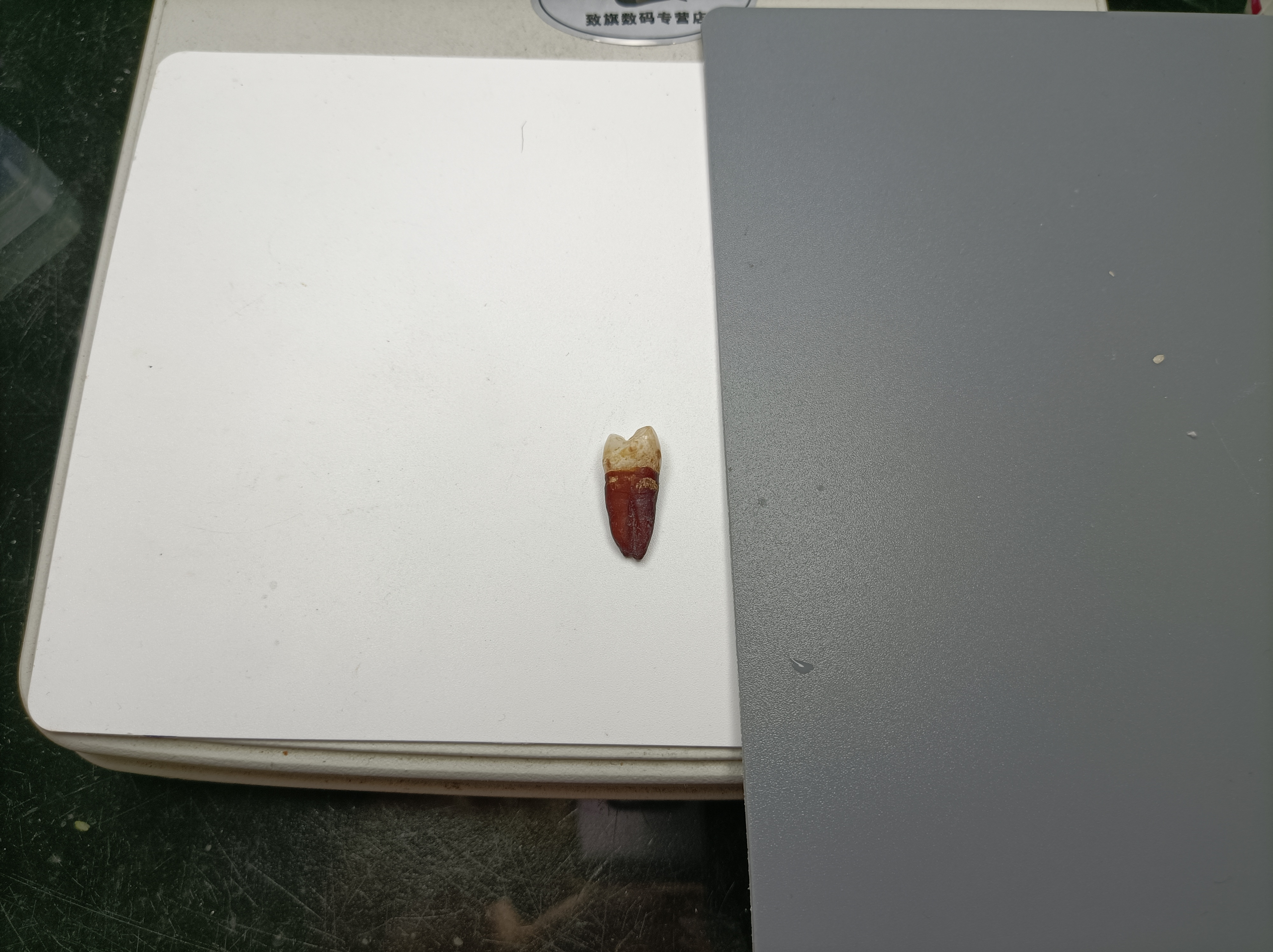

Supplement: Supplementary file 6 — Source data [file 41467_2022_32132_MOESM6_ESM.zip › Source data/supporting/S14/-10/150-2.jpg]

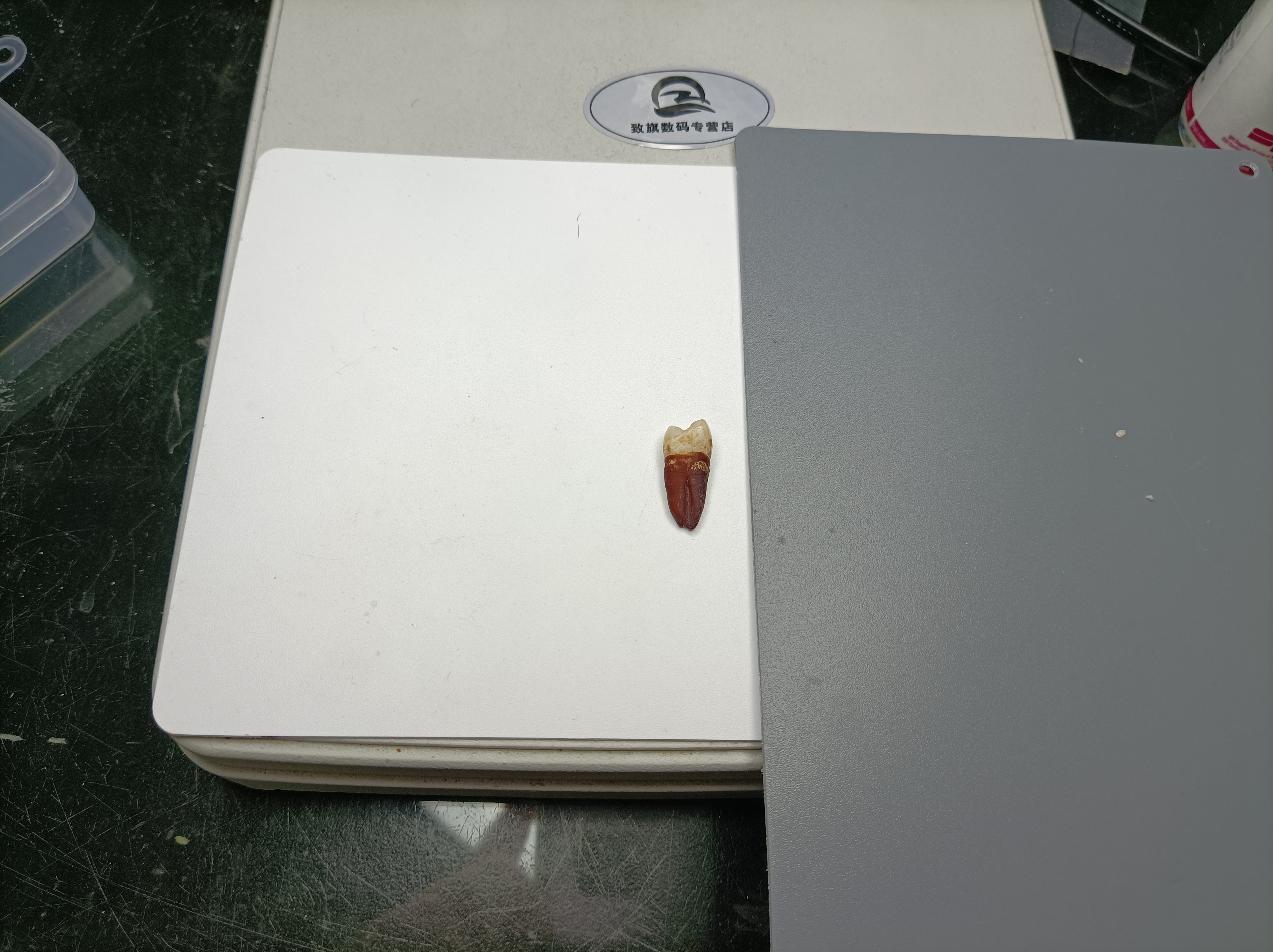

Supplement: Supplementary file 6 — Source data [file 41467_2022_32132_MOESM6_ESM.zip › Source data/supporting/S14/-10/200-2.jpg]

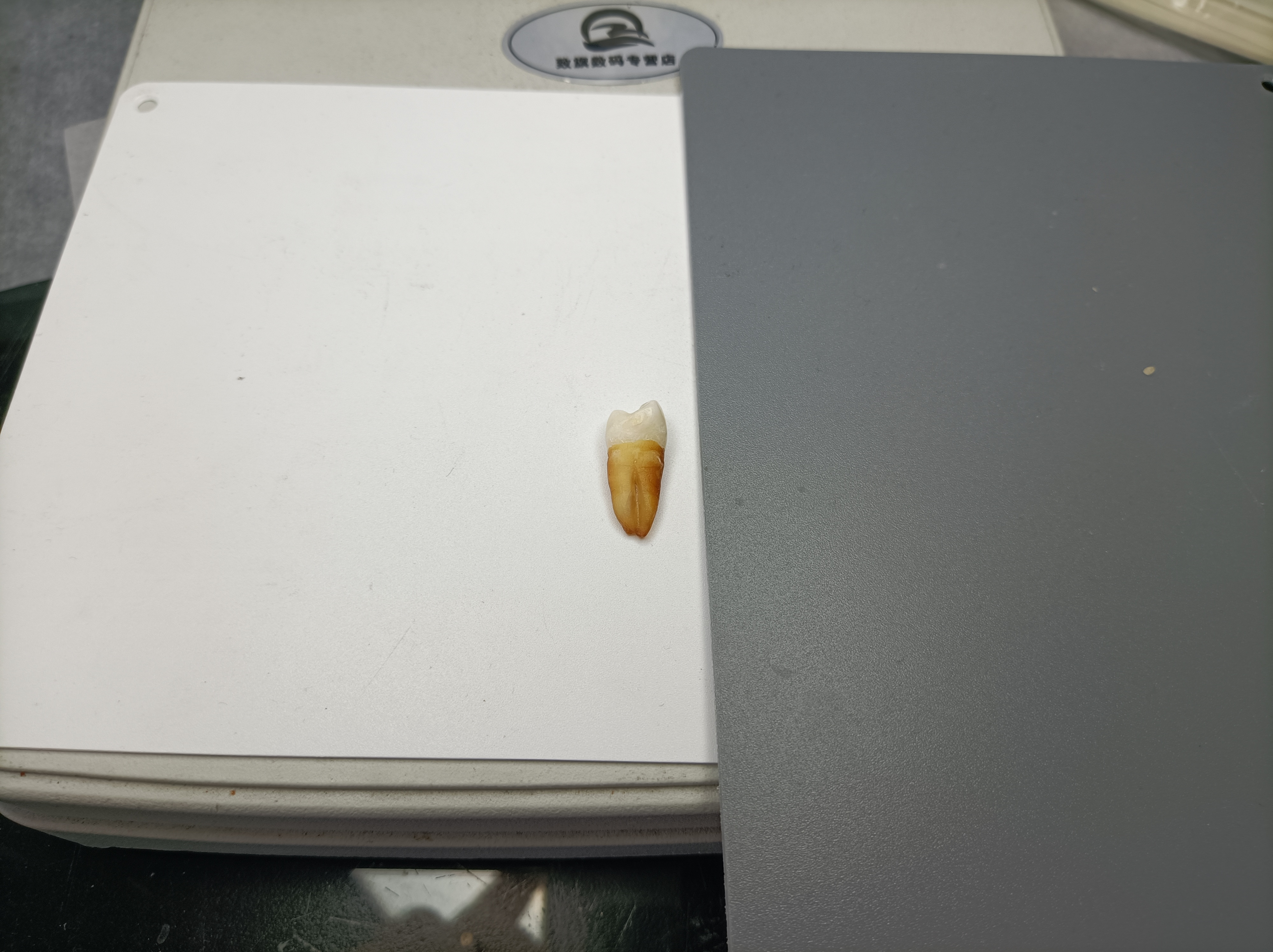

Supplement: Supplementary file 6 — Source data [file 41467_2022_32132_MOESM6_ESM.zip › Source data/supporting/S14/-10/2000.jpg]

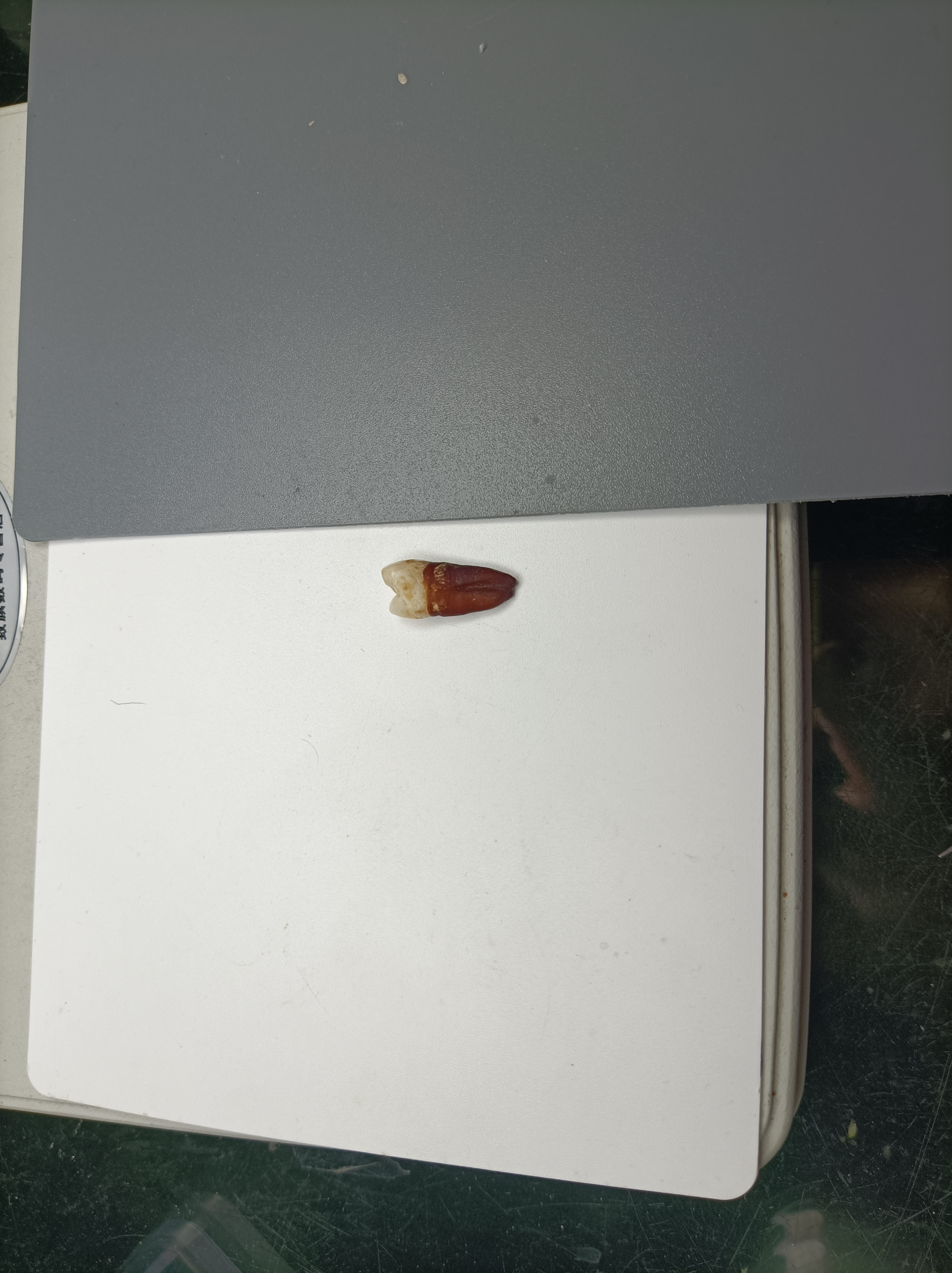

Supplement: Supplementary file 6 — Source data [file 41467_2022_32132_MOESM6_ESM.zip › Source data/supporting/S14/-10/300-2.jpg]

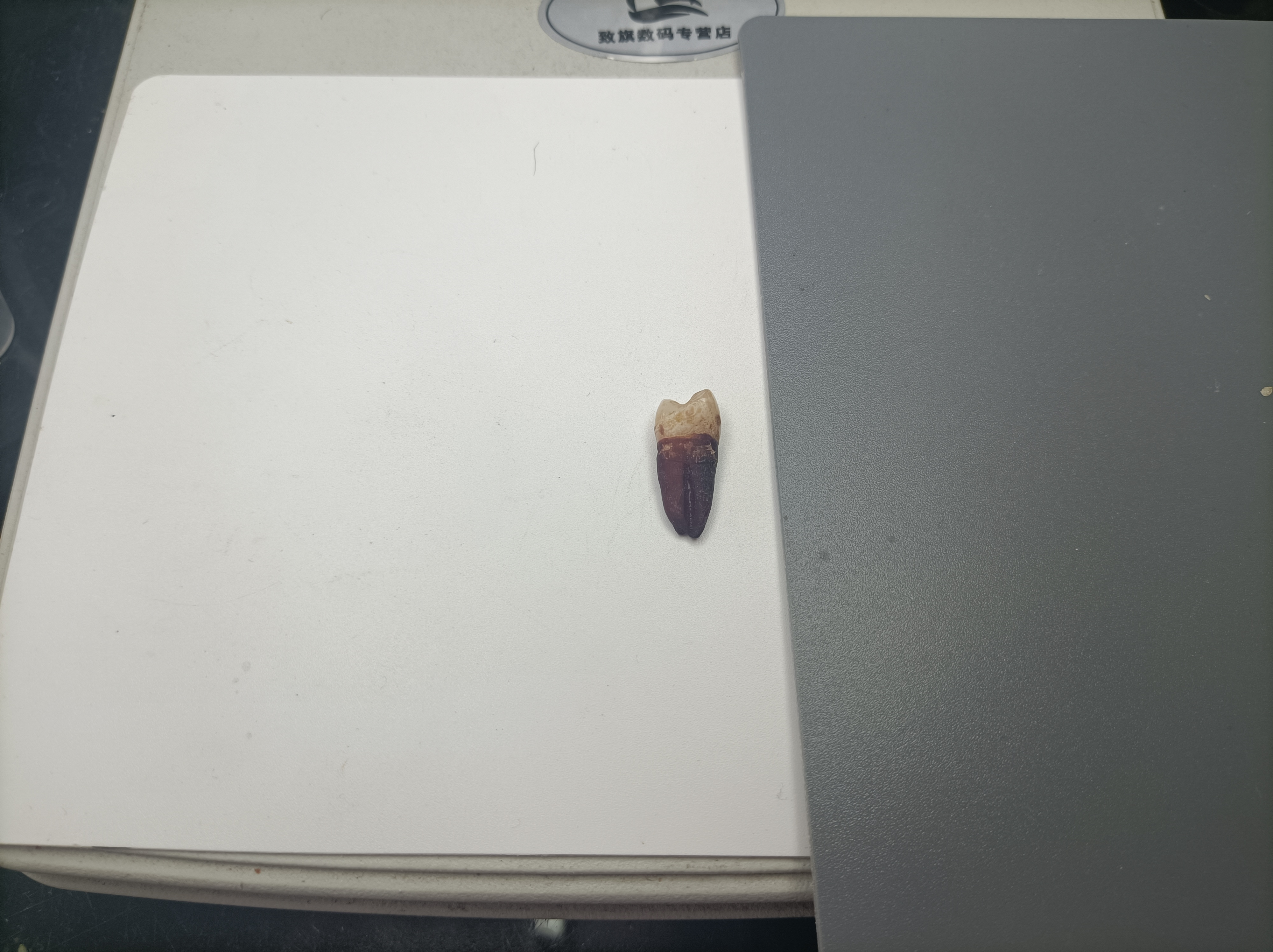

Supplement: Supplementary file 6 — Source data [file 41467_2022_32132_MOESM6_ESM.zip › Source data/supporting/S14/-10/50-2.jpg]

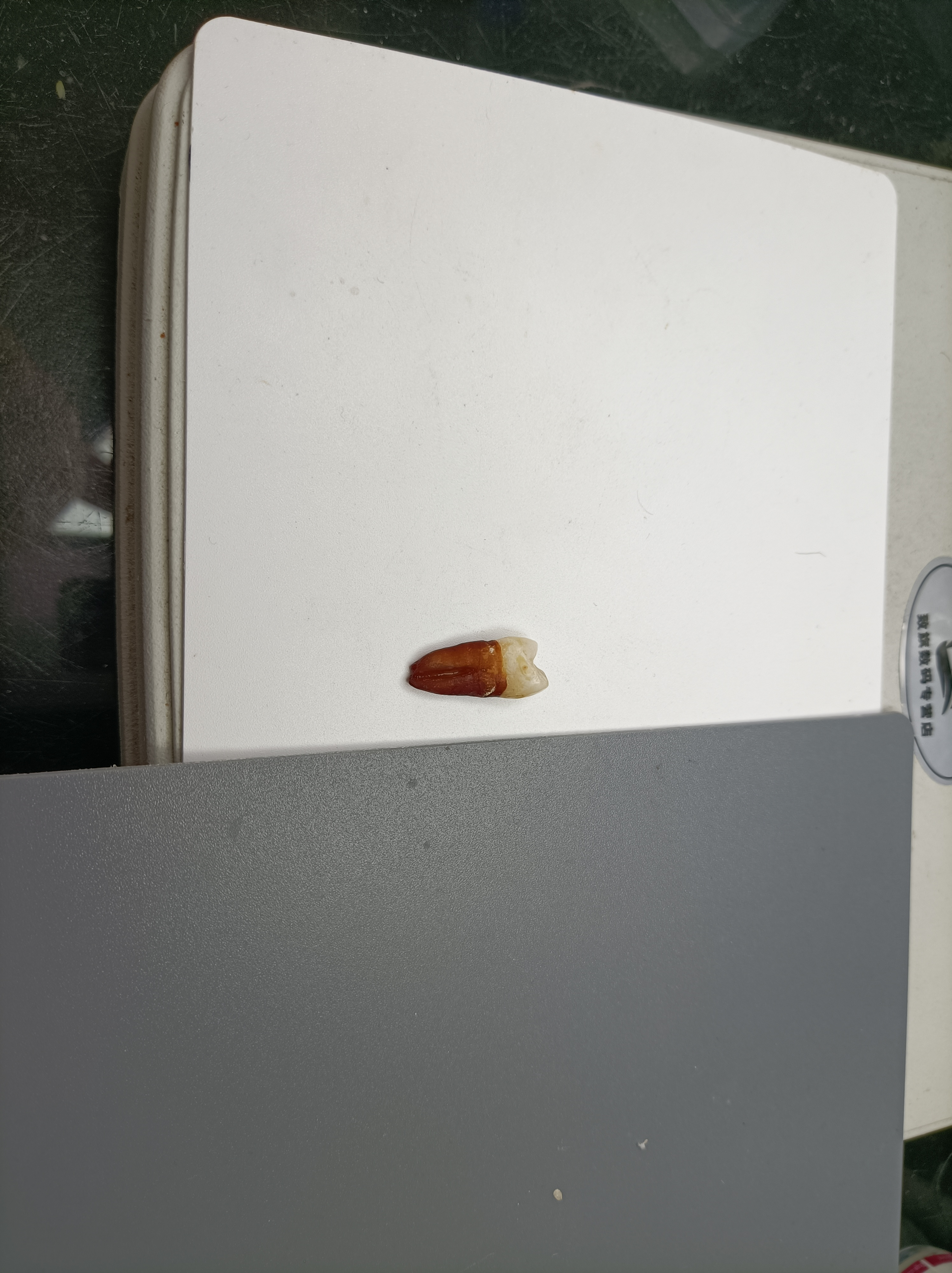

Supplement: Supplementary file 6 — Source data [file 41467_2022_32132_MOESM6_ESM.zip › Source data/supporting/S14/-10/500-2.jpg]

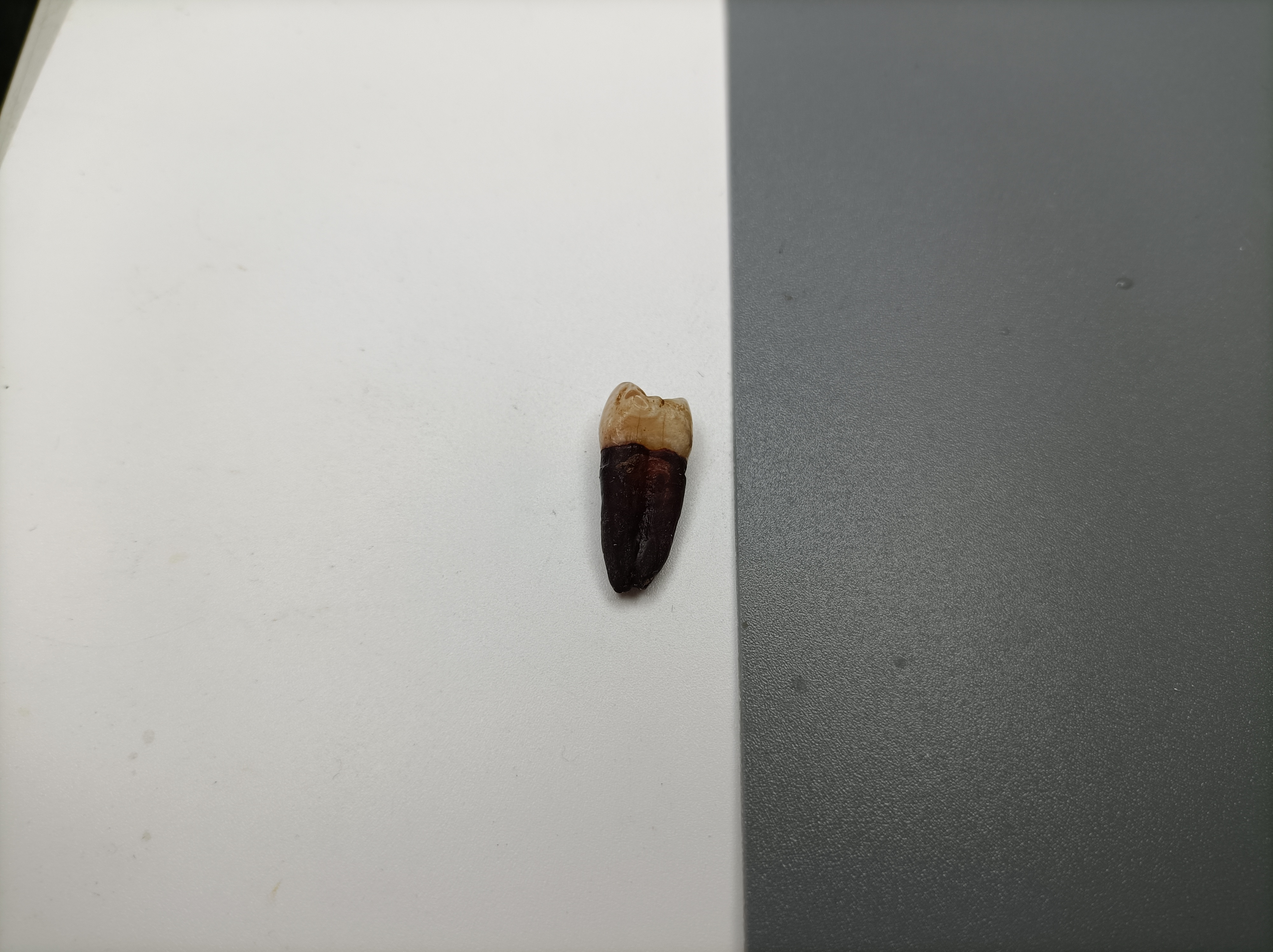

Supplement: Supplementary file 6 — Source data [file 41467_2022_32132_MOESM6_ESM.zip › Source data/supporting/S14/-10/新建文件夹/0.jpg]

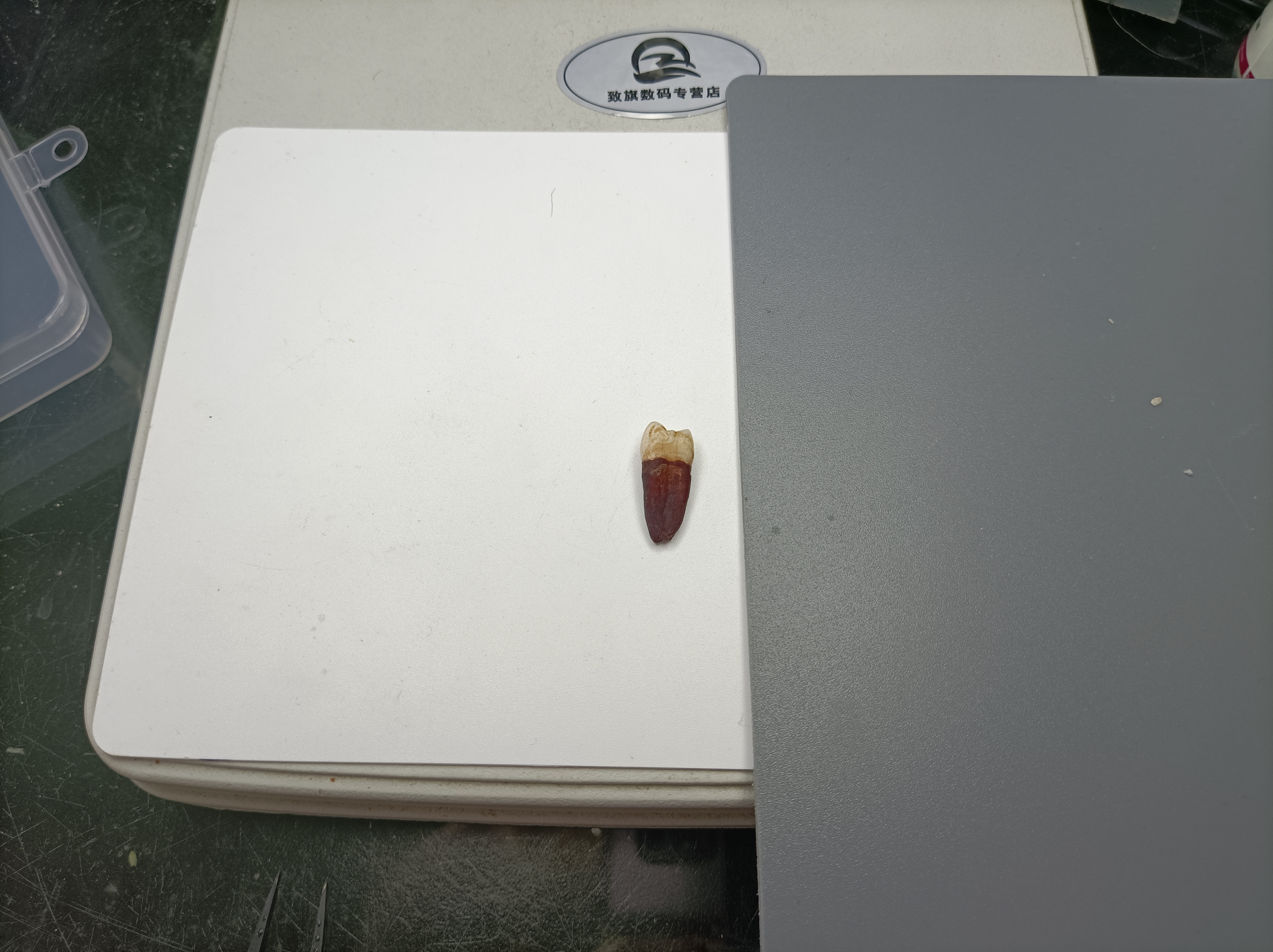

Supplement: Supplementary file 6 — Source data [file 41467_2022_32132_MOESM6_ESM.zip › Source data/supporting/S14/-10/新建文件夹/100.jpg]

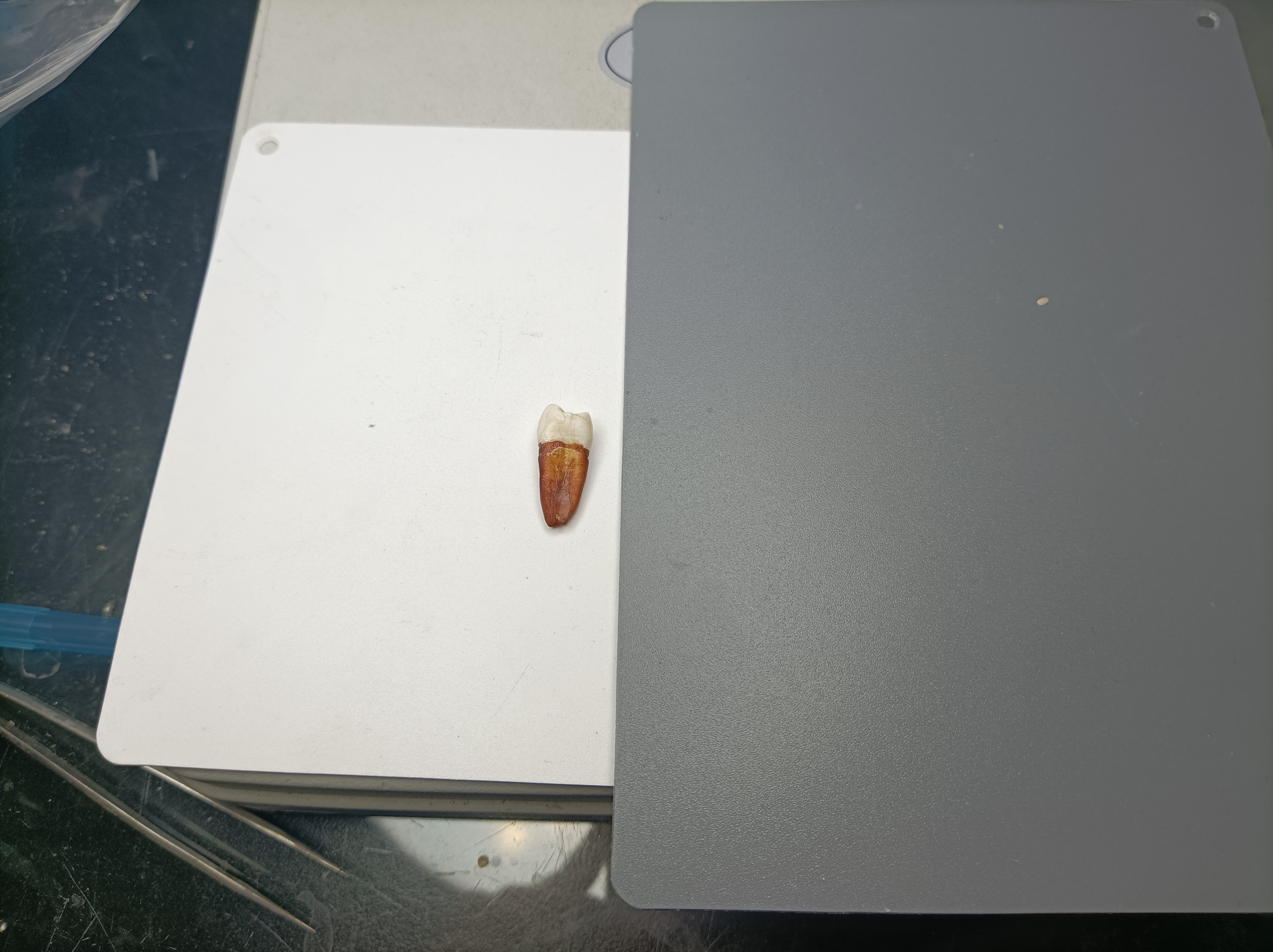

Supplement: Supplementary file 6 — Source data [file 41467_2022_32132_MOESM6_ESM.zip › Source data/supporting/S14/-10/新建文件夹/1000.jpg]

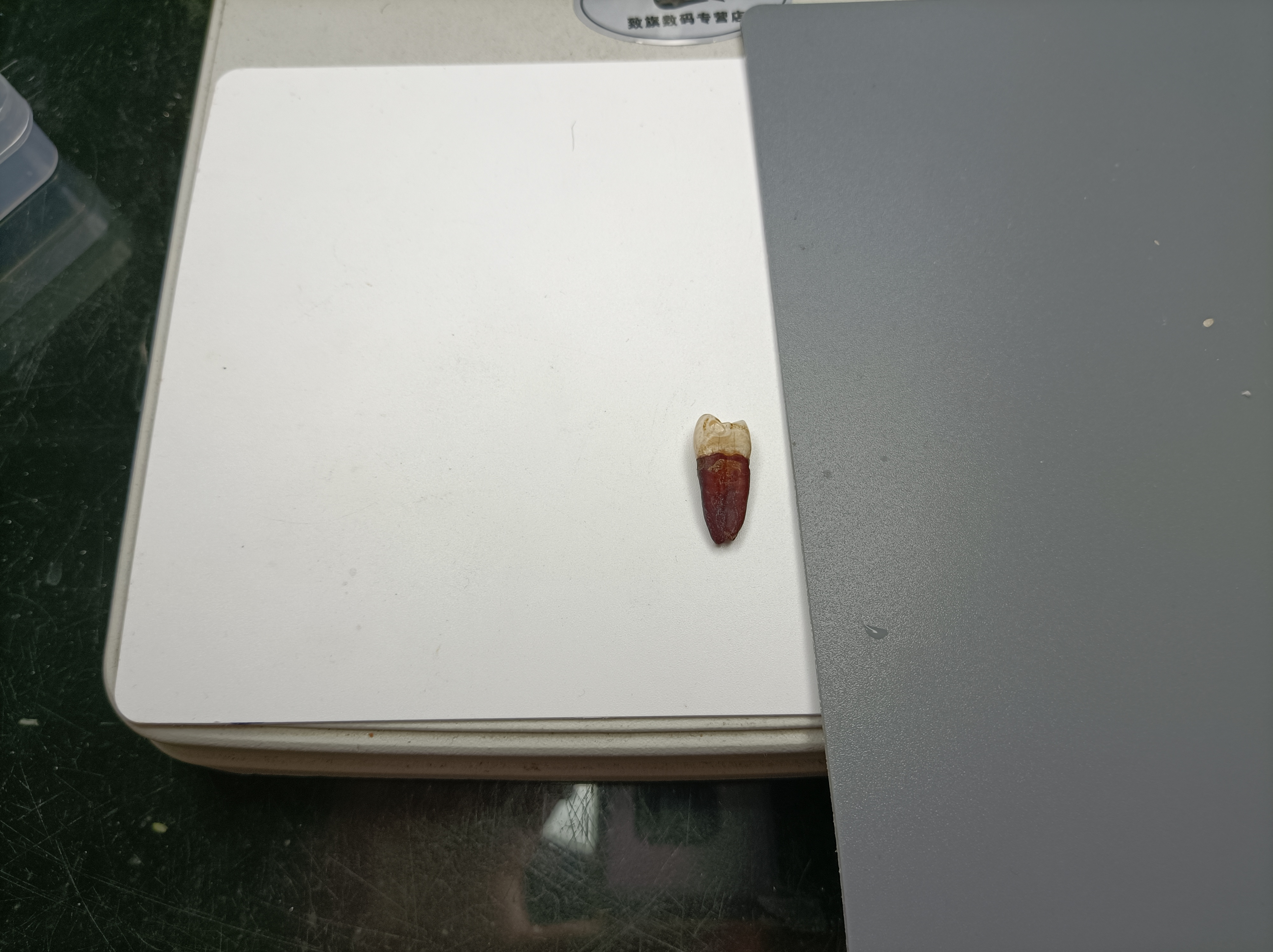

Supplement: Supplementary file 6 — Source data [file 41467_2022_32132_MOESM6_ESM.zip › Source data/supporting/S14/-10/新建文件夹/150.jpg]

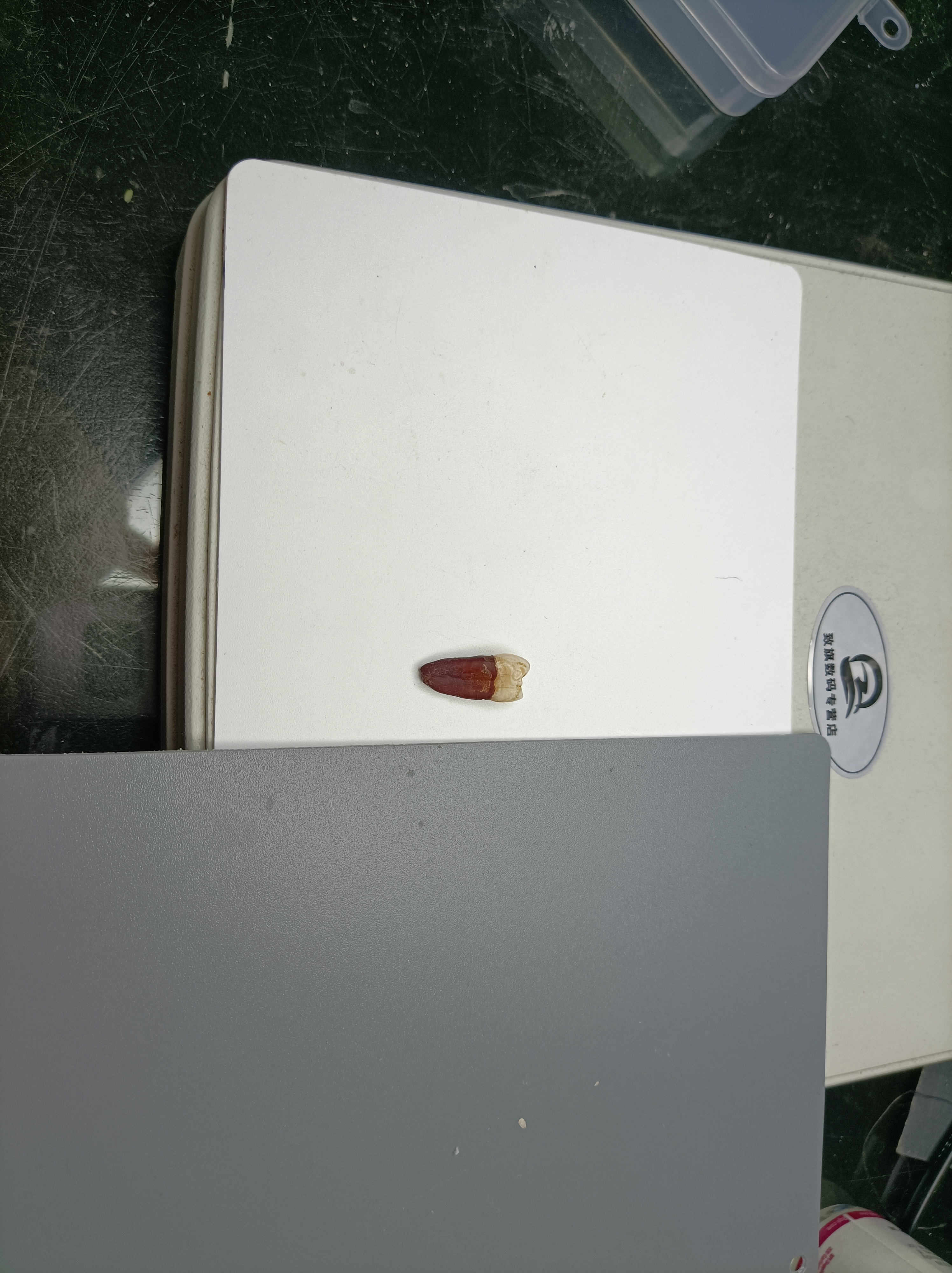

Supplement: Supplementary file 6 — Source data [file 41467_2022_32132_MOESM6_ESM.zip › Source data/supporting/S14/-10/新建文件夹/200.jpg]

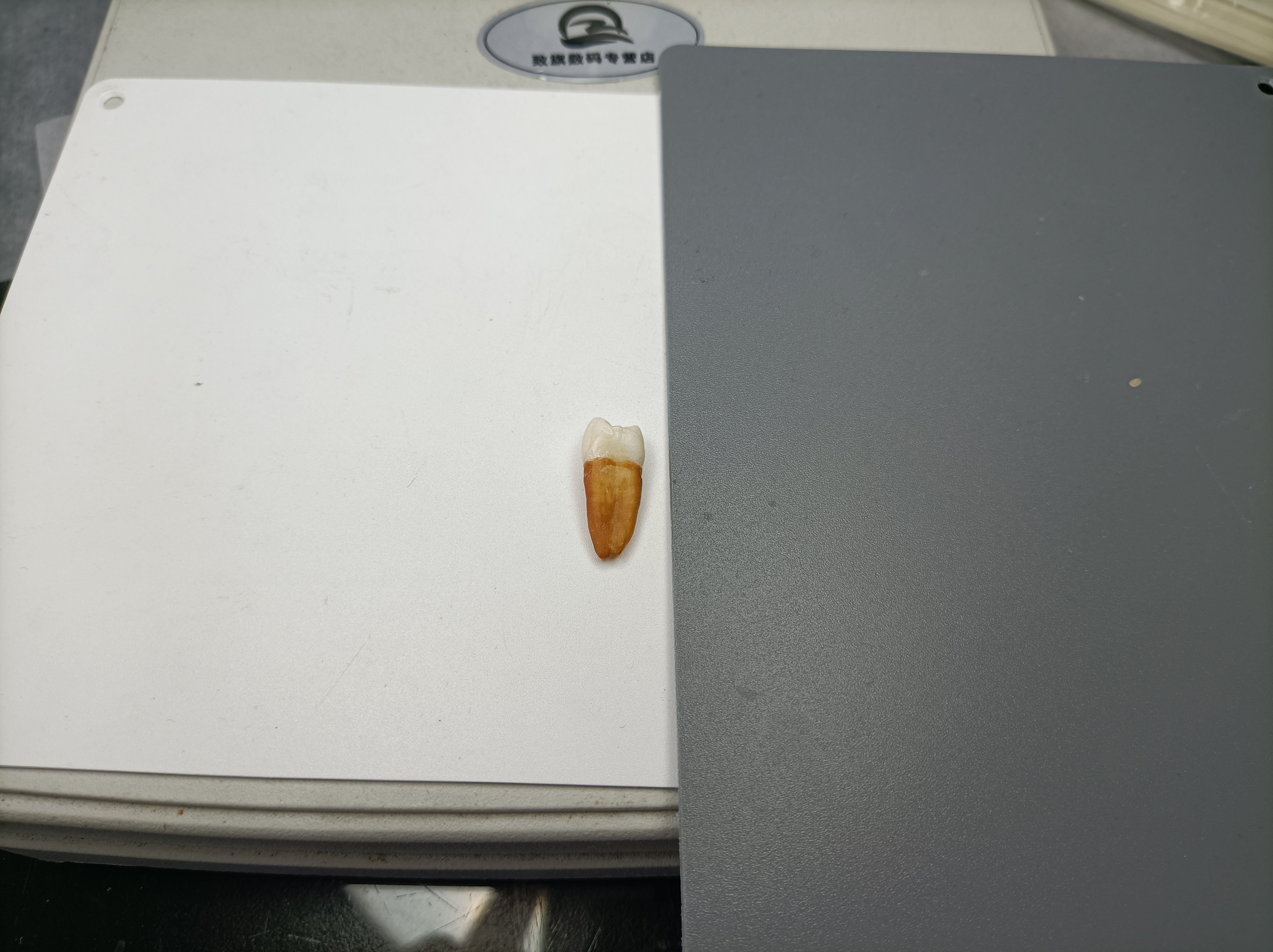

Supplement: Supplementary file 6 — Source data [file 41467_2022_32132_MOESM6_ESM.zip › Source data/supporting/S14/-10/新建文件夹/2000.jpg]

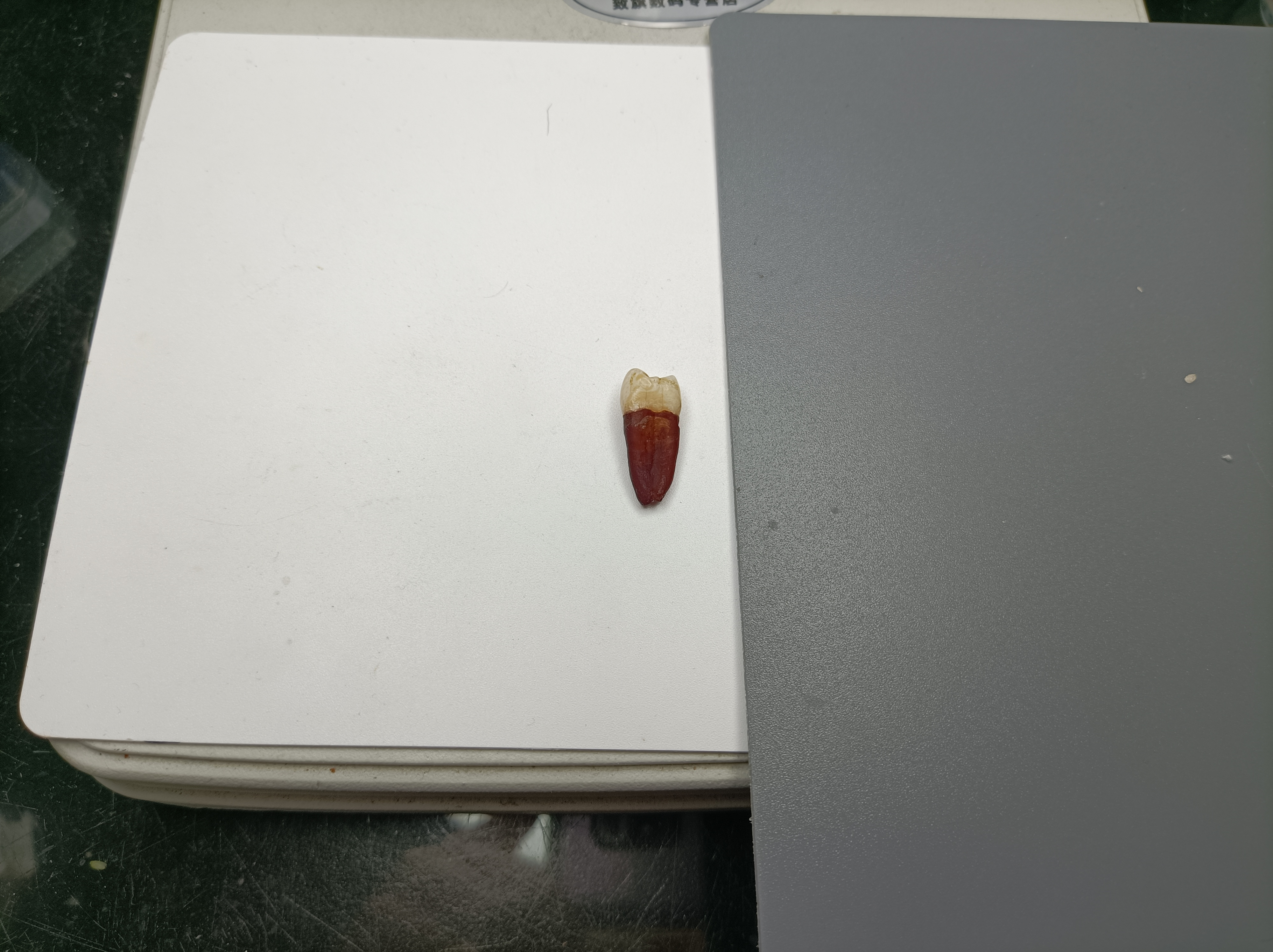

Supplement: Supplementary file 6 — Source data [file 41467_2022_32132_MOESM6_ESM.zip › Source data/supporting/S14/-10/新建文件夹/300.jpg]

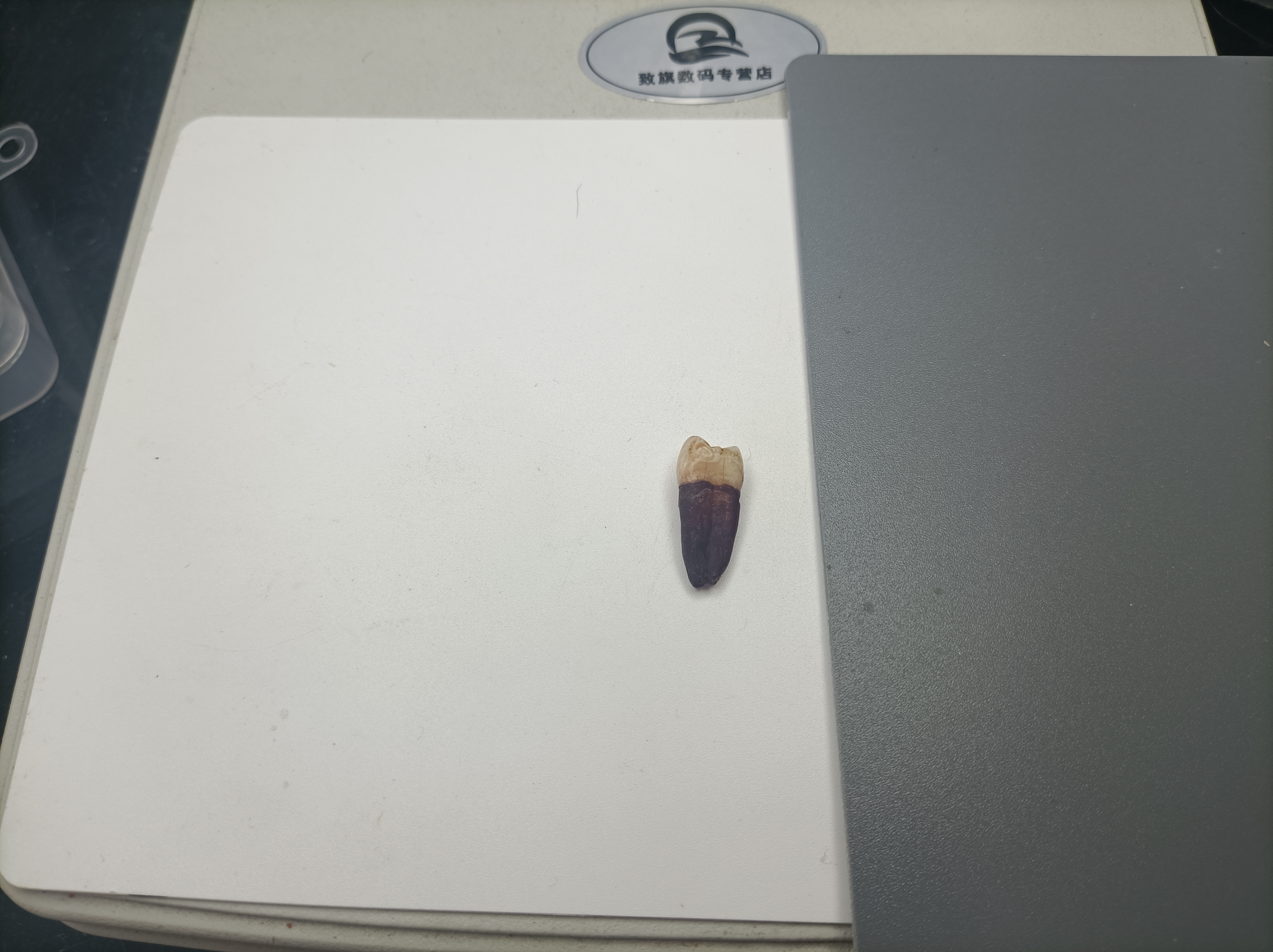

Supplement: Supplementary file 6 — Source data [file 41467_2022_32132_MOESM6_ESM.zip › Source data/supporting/S14/-10/新建文件夹/50.jpg]

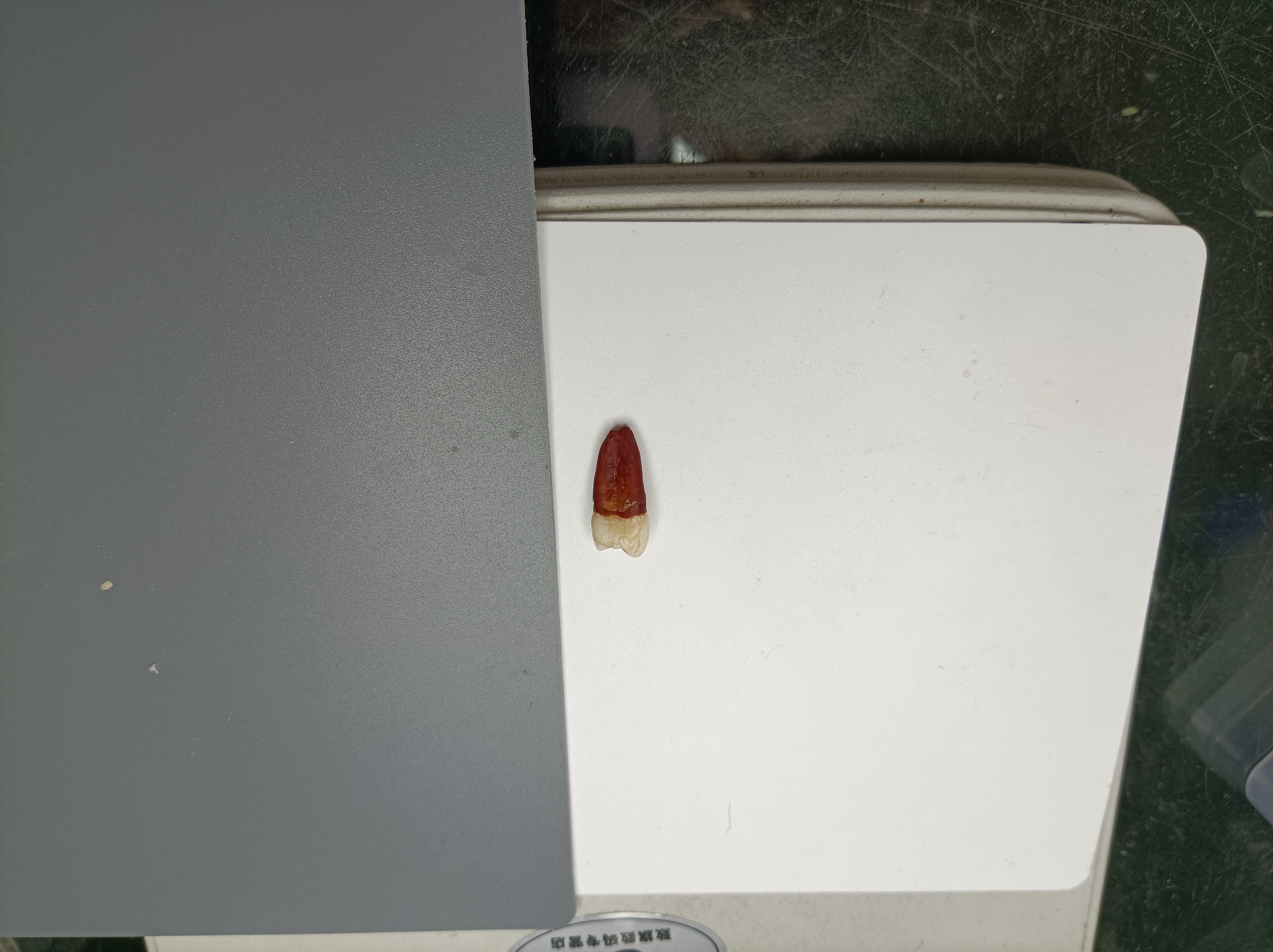

Supplement: Supplementary file 6 — Source data [file 41467_2022_32132_MOESM6_ESM.zip › Source data/supporting/S14/-10/新建文件夹/500.jpg]

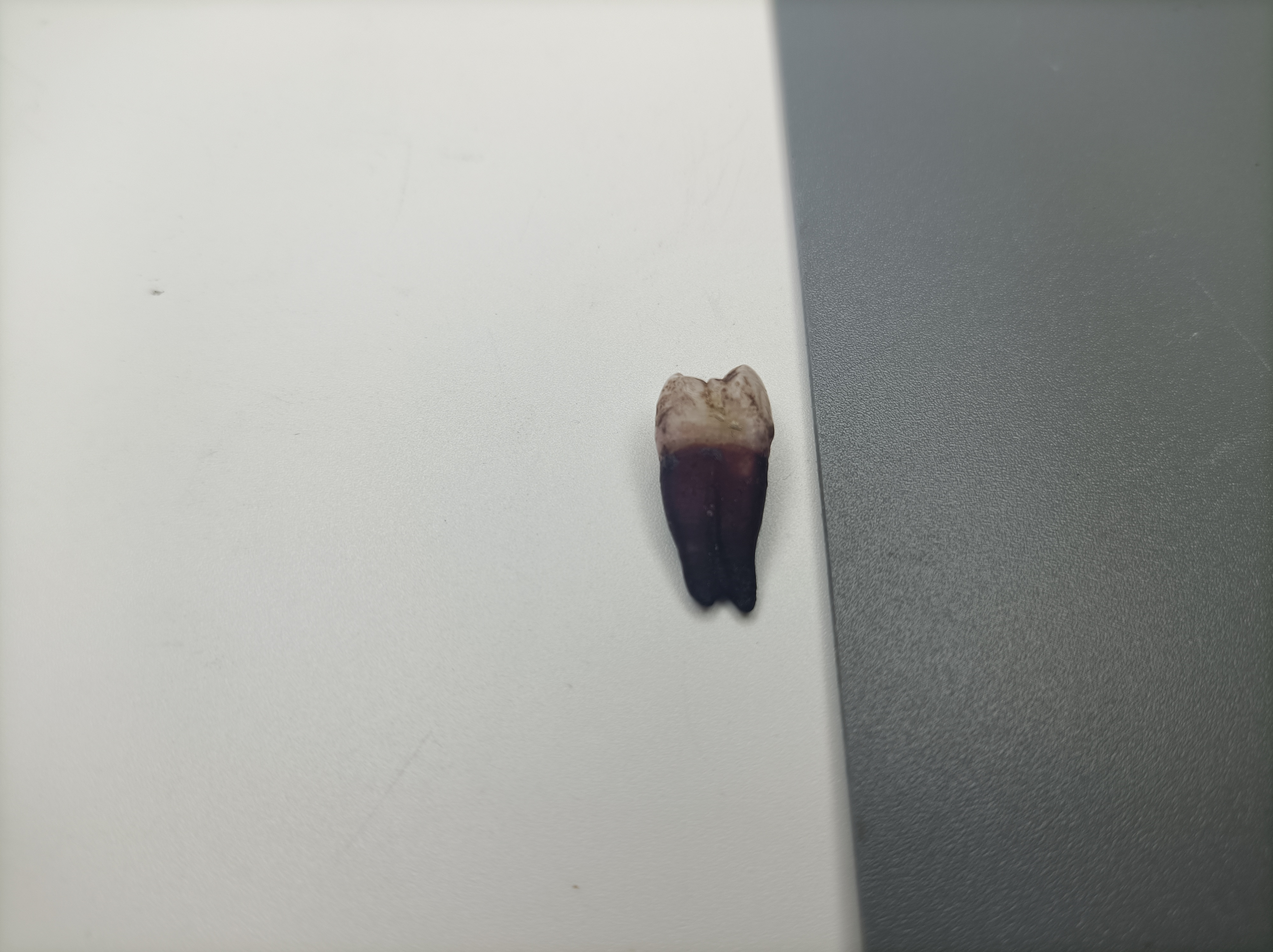

Supplement: Supplementary file 6 — Source data [file 41467_2022_32132_MOESM6_ESM.zip › Source data/supporting/S14/10/0.jpg]

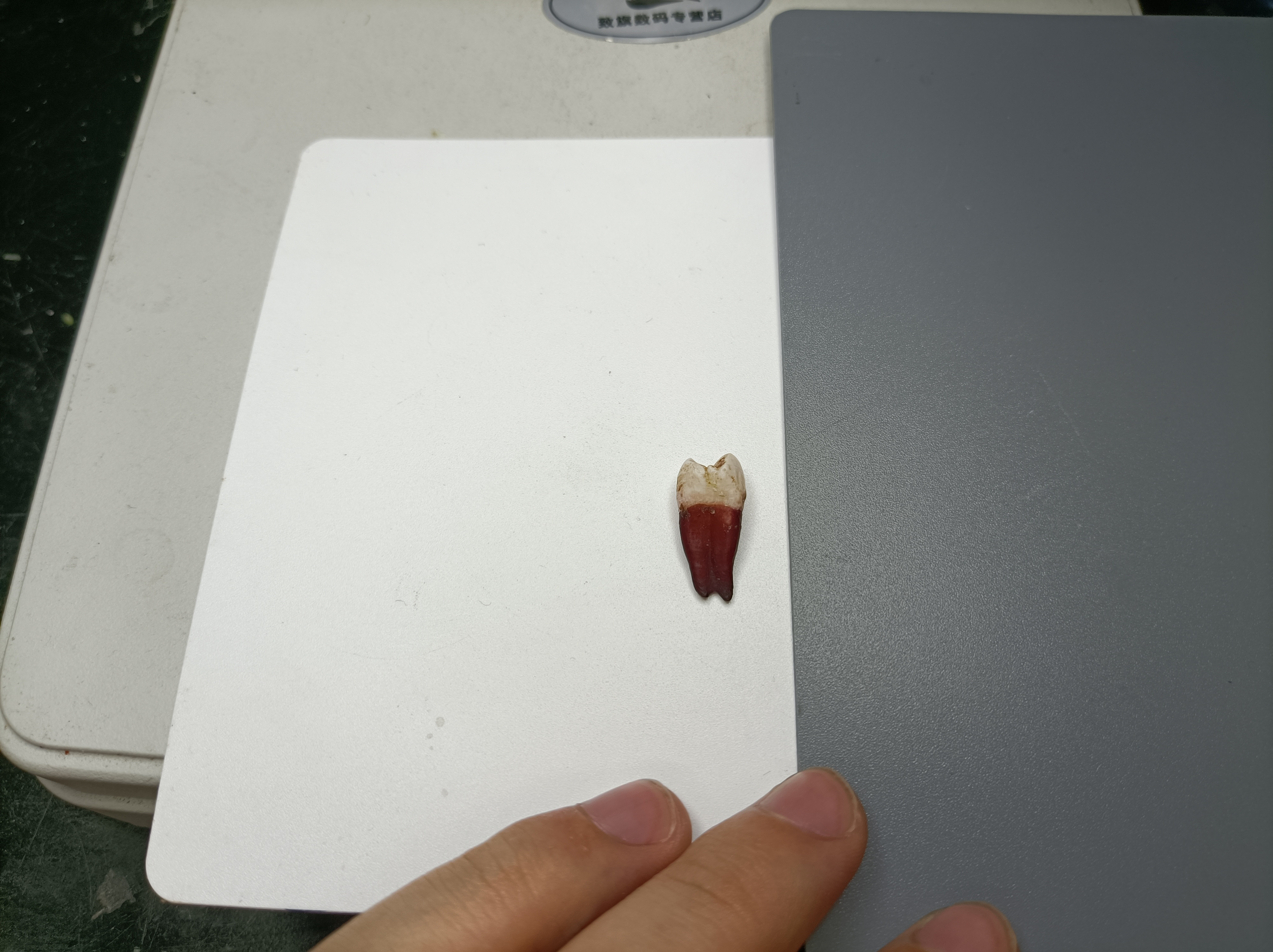

Supplement: Supplementary file 6 — Source data [file 41467_2022_32132_MOESM6_ESM.zip › Source data/supporting/S14/10/100.jpg]

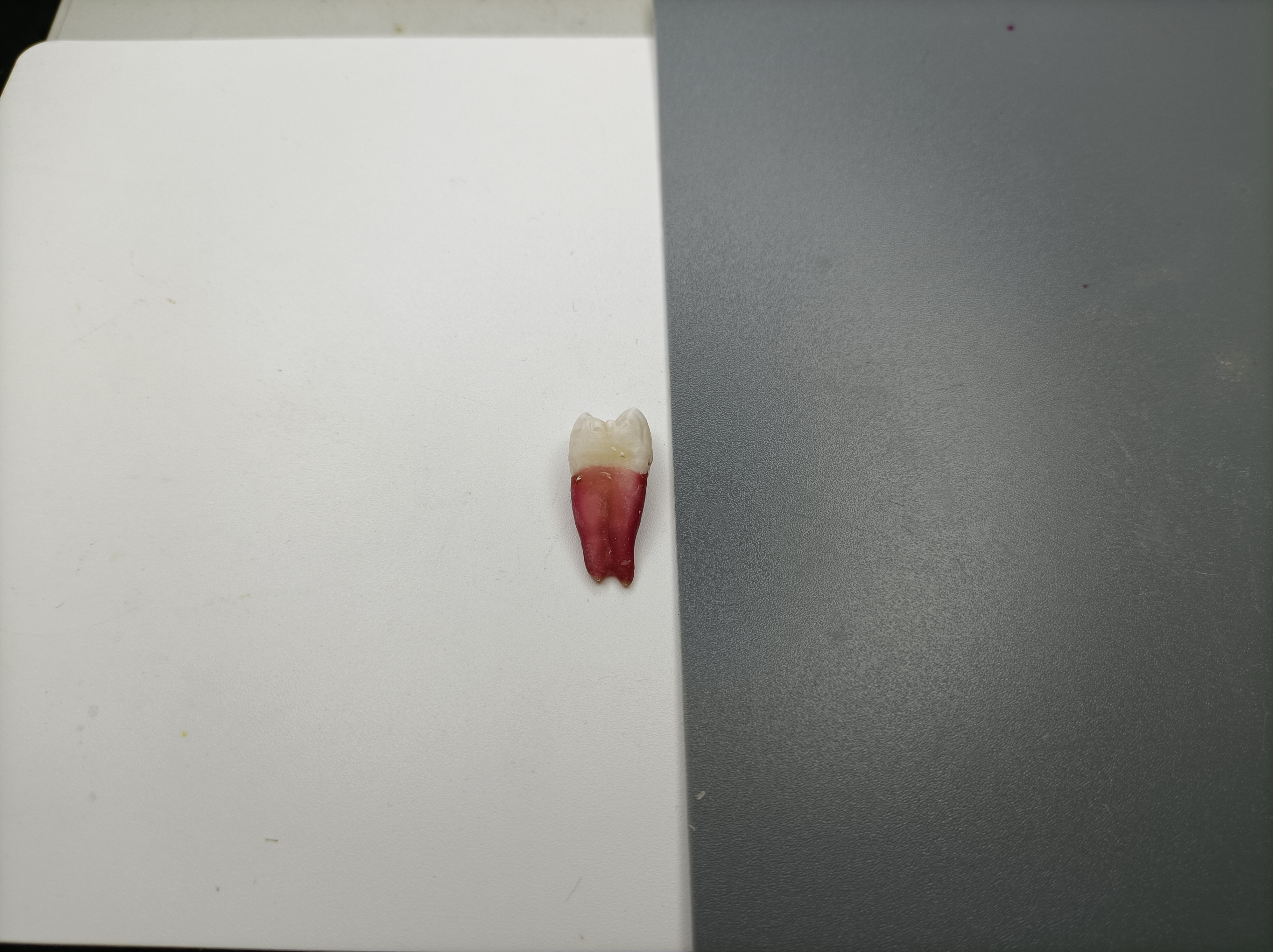

Supplement: Supplementary file 6 — Source data [file 41467_2022_32132_MOESM6_ESM.zip › Source data/supporting/S14/10/1000.jpg]

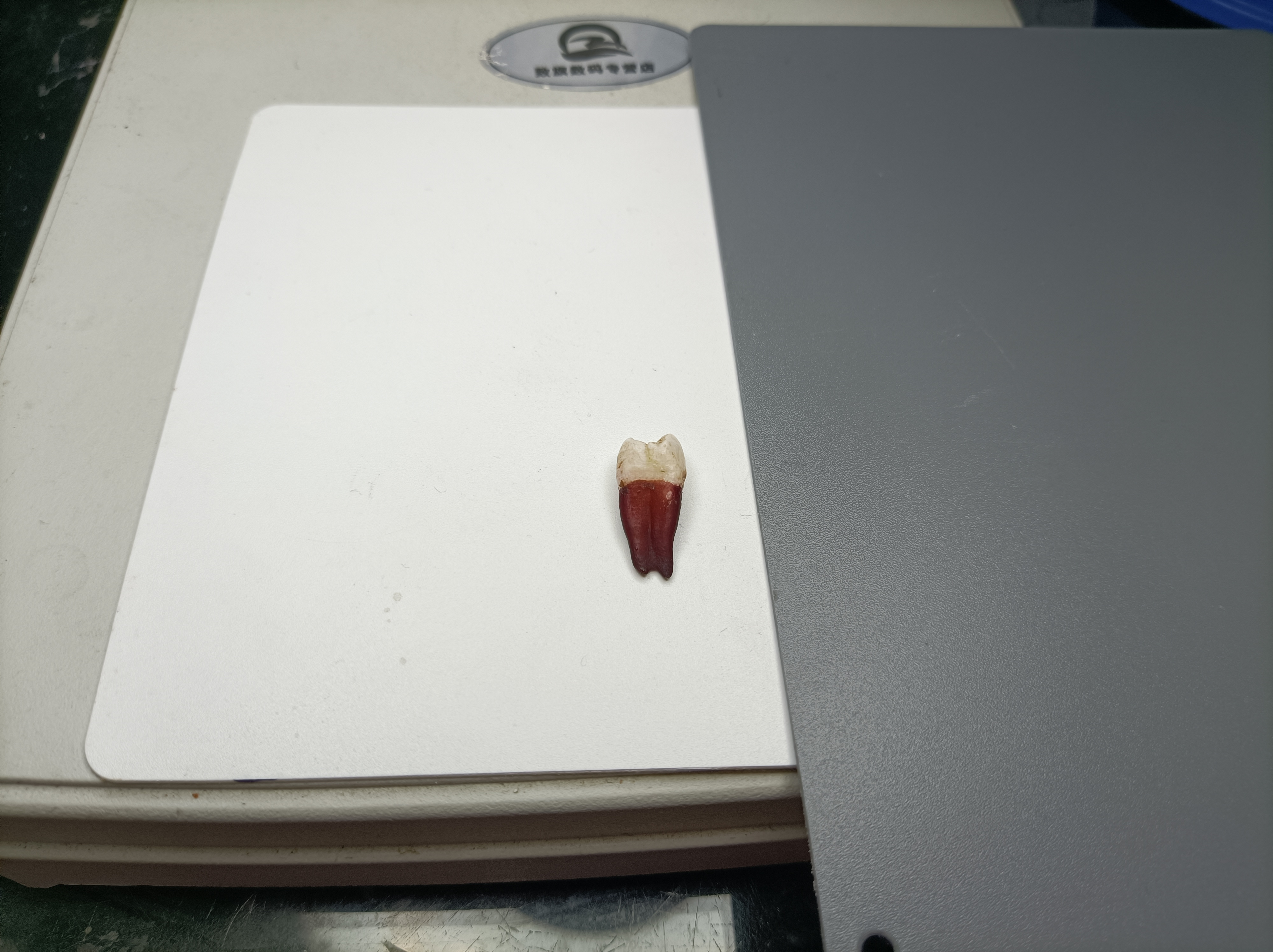

Supplement: Supplementary file 6 — Source data [file 41467_2022_32132_MOESM6_ESM.zip › Source data/supporting/S14/10/150.jpg]

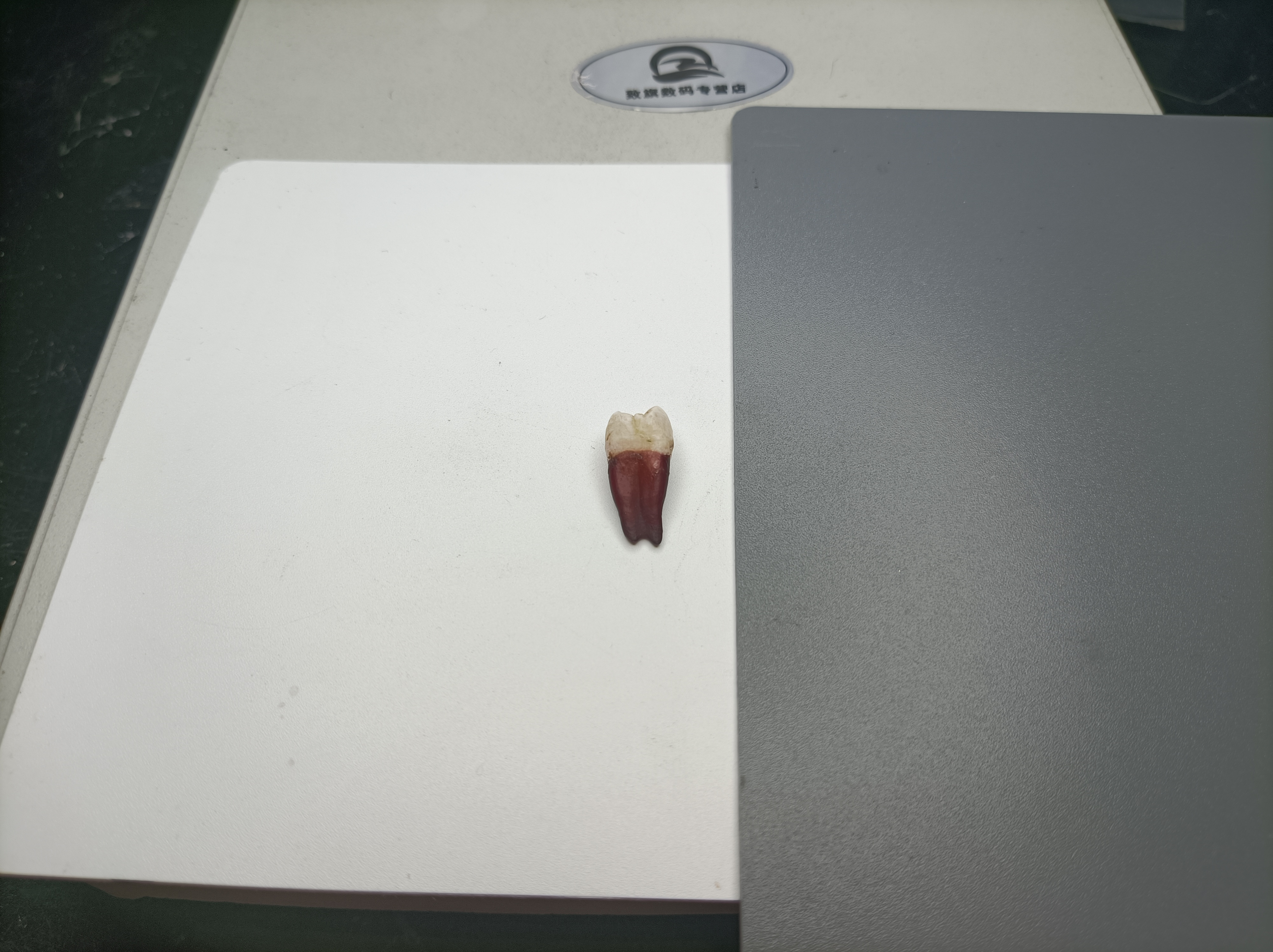

Supplement: Supplementary file 6 — Source data [file 41467_2022_32132_MOESM6_ESM.zip › Source data/supporting/S14/10/200.jpg]
